# Supplementary material for: Allylation of C‐, N‐, and O‐Nucleophiles via a Mechanochemically‐Driven Tsuji–Trost Reaction Suitable for Late‐Stage Modification of Bioactive Molecules
Source: Angew Chem Int Ed Engl. 2023 Nov 29;63(1):e202314637. doi: 10.1002/anie.202314637 (PMC10952285; doi:10.1002/anie.202314637)
Supplement: Supplementary file 1 — Supporting Information [file ANIE-63-0-s001.pdf]

Angewandte Chemie
Eine Zeitschrift der Gesellschaft Deutscher Chemiker

Supporting Information

Allylation of *C*-, *N*-, and *O*-Nucleophiles via a Mechanochemically-Driven Tsuji–Trost Reaction Suitable for Late-Stage Modification of Bioactive Molecules

*J. Templ, M. Schnürch**

Allylation of *C-, N-,* and *O*-Nucleophiles *via* a Mechanochemically-Driven Tsuji-Trost Reaction Suitable for Late-Stage Modification of Bioactive Molecules

Supporting Information

Johanna Templ[a] and Michael Schnürch[a]*

[a] J. Templ, M. Schnürch
Institute of Applied Synthetic Chemistry
TU Wien
Getreidemarkt 9/E163
1060 Vienna
AUSTRIA
*E-mail: michael.schnuerch@tuwien.ac.at

Contents

General Experimental Details 4

General Procedures 5

Optimization of Reaction Parameters 7

Control Experiments 9

Preparation of Allylic Trimethylammonium Salts 11

Nucleophile Scope 13

O-Nucleophiles 13

N-Nucleophiles 19

C-Nucleophiles 24

Allylating Agent Scope 26

NMR-Spectra 30

List of references 96

Figure 1. Optimization of reaction parameters - base 7
Figure 2. Optimization of reaction parameters - catalyst and ligand loading 7
Figure 3. Optimization of reaction parameters - milling time 8
Figure 4. NMR of the crude reaction mixtures after silica filtration. Top spectrum (blue): cinnamyl chloride is used as the allylating agent; bottom spectrum (red): allyl trimethylammonium chloride is used as the allylating agent. The bottom spectrum shows full conversion and solely product formation (cf. NMR-spectrum of compound 44) 10

List of Tables

Table 1. Optimization of Reaction Conditions – base 7
Table 2. Optimization of Reaction Conditions – catalyst and milling time 7
Table 3. Optimization of Reaction Conditions – milling time 8
Table 4. Optimization of Reaction Conditions – chiral ligand screening 8
Table 5. Control experiments – reactions in solution 9
Table 6. Control experiments – reactions using allyl bromide as the allylating agent 9

General Experimental Details

All Chemicals were purchased from commercial suppliers and, unless noted otherwise, used without further purification.

For small-scale reactions at a 0.5 mmol scale in solution, the used 8 mL glass vials were sealed with Wheaton® screw caps containing a PTFE faced 14B styrene-butadiene rubber liner for small-scale reaction above room temperature and heated in a metallic reaction block. All reaction temperatures refer to external temperatures.

Mechanochemical reactions were conducted in an IST636 mixer mill, using a Teflon milling jar (7 mL) and 2 ZrO_2 milling balls (one with 7 mm diameter, one with 10 mm diameter) at a frequency of 30 Hz under ambient conditions without external heating. Reaction vessels were purchased from InSolido Technologies.

^{1}H NMR, ^{13}C NMR, and ^{19}F NMR spectra were recorded on a Bruker Avance UltraShield 400 at ambient temperature. Chemical Shifts (δ) are reported in ppm. Coupling constants (J) are given in Hertz (Hz) and multiplicities are assigned as s (singlet), d (doublet), t (triplet), q (quartet), m (multiplet), dd (doublet of doublets), td (triplet of doublets), and dt (doublet of triplets).

Column chromatography was performed on standard manual glass columns using Merck silica gel 60 (40 µm – 63 µm). Thin Layer Chromatography (TLC) analysis was performed on aluminum-backed unmodified Merck silica gel 60 F_{245} plates. Visualization was realized under UV irradiation or via heat staining using a ceric ammonium molybdate aqueous solution.

HR-MS analysis was performed using HTC PAL system auto sampler, an Agilent 1100/1200 HPLC and Agilent 6230 AJS ESI-TOF mass spectrometer. Data evaluation was performed using Agilent MassHunter Qualitative Analysis B.07.00. Identification was based on peaks obtained from extracted ion chromatograms (extraction width ± 20 ppm).

Chiral HPLC measurements were carried out on a DIONEX UPLC equipped with a photodiode array (PDA) plus detector (190–360 nm), using CHIRALCEL OD column (0.46 cm diameter, 25 cm length)

General Procedures

General Procedure A – Optimization screening

4-Hydroxybiphenyl (I) (85 mg, 0.5 mmol, 1 equiv.), allyl trimethylammonium chloride (II), the respective base (K_2CO_3 or Cs_2CO_3), Pd[(allyl)Cl]$_2$, and *rac*-BINAP were placed in a 7 mL Teflon milling jar, equipped with two ZrO_2 milling balls, one with 7 mm diameter, one with 10 mm diameter. The two different sizes of milling balls were chosen, since it was observed that two 7 mm milling balls got stuck relatively quickly in the milling process. The closed vessel was mounted into the holding station of the mixer mill and milling was conducted at 30 Hz for the indicated time. After the reaction, the crude material was washed out with dichloromethane (DCM), filtered over a short plug of silica in cotton-stuffed Pasteur pipette, eluted with small amounts of DCM (2-4 mL) and the solvent was subsequently removed under reduced pressure. The residue was purified *via* column chromatography (dry load on celite, 5 g silica, LP:EA 50:1).

General Procedure B – synthesis of the allyl trimethyl ammonium salts

Procedure B1 – chlorination of allylic alcohol groups

To a stirred solution of the respective allylic alcohol (1 equiv.) in pentane (1.5 M) was added concentrated hydrochloric acid (3 equiv., 37 %) under vigorous stirring at 0 °C. As soon as the addition of the acid was completed, the reaction was allowed to warm to room temperature and stirred for the a given time. 5 mL of water were added, and product was extracted 3 times with each 20-30 mL pentane. The combined organic extracts were washed 4 times with each 10-20 mL sat. $NaHCO_3$ solution, once with brine, dried over Na_2SO_4, filtered and concentrated at 50 °C water bath temperature and atmospheric pressure. The crude product was used for the next step without further purification and with small amounts of pentane still present. *It is important to note that no acid should be present in the crude mixture to prevent a formation of trimethylamine hydrochloride salt in the subsequent quaternization step.*

Procedure B2 – quaternary ammonium salt formation from the respective allyl chloride derivative

Either commercially available allylic chlorides were used directly or the crude reaction mixtures from the previous chlorination step (procedure B1) were used without further purification.
A round bottom flask was charged with the allylic chloride and a 4.2 M solution of trimethylamine in EtOH (2.5 equiv.) was added dropwise *via* a syringe at 0 °C. The reaction was stirred for 18 hours at room temperature. Subsequently, all volatiles were evaporated under reduced pressure. The obtained off-white solid was dispersed in pentane, filtered, washed several times with pentane and finally dried *in vacuo* to obtain the quaternary allylic ammonium chloride. *It has to be noted that the ammonium salts are highly hygroscopic and suction filtration should be conducted fast without any prolonged exposal to air.*

General Procedure C – for the preparation of the nucleophile scope:

A Teflon milling jar (7 mL) equipped with two ZrO_2 milling balls, one with 7 mm diameter, one with 10 mm diameter, was charged with the respective nucleophile (0.5 mmol, 1 equiv.), the allyl trimethylammonium chloride (76 mg, 0.55 mmol, 1.1 equiv.), Pd[(allyl)Cl]$_2$ (0.95 mg, 0.5 mol%), *rac*-BINAP (3.11 mg, 1 mol%), and either K_2CO_3 (138 mg, 1 mmol, 2 equiv.) or Cs_2CO_3 (329 mg, 1 mmol, 2 equiv.). The two different sizes of milling balls were chosen, since it was observed that two 7 mm milling balls got stuck relatively quickly in the milling process. *The choice of base will be specified within the respective compound characterization section.* The closed vessel was mounted into the holding station of the mixer mill and milling was conducted at 30 Hz for 90 minutes. After the reaction, the crude material was washed out with dichloromethane (DCM), filtered over a short plug of silica in a cotton-stuffed Pasteur pipette, eluted with small amounts of DCM (2-4 mL) and the solvent was subsequently removed under reduced pressure. If not stated otherwise, no further purification step was needed.

General Procedure D – for the preparation of the allylating agent scope:

A Teflon milling jar (7 mL) equipped with two ZrO_2 milling balls (one with 7 mm diameter, one with 10 mm diameter) was charged with the respective nucleophile (0.5 mmol, 1 equiv.), the respective allyl ammonium chloride (0.7 mmol, 1.4 equiv.), $Pd[(allyl)Cl]_2$ (2.7 mg, 1.5 mol%), *rac*-BINAP (9.5 mg, 3 mol%), and either K_2CO_3 (138 mg, 1 mmol, 2 equiv.) or Cs_2CO_3 (329 mg, 1 mmol, 2 equiv.). The two different sizes of milling balls were chosen, since it was observed that two 7 mm milling balls got stuck relatively quickly in the milling process. Subsequently, water was added *via* an Eppendorf® pipette (45 µL, 5 equiv.) *The choice of base will be specified within the respective compound characterization section.* The closed vessel was mounted into the holding station of the mixer mill and milling was conducted at 30 Hz for 90 minutes. After the reaction, the crude material was washed out with dichloromethane (DCM), filtered over a short plug of silica in a cotton-stuffed Pasteur pipette, eluted with small amounts of DCM (2-4 mL) and the solvent was subsequently removed under reduced pressure. If not stated otherwise, no further purification step was needed.

Optimization of Reaction Parameters

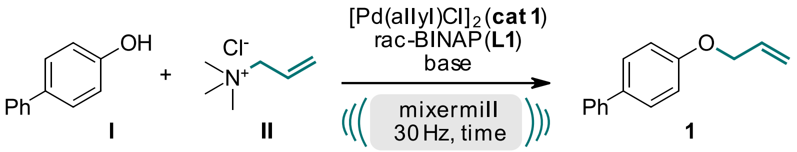

Base:

Reactions were performed following the general procedure A, using K_2CO_3 or Cs_2CO_3 as the base, allyl trimethylammonium chloride (76 mg, 0.55 mmol, 1.1 equiv.), Pd[(allyl)Cl]$_2$ (4.57 mg, 5 mol%), *rac*-BINAP (15.6 mg, 10 mol%) with a milling time of 120 minutes. Isolated yields are shown.

Table 1. Optimization of reaction parameters - base

entry	base	base equivalents	yield [%]
1	no base	-	0
2	Cs_2CO_3	0.3	42
3	Cs_2CO_3	1.1	77
4	Cs_2CO_3	2	93
5	K_2CO_3	2	90

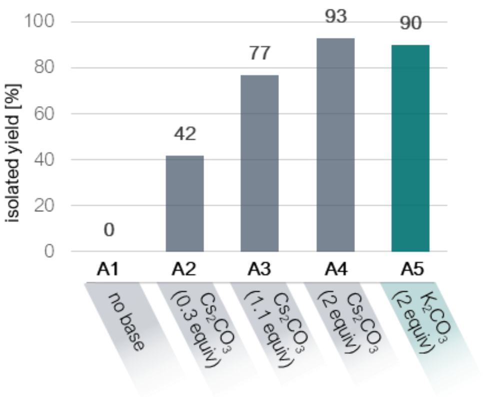

Figure 1. Optimization of reaction parameters - base

Catalyst and ligand loading:

Reactions were performed following the general procedure A, using K_2CO_3 (138 mg, 1 mmol, 2 equiv.) as the base, allyl trimethylammonium chloride (76 mg, 0.55 mmol, 1.1 equiv.), Pd[(allyl)Cl]$_2$, *rac*-BINAP with a milling time of 120 minutes. Isolated yields are shown.

Table 2. Optimization of reaction parameters – catalyst and ligand loading

entry	Pd[(allyl)Cl]$_2$ [mol%]	*rac*-BINAP [mol%]	yield [%]
1	5	10	90
2	2.5	5	94
3	1	2	94
4	0.5	1	93
5	-	-	0
6	0.5	-	5

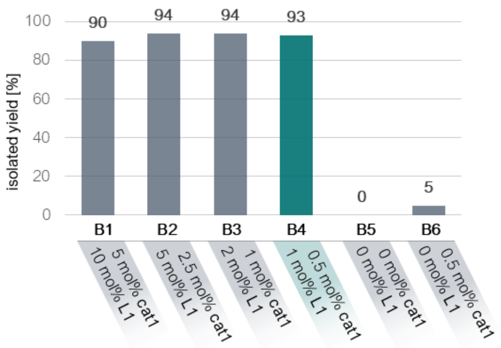

Figure 2. Optimization of reaction parameters - catalyst and ligand loading

Milling time:

Reactions were performed following the general procedure A, using K_2CO_3 (138 mg, 1 mmol, 2 equiv.) as the base, allyl trimethylammonium chloride (76 mg, 0.55 mmol, 1.1 equiv.), Pd[(allyl)Cl]$_2$ (0.91 mg, 0.5 mol%), *rac*-BINAP (3.11 mg, 1 mol%). The milling time is given in the table below. Isolated yields are shown.

Table 3. Optimization of reaction parameters – milling time

entry	milling time [min]	yield [%]
1	30	30
2	60	36
3	90	97
4	120	93

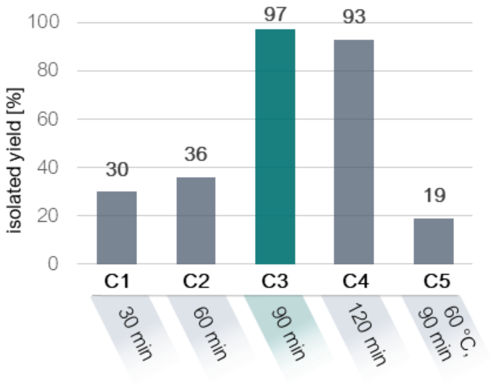

Figure 3. Optimization of reaction parameters - milling time

Chiral ligand screening:

Reactions were performed, following the general procedure D from commercially available 4-methoxyphenol with the deviation that the chiral ligand was varied (3 mol %). The enantiomers were separated using CHIRALCEL OD column eluting with 99.5:0.5 heptane:iso-propanol at 1.0 mL/min. Retention times for racemic mixture: **(R)-43** 10.87 min; **(S)-43** 13.70 min. The absolute configurations of the major isomers were assigned based on reported data in literature. [27] The detailed chromatograms and a detailed procedure for the reaction using (R)-SEGPHOS as the ligand are attached at the end of the document.

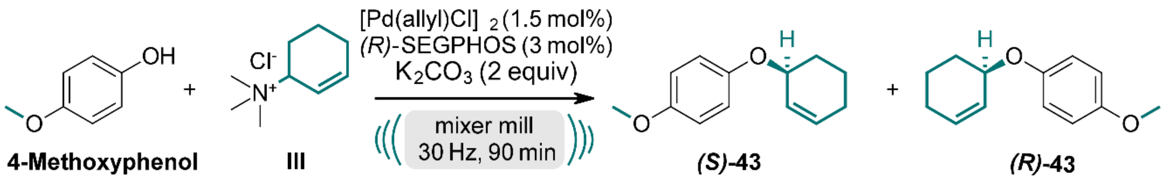

Table 4. Optimization of Reaction Conditions – chiral ligand screening

entry	ligand	CAS number ligand	***(R)*-43 rel. [%]**	***(S)*-43 rel. [%]**	*ee* [%]
1	*rac*-BINAP	98327-87-8	50	50	-
2	*(R)*-BINAP	76189-55-4	36	64	28
3	*(S)*-tol-BINAP	100165-88-6	61	39	22
4	*(S,S)*-DACH-phenyl-Trost	169689-05-8	46	54	8
5	*(R)*-SEGPHOS	244261-66-3	76	24	52

Control Experiments

Reactions in solution:

An 8 mL glass vial was charged with 4-hydroxybiphenyl (85 mg, 0.5 mmol, 1 equiv.), the allyl trimethylammonium chloride (76 mg, 0.55 mmol, 1.1 equiv.), Pd[(allyl)Cl]$_2$ (0.95 mg, 0.5 mol%), *rac*-BINAP (3.11 mg, 1 mol%), and either K_2CO_3 (138 mg, 1 mmol, 2 equiv.) and 1 mL toluene (p.A. grade) was added. The vial was closed and stirred at room temperature (control 1) or 60 °C (control 2) for 90 minutes. After the reaction, the crude reaction mixture was filtered over a short plug of silica in a cotton-stuffed Pasteur pipette, eluted with small amounts of DCM (2-4 mL) and the solvent was subsequently removed under reduced pressure. The residue was purified *via* column chromatography (dry load on celite, 5 g silica, LP:EA 50:1). Only product and residual starting material were obtained.

Table 5. Control experiments – reactions in solution

experiment	solvent	reaction time [min]	temperature [°C]	yield [%]
control 1	toluene	90	25	4
control 2	toluene	90	60	19

Reaction using allyl bromide as the allylating agent:

A Teflon milling jar (7 mL) equipped with two ZrO_2 milling balls (one with 7 mm diameter, one with 10 mm diameter) was charged with the 4-hydroxybiphenyl (85 mg, 0.5 mmol, 1 equiv.), allyl bromide (66 mg, 0.55 mmol, 1.1 equiv.), Cs_2CO_3 (247 mg, 0.75 mmol, 1.5 equiv.), once with Pd[(allyl)Cl]$_2$ (0.95 mg, 5 mol%), *rac*-BINAP (3.11 mg, 10 mol%) (control 3), and once without any catalyst and ligand (control 4). The closed vessel was mounted into the holding station of the mixer mill and milling was conducted at 30 Hz for 0 minutes. After the reaction, the crude material was washed out with dichloromethane (DCM), filtered over a short plug of silica in a cotton-stuffed Pasteur pipette, eluted with small amounts of DCM (2-4 mL) and the solvent was subsequently removed under reduced pressure.

Table 6. Control experiments – reactions using allyl bromide as the allylating agent

experiment	Pd[(allyl)Cl]$_2$ [mol%]	*rac*-BINAP [mol%]	reaction time [min]	yield [%]
control 3	5	10	120	93
control 4	-	-	120	91

Reaction using cinnamyl chloride instead of cinnamyl trimethylammonium chloride:

A Teflon milling jar (7 mL) equipped with two ZrO_2 milling balls (one with 7 mm diameter, one with 10 mm diameter) was charged with the 4-hydroxybiphenyl (85 mg, 0.5 mmol, 1 equiv.), cinnamyl chloride (88 mg, 0.55 mmol, 1.1 equiv.), Cs_2CO_3 (247 mg, 0.75 mmol, 1.5 equiv.), once with Pd[(allyl)Cl]$_2$ (0.95 mg, 1 mol%), *rac*-BINAP (3.11 mg, 2 mol%). The closed vessel was mounted into the holding station of the mixer mill and milling was conducted at 30 Hz for 90 minutes. After the reaction, the crude material was washed out with dichloromethane (DCM), filtered over a short plug of silica in a cotton-stuffed Pasteur pipette, eluted with small amounts of DCM (2-4 mL) and the solvent was subsequently removed under reduced pressure to obtain 119 mg of a crude brown oil. Crude NMR showed the formation of multiple products and starting material.

The NMR spectra shows the crude reaction mixture after silica filtration when cinnamyl chloride is used as allylating agent (top spectrum, blue) and when cinnamyl trimethylammonium chloride is used as the allylating agent (bottom spectrum, red) in the reaction.

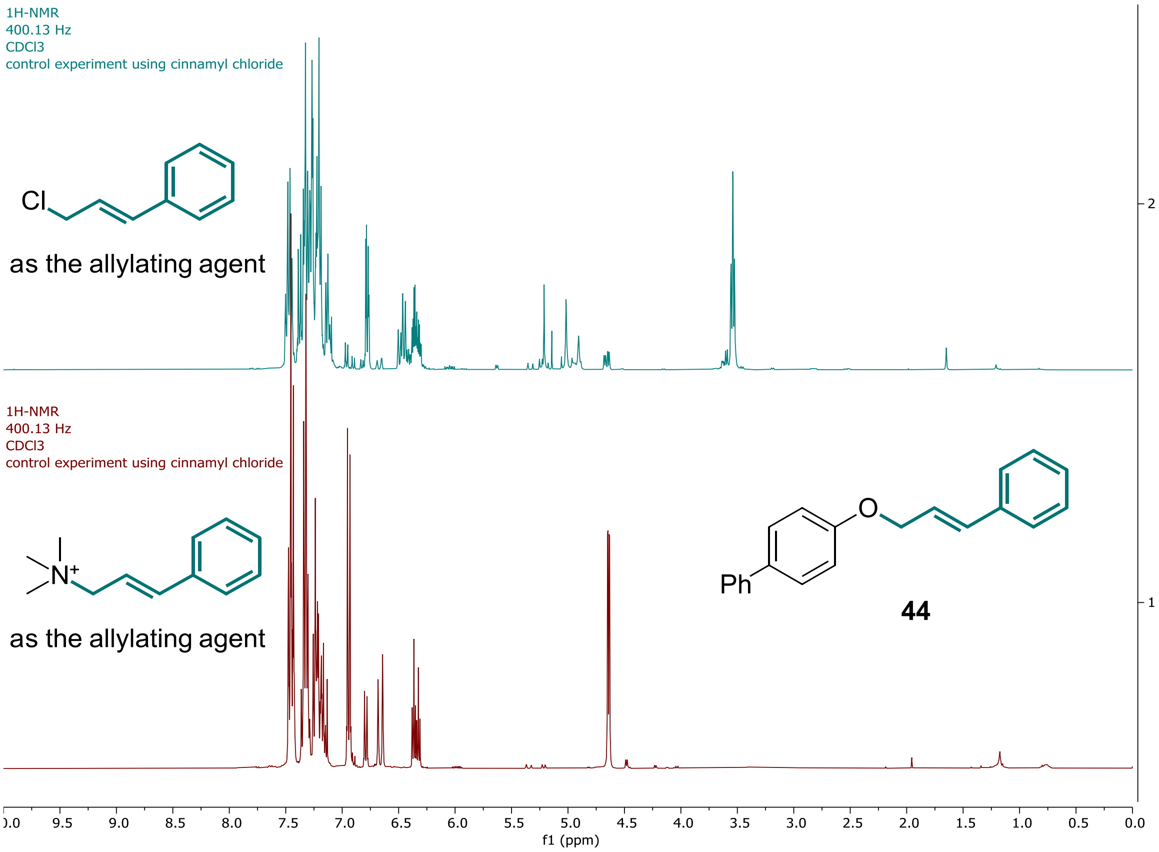

Figure 4. NMR of the crude reaction mixtures after silica filtration. Top spectrum (blue): cinnamyl chloride is used as the allylating agent; bottom spectrum (red): allyl trimethylammonium chloride is used as the allylating agent. The bottom spectrum shows full conversion and solely product formation (cf. NMR-spectrum of compound 44)

Preparation of Allylic Trimethylammonium Salts

2-Cyclohexen-1-yltrimethylammonium chloride **(III)**

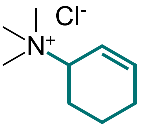

The allylic chloride intermediate was prepared according to the general procedure B1 from commercially available 2-cyclohexen-1-ol (5.17 g, 5.2 mL, 50 mmol, 1 equiv.) and conc. HCl (12.7 mL, 37 %, 3 equiv.) for 18 h. The crude product was subsequently reacted with a solution of trimethylamine in EtOH (30 mL, 2.5 equiv., 4.2 M). The title compound was obtained as a white powder (6.9 g, 79 %)

^{1}H NMR (400 MHz,) δ 6.31 – 6.21 (m, 1H), 5.81 (dt, *J* = 10.5, 2.1 Hz, 1H), 4.48 (ddt, *J* = 8.8, 5.6, 2.8 Hz, 1H), 3.39 (d, *J* = 1.5 Hz, 9H), 2.33 – 2.22 (m, 1H), 2.05 (dt, *J* = 6.2, 3.4 Hz, 2H), 1.97 – 1.86 (m, 1H), 1.79 – 1.57 (m, 2H).

^{13}C NMR (101 MHz, $CDCl_3$) δ 138.7, 118.7, 70.7, 50.7, 24.3, 23.4, 20.6.

HRMS (ESI): m/z [M]$^+$ calcd. for $C_9H_{18}N$: 140.1434; found: 140.1437.

(3-Phenyl-2-propenyl)trimethylammonium chloride **(IV)**

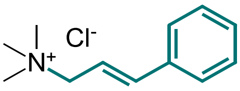

Prepared according to procedure B2 from commercially available cinnamyl chloride (3.2 g, 2.9 mL, 20 mmol, 1 equiv.) with a solution of trimethylamine in EtOH (4.2 mL, 2.5 equiv., 4.2 M). The title compound was obtained as a white powder (41.5 g, 99 %)

^{1}H NMR (400 MHz, $CDCl_3$) δ 7.37 (dq, *J* = 7.4, 2.5 Hz, 2H), 7.31 – 7.20 (m, 3H), 7.01 (d, *J* = 15.6 Hz, 1H), 6.23 (dt, *J* = 15.5, 7.7 Hz, 1H), 4.54 (d, *J* = 7.7 Hz, 2H), 3.39 (s, 9H).

^{13}C NMR (101 MHz, $CDCl_3$) δ 144.0, 134.6, 129.5, 128.9, 127.3, 114.4, 67.8, 52.7.

HRMS (ESI): m/z [M]$^+$ calcd. for $C_{12}H_{18}N$: 176.1434; found:176.1435.

(6,6-Dimethyl-2-hepten-4-ynyl)trimethylammonium chloride **(V)**

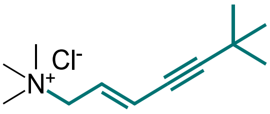

Prepared according to procedure B2 from commercially available 1-chloro-6,6-dimethyl-2-hepten-4-yne (1.12 g, 1.24 mL, 7 mmol, 1 equiv.) with a solution of trimethylamine in EtOH (12 mL, 2.5 equiv., 4.2 M). The title compound was obtained as a white powder (4.04 g, 95 %)

^{1}H NMR (400 MHz, $CDCl_3$) δ 6.25 (dt, *J* = 15.4, 1.0 Hz, 1H), 5.97 (dt, *J* = 15.5, 7.8 Hz, 1H), 4.45 (dd, *J* = 7.7, 1.0 Hz, 2H), 3.40 (s, 9H), 1.23 (s, 9H).

^{13}C NMR (101 MHz, $CDCl_3$) δ 125.7, 125.3, 104.6, 75.9, 67.3, 52.8, 30.7, 28.2.

HRMS (ESI): m/z [M]$^+$ calcd. for $C_{12}H_{22}N$: 180.1747; found: 180.1749.

2,4-Pentadienyltrimethylammonium chloride **(VI)**

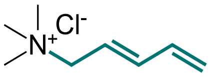

The allylic chloride intermediate was prepared according to the general procedure B1 from commercially available 1,4-pentadien-3-ol (841, 0.97 mL, 10 mmol, 1 equiv.) and conc. HCl (2.6 mL, 37 %, 3 equiv.) for 2 h. The crude product was subsequently reacted with a solution of trimethylamine in EtOH (6 mL, 2.5 equiv., 4.2 M). The title compound was obtained as an off-white powder (840 mg, 52 %)

^{1}H NMR (400 MHz, $CDCl_3$) δ 6.63 (dd, *J* = 15.1, 10.6 Hz, 1H), 6.32 (dt, *J* = 17.0, 10.3 Hz, 1H), 5.71 (dt, *J* = 15.3, 7.8 Hz, 1H), 5.38 (d, *J* = 16.9 Hz, 1H), 5.29 (d, *J* = 10.1 Hz, 1H), 4.40 (d, *J* = 7.8 Hz, 2H), 3.36 (s, 9H).

^{13}C NMR (101 MHz, $CDCl_3$) δ 144.6, 134.7, 123.1, 118.1, 67.6, 52.7.

HRMS (ESI): m/z $[M]^+$ calcd. for $C_8H_{16}N$: 126.1277; found: 126.1279.

(1-Methyl-2-butenyl)trimethylammonium chloride **(VII)**

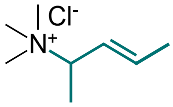

The allylic chloride intermediate was prepared according to the general procedure B1 from commercially available 3-penten-2-ol (1.7 g, 2 mL, 19 mmol, 1 equiv.) and conc. HCl (4.8 mL, 37 %, 3 equiv.) for 18 h. The crude product was subsequently reacted with a solution of trimethylamine in EtOH (11 mL, 2.5 equiv., 4.2 M). The title compound was obtained as a white powder (1.42 g, 46 %).

^{1}H NMR (400 MHz, $CDCl_3$) δ 6.20 (dq, *J* = 15.1, 6.6 Hz, 1H), 5.38 (ddq, *J* = 15.0, 9.3, 1.7 Hz, 1H), 4.55 (dq, *J* = 9.3, 6.7 Hz, 1H), 3.33 – 3.27 (m, 9H), 1.76 (dd, *J* = 6.6, 1.7 Hz, 3H), 1.46 (d, *J* = 6.7 Hz, 3H).

^{13}C NMR (101 MHz, $CDCl_3$) δ 139.2 123.2, 72.3, 50.7, 44.9, 18.3, 15.5.

HRMS (ESI): m/z $[M]^+$ calcd. for $C_8H_{18}N$: 128.1434; found: 128.1435.

Nucleophile Scope

O-Nucleophiles

4-Allyloxy-biphenyl **(1)** [CAS: 20281-44-1]

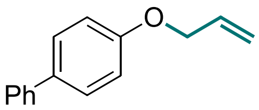

Prepared, following the general procedure C from commercially available starting material. K_2CO_3 was used as the base. Without further purification, the title compound was obtained as a white solid (120 mg, 97 %).

Analytical data is in accordance with literature [28]

^{1}H NMR (400 MHz, Chloroform-*d*) δ 7.67 – 7.55 (m, 4H), 7.48 (dd, *J* = 8.4, 6.9 Hz, 2H), 7.42 – 7.33 (m, 1H), 7.10 – 7.02 (m, 2H), 6.22 – 6.08 (m, 1H), 5.51 (dq, *J* = 17.3, 1.6 Hz, 1H), 5.38 (dq, *J* = 10.5, 1.4 Hz, 1H), 4.63 (dt, *J* = 5.3, 1.6 Hz, 2H).

^{13}C NMR (101 MHz, $CDCl_3$) δ 158.3, 140.9, 134.0, 133.4, 128.8, 128.2, 126.81, 126.77, 115.1, 68.9.

HRMS (ESI): m/z $[M+H]^+$ calcd. for $C_{15}H_{15}O$: 211.1118; found: 211.1115.

Scale up synthesis: The synthesis of 1 was additionally performed on a 5.5 mmol scale in two separate Teflon milling vessels (25 mL) each equipped with two ZrO_2 milling balls (12.7 mm). Each vessel was charged with 4-hydroxybiphenyl (468 mg, 2.75 mmol, 1 equiv.), allyl trimethylammonium chloride (419 mg, 3.03 mmol, 1.1 equiv.), K_2CO_3 (760 mg, 5.5 mmol, 2 equiv.), $Pd[(allyl)Cl]_2$ (5.03 mg, 0.013 mmol, 0.5 mol%), and *rac*-BINAP (17.7, 0.03 mmol, 1 mol%). The closed vessel was mounted into the holding station of the mixer mill and milling was conducted at 30 Hz for 90 minutes. After the reaction, the reaction mixtures of both milling vessels were combined and the crude material was washed out with dichloromethane (DCM), filtered over a short plug of silica in cotton-stuffed Pasteur pipette, eluted with DCM (30 mL) and the solvent was subsequently removed under reduced pressure. Without any further purification, the title compound was obtained as white solid in quantitative yields (1.15 g, 99 %). Analytical data was in accordance with previous measurements.

Allyl 4-(tert-butyl)phenylether **(2)** [CAS: 24806-16-4]

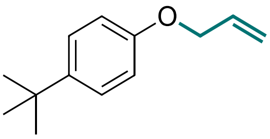

Prepared, following the general procedure C from commercially available starting material. K_2CO_3 was used as the base. Without further purification, the title compound was obtained as a colorless oil (94 mg, 99 %)
Analytical data is in accordance with literature[29]

^{1}H NMR (400 MHz, Chloroform-*d*) δ 7.36 – 7.28 (m, 2H), 6.92 – 6.84 (m, 2H), 6.15 – 6.01 (m, 1H), 5.43 (dq, *J* = 17.3, 1.6 Hz, 1H), 5.29 (dq, *J* = 10.5, 1.4 Hz, 1H), 4.54 (dt, *J* = 5.3, 1.6 Hz, 2H), 1.32 (s, 9H).

^{13}C NMR (101 MHz, $CDCl_3$) δ 156.5, 143.6, 133.7, 126.3, 117.6, 114.3, 69.0, 34.2, 31.7.

Allyl phenyl ether **(3)** [CAS: 1746-13-0]

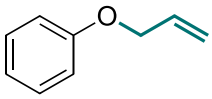

Prepared, following the general procedure C from commercially available starting material. K_2CO_3 was used as the base. Without further purification, the title compound was obtained as a colorless oil (63 mg, 94 %).
Analytical data is in accordance with literature[30]

^{1}H NMR (400 MHz, Chloroform-*d*) δ 7.35 – 7.27 (m, 2H), 7.03 – 6.91 (m, 3H), 6.16 – 6.02 (m, 1H), 5.44 (dq, *J* = 17.2, 1.6 Hz, 1H), 5.31 (dq, *J* = 10.5, 1.5 Hz, 1H), 4.56 (dt, *J* = 5.3, 1.6 Hz, 2H).

^{13}C NMR (101 MHz, $CDCl_3$) δ 158.7, 133.5, 129.6, 121.0, 117.7, 114.9, 68.8.

1-(Allyloxy)-4-fluorobenzene **(4)** [CAS: 13990-72-2]

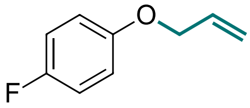

Prepared, following the general procedure C from commercially available starting material. K_2CO_3 was used as the base. Without further purification, the title compound was obtained as yellow oil (67 mg, 88 %)
Analytical data is in accordance to literature[31]

^{1}H NMR (400 MHz, Chloroform-*d*) δ 7.03 – 6.92 (m, 2H), 6.90 – 6.81 (m, 2H), 6.12 – 5.98 (m, 1H), 5.41 (dq, *J* = 17.3, 1.6 Hz, 1H), 5.29 (dq, *J* = 10.5, 1.4 Hz, 1H), 4.50 (dt, *J* = 5.3, 1.6 Hz, 2H).

^{13}C NMR (101 MHz, Chloroform-*d*) δ 157.4 (d, *J* = 238.3 Hz), 154.8 (d, *J* = 2.1 Hz), 133.3, 117.9, 116.0 (d, *J* = 9.3 Hz), 115.8 (d, *J* = 5.8 Hz), 69.6.

^{19}F NMR (376 MHz, Chloroform-*d*) δ -123.94.

5-(allyloxy)-2-chloro-1,3-dimethylbenzene **(5)** [CAS: 93589-80-1]

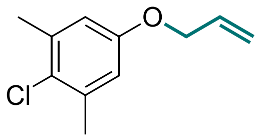

Prepared, following the general procedure C from commercially available starting material. K_2CO_3 was used as the base. Without further purification, the title compound was obtained as a colorless oil (98 mg, 99 %).
Analytical data is in accordance with literature[32]

^{1}H NMR (400 MHz, Chloroform-*d*) δ 6.67 (s, 2H), 6.12 – 5.98 (m, 1H), 5.41 (dq, *J* = 17.3, 1.7 Hz, 1H), 5.29 (dq, *J* = 10.5, 1.5 Hz, 1H), 4.50 (dt, *J* = 5.3, 1.6 Hz, 2H), 2.36 (s, 6H).

^{13}C NMR (101 MHz, $CDCl_3$) δ 156.5, 137.2, 133.3, 126.5, 117.7, 114.9, 69.0, 21.1.

1-(Allyloxy)-2-bromobenzene **(6)** [CAS: 60333-75-7]

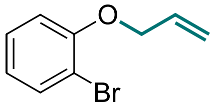

Prepared, following the general procedure C from commercially available starting material. K_2CO_3 was used as the base. Without further purification, the title compound was obtained as a slightly yellow oil (99 mg, 92 %)
Analytical data is in accordance with literature[33]

^{1}H NMR (400 MHz, Chloroform-*d*) δ 7.55 (dd, *J* = 7.9, 1.7 Hz, 1H), 7.29 – 7.20 (m, 1H), 6.90 (dd, *J* = 8.2, 1.4 Hz, 1H), 6.84 (td, *J* = 7.6, 1.4 Hz, 1H), 6.15 – 6.01 (m, 1H), 5.50 (dq, *J* = 17.3, 1.7 Hz, 1H), 5.32 (dq, *J* = 10.5, 1.5 Hz, 1H), 4.62 (dt, *J* = 5.0, 1.7 Hz, 2H).

^{13}C NMR (101 MHz, $CDCl_3$) δ 155.04, 133.5, 132.7, 128.5, 122.1, 117.8, 113.7, 112.4, 69.7.

1-Bromo-2-methoxy-4-allyloxybenzene **(7)** [CAS: 200336-42-1]

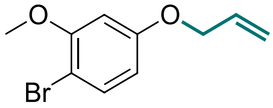

Prepared, following the general procedure C from commercially available starting material. K_2CO_3 was used as the base. Without further purification, the title compound was obtained as a colorless oil (120 mg, 99 %)

^{1}H NMR (400 MHz, Chloroform-*d*) δ 7.39 (d, *J* = 8.7 Hz, 1H), 6.52 (d, *J* = 2.7 Hz, 1H), 6.40 (dd, *J* = 8.7, 2.7 Hz, 1H), 6.11 – 5.97 (m, 1H), 5.41 (dq, *J* = 17.3, 1.6 Hz, 1H), 5.30 (dq, *J* = 10.5, 1.4 Hz, 1H), 4.51 (dt, *J* = 5.3, 1.6 Hz, 2H), 3.86 (s, 3H).

^{13}C NMR (101 MHz, $CDCl_3$) δ 159.3, 156.7, 133.2, 133.0, 118.1, 106.9, 102.7, 100.8, 69.2, 56.2.

HRMS (ESI): m/z $[M+H]^+$ calcd. for $C_{10}H_{12}BrO_2$: 243.0015; found: 243,0015.

1-(Allyloxy)-4-methoxybenzene **(8)** [CAS: 13391-35-0]

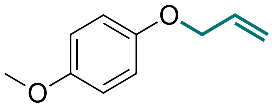

Prepared, following the general procedure C from commercially available starting material. K_2CO_3 was used as the base. Without further purification, the title compound was obtained as a colorless oil (81 mg, 99 %).
Analytical data is in accordance with literature[32]

^{1}H NMR (400 MHz, Chloroform-*d*) δ 6.91 – 6.79 (m, 4H), 6.13 – 5.99 (m, 1H), 5.41 (dq, *J* = 17.2, 1.6 Hz, 1H), 5.28 (dq, *J* = 10.5, 1.4 Hz, 1H), 4.49 (dt, *J* = 5.3, 1.6 Hz, 2H), 3.77 (s, 3H).

^{13}C NMR (101 MHz, $CDCl_3$) δ 154.0, 152.9, 133.8, 117.6, 115.9, 114.7, 69.6, 55.8.

4-Allyl-1-(allyloxy)-2-methoxybenzene **(9)** [CAS: 4125-45-5]

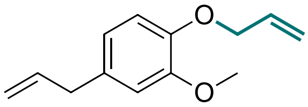

Prepared, following the general procedure C from commercially available starting material. K_2CO_3 was used as the base. Without further purification, the title compound was obtained as a colorless oil (93 mg, 91 %).

Analytical data is in accordance with literature[34]

^{1}H NMR (400 MHz, Chloroform-*d*) δ 6.82 (d, *J* = 8.0 Hz, 1H), 6.75 – 6.65 (m, 2H), 6.16 – 6.03 (m, 1H), 6.02 – 5.90 (m, 1H), 5.39 (dq, *J* = 17.3, 1.6 Hz, 1H), 5.27 (dq, *J* = 10.5, 1.4 Hz, 1H), 5.13 – 5.07 (m, 1H), 5.07 – 5.02 (m, 1H), 4.59 (dt, *J* = 5.4, 1.5 Hz, 2H), 3.87 (s, 3H), 3.34 (dt, *J* = 6.7, 1.5 Hz, 2H).

^{13}C NMR (101 MHz, $CDCl_3$) δ 149.5, 146.5, 137.8, 133.7, 133.2, 120.5, 117.9, 115.8, 113.8, 112.4, 70.2, 56.0, 39.9.

HRMS (ESI): m/z $[M+H]^+$ calcd. for $C_{13}H_{17}O_2$: 205.1223; found: 205,1224

1-(Allyloxy)-2-methoxybenzene **(10)** [CAS: 4125-43-3]

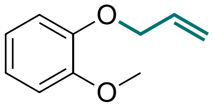

Prepared, following the general procedure C from commercially available starting material. K_2CO_3 was used as the base. Without further purification, the title compound was obtained as a colorless oil (82 mg, 99%).

Analytical data is in accordance with literature[35]

^{1}H NMR (400 MHz, Chloroform-*d*) δ 6.98 – 6.83 (m, 4H), 6.17 – 6.03 (m, 1H), 5.41 (dq, *J* = 17.3, 1.6 Hz, 1H), 5.29 (dq, *J* = 10.5, 1.4 Hz, 1H), 4.62 (dt, *J* = 5.5, 1.5 Hz, 2H), 3.88 (s, 3H).

^{13}C NMR (101 MHz, $CDCl_3$) δ 149.6, 148.1, 133.5, 121.3,0 120.8, 117.9, 113.7, 111.8, 69.9, 55.9.

HRMS (ESI): m/z $[M+H]^+$ calcd. for $C_{10}H_{13}O_2$: 165.0910; found: 165.0911

O-Allyl-sesamol **(11)** [CAS: 19202-22-3]

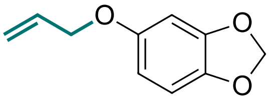

Prepared, following the general procedure C from commercially available starting material. K_2CO_3 was used as the base. Without further purification, the title compound was obtained as a slightly yellow oil (88 mg, 99 %).

Analytical data is in accordance with literature. [36]

^{1}H NMR (400 MHz, Chloroform-*d*) δ 6.70 (d, *J* = 8.5 Hz, 1H), 6.52 (d, *J* = 2.5 Hz, 1H), 6.34 (dd, *J* = 8.5, 2.5 Hz, 1H), 6.11 – 5.97 (m, 1H), 5.91 (s, 2H), 5.40 (dq, *J* = 17.3, 1.6 Hz, 1H), 5.28 (dq, *J* = 10.5, 1.4 Hz, 1H), 4.46 (dt, *J* = 5.4, 1.5 Hz, 2H).

^{13}C NMR (101 MHz, $CDCl_3$) δ 154.2, 148.3, 141.8, 133.5, 117.7, 108.0, 106.1, 101.2, 98.4, 69.9.

HRMS (ESI): m/z $[M+H]^+$ calcd. for $C_{10}H_{11}O_3$: 179.0703; found: 179.0702

1-Allyloxy-3-nitrobenzene **(12)** [CAS: 58621-55-9]

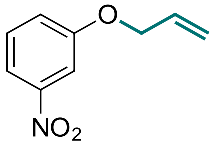

Prepared, following the general procedure C from commercially available starting material. Cs_2CO_3 was used as the base. Without further purification, the title compound was obtained as slightly yellow oil (88 mg, 98 %).

Analytical data is in accordance with literature. [37]

^{1}H NMR (400 MHz, Chloroform-*d*) δ 7.84 – 7.77 (m, 1H), 7.73 (t, *J* = 2.3 Hz, 1H), 7.42 (t, *J* = 8.2 Hz, 1H), 7.27 – 7.20 (m, 1H), 6.11 – 5.97 (m, 1H), 5.44 (dq, *J* = 17.3, 1.6 Hz, 1H), 5.34 (dq, *J* = 10.5, 1.4 Hz, 1H), 4.61 (dt, *J* = 5.3, 1.6 Hz, 2H).

^{13}C NMR (101 MHz, $CDCl_3$) δ 159.2, 149.3, 132.2, 130.0, 122.00, 118.6, 116.0, 109.2, 69.4.

HRMS (ESI): m/z [M+H]$^+$ calcd. for $C_9H_{10}NO_3$: 180.0655; found: 180.0656

2-(Allyloxy)benzaldehyde **(13)** [CAS: 28752-82-1]

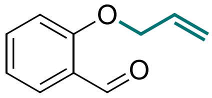

Prepared, following the general procedure C from commercially available starting material. K_2CO_3 was used as the base. Without further purification, the title compound was obtained as yellow oil (60 mg, 74 %).

Analytical data is in accordance with literature. [38]

^{1}H NMR (400 MHz, Chloroform-*d*) δ 10.53 (d, *J* = 0.8 Hz, 1H), 7.83 (dd, *J* = 7.7, 1.9 Hz, 1H), 7.56 – 7.48 (m, 1H), 7.06 – 6.92 (m, 2H), 6.14 – 6.00 (m, 1H), 5.45 (dq, *J* = 17.3, 1.6 Hz, 1H), 5.33 (dq, *J* = 10.6, 1.5 Hz, 1H), 4.65 (dt, *J* = 5.2, 1.6 Hz, 2H).

^{13}C NMR (101 MHz, $CDCl_3$) δ 189.6, 161.0, 135.9, 132.5, 128.5, 121.0, 118.2, 113.0, 69.3.

HRMS (ESI): m/z [M+H]$^+$ calcd. for $C_{10}H_{11}O_2$: 163.0754; found 163.0754

Allyl furfuryl ether **(14)** [CAS: 113505-00-3]

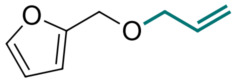

Prepared, following the general procedure C from commercially available starting material. Cs_2CO_3 was used as the base. Without further purification, the title compound was obtained as yellow oil (51 mg, 74 %).

Analytical data is in accordance with literature. [39]

^{1}H NMR (400 MHz, Chloroform-*d*) δ 7.41 (dd, *J* = 1.8, 0.9 Hz, 1H), 6.37 – 6.28 (m, 2H), 5.99 – 5.85 (m, 1H), 5.35 – 5.25 (m, 1H), 5.21 (dq, *J* = 10.4, 1.3 Hz, 1H), 4.46 (s, 2H), 4.02 (dt, *J* = 5.7, 1.4 Hz, 2H).

^{13}C NMR (101 MHz, $CDCl_3$) δ 151.9, 142.9, 134.5, 117.6, 110.4, 109.4, 71.1, 63.9.

HRMS (ESI): m/z [M+H]$^+$ calcd. for $C_8H_{11}O_2$: 139.0754; found: 139.0751

Benzyl allyl ether **(15)** [CAS: 14593-43-2]

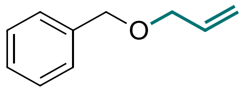

Prepared, following the general procedure C from commercially available starting material. Cs_2CO_3 was used as the base. Without further purification, the title compound was obtained as a colorless oil (56 mg, 76 %).

Analytical data is in accordance with literature. [40]

^{1}H NMR (400 MHz, Chloroform-*d*) δ 7.42 – 7.27 (m, 5H), 6.06 – 5.92 (m, 1H), 5.34 (dq, *J* = 17.2, 1.7 Hz, 1H), 5.23 (dq, *J* = 10.4, 1.4 Hz, 1H), 4.55 (s, 2H), 4.06 (dt, *J* = 5.6, 1.5 Hz, 2H).

^{13}C NMR (101 MHz, $CDCl_3$) δ 138.4, 134.9, 128.5, 127.8, 127.7, 117.2, 72.2, 71.3.

2-(2-propenyloxy)ethylbenzene **(16)** [CAS: 14289-65-7]

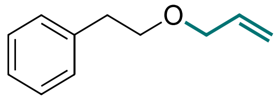

Prepared, following the general procedure C from commercially available starting material. Cs_2CO_3 was used as the base. Without further purification, the title compound was obtained as a colorless oil (68 mg, 84 %).

Analytical data is in accordance with literature. [41]

^{1}H NMR (400 MHz, Chloroform-*d*) δ 7.33 – 7.24 (m, 2H), 7.24 – 7.14 (m, 3H), 5.98 – 5.83 (m, 1H), 5.25 (dq, *J* = 17.2, 1.7 Hz, 1H), 5.16 (dq, *J* = 10.4, 1.4 Hz, 1H), 3.99 (dt, *J* = 5.6, 1.5 Hz, 2H), 3.65 (t, *J* = 7.3 Hz, 2H), 2.91 (t, *J* = 7.3 Hz, 2H).

^{13}C NMR (101 MHz, $CDCl_3$) δ 139.1, 135.0, 129.0, 128.5, 126.3, 116.9, 72.0, 71.4, 36.5.

2-[4-(Allyloxy)-3-methoxyphenyl]-1,3-dioxolane (**17**)

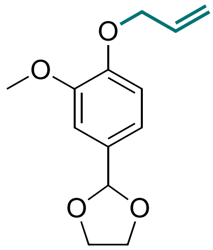

Prepared, following the general procedure C from commercially available starting material. Cs_2CO_3 was used as the base. Without further purification, the title compound was obtained as a colorless oil (107 mg, 91 %).

^{1}H NMR (400 MHz, $CDCl_3$) δ 7.05 – 6.96 (m, 2H), 6.86 (d, *J* = 8.2 Hz, 1H), 6.14 – 6.00 (m, 1H), 5.74 (s, 1H), 5.39 (dq, *J* = 17.3, 1.6 Hz, 1H), 5.27 (dq, *J* = 10.5, 1.4 Hz, 1H), 4.61 (dt, *J* = 5.4, 1.5 Hz, 2H), 4.17 – 4.07 (m, 2H), 4.07 – 3.96 (m, 2H), 3.89 (s, 3H).

^{13}C NMR (101 MHz, $CDCl_3$) δ 149.6, 148.9, 133.3, 130.7, 119.3, 118.1, 113.0, 109.6, 103.8, 70.0, 65.3, 56.0.

HRMS (ESI): m/z $[M+H]^+$ calcd. for C13H17O4: 237.1122; found: 237.1125

2-(4-(allyloxy)phenyl)pyridine **(18)** [CAS: 1623748-79-7]

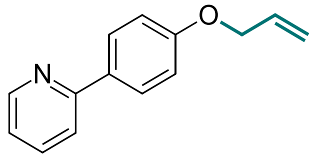

Prepared, following the general procedure C from commercially available starting material. Cs_2CO_3 was used as the base. The crude product was purified *via* column chromatography (6 g, silica, pentane:EtOAc 10:1 – 9:1) to obtain the title compound as a white solid (64 mg, 61 %).

Analytica data is in accordance with literature. [42]

^{1}H NMR (400 MHz, Chloroform-*d*) δ 8.68 – 8.62 (m, 1H), 7.98 – 7.90 (m, 2H), 7.74 – 7.67 (m, 1H), 7.66 (dt, *J* = 8.1, 1.3 Hz, 1H), 7.20 – 7.12 (m, 1H), 7.05 – 6.97 (m, 2H), 6.15 – 6.01 (m, 1H), 5.44 (dq, *J* = 17.3, 1.6 Hz, 1H), 5.30 (dq, *J* = 10.5, 1.4 Hz, 1H), 4.59 (dt, *J* = 5.3, 1.6 Hz, 2H).

^{13}C NMR (101 MHz, $CDCl_3$) δ 159.6, 157.2, 149.7, 136.8, 133.2, 132.3, 128.3, 121.5, 119.9, 117.9, 115.0, 69.0.

HRMS (ESI): m/z $[M+H]^+$ calcd. for $C_{14}H_{14}NO$: 212.1070; found: 212.1077

(E)-1-(Allyloxy)-3,7-dimethylocta-2,6-diene **(19)** [CAS: 35534-61-3]

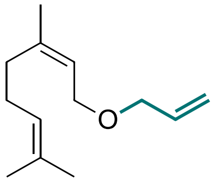

Prepared, following the general procedure C from commercially available starting material. K_2CO_3 was used as the base. Without further purification, the title compound was obtained as colorless oil (76 mg, 78 %).

Analytical data is in accordance with literature. [43]

^{1}H NMR (400 MHz, Chloroform-*d*) δ 6.00 – 5.85 (m, 1H), 5.36 (tq, *J* = 6.8, 1.3 Hz, 1H), 5.27 (dq, *J* = 17.2, 1.7 Hz, 1H), 5.17 (dq, *J* = 10.3, 1.3 Hz, 1H), 5.12 – 5.06 (m, 1H), 3.99 (d, *J* = 6.8 Hz, 2H), 3.96 (dt, *J* = 5.7, 1.4 Hz, 2H), 2.15 – 2.07 (m, 2H), 2.06 – 2.00 (m, 2H), 1.67 (s, 3H), 1.66 (s, 3H), 1.59 (s, 3H).

^{13}C NMR (101 MHz, $CDCl_3$) δ 140.3, 135.2, 131.8, 124.1, 120.9, 117.0, 71.1, 66.7, 39.7, 26.5, 25.8, 17.8, 16.6

HRMS (ESI): m/z $[M+H]^+$ calcd. for $C_{13}H_{23}O$: 195.1743; found: 195.1742

O-Allyl citronellol **(20)** [CAS: 139694-24-9]

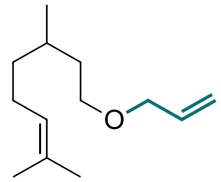

Prepared, following the general procedure C from commercially available starting material. Cs_2CO_3 was used as the base. Without further purification, the title compound was obtained as a colorless oil (94 mg, 96 %).

Analytical data is in accordance with literature. [44]

^{1}H NMR (400 MHz, Chloroform-*d*) δ 5.99 – 5.81 (m, 1H), 5.25 (dq, *J* = 17.2, 1.8 Hz, 1H), 5.15 (dq, *J* = 10.4, 1.4 Hz, 1H), 5.13 – 5.04 (m, 1H), 3.95 (dt, *J* = 5.6, 1.5 Hz, 2H), 3.51 – 3.37 (m, 2H), 2.06 – 1.88 (m, 2H), 1.71 – 1.50 (m, 8H), 1.46 – 1.27 (m, 2H), 1.21 – 1.09 (m, 1H), 0.89 (d, *J* = 6.6 Hz, 3H).

^{13}C NMR (101 MHz, $CDCl_3$) δ 135.2, 131.2, 124.9, 116.7, 71.9, 68.8, 37.4, 36.8, 29.7, 25.8, 25.6, 19.7, 17.7.

3-Allyloxy estrone **(21)** [CAS: 1624-67-5]

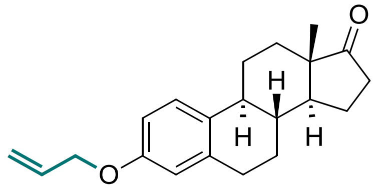

Prepared, following the general procedure C from commercially available starting material. K_2CO_3 was used as the base. Without further purification, the title compound was obtained as yellow oil (155 mg, 99 %).

Analytical data is in accordance with literature. [45]

^{1}H NMR (400 MHz, Chloroform-*d*) δ 7.20 (dd, *J* = 8.7, 1.1 Hz, 1H), 6.73 (dd, *J* = 8.6, 2.8 Hz, 1H), 6.69 – 6.62 (m, 1H), 6.12 – 5.98 (m, 1H), 5.41 (dq, *J* = 17.2, 1.6 Hz, 1H), 5.27 (dq, *J* = 10.5, 1.4 Hz, 1H), 4.51 (dt, *J* = 5.3, 1.6 Hz, 2H), 2.93 – 2.82 (m, 2H), 2.56 – 2.45 (m, 1H), 2.44 – 2.32 (m, 1H), 2.32 – 1.89 (m, 6H), 1.69 – 1.36 (m, 7H), 0.91 (s, 3H).

^{13}C NMR (101 MHz, $CDCl_3$) δ 221.1, 156.8, 137.9, 133.7, 132.3, 126.4, 117.6, 114.9, 112.5, 68.9, 50.6, 48.2, 44.1, 38.5, 36.0, 31.7, 29.8, 26.7, 26.0, 21.7, 14.0.

HRMS (ESI): m/z $[M+H]^+$ calcd. for $C_{21}H_{27}O_2$: 311.2006; found: 311.2010

N-Nucleophiles

N-Allyl-4-fluoroaniline **(22)** [CAS: 83164-79-8]

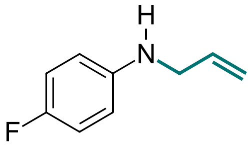

Prepared following the general procedure C with the deviation that no base was used. The crude product was purified *via* column chromatography (dry load, pentane:Et_2O 95:5). The title compound was obtained as yellow oil (47 mg, 62 %) along with small amounts of the bis-allylated compound **24** (10 mg, 11 %).

Analytical data is in accordance with literature. [46]

^{1}H NMR (400 MHz, Chloroform-*d*) δ 6.94 – 6.83 (m, 2H), 6.61 – 6.51 (m, 2H), 6.02 – 5.88 (m, 1H), 5.28 (dq, *J* = 17.2, 1.7 Hz, 1H), 5.17 (dq, *J* = 10.3, 1.5 Hz, 1H), 3.74 (dt, *J* = 5.4, 1.7 Hz, 2H), 3.71 – 3.55 (m, 1H).

^{13}C NMR (101 MHz, $CDCl_3$) δ 156.0 (d, *J* = 234.9 Hz), 144.5, 135.5, 116.5, 115.8 (d, *J* = 22.3 Hz), 113.9 (d, *J* = 7.4 Hz), 47.3.

HRMS (ESI): m/z $[M+H]^+$ calcd. for $C_9H_{11}FN$: 152.0870; found: 152.0867

N-Allyl-4-fluoro-*N*-methylaniline **(23)** [CAS: 1687753-93-0]

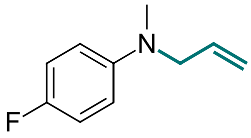

Prepared, following the general procedure C from commercially available starting material. Cs_2CO_3 was used as the base. Without further purification, the title compound was obtained as a slightly yellow oil (76 mg, 92 %)

Analytical data is in accordance with literature.[47]

^{1}H NMR (400 MHz, Chloroform-*d*) δ 7.00 – 6.89 (m, 2H), 6.72 – 6.63 (m, 2H), 5.91 – 5.77 (m, 1H), 5.23 – 5.13 (m, 2H), 3.91 – 3.85 (m, 2H), 2.91 (s, 3H).

^{13}C NMR (101 MHz, $CDCl_3$) δ 155.6 (d, *J* = 235.0 Hz), 146.4, 133.9, 116.6, 115.5 (d, *J* = 22.0 Hz), 113.9 (d, *J* = 7.3 Hz), 56.2, 38.7

^{19}F NMR (376 MHz, Chloroform-*d*) δ -129.26.

N,N-diallyl-4-fluoroaniline **(24)** [83164-94-7]

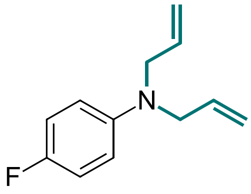

Prepared, following the general procedure C from commercially available starting material with the deviation that 2.5 equivalents of allyl trimethylammonium chloride were used and 3 equivalents of Cs_2CO_3 as the base. The crude product was purified *via* column chromatography (5 g silica, pentane:Et_2O, 100:1) to obtain the title compound as slightly yellow oil (75 mg, 78 %).

Analytical data is in accordance with literature. [48]

^{1}H NMR (400 MHz, Chloroform-d) δ 6.95 – 6.84 (m, 2H), 6.68 – 6.58 (m, 2H), 5.99 – 5.65 (m, 2H), 5.22 – 5.12 (m, 4H), 3.88 (dt, J = 4.9, 1.7 Hz, 4H).

^{13}C NMR (101 MHz, $CDCl_3$) δ 155.4 (d, *J* = 234.8 Hz), 145.5, 134.2, 116.3, 115.5 (d, *J* = 22.0 Hz), 113.7 (d, *J* = 7.2 Hz), 53.5.

HRMS (ESI): m/z $[M+H]^+$ calcd. for $C_{12}H_{15}FN$: 192.1183; found: 192.1183.

1-Allylbenzimidazole **(25)** [CAS: 19018-22-5]

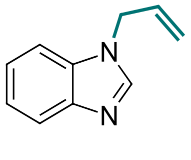

Prepared, following the general procedure C from commercially available starting material. Cs_2CO_3 was used as the base. Without further purification, the title compound was obtained as a colorless oil (79 mg, 99 %).

Analytical data is in accordance with literature.[49]

^{1}H NMR (400 MHz, Chloroform-*d*) δ 7.78 (d, *J* = 2.1 Hz, 1H), 7.76 – 7.68 (m, 1H), 7.32 – 7.13 (m, 3H), 5.97 – 5.82 (m, 1H), 5.23 – 5.15 (m, 1H), 5.13 – 5.03 (m, 1H), 4.69 – 4.62 (m, 2H).

^{13}C NMR (101 MHz, $CDCl_3$) δ 144.0, 143.0, 133.9, 132.0, 123.0, 122.2, 120.4, 118.6, 110.0, 47.4.

HRMS (ESI): m/z $[M+H]^+$ calcd. for $C_{10}H_{11}N_2$: 159.0917; found: 159.0917

1-Allylbenzotrialzole **(26)** [CAS: 52298-91-6]

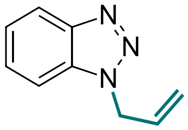

Prepared, following the general procedure C from commercially available starting material. K_2CO_3 was used as the base The crude product was purified *via* column chromatography (6 g silica, LP:EA 10:1 – 2:1) to obtain the title compound (N^1-allylbenzotriazole) (56 mg, 70 %) and the N^2-allylbenzotriazole (12 mg, 15 %) as a colorless oil. The overall yield of the allylated benzotriazole was 68 mg (85 %) with a ratio of N^1/N^2 4.7:1.

Analytical data was in accordance with literature. [20]

^{1}H NMR (400 MHz, Chloroform-*d*) δ 8.06 (dt, *J* = 8.4, 1.0 Hz, 1H), 7.51 (dt, *J* = 8.3, 1.1 Hz, 1H), 7.48 – 7.42 (m, 1H), 7.40 – 7.31 (m, 1H), 6.13 – 5.99 (m, 1H), 5.32 (dt, *J* = 10.2, 1.2 Hz, 1H), 5.30 – 5.20 (m, 3H).

^{13}C NMR (101 MHz, $CDCl_3$) δ 146.3, 133.0, 131.3, 127.4, 124.0, 120.2, 119.4, 109.8, 51.0.

HRMS (ESI): m/z $[M+H]^+$ calcd. for $C_9H_{10}N_3$: 160.0869; found: 160.0872

1-Allyl-4-(2-methoxy-phenyl)-piperazine **(27)** [CAS: 6322-40-3]

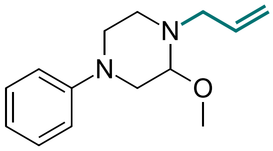

Prepared, following the general procedure C from commercially available starting material. K_2CO_3 was used as the base. Without further purification, the title compound was obtained as yellow oil (104 mg, 90 %)

^{1}H NMR (400 MHz, Chloroform-*d*) δ 7.05 – 6.88 (m, 3H), 6.85 (dd, *J* = 7.9, 1.5 Hz, 1H), 5.98 – 5.84 (m, 1H), 5.22 (dq, *J* = 17.2, 1.6 Hz, 1H), 5.19 – 5.13 (m, 1H), 3.85 (s, 3H), 3.19 – 2.98 (m, 6H), 2.66 (t, *J* = 4.7 Hz, 4H).

^{13}C NMR (101 MHz, $CDCl_3$) δ 152.4, 141.5, 135.2, 122.9, 121.0, 118.3, 118.1, 111.3, 62.0, 55.4, 53.4, 50.7.

HRMS (ESI): m/z $[M+H]^+$ calcd. for $C_{14}H_{21}N_2O$: 233.1648; found: 233.1651

N-Allyl-N-phenylhydrazine **(28)** [CAS: 31928-39-9]

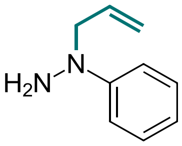

Prepared, following the general procedure C from commercially available starting material with the deviation that 1 equivalent allyl trimethylammonium chloride was used, 1 mol% of $Pd[(allyl)Cl]_2$, and 2 mol% *rac*-BINAP. Cs_2CO_3 was used as the base. Without further purification, the title compound was obtained as brown oil (57 mg, 77 %).

Analytical data is in accordance with literature. [50]

^{1}H NMR (400 MHz, Chloroform-d) δ 7.51 – 7.42 (m, 2H), 7.24 (s, 2H), 7.01 (t, J = 7.3 Hz, 1H), 6.16 – 6.02 (m, 1H), 5.53 – 5.43 (m, 2H), 4.23 (d, J = 5.9 Hz, 2H), 3.80 (bs, 2H).

^{13}C NMR (101 MHz, $CDCl_3$) δ 151.5, 132.8, 129.1, 118.7, 118.6, 113.8, 59.0.

HRMS (ESI): m/z $[M+H]^+$ calcd. for $C_9H_{13}N_2$: 149.1073; found: 149.1078

N-Allylphenothiazine **(29)** [CAS: 20962-92-9]

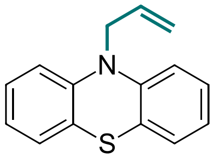

Prepared, following the general procedure C from commercially available starting material. Cs_2CO_3 was used as the base. The crude product was purified *via* column chromatography (dry load, 6 g, silica, pentane:Et_2O 50:1) to obtain the title compound as a colorless oil (72 mg, 60 %).

Analytical data is in accordance with literature.[51]

^{1}H NMR (400 MHz, Chloroform-d) δ 7.08 (dd, J = 8.3, 6.7 Hz, 4H), 6.93 – 6.80 (m, 4H), 6.07 – 5.94 (m, 1H), 5.38 – 5.24 (m, 2H), 4.48 (dt, J = 4.2, 2.0 Hz, 2H).

^{13}C NMR (101 MHz, $CDCl_3$) δ 144.6, 133.3, 127.3, 126.9, 123.2, 122.5, 117.6, 115.4, 51.4.

HRMS (ESI): m/z $[M+H]^+$ calcd. for $C_{15}H_{14}NS$: 240.0841; found: 240.0838

7-Allyl-1,3-dimethylxanthine **(30)** [CAS: 61444-26-6]

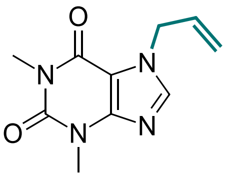

Prepared, following the general procedure C from commercially available starting material. Cs_2CO_3 was used as the base. Without further purification, the title compound was obtained as a white solid (98 mg, 89 %).

Analytical data is in accordance with literature.[52]

^{1}H NMR (400 MHz, Chloroform-d) δ 7.55 (s, 1H), 6.11 – 5.96 (m, 1H), 5.31 (dq, J = 10.2, 1.2 Hz, 1H), 5.23 (dq, J = 17.0, 1.3 Hz, 1H), 4.93 (dt, J = 5.9, 1.5 Hz, 2H), 3.58 (s, 3H), 3.39 (s, 3H).

^{13}C NMR (101 MHz, $CDCl_3$) δ 155.3, 151.8, 148.9, 140.8, 132.2, 119.5, 107.0, 49.1, 29.9, 28.1.

HRMS (ESI): m/z $[M+H]^+$ calcd. for $C_{10}H_{13}N_4O_2$: 221.1033; found: 221.1035

N-allyl azathioprine **(31)**

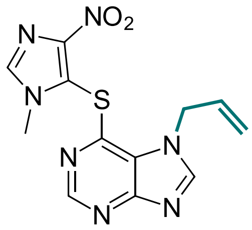

Prepared, following the general procedure C from commercially available starting material with the deviation that short pad silica filtration was conducted using EtOAc:MeOH 10:1 as eluent. Cs_2CO_3 was used as the base. The crude material was purified *via* column chromatography (5 g silica, EtOAc:MeOH 20:1, 10:1) to obtain the title compound as a colorless oil (57 mg, 35 %).

^{1}H NMR (400 MHz, Chloroform-d) δ 8.54 (s, 1H), 8.01 (s, 1H), 7.74 (s, 1H), 6.07 – 5.93 (m, 1H), 5.31 (dq, J = 10.3, 1.2 Hz, 1H), 5.22 (dq, J = 17.0, 1.4 Hz, 1H), 4.85 (dt, J = 5.8, 1.5 Hz, 2H), 3.73 (s, 3H).

^{13}C NMR (101 MHz, $CDCl_3$) δ 156.4, 152.1, 149.9, 144.1, 138.1, 131.2, 131.1, 119.9, 119.1, 116.9, 46.1, 33.3.

HRMS (ESI): m/z $[M+H]^+$ calcd. for $C_{12}H_{12}N_7O_2S$: 318.0768; found: 318.0772

N-Allylbetahistine **(32)**

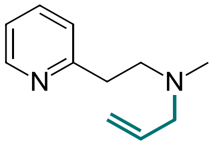

Prepared, following the general procedure C from commercially available starting material with the deviation that 3 equivalents of. Cs_2CO_3 were used as the base, and the short pad silica filtration was conducted using DCM:MeOH 10:1 as the eluent. Without further purification, the title compound was obtained as a slightly yellow oil (86 mg, 98 %).

^{1}H NMR (400 MHz, Chloroform-*d*) δ 8.54 – 8.46 (m, 1H), 7.57 (td, *J* = 7.7, 1.9 Hz, 1H), 7.16 (dt, *J* = 7.8, 1.1 Hz, 1H), 7.12 – 7.04 (m, 1H), 5.92 – 5.77 (m, 1H), 5.21 – 5.07 (m, 2H), 3.06 (dt, *J* = 6.6, 1.3 Hz, 2H), 3.00 – 2.92 (m, 2H), 2.83 – 2.73 (m, 2H), 2.29 (s, 3H).

^{13}C NMR (101 MHz, $CDCl_3$) δ 160.6, 149.4, 136.4, 135.7, 123.3, 121.2, 117.7, 61.0, 57.2, 42.1, 36.3.

HRMS (ESI): m/z $[M+H]^+$ calcd. for $C_{11}H_{17}N_2$: 177.1386; found 177.1383

N-Allyl fluoxetine **(33)** [123604-97-7]

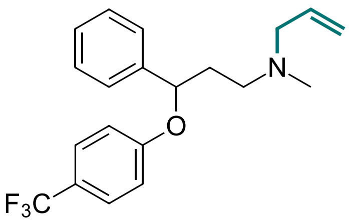

Prepared, following the general procedure C from commercially available starting material. Cs_2CO_3 was used as the base. Without further purification, the title compound was obtained as a yellow oil (134 mg, 77 %)

^1H NMR (400 MHz, Chloroform-d) δ 7.48 – 7.40 (m, 2H), 7.40 – 7.31 (m, 4H), 7.31 – 7.21 (m, 1H), 6.96 – 6.88 (m, 2H), 5.81 (ddt, J = 16.7, 10.2, 6.5 Hz, 1H), 5.30 (dd, J = 8.3, 4.8 Hz, 1H), 5.16 (dq, J = 17.1, 1.6 Hz, 1H), 5.12 – 5.06 (m, 1H), 3.00 (ddt, J = 6.5, 5.1, 1.3 Hz, 2H), 2.56 (ddt, J = 12.5, 7.9, 6.8 Hz, 1H), 2.46 (ddd, J = 12.5, 8.0, 5.5 Hz, 1H), 2.28 – 2.13 (m, 4H), 2.06 – 1.93 (m, 1H).

^{13}C NMR (101 MHz, $CDCl_3$) δ 160.9, 141.4, 135.8, 128.9, 127.9, 126.8 (q, *J* = 3.7 Hz), 126.0, 122.8 (q, *J* = 32.6 Hz), 117.5, 115.9, 78.6, 61.1, 53.3, 42.2, 36.7.

^{19}F NMR (376 MHz, Chloroform-d) δ -61.51.

HRMS (ESI): m/z $[M+H]^+$ calcd. for $C_{20}H_{23}NOS$: 350.1726; found: 350.1727

N-Allyl duloxetine **(34)**

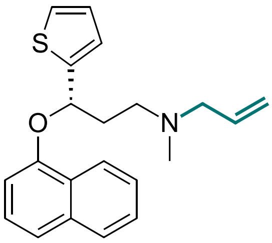

Prepared, following the general procedure C from commercially available starting material. K_2CO_3 was used as the base. Without further purification, the title compound was obtained as a slightly yellow oil (141 mg, 84 %)

^1H NMR (400 MHz, Chloroform-d) δ 8.31 – 8.22 (m, 1H), 7.73 – 7.64 (m, 1H), 7.44 – 7.32 (m, 2H), 7.32 – 7.26 (m, 1H), 7.22 – 7.14 (m, 1H), 7.11 (dd, J = 5.0, 1.2 Hz, 1H), 6.97 (dt, J = 3.4, 1.0 Hz, 1H), 6.84 (dd, J = 5.0, 3.5 Hz, 1H), 6.82 – 6.75 (m, 1H), 5.79 – 5.64 (m, 2H), 5.04 (dq, J = 17.1, 1.6 Hz, 1H), 5.00 – 4.92 (m, 1H), 2.91 (dt, J = 6.5, 1.3 Hz, 2H), 2.58 – 2.43 (m, 2H), 2.43 – 2.29 (m, 1H), 2.16 (s, 3H), 2.15 – 2.05 (m, 1H).

^{13}C NMR (101 MHz, $CDCl_3$) δ 153.7, 145.6, 135.8, 134.7, 127.6, 126.6, 126.4, 126.3, 125.9, 125.3, 124.8, 124.7, 122.4, 120.6, 117.5, 107.2, 74.8, 61.2, 53.3, 42.3, 36.9.

HRMS (ESI): m/z $[M+H]^+$ calcd. for $C_{21}H_{24}NOS$: 338.1573; found 338.1571

N-Allyl paroxetine **(35)**

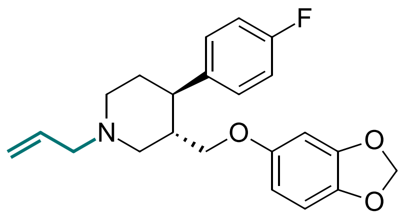

Prepared, following the general procedure C from commercially available starting material with the deviation that the short pad silica filtration was conducted using DCM:MeOH 20:1 as the eluent. K_2CO_3 was used as the base. Without further purification, the title compound was obtained as a red oil (180 mg, 98 %).

^1H NMR (400 MHz, Chloroform-d) δ 7.21 – 7.11 (m, 2H), 7.01 – 6.91 (m, 2H), 6.61 (d, J = 8.5 Hz, 1H), 6.34 (d, J = 2.5 Hz, 1H), 6.12 (dd, J = 8.5, 2.5 Hz, 1H), 6.01 – 5.84 (m, 3H), 5.30 – 5.13 (m, 2H), 3.57 (dd, J = 9.4, 2.9 Hz, 1H),

3.45 (dd, J = 9.4, 6.9 Hz, 1H), 3.31 – 3.22 (m, 1H), 3.15 – 3.00 (m, 3H), 2.47 (td, J = 11.2, 5.1 Hz, 1H), 2.27 – 2.14 (m, 1H), 2.10 – 1.96 (m, 2H), 1.94 – 1.76 (m, 2H).

^{13}C NMR (101 MHz, $CDCl_3$) δ 161.6 (d, *J* = 244.2 Hz), 154.5, 148.2, 141.6, 139.8 (d, *J* = 3.2 Hz), 135.2, 128.9 (d, *J* = 7.8 Hz), 118.1, 115.5 (d, *J* = 21.1 Hz), 107.9, 105.7, 101.2, 98.1, 69.7, 62.3, 57.6, 54.1, 44.2, 42.3, 34.5.

HRMS (ESI): m/z $[M+H]^+$ calcd. for $C_{22}H_{25}FNO_3$: 370.1813; found: 370.1811.

N,N-Diallyl celecoxib **(36)**

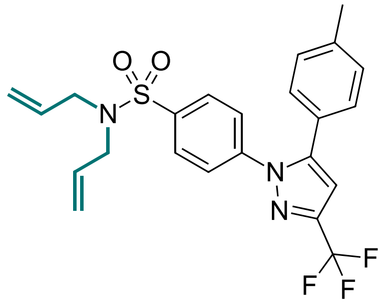

Prepared, following the general procedure C from commercially available starting material with the deviation that 2.5 equivalents of allyl trimethylammonium chloride, and 3 equivalents of Cs_2CO_3 were used. The crude product was purified *via* column chromatography (10 g silica, pentane:EtOAc 20:1, 10:1) to obtain the title compound as colorless oil (201 mg, 87 %)

^{1}H NMR (400 MHz, Chloroform-d) δ 7.85 – 7.77 (m, 2H), 7.50 – 7.42 (m, 2H), 7.20 – 7.13 (m, 2H), 7.13 – 7.05 (m, 2H), 6.74 (s, 1H), 5.66 – 5.51 (m, 2H), 5.20 – 5.10 (m, 4H), 3.81 (dt, J = 6.3, 1.4 Hz, 4H), 2.38 (s, 3H).

^{13}C NMR (101 MHz, $CDCl_3$) δ 145.4, 144.2 (q, *J* = 38.5 Hz), 142.4, 140.2, 139.9, 132.2, 129.8, 128.8, 128.3, 125.8, 125.7, 121.2 (q, *J* = 269.1 Hz), 119.5, 106.3, 49.4, 21.4.

^{19}F NMR (376 MHz, Chloroform-d) δ -62.42.

HRMS (ESI): m/z $[M+H]^+$ calcd. for $C_{23}H_{23}F_3N_3O_2S$: 462.1458; found: 462.1453.

C-Nucleophiles

2,2-Diallylmalononitrile **(37)** [CAS: 90557-34-9]

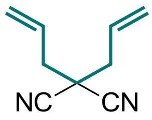

Prepared, following the general procedure C from commercially available starting material with the deviation that 2.5 equivalents of allyl trimethylammonium chloride were used. K_2CO_3 was used as the base. Without further purification, the title compound was obtained as a yellow oil (73 mg, 99 %) yellow oil

Analytical data is in accordance with literature. [53]

^{1}H NMR (400 MHz, Chloroform-d) δ 5.97 – 5.82 (m, 2H), 5.48 – 5.36 (m, 4H), 2.68 (dt, J = 7.3, 1.1 Hz, 4H).

^{13}C NMR (101 MHz, $CDCl_3$) δ 128.5, 123.5, 115.0, 41.0, 37.4.

2-(Isopropenylcarbonyloxy)ethyl 2-acetyl-4-pentenoate **(38)**

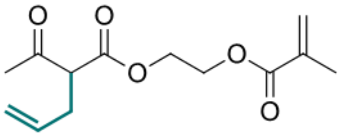

Prepared, following the general procedure C from commercially available starting material. K_2CO_3 was used as the base. The crude product was purified *via* column chromatography (6 g, silica, pentane:EtOAc 30:1 – 10:1) to obtain the title compound as a yellow oil (37 mg, 24 %)

^{1}H NMR (400 MHz, Chloroform-d) δ 6.11 (dq, J = 2.0, 1.1 Hz, 1H), 5.81 – 5.66 (m, 1H), 5.60 (p, J = 1.6 Hz, 1H), 5.09 (dq, J = 17.1, 1.6 Hz, 1H), 5.04 (dq, J = 10.2, 1.3 Hz, 1H), 4.46 – 4.30 (m, 4H), 3.56 (t, J = 7.4 Hz, 1H), 2.67 – 2.53 (m, 2H), 2.23 (s, 3H), 1.94 (dd, J = 1.6, 1.0 Hz, 3H).

^{13}C NMR (101 MHz, $CDCl_3$) δ 202.1, 169.2, 167.1, 136.0, 134.2, 126.3, 117.8, 63.1, 62.2, 59.2, 32.3, 29.3, 18.4.

HRMS (ESI): m/z $[M+Na]^+$ calcd. for $C_{13}H_{18}NaO_5$: 277.1046; found: 277.1047

Ethyl 2-benzoylpent-4-enoate **(39)** [CAS: 63202-75-5]

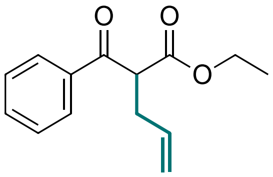

Prepared, following the general procedure C from commercially available starting material. K_2CO_3 was used as the base. The crude product was purified *via* column chromatography (6 g, silica, pentane:Et_2O 100:1 – 30:1) to obtain the title compound as colorless oil (91 mg, 78 %).

Analytical data is in accordance with literature.[54]

^{1}H NMR (400 MHz, Chloroform-d) δ 8.03 – 7.95 (m, 2H), 7.62 – 7.53 (m, 1H), 7.52 – 7.42 (m, 2H), 5.89 – 5.74 (m, 1H), 5.11 (dq, J = 17.1, 1.5 Hz, 1H), 5.03 (dq, J = 10.2, 1.3 Hz, 1H), 4.39 (t, J = 7.2 Hz, 1H), 4.14 (qd, J = 7.1, 2.0 Hz, 2H), 2.83 – 2.67 (m, 2H), 1.16 (t, J = 7.1 Hz, 3H).

^{13}C NMR (101 MHz, $CDCl_3$) δ 194.6, 169.5, 136.3, 134.6, 133.6, 128.8, 128.7, 117.5, 61.6, 54.0, 33.1, 14.1.

HRMS (ESI): m/z $[M+H]^+$ calcd. for $C_{14}H_{17}O_3$: 233.1172; found: 233.1170

4-Methyl-4-nitro-1-pentene **(40)** [CAS: 81500-64-3]

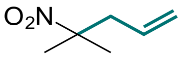

Prepared, following the general procedure C from commercially available starting material. K_2CO_3 was used as the base. Without further purification, the title compound was obtained as a colorless oil (45 mg, 70 %).

Analytical data is in accordance with literature. [55]

^{1}H NMR (400 MHz, Chloroform-d) δ 5.76 – 5.61 (m, 1H), 5.22 – 5.10 (m, 2H), 2.64 (dd, J = 7.5, 1.4 Hz, 2H), 1.59 (s, 6H).

^{13}C NMR (101 MHz, $CDCl_3$) δ 131.2, 120.5, 87.9, 5.12, 25.6.

---

tert-Butyl 2-allyl-2-cyano-4-pentenoate **(41)**

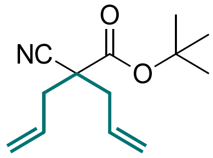

Prepared, following the general procedure C from commercially available starting material with the deviation that 2.5 equivalents of allyl trimethylammonium chloride were used. Cs_2CO_3 was used as the base. Without further purification, the title compound was obtained as a colorless oil (105 mg, 95 %).

^1H NMR (400 MHz, Chloroform-d) δ 5.89 – 5.74 (m, 2H), 5.28 – 5.18 (m, 4H), 2.66 – 2.55 (m, 2H), 2.55 – 2.44 (m, 2H), 1.48 (s, 9H).

^{13}C NMR (101 MHz, $CDCl_3$) δ 167.0, 130.8, 120.8, 118.9, 84.3, 49.8, 40.9, 28.0.

HRMS (ESI): m/z $[M+H]^+$ calcd. for $C_{13}H_{20}NO_2$: 222.1489; found: 222.1486

3-(4-Biphenylyloxy)cyclohexene **(42)**

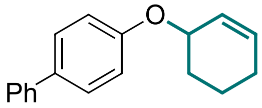

Prepared, following the general procedure D from commercially available starting material. K_2CO_3 was used as the base. The crude material was purified *via* column chromatography (dry load, 5 g silica, pentane:EtOAc 50:1) to obtain the title compound as a white solid (108 mg, 86 %).

^{1}H NMR (400 MHz, Chloroform-d) δ 7.63 – 7.51 (m, 4H), 7.49 – 7.40 (m, 2H), 7.37 – 7.29 (m, 1H), 7.08 – 7.00 (m, 2H), 6.03 (dtd, J = 10.1, 3.6, 1.2 Hz, 1H), 5.94 (dq, J = 10.1, 2.3 Hz, 1H), 4.92 – 4.83 (m, 1H), 2.26 – 1.83 (m, 5H), 1.76 – 1.62 (m, 1H).

^{13}C NMR (101 MHz, $CDCl_3$) δ 157.5, 141.0, 133.8, 132.4, 128.8, 128.3, 126.8, 126.7, 126.4, 116.2, 71.1, 28.4, 25.2, 19.1.

HRMS (ESI): m/z $[M+H]^+$ calcd. for $C_{18}H_{19}O$: 251.1430; found 251.1431

p-(2-Cyclohexen-1-yloxy)methoxybenzene **(43)** [CAS: 175735-18-9]

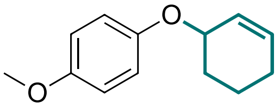

Prepared, following the general procedure D from commercially available starting material. K_2CO_3 was used as the base. The crude material was purified *via* column chromatography (dry load, 5 g silica, pentane:EtOAc 80:1) to obtain the title compound as a colorless oil (75 mg, 74 %)

Analytical data is in accordance with literature.[1a]

^{1}H NMR (400 MHz, $CDCl_3$) δ 6.93 – 6.78 (m, 4H), 6.00 – 5.91 (m, 1H), 5.87 (dq, *J* = 10.0, 2.3 Hz, 1H), 4.72 – 4.63 (m, 1H), 3.77 (s, 3H), 2.20 – 1.77 (m, 5H), 1.71 – 1.55 (m, 1H).

^{13}C NMR (101 MHz, $CDCl_3$) δ 154.0, 152.0, 132.0, 126.8, 117.5, 114.8, 72.1, 55.8, 28.5, 25.3, 19.1.

HRMS (ESI): m/z $[M+Na]^+$ calcd. for $C_{13}H_{16}NaO_2$: 227.1043; found: 227.1048.

(E)-Cinnamyl 1,1'-biphenyl ether **(44)** [CAS: 2410719-96-7]

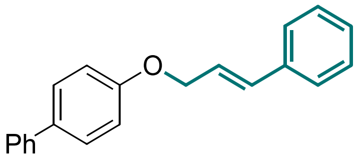

Prepared, following the general procedure D from commercially available starting material. K_2CO_3 was used as the base. Without further purification, the title compound was obtained as a slightly yellow oil (137 mg, 96 %).

Analytical data is in accordance with literature. [56]

^{1}H NMR (400 MHz, Chloroform-d) δ 7.61 – 7.51 (m, 4H), 7.48 – 7.38 (m, 4H), 7.38 – 7.26 (m, 4H), 7.09 – 7.01 (m, 2H), 6.77 (dt, J = 16.0, 1.6 Hz, 1H), 6.46 (dt, J = 16.0, 5.8 Hz, 1H), 4.76 (dd, J = 5.8, 1.5 Hz, 2H).

^{13}C NMR (101 MHz, CDCl3) δ 158.3, 140.9, 136.5, 134.1, 133.2, 128.8, 128.7, 128.3, 128.0, 126.8, 126.8, 126.7, 124.5, 115.2, 68.8.

^{13}C NMR (101 MHz, $CDCl_3$) δ 158.4, 140.9, 136.6, 134.1, 133.2, 128.9, 128.8, 128.3, 128.1, 126.9, 126.8, 126.7, 124.6, 115.2, 68.9

4-[(E)-6,6-Dimethyl-2-hepten-4-ynyloxy]biphenyl **(45)**

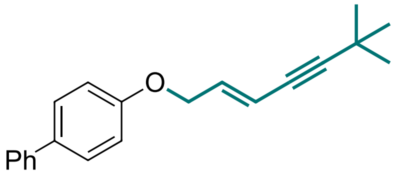

Prepared, following the general procedure D from commercially available starting material with the deviation that 2 mol% of Pd[(allyl)Cl]$_2$ and 4 mol% of *rac*-BINAP were used. K_2CO_3 was used as the base. Without further purification, the title compound was obtained as yellow solid (144 mg, 99 %).

^{1}H NMR (400 MHz, Chloroform-d) δ 7.61 – 7.49 (m, 4H), 7.43 (t, J = 7.7 Hz, 2H), 7.36 – 7.27 (m, 1H), 7.02 – 6.94 (m, 2H), 6.24 (dt, J = 15.9, 5.5 Hz, 1H), 5.88 (dt, J = 15.9, 1.7 Hz, 1H), 4.61 (dd, J = 5.5, 1.7 Hz, 2H), 1.27 (s, 9H).

^{13}C NMR (101 MHz, $CDCl_3$) δ 158.1, 140.9, 135.9, 134.2, 128.9, 128.3, 126.9, 126.8, 115.1, 113.5, 100.3, 76.8, 68.0, 31.1, 28.1.

HRMS (ESI): m/z [M+H]$^+$ calcd. for $C_{21}H_{23}O$: 291.1743; found: 291.1742

4-biphenylyl 2,4-pentadienyl ether **(46)** [CAS: 103993-00-6]

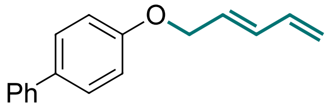

Prepared, following the general procedure D from commercially available starting material. K_2CO_3 was used as the base. The crude material was purified *via* column chromatography (5 g silica, pentane:Et_2O 100:1) to obtain the title compound as white solid (96 mg, 81 %)

^{1}H NMR (400 MHz, Chloroform-d) δ 7.60 – 7.50 (m, 4H), 7.47 – 7.39 (m, 2H), 7.36 – 7.29 (m, 1H), 7.05 – 6.96 (m, 2H), 6.49 – 6.33 (m, 2H), 6.02 – 5.90 (m, 1H), 5.35 – 5.23 (m, 1H), 5.23 – 5.12 (m, 1H), 4.63 (d, J = 5.2 Hz, 2H).

^{13}C NMR (101 MHz, $CDCl_3$) δ 158.3, 140.9, 136.2, 134.1, 133.9, 128.9, 128.5, 128.3, 126.9, 126.8, 118.4, 115.2, 68.3.

HRMS (ESI): m/z [M+H]$^+$ calcd. for $C_{17}H_{17}O$: 237.1274; found: 237.1276

4-[(E)-1-Methyl-2-butenyloxy]biphenyl **(47)**

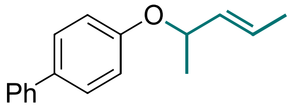

Prepared, following the general procedure D from commercially available starting material. K_2CO_3 was used as the base. The crude material was purified *via* column chromatography (5 g silica, pentane:EtOAc 80:1) to obtain the title compound as a colorless oil (83 mg, 70 %)

^{1}H NMR (400 MHz, $CDCl_3$) δ 7.63 – 7.57 (m, 2H), 7.57 – 7.50 (m, 2H), 7.45 (dd, J = 8.5, 6.9 Hz, 2H), 7.38 – 7.29 (m, 1H), 7.06 – 6.96 (m, 2H), 5.86 – 5.73 (m, 1H), 5.62 (ddq, J = 15.4, 6.5, 1.6 Hz, 1H), 4.85 (p, J = 6.4 Hz, 1H), 1.84 – 1.72 (m, 3H), 1.48 (d, J = 6.3 Hz, 3H).

^{13}C NMR (101 MHz, $CDCl_3$) δ 157.8, 141.0, 133.7, 132.3, 128.8, 128.1, 127.5, 126.8, 126.7, 116.4, 74.6, 21.7, 17.9.

HRMS (ESI): m/z [M+H]$^+$ calcd. for $C_{17}H_{19}O$: 239.1430; found: 239.1435.

N-2-Cyclohexen-1-yl-N-methyl(p-fluorophenyl)amine **(48)**

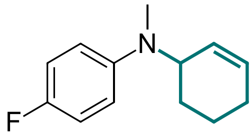

Prepared, following the general procedure D from commercially available starting material. Cs_2CO_3 was used as the base. The crude material was purified *via* column chromatography (5 g silica, pentane:Et_2O 95:5) to obtain the title compound as yellow oil (52 mg, 51 %)

^{1}H NMR (400 MHz, $CDCl_3$) δ 6.99 – 6.88 (m, 2H), 6.78 – 6.62 (m, 2H), 5.96 – 5.84 (m, 1H), 5.68 – 5.59 (m, 1H), 4.39 – 4.29 (m, 1H), 2.74 (s, 3H), 2.04 (dq, J = 5.5, 2.8 Hz, 2H), 1.88 – 1.73 (m, 2H), 1.72 – 1.50 (m, 2H).

^{13}C NMR (101 MHz, $CDCl_3$) δ 155.5 (d, *J* = 235.2 Hz), 146.7, 130.8, 130.0, 115.6 (d, *J* = 21.9 Hz), 114.5 (d, *J* = 7.1 Hz), 56.2, 33.0, 25.1, 21.7.

^{19}F NMR (376 MHz, $CDCl_3$) δ -129.20.

HRMS (ESI): m/z $[M+H]^+$ calcd. for $C_{13}H_{17}FN$: 206.1340; found: 206.1336.

N-Methyl[2-(2-pyridyl)ethyl][*(E)*-3-phenyl-2-propenyl]amine **(49)**

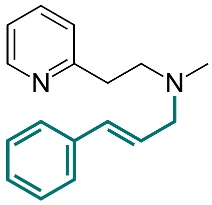

Prepared, following the general procedure D from commercially available starting material with the deviation that 3 equivalents of Cs_2CO_3 were used as the base and silica filtration was conducted using DCM:MeOH 20:1 as eluent.. The crude material was purified *via* column chromatography (5 g silica, DCM:MeOH 10:1) to obtain the title compound as yellow oil (91 mg, 72 %)

^{1}H NMR (400 MHz, $CDCl_3$) δ 8.59 (ddd, *J* = 4.9, 1.9, 0.9 Hz, 1H), 7.64 (td, *J* = 7.6, 1.9 Hz, 1H), 7.47 – 7.33 (m, 4H), 7.33 – 7.20 (m, 2H), 7.16 (ddd, *J* = 7.6, 4.9, 1.2 Hz, 1H), 6.58 (dt, *J* = 16.0, 1.5 Hz, 1H), 6.34 (dt, *J* = 15.9, 6.7 Hz, 1H), 3.31 (dd, *J* = 6.7, 1.4 Hz, 2H), 3.08 (dd, *J* = 9.4, 6.2 Hz, 2H), 2.98 – 2.87 (m, 2H), 2.43 (s, 3H).

^{13}C NMR (101 MHz, $CDCl_3$) δ 160.4, 149.3, 137.1, 136.4, 132.6, 128.5, 127.4, 127.3, 126.3, 123.2, 121.3, 60.2, 57.2, 42.1, 36.3.

HRMS (ESI): m/z $[M+H]^+$ calcd. for $C_{17}H_{21}N_2$: 253.1700; found: 253.1697.

Ethyl [(E)-3-phenyl-2-propenyl]benzoylacetate **(50)**

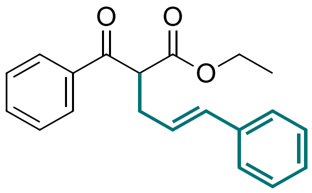

Prepared, following the general procedure D from commercially available starting material. K_2CO_3 was used as the base. The crude material was purified *via* column chromatography (5 g silica, pentane:Et_2O 100:1, 50:1, 20:1, 10:1) to obtain the title compound as a colorless oil (91 mg, 59 %)

^{1}H NMR (400 MHz, $CDCl_3$) δ 8.07 – 7.95 (m, 2H), 7.65 – 7.54 (m, 1H), 7.53 – 7.42 (m, 2H), 7.33 – 7.24 (m, 4H), 7.23 – 7.16 (m, 1H), 6.49 (dt, *J* = 15.8, 1.4 Hz, 1H), 6.20 (dt, *J* = 15.7, 7.2 Hz, 1H), 4.46 (dd, *J* = 7.6, 6.8 Hz, 1H), 4.16 (qd, *J* = 7.1, 2.7 Hz, 2H), 3.01 – 2.83 (m, 2H), 1.17 (t, *J* = 7.1 Hz, 3H).

^{13}C NMR (101 MHz, $CDCl_3$) δ 194.6, 169.5, 137.2, 136.3, 133.7, 132.8, 128.9, 128.8, 128.6, 127.5, 126.3, 126.2, 61.6, 54.5, 32.5, 14.2.

HRMS (ESI): m/z $[M+H]^+$ calcd. for $C_{20}H_{21}O_3$: 309.1485; found: 309.1485.

Ethyl *(E)*-2-benzoyl-8,8-dimethyl-4-nonen-6-ynoate **(51)**

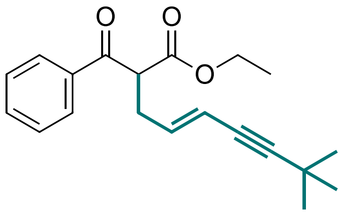

Prepared, following the general procedure D from commercially available starting material. K_2CO_3 was used as the base. The crude material was purified *via* column chromatography (5 g silica, pentane:Et_2O 70:1, 50:1, 20:1, 10:1) to obtain the title compound as a colorless oil (90 mg, 58 %).

^{1}H NMR (400 MHz, $CDCl_3$) δ 8.05 – 7.91 (m, 2H), 7.58 (tq, *J* = 6.9, 1.7 Hz, 1H), 7.52 – 7.42 (m, 2H), 5.99 (dt, *J* = 15.7, 7.3 Hz, 1H), 5.57 (dt, *J* = 15.7, 1.5 Hz, 1H), 4.35 (t, *J* = 7.2 Hz, 1H), 4.13 (qd, *J* = 7.1, 1.3 Hz, 2H), 2.84 – 2.68 (m, 2H), 1.22 – 1.11 (m, 12H).

^{13}C NMR (101 MHz, $CDCl_3$) δ 194.2, 169.2, 138.0, 136.1, 133.7, 128.9, 128.8, 113.2, 98.4, 77.1, 61.7, 54.0, 32.2, 31.1, 27.9, 14.1.

HRMS (ESI): m/z $[M+H]^+$ calcd. for $C_{20}H_{25}O_3$: 313.1798; found: 313.1799.

Enantioselective Allylation

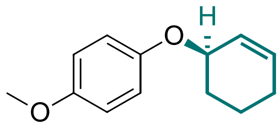

(R)-43

(R)-Cyclohex-2-enyl 4'-methoxyphenyl ether ***(R)*-43** [CAS: 854735-43-6]

Prepared, following the general procedure D from commercially available starting material with the deviation that *(R)*-SEGPHOS (CAS: 244261-66-3) was used as the ligand. K_2CO_3 was used as the base. The crude material was purified *via* column chromatography (dry load on celite, 5 g silica, pentane:Et_2O 80:1) to obtain the enantioenriched titlecompound as a colorless oil (74 mg, 73 %, 52 % ee).

NMR-Spectra matched with the spectra obtained for rac-**43**.

$[\alpha]_D^{20}$ +63.84 (c = 1.0, $CHCl_3$)

The enantiomers were separated using CHIRALCEL OD column eluting with 99.5:0.5 heptane:iso-propanol at 1.0 mL/min. Retention times for racemic mixture: ***(R)*-43** 10.87 min; ***(S)*-43** 13.70 min. Retention times major isomer *(R)* 10.89 min; minor isomer *(S)* 13.64 min. The absolute configurations of the major isomers were assigned based on reported data in literature. [27]

NMR-Spectra

2-Cyclohexen-1-yltrimethylammonium chloride **(III)**

1H-NMR
400.13 Hz

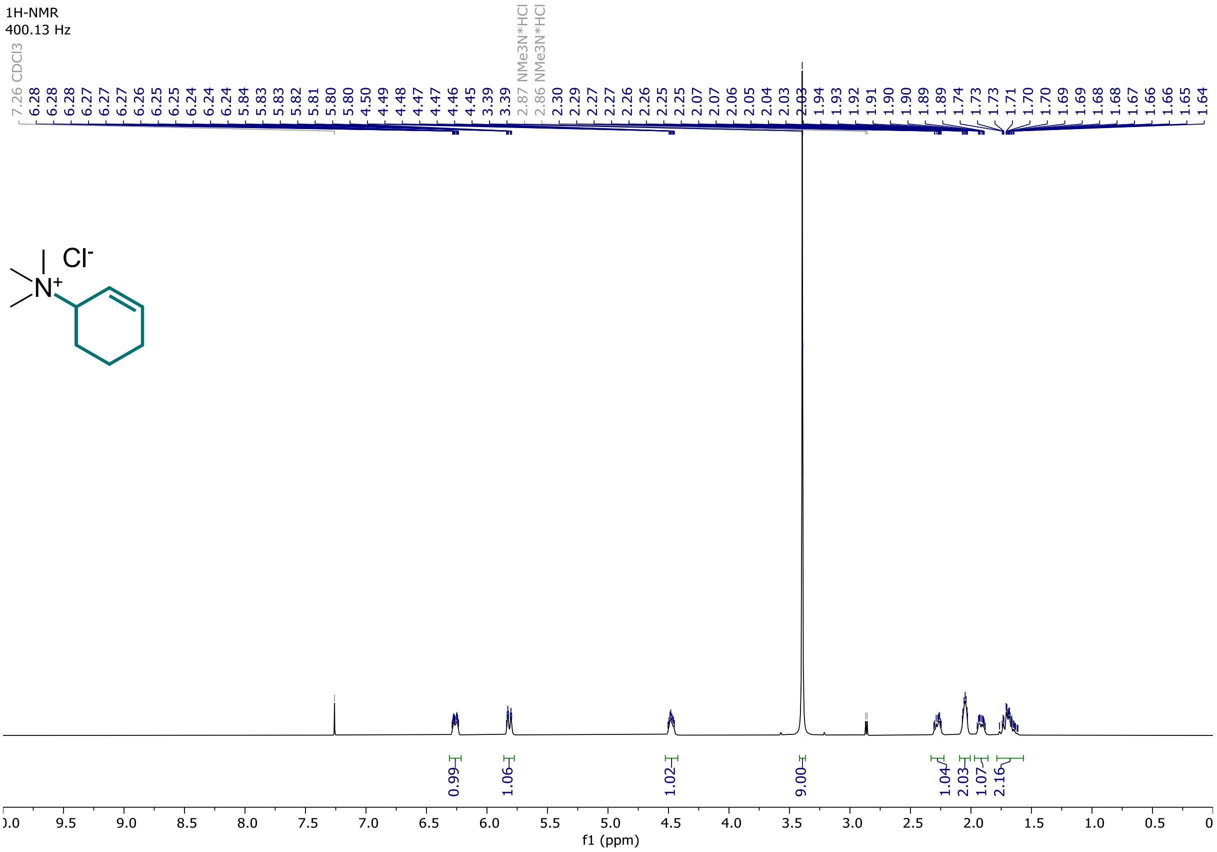

13C-NMR
100.62 Hz
CDCl3

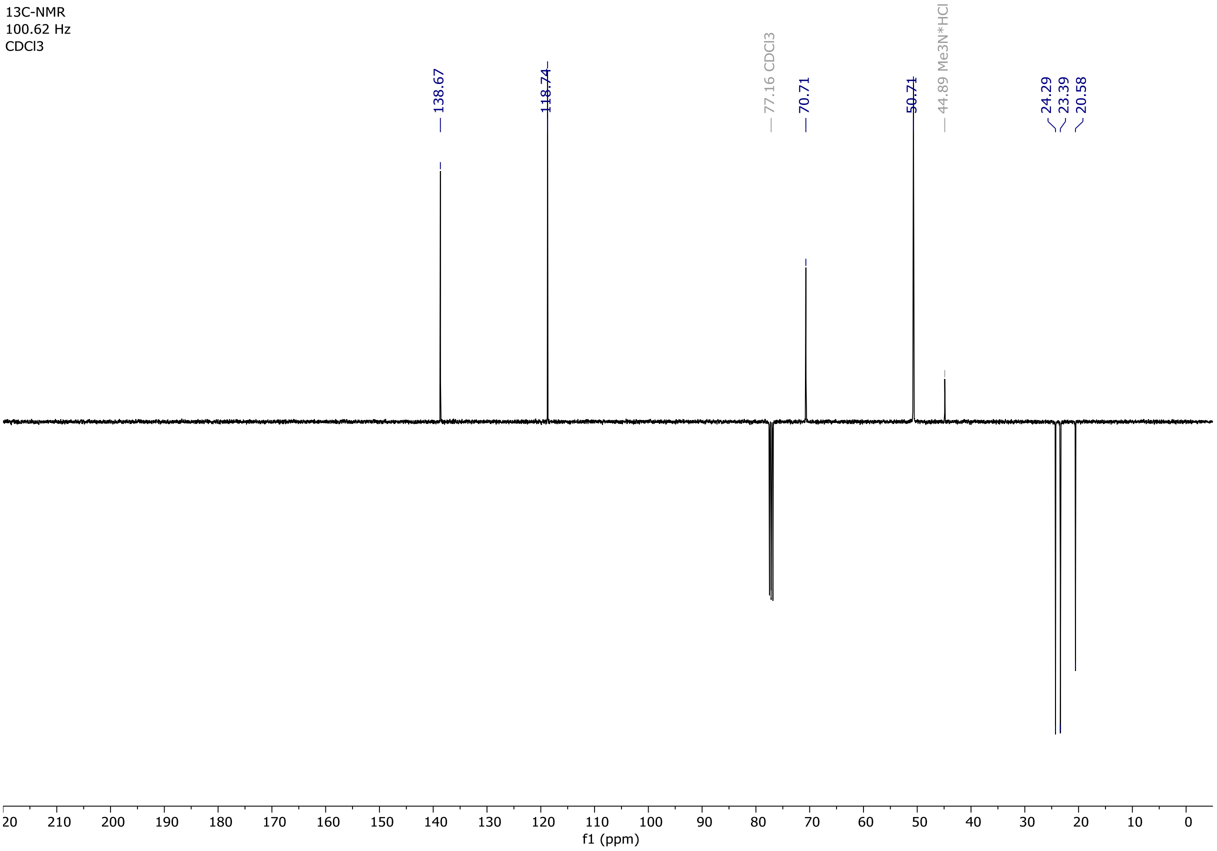

(3-Phenyl-2-propenyl)trimethylammonium chloride **(IV)**

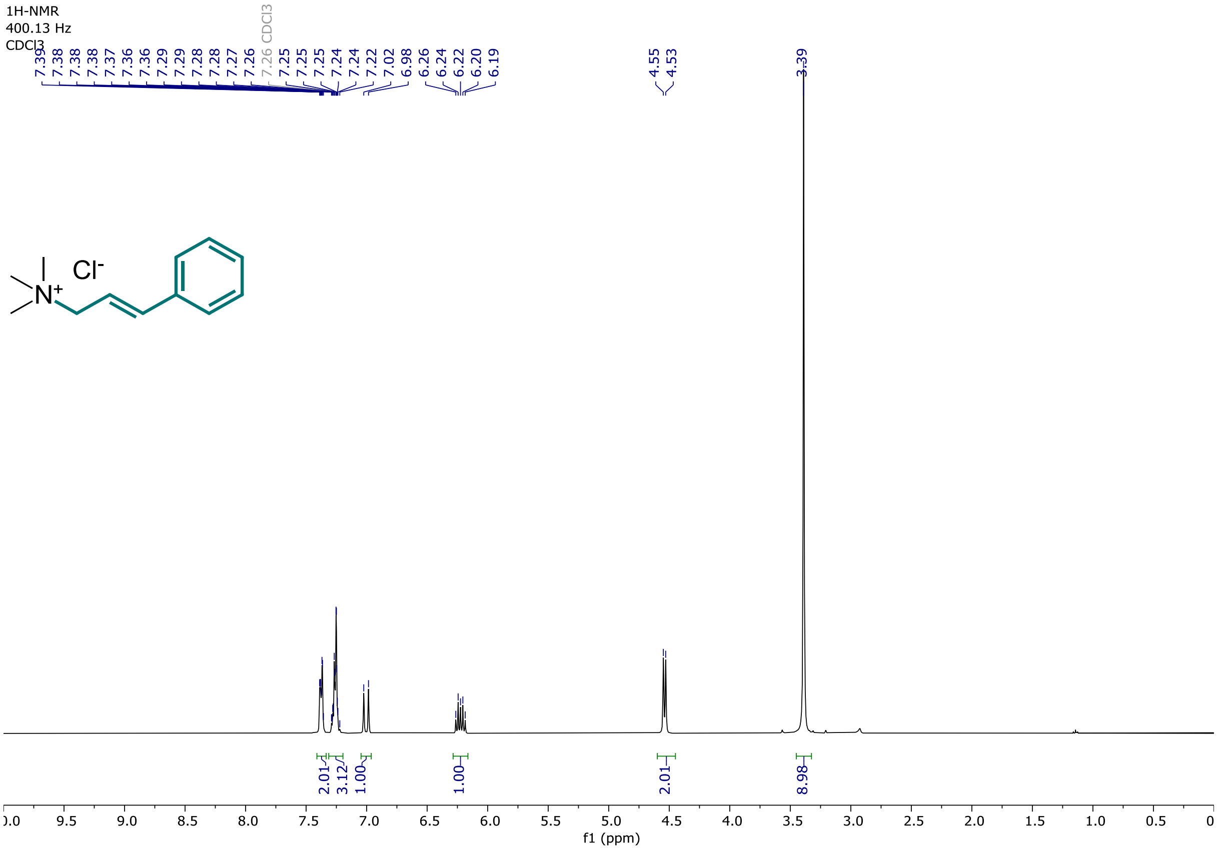

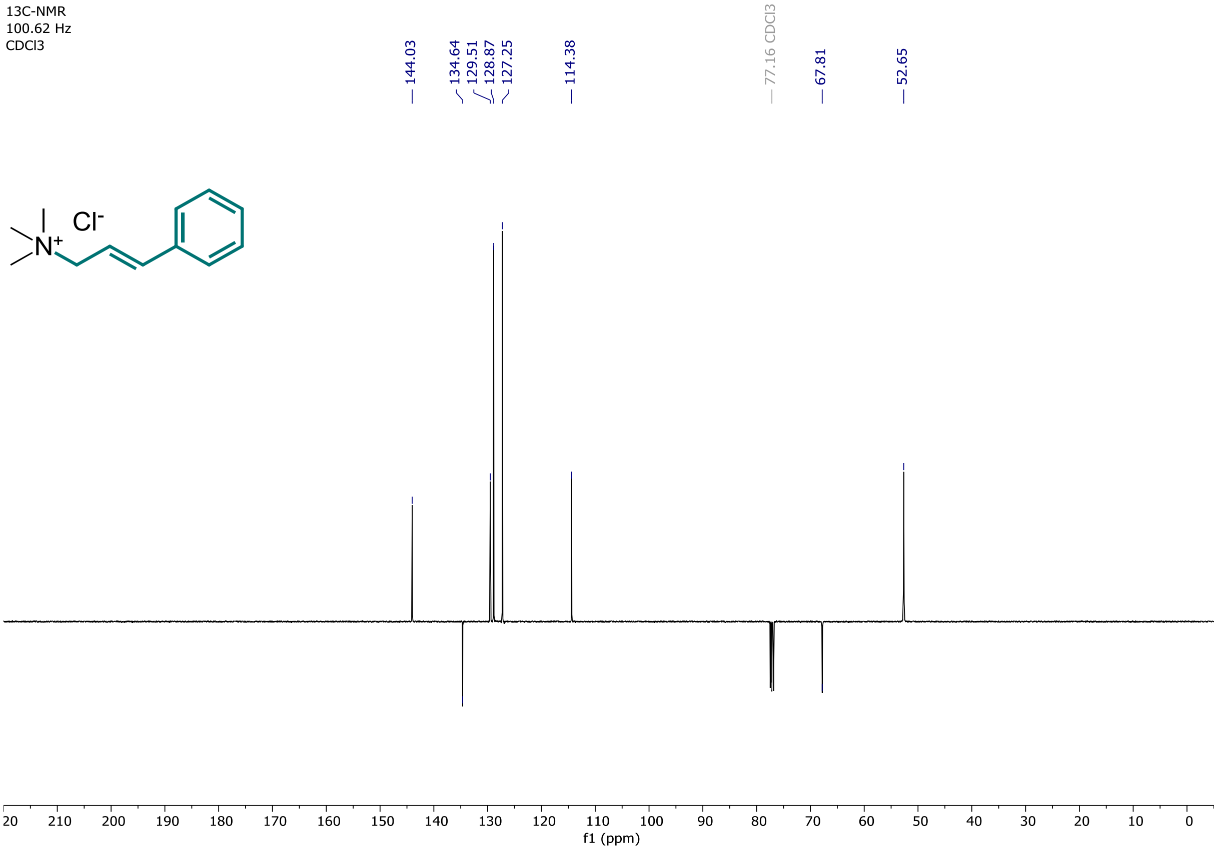

(6,6-Dimethyl-2-hepten-4-ynyl)trimethylammonium chloride **(V)**

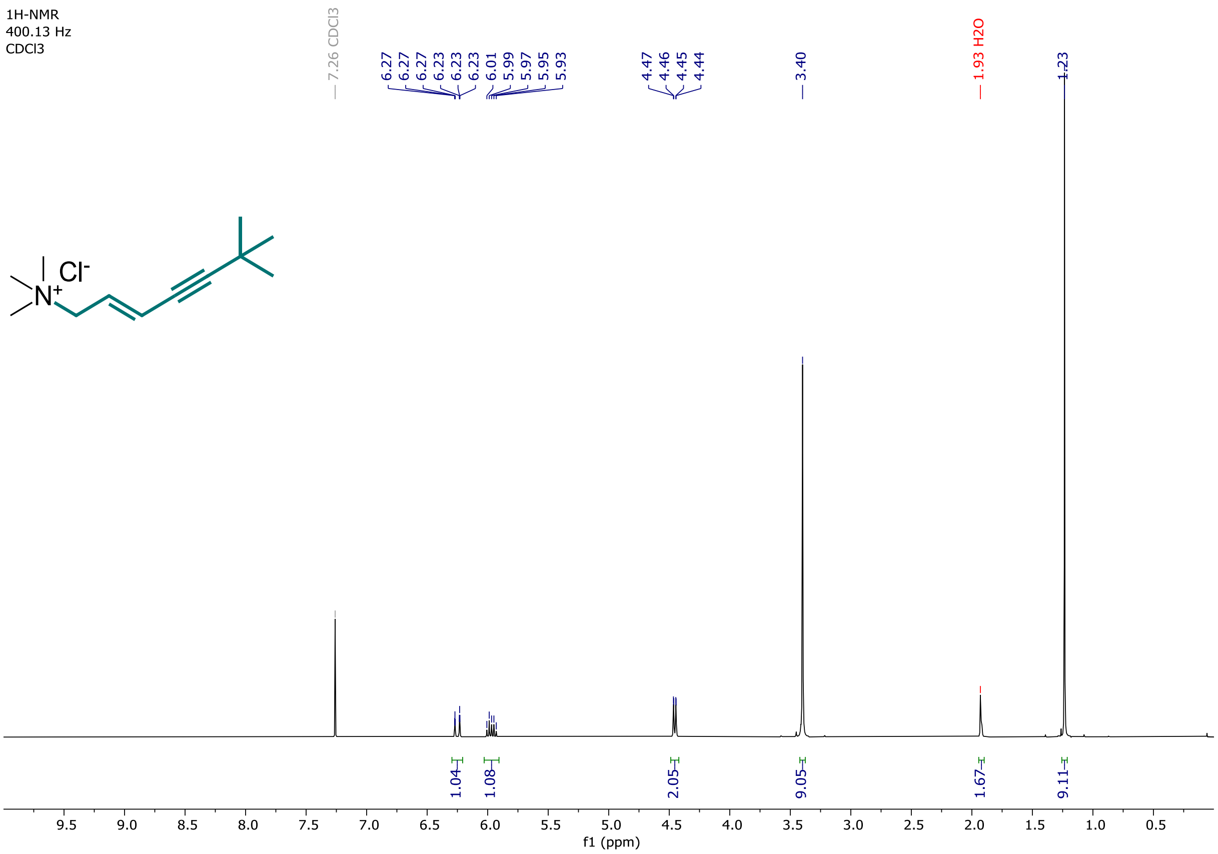

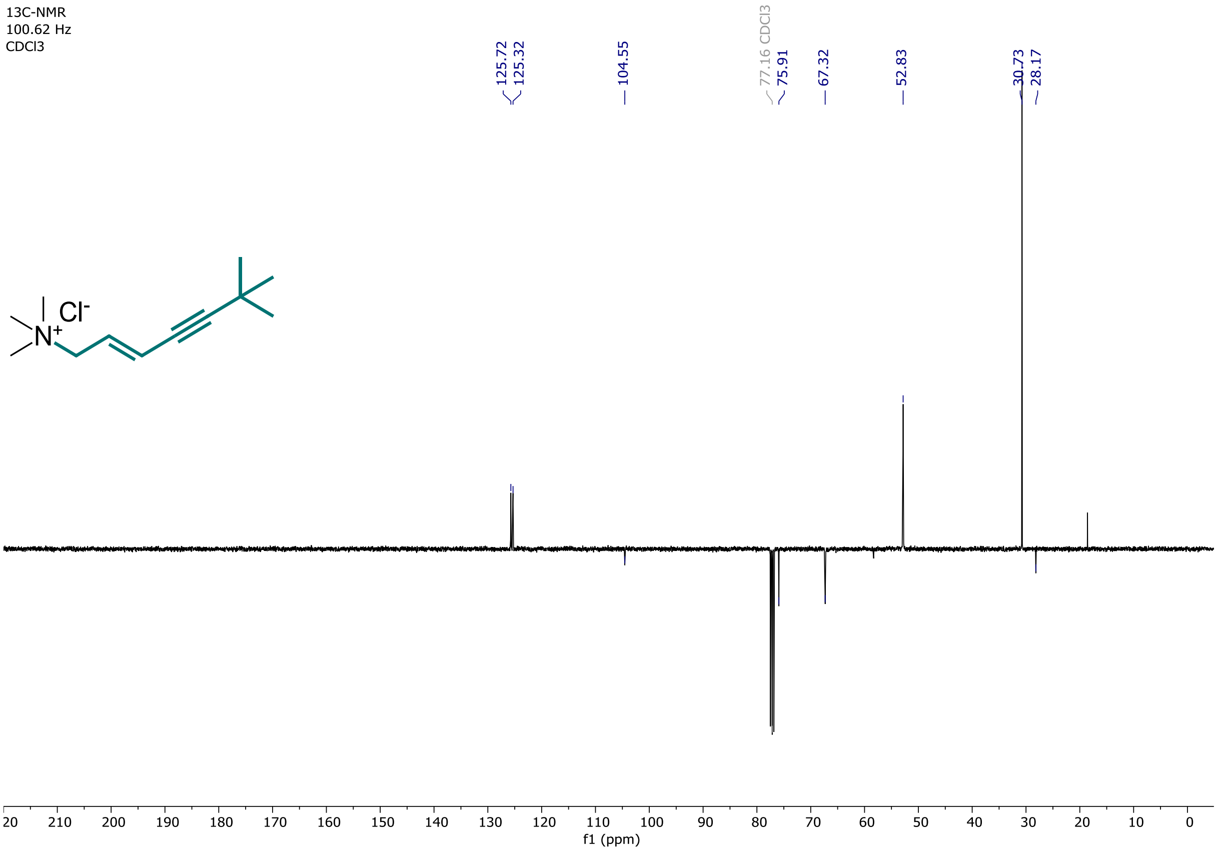

2,4-Pentadienyltrimethylammonium chloride **(VI)**

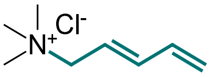

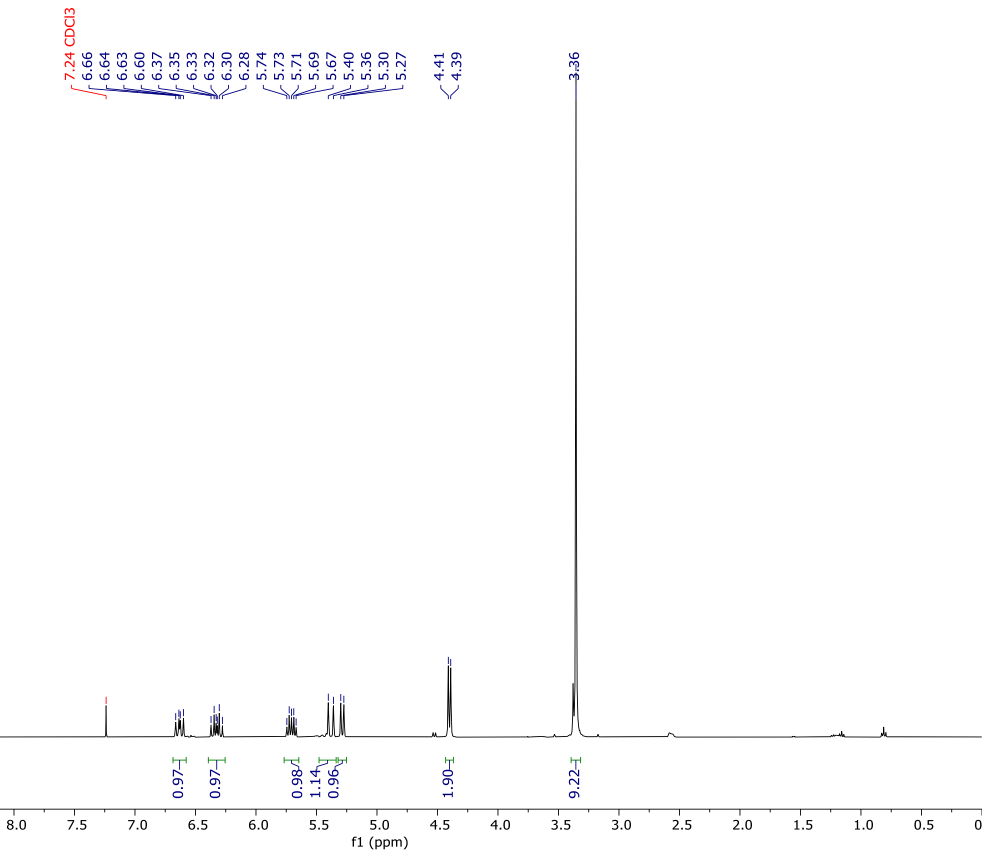

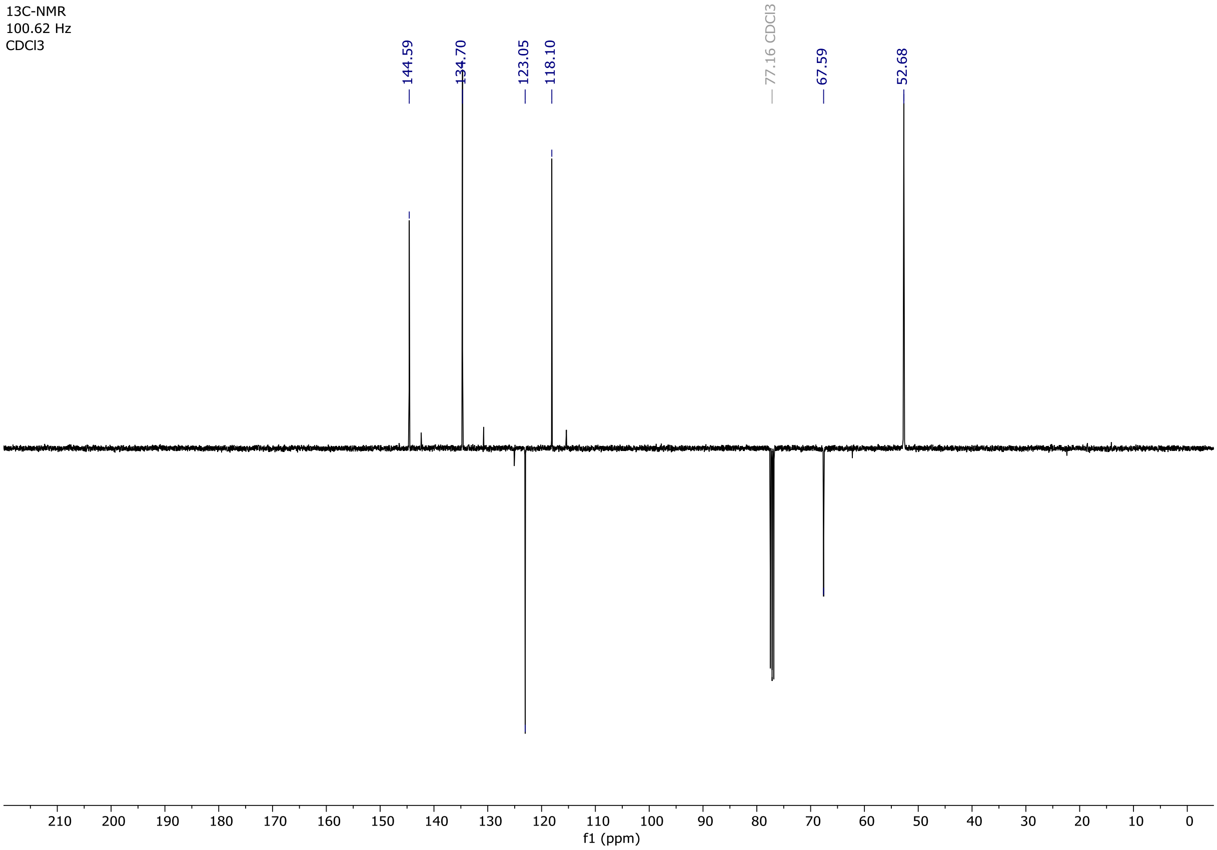

(1-Methyl-2-butenyl)trimethylammonium chloride **(VII)**

1H-NMR
400.13 Hz
CDCl3

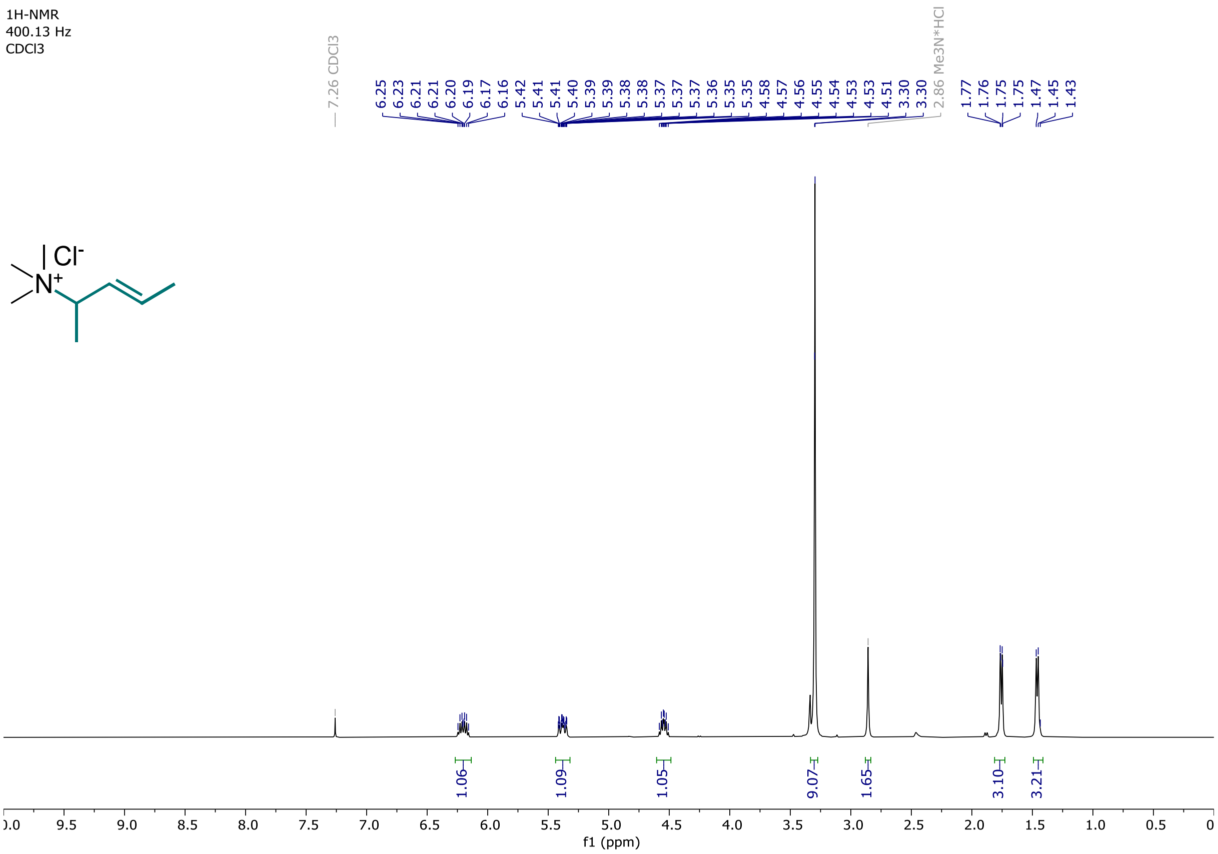

13C-NMR
100.62 Hz
CDCl3

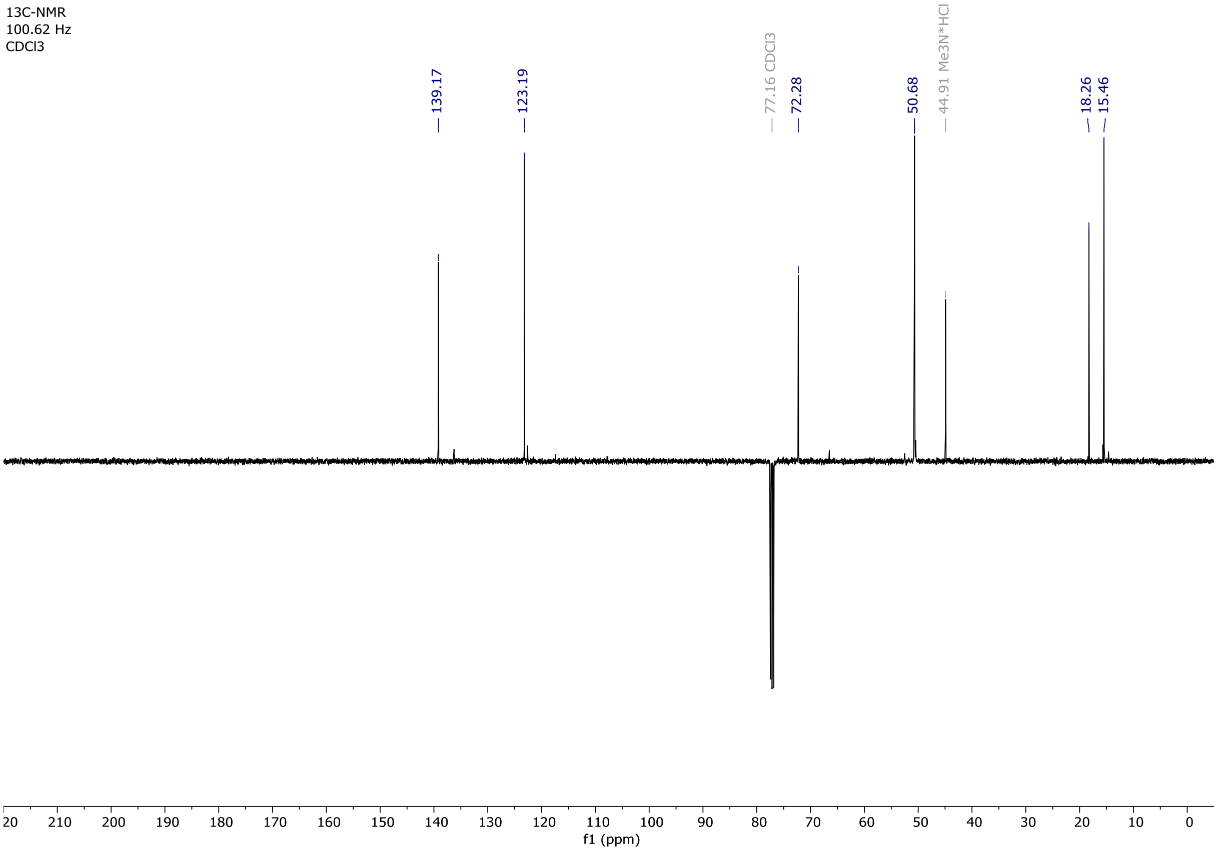

4-Allyloxy-biphenyl (**1**)

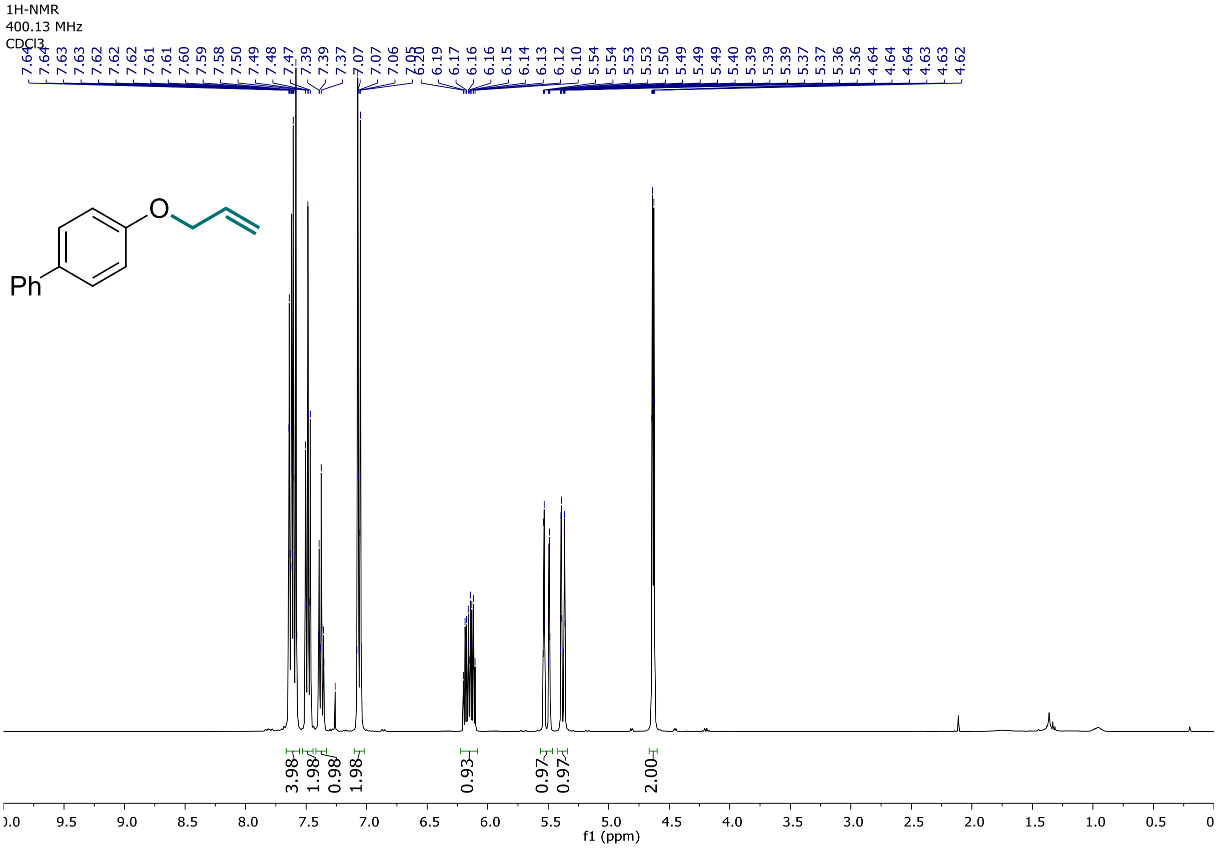

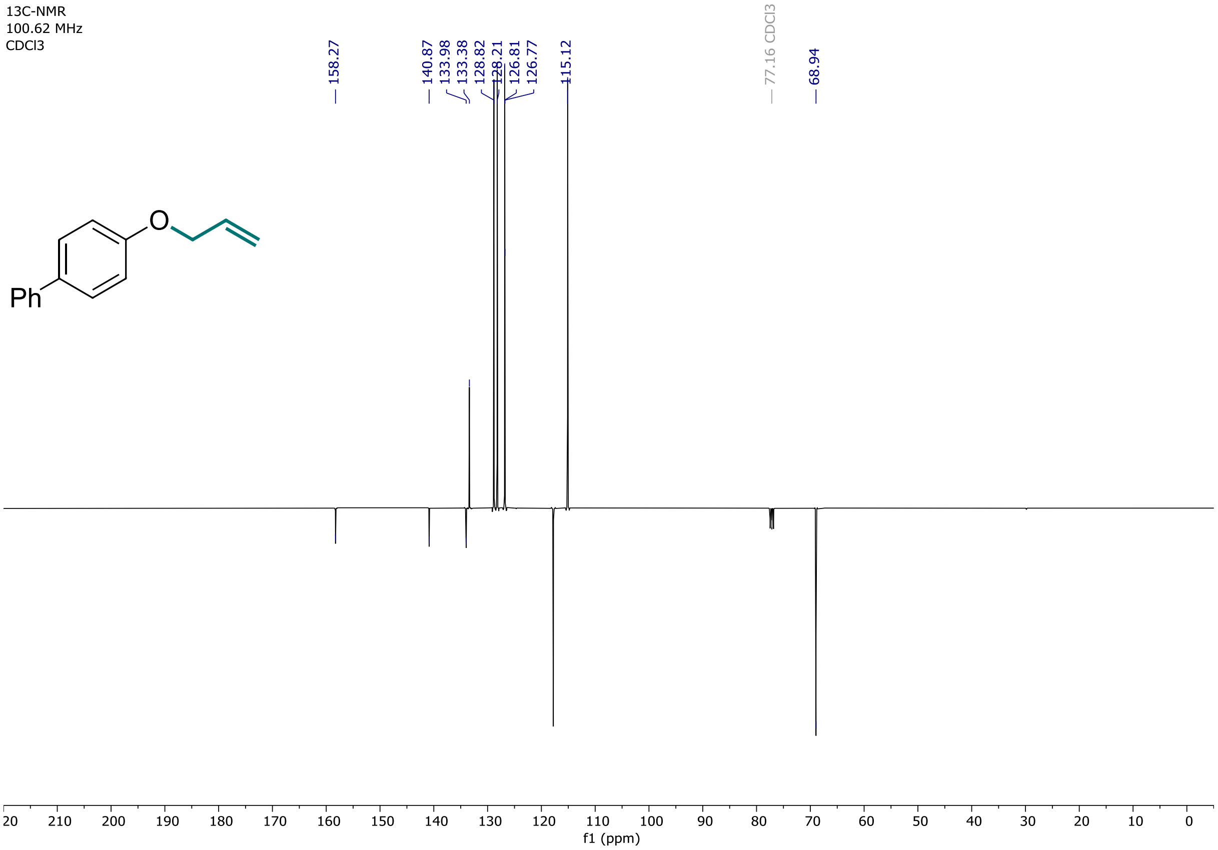

Allyl 4-(tert-butyl)phenyl ether **(2)**

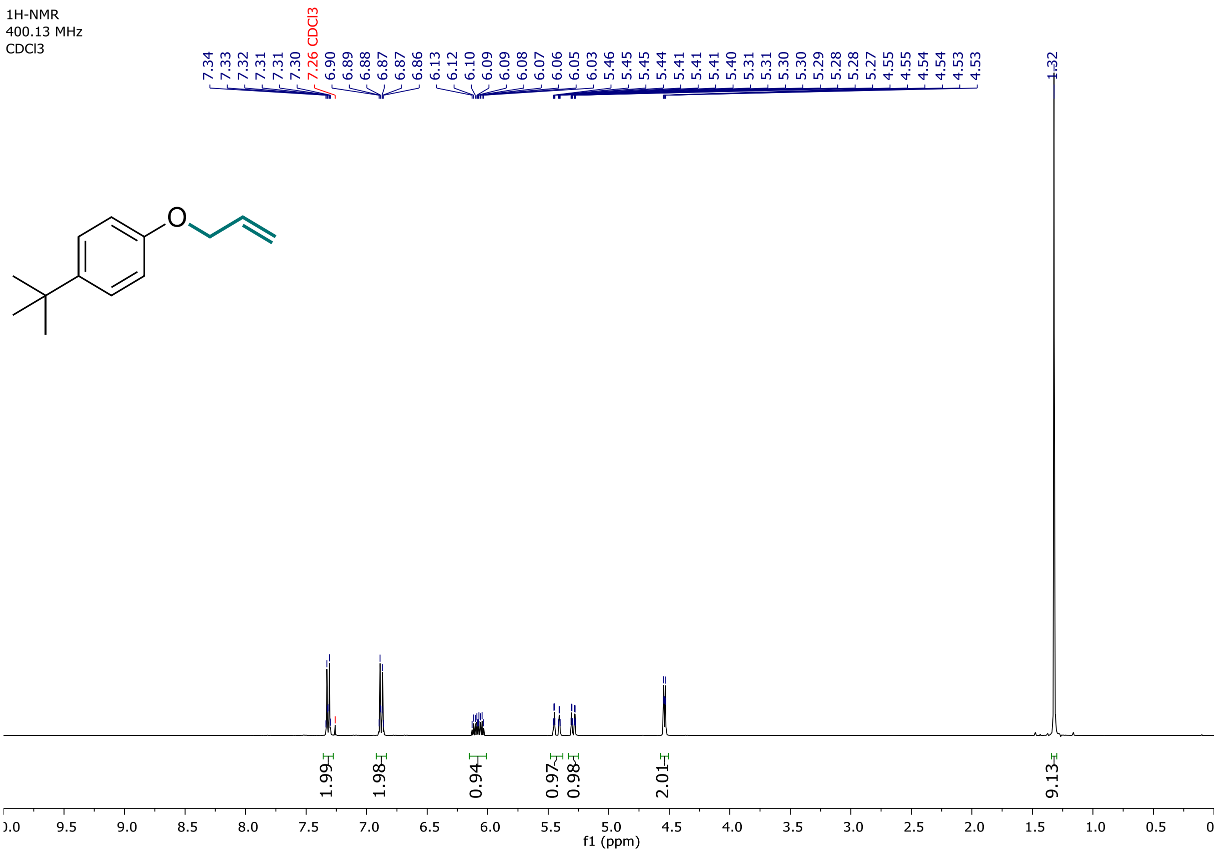

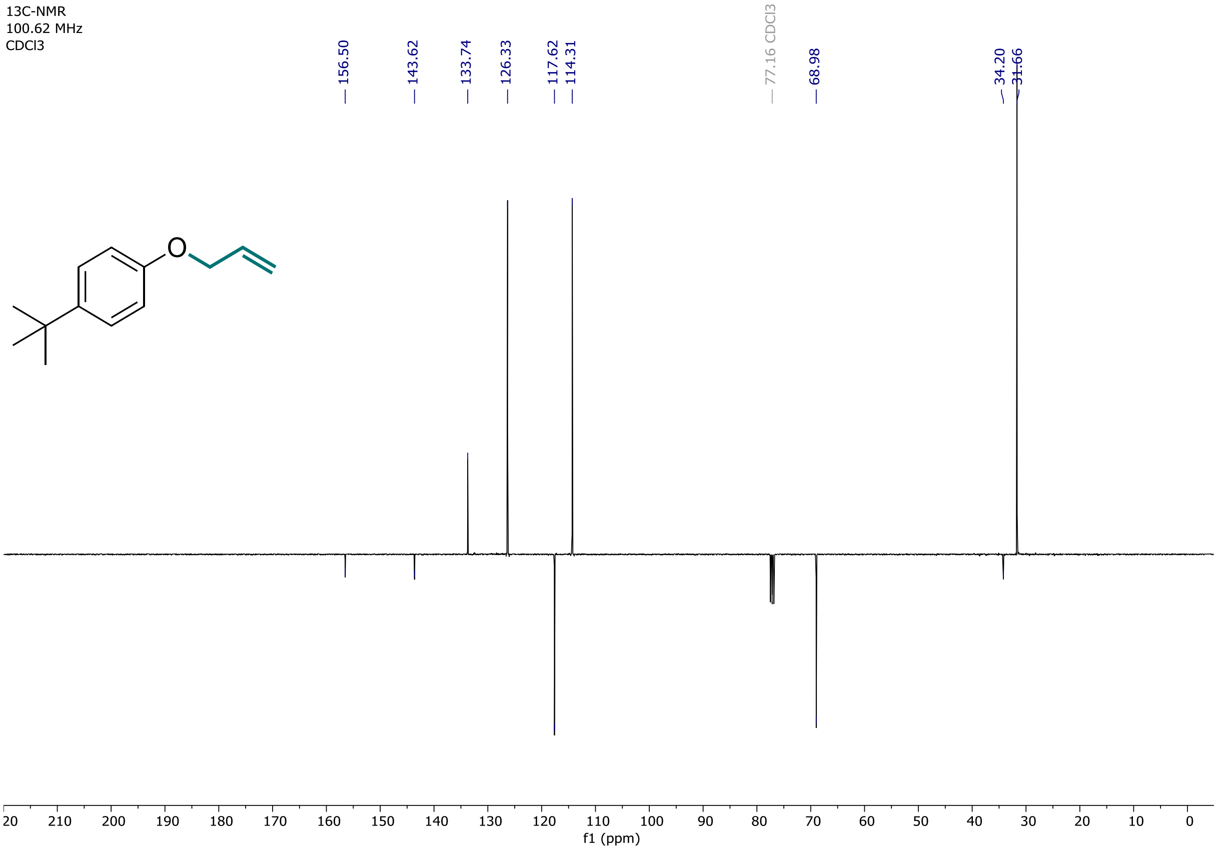

Allyl phenyl ether **(3)**

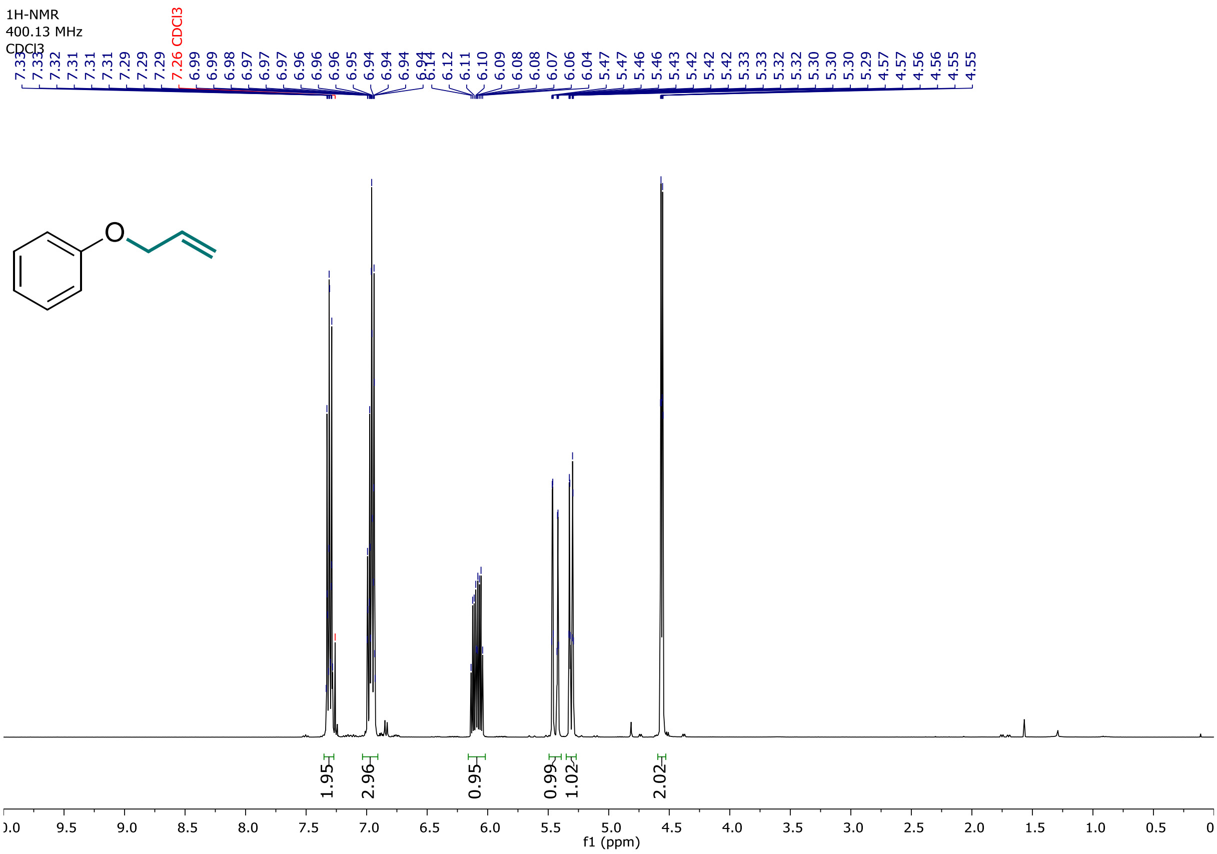

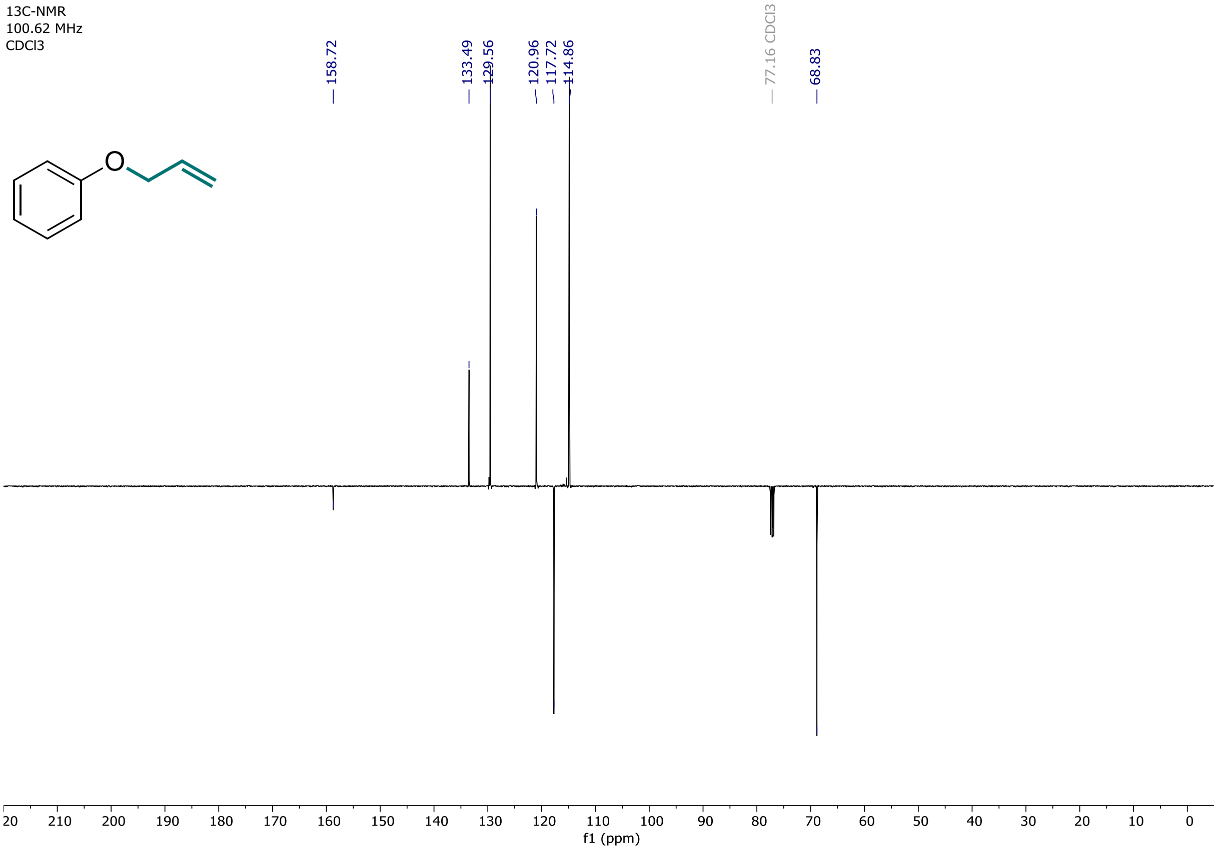

1-(Allyloxy)-4-fluorobenzene **(4)**

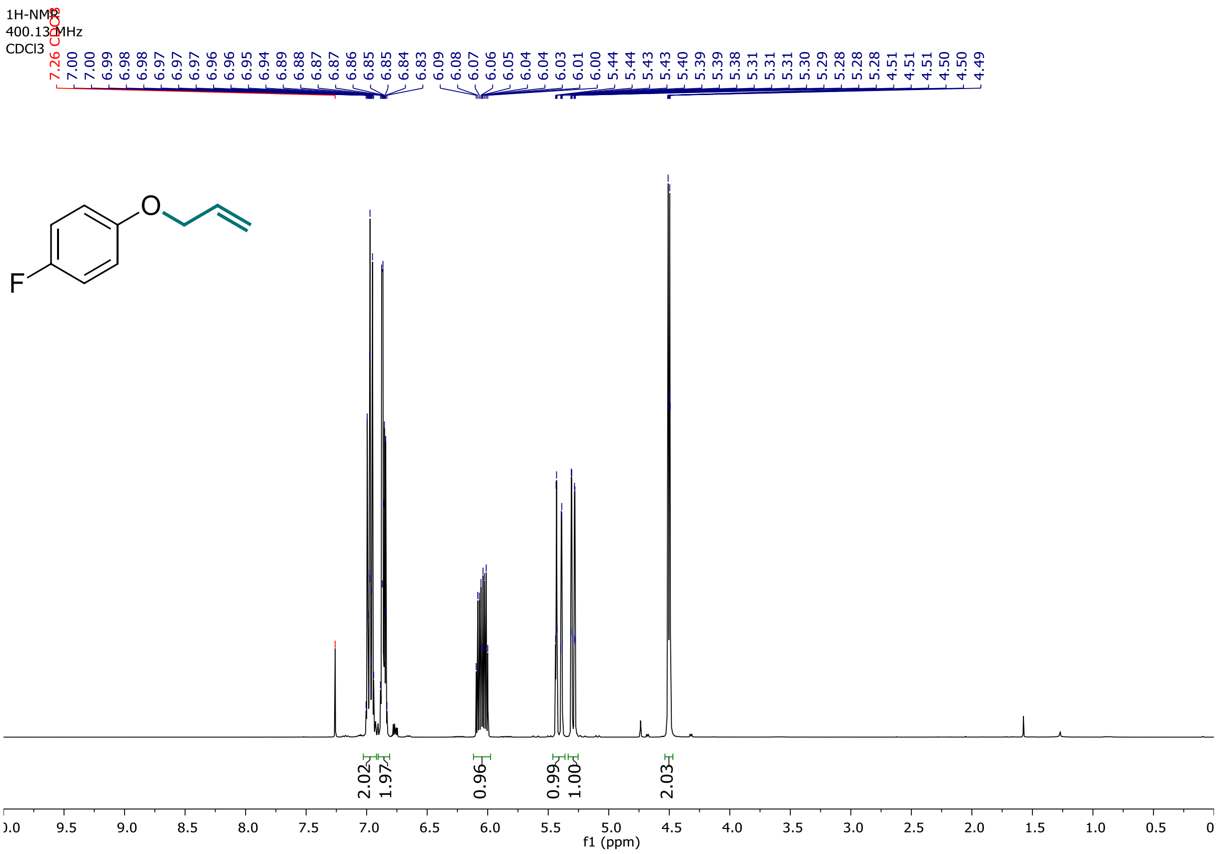

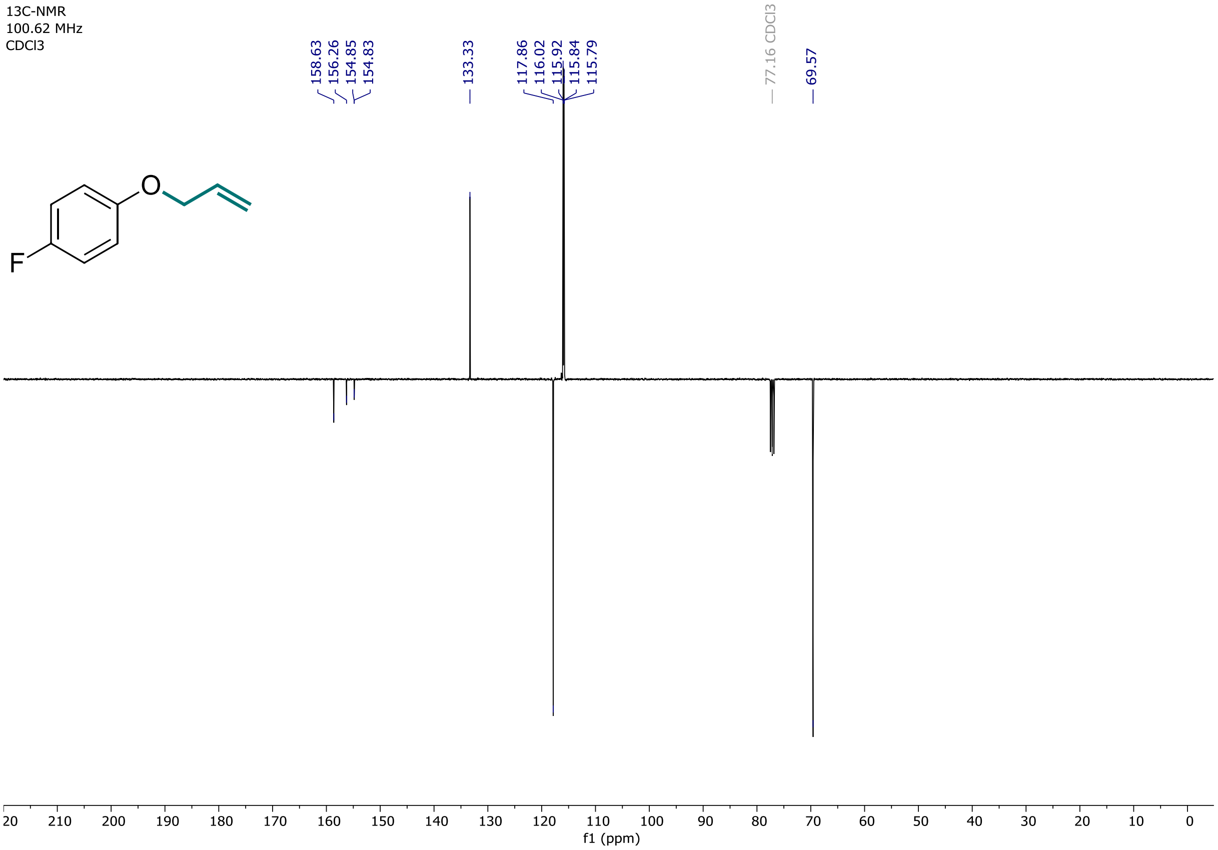

19F-NMR
376.46 MHz
CDCl3

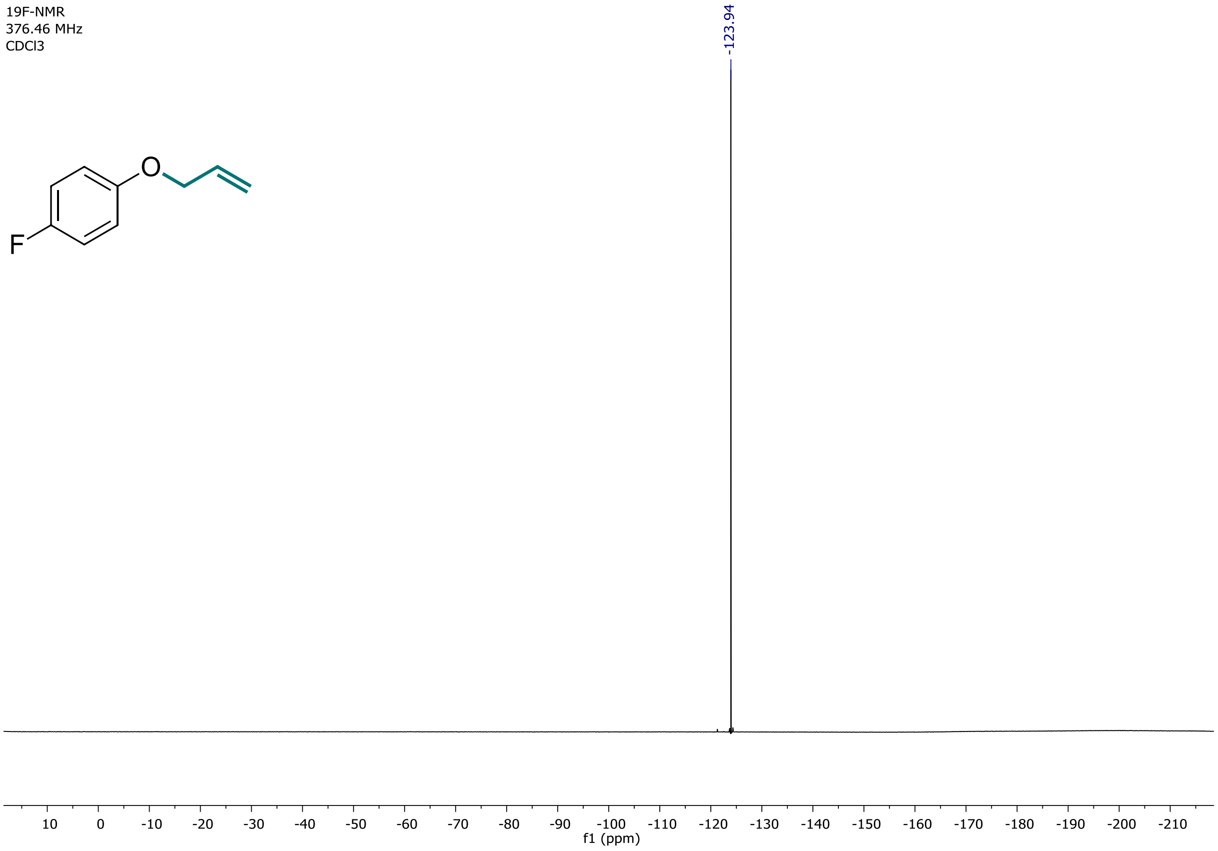

5-(allyloxy)-2-chloro-1,3-dimethylbenzene **(5)**

1H-NMR
400.13 MHz
CDCl3

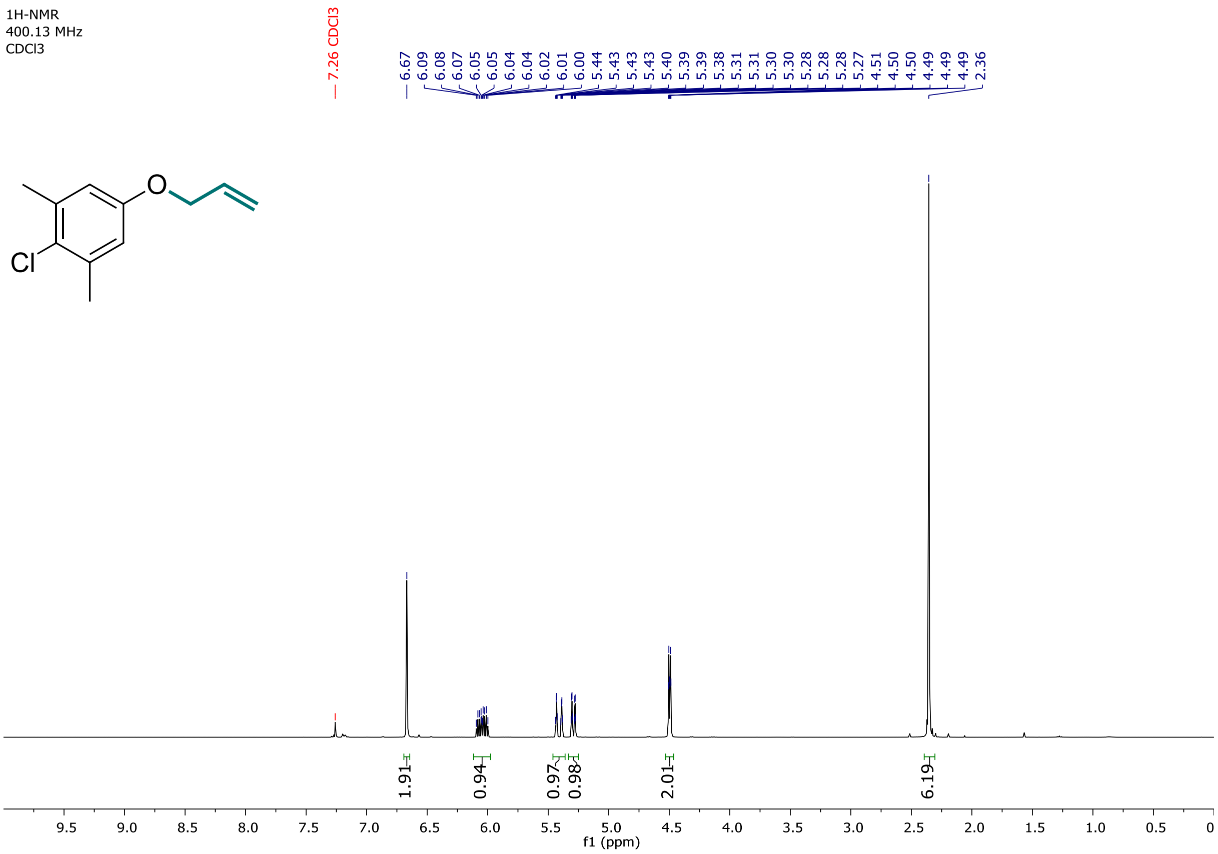

13C-NMR
100.62 MHz
CDCl3

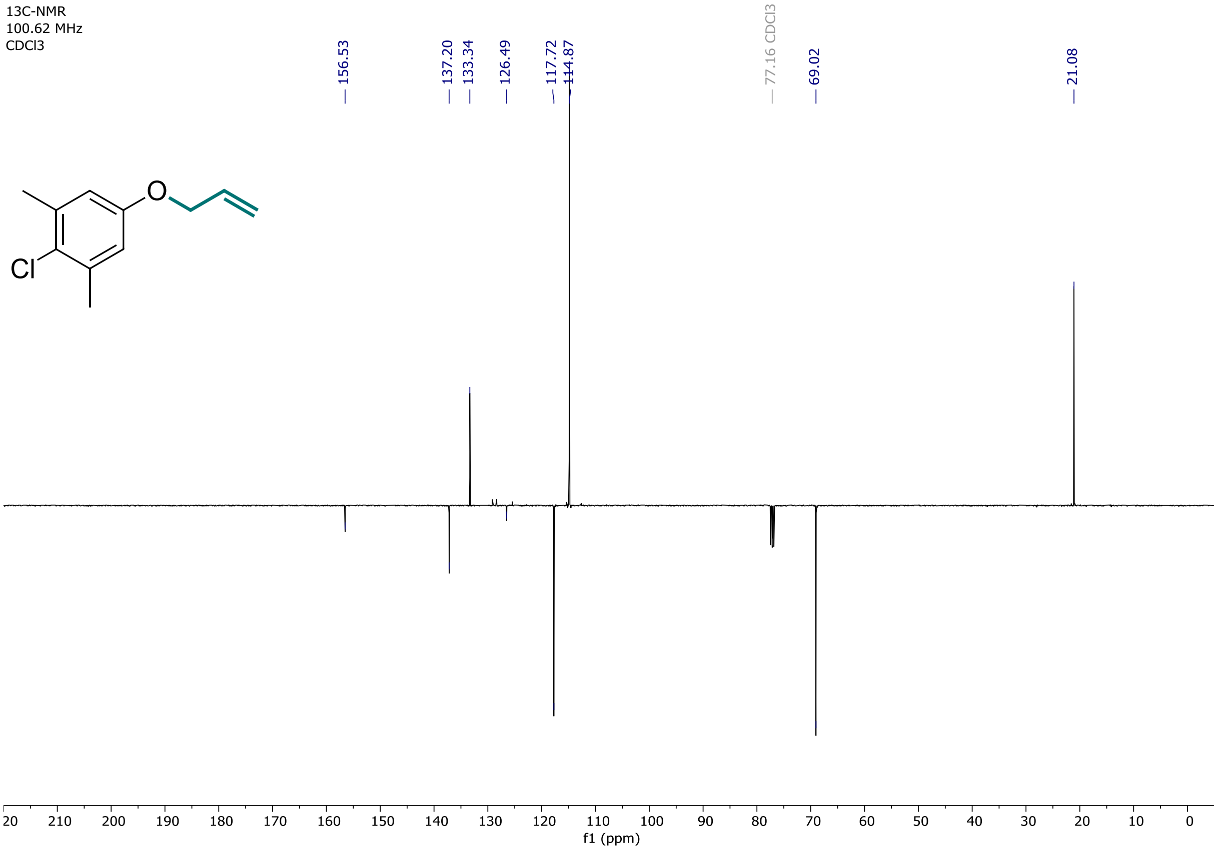

1-(Allyloxy)-2-bromobenzene **(6)**

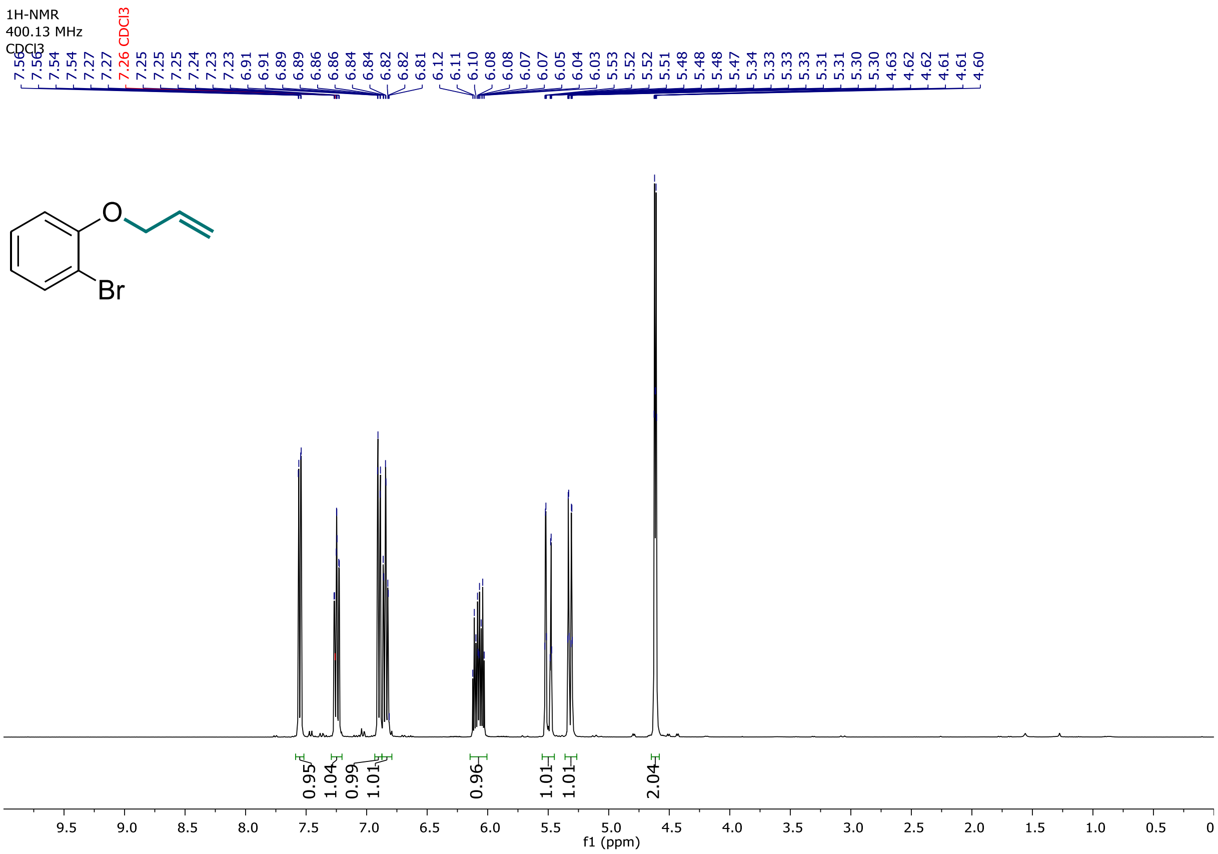

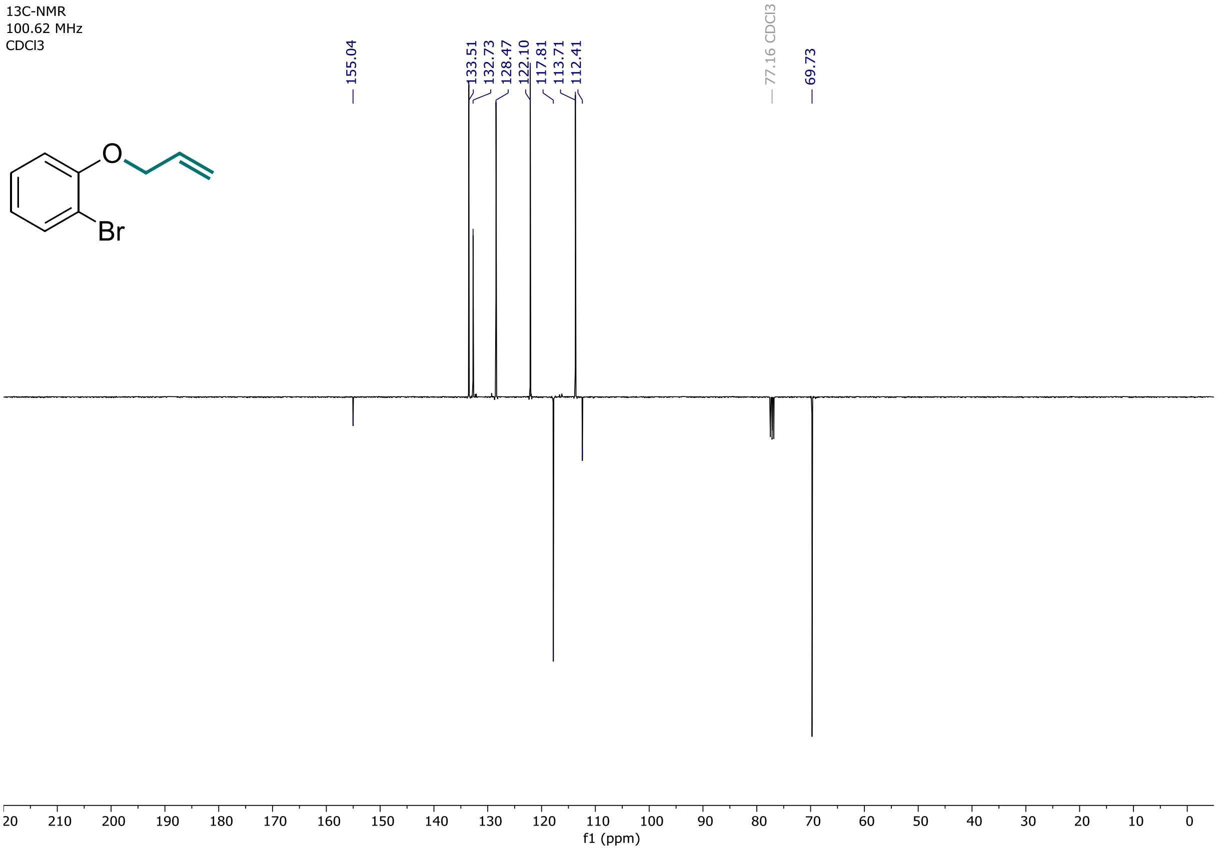

1-Bromo-2-methoxy-4-allyloxybenzene **(7)**

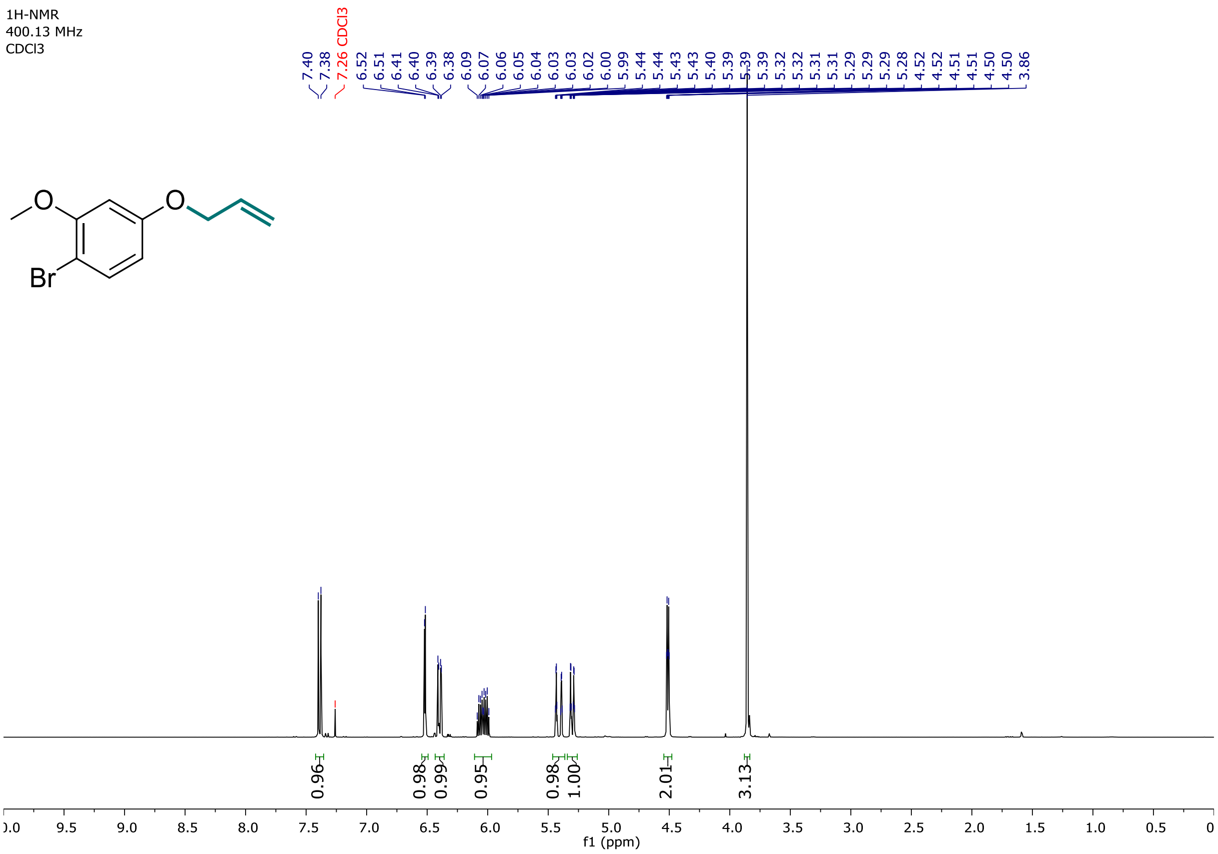

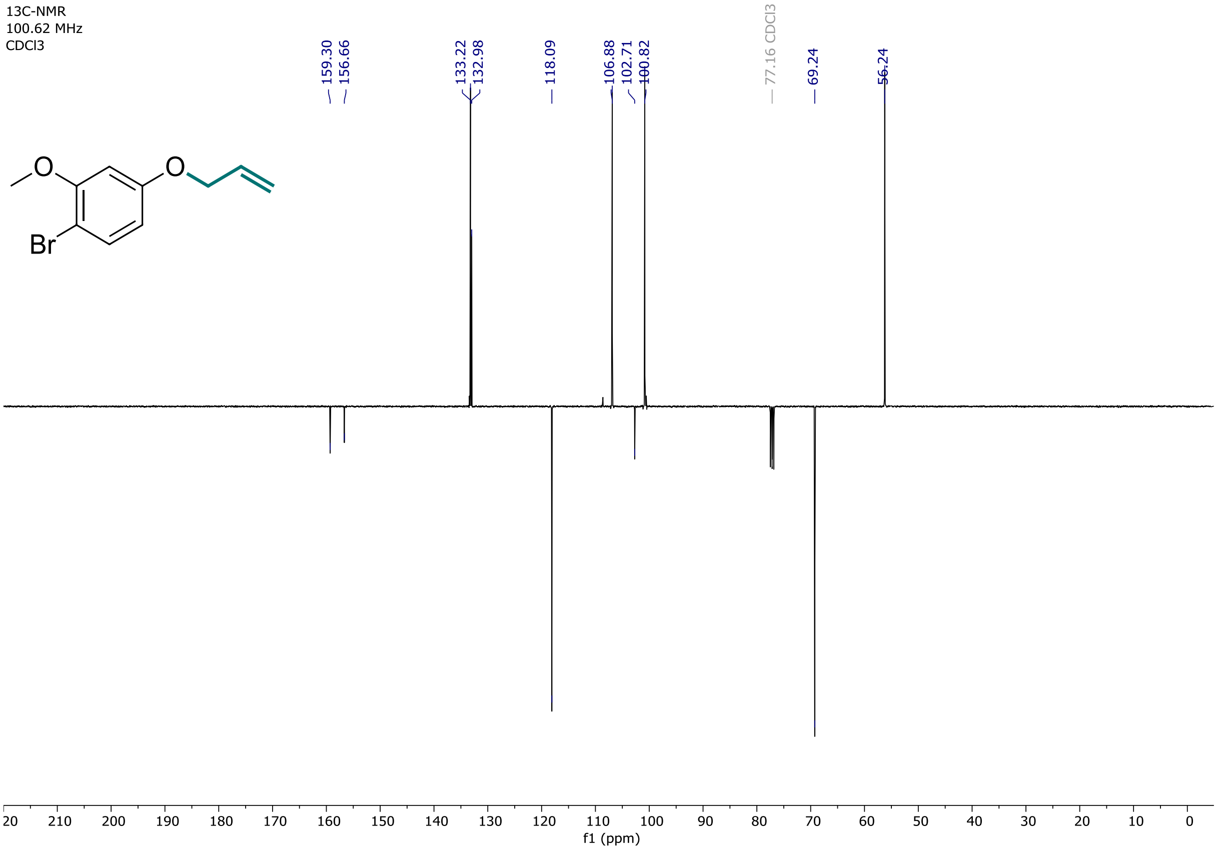

1-(Allyloxy)-4-methoxybenzene **(8)**

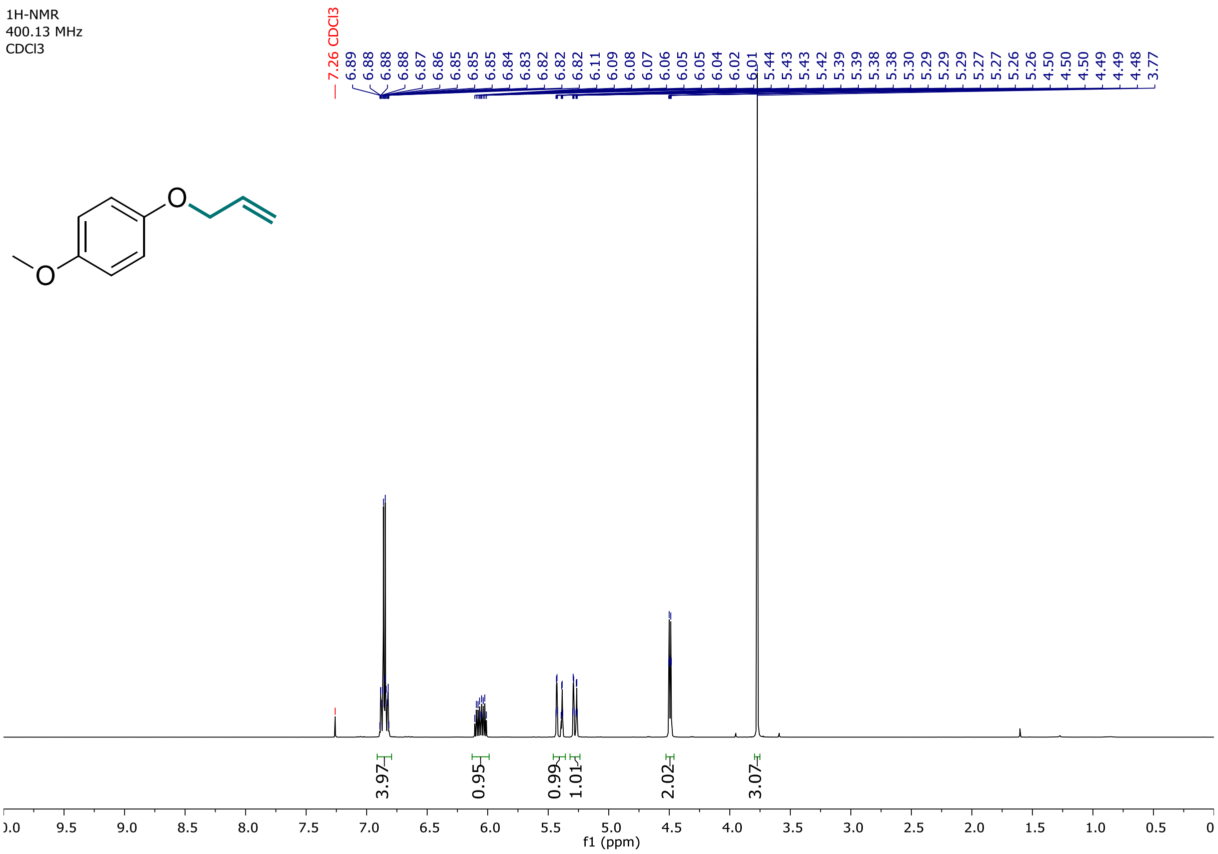

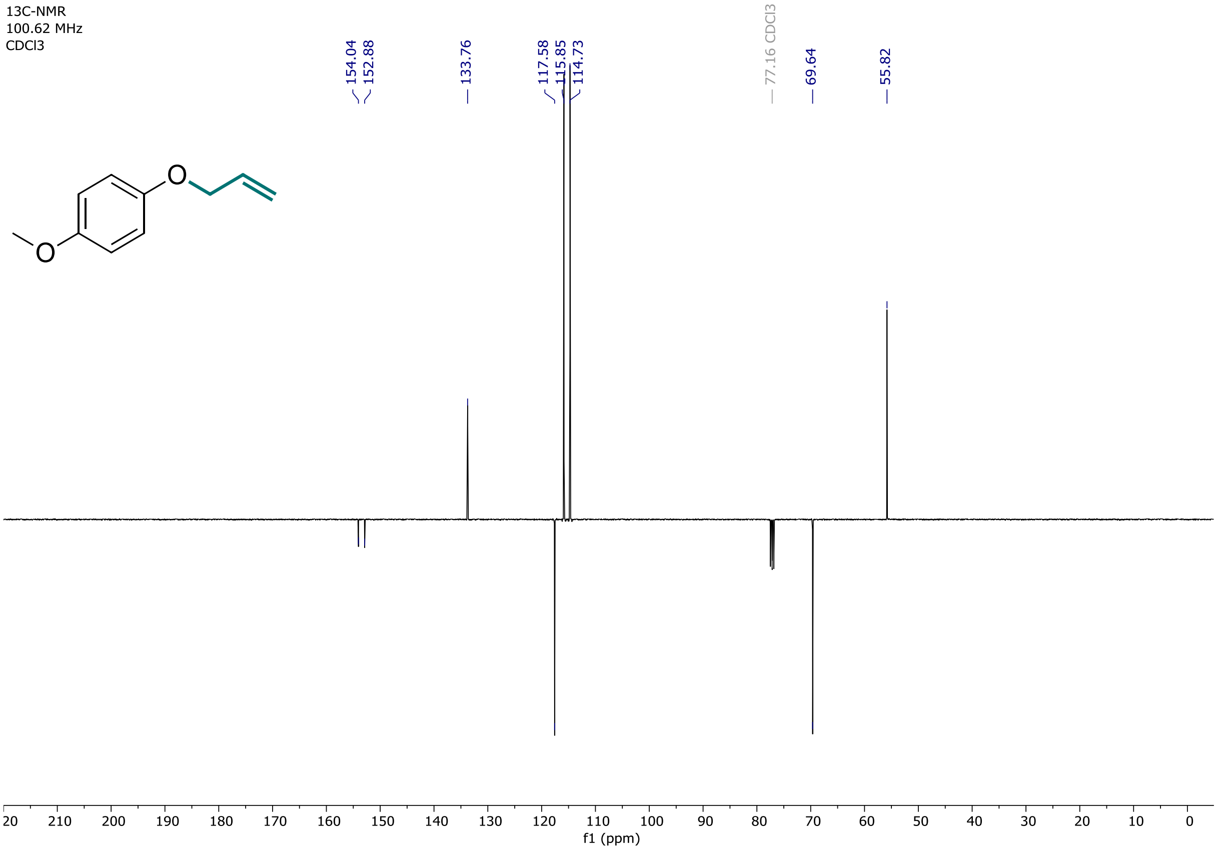

4-Allyl-1-(allyloxy)-2-methoxybenzene **(9)**

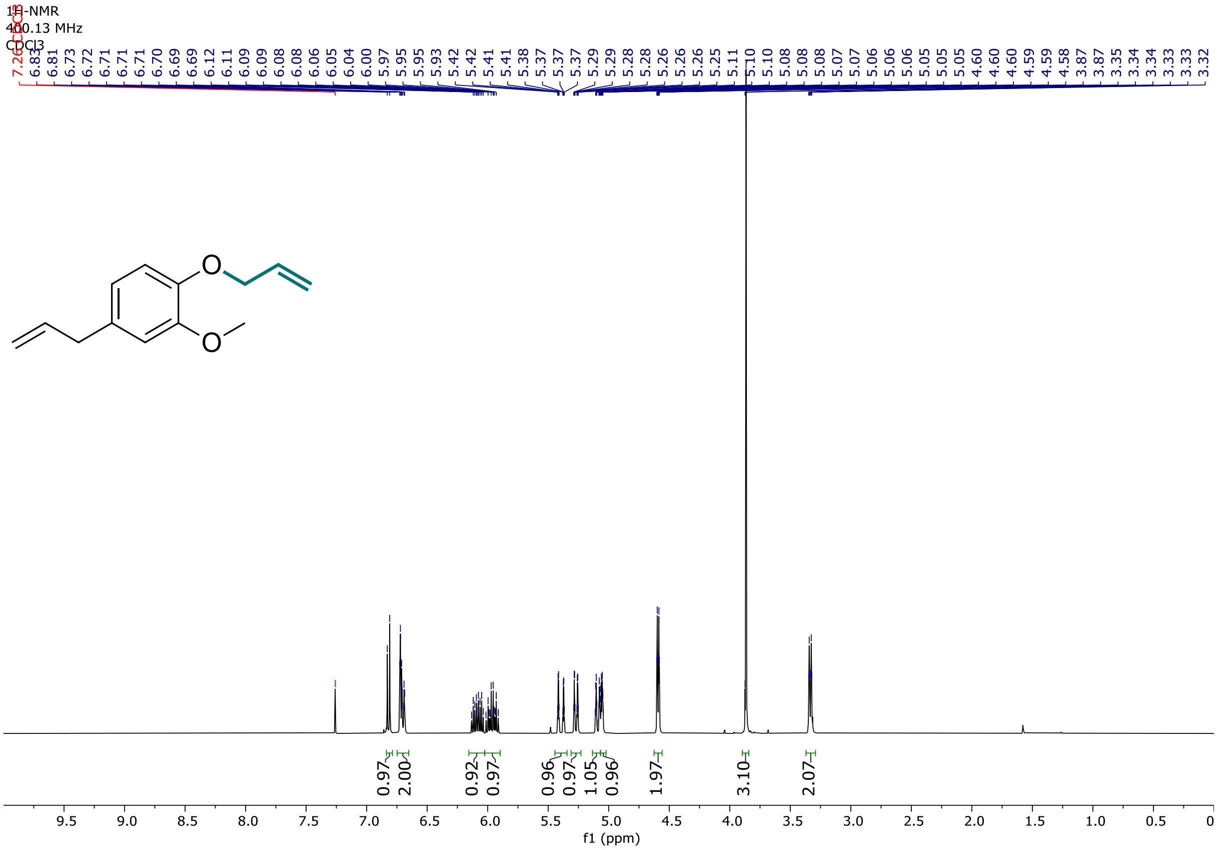

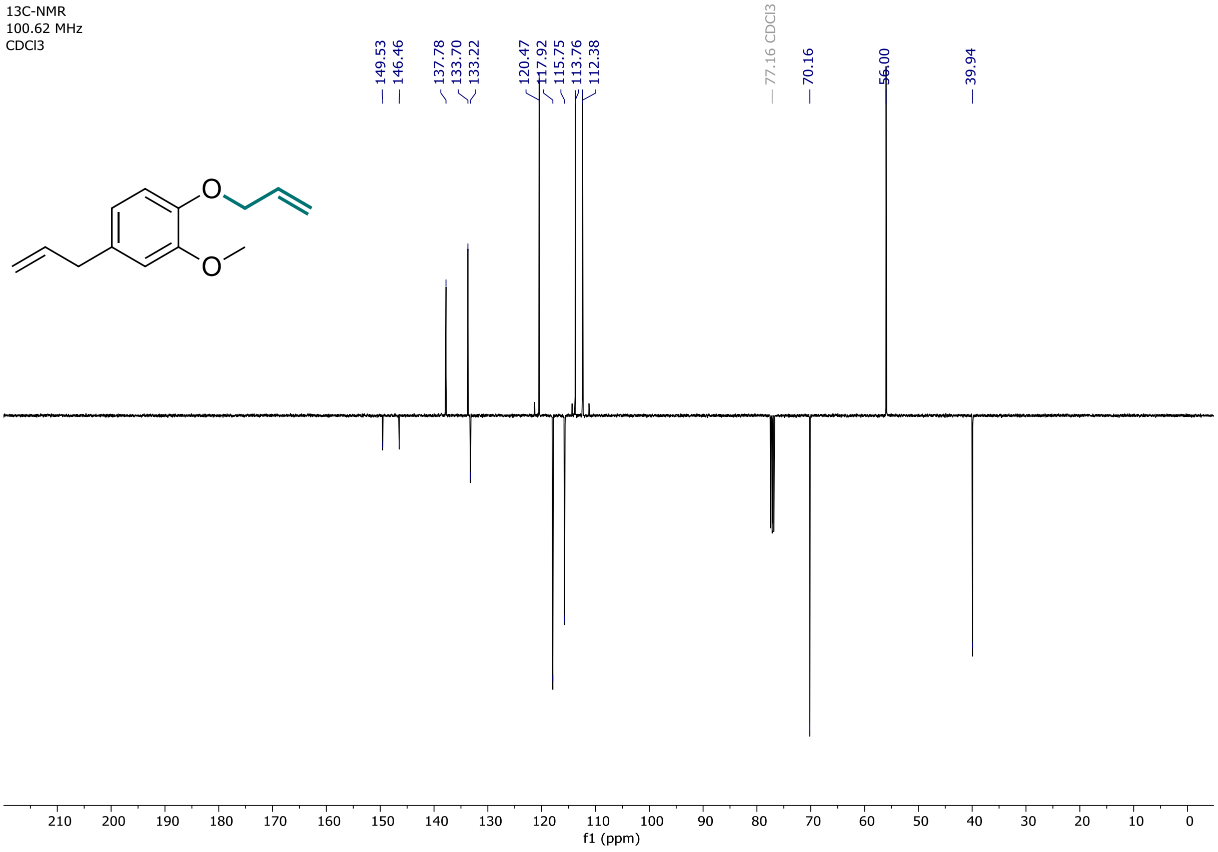

1-(Allyloxy)-2-methoxybenzene **(10)**

1H-NMR
400.13 MHz
CDCl3

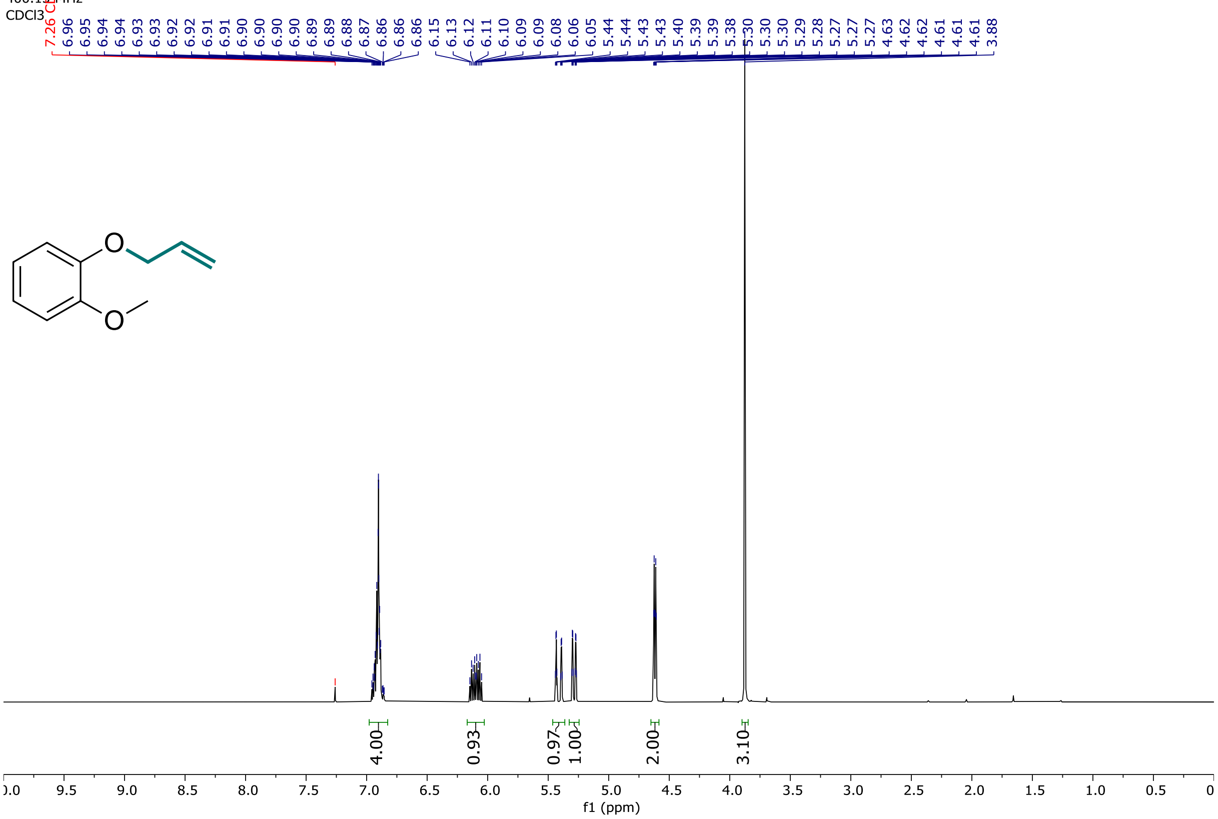

13C-NMR
100.62 MHz
CDCl3

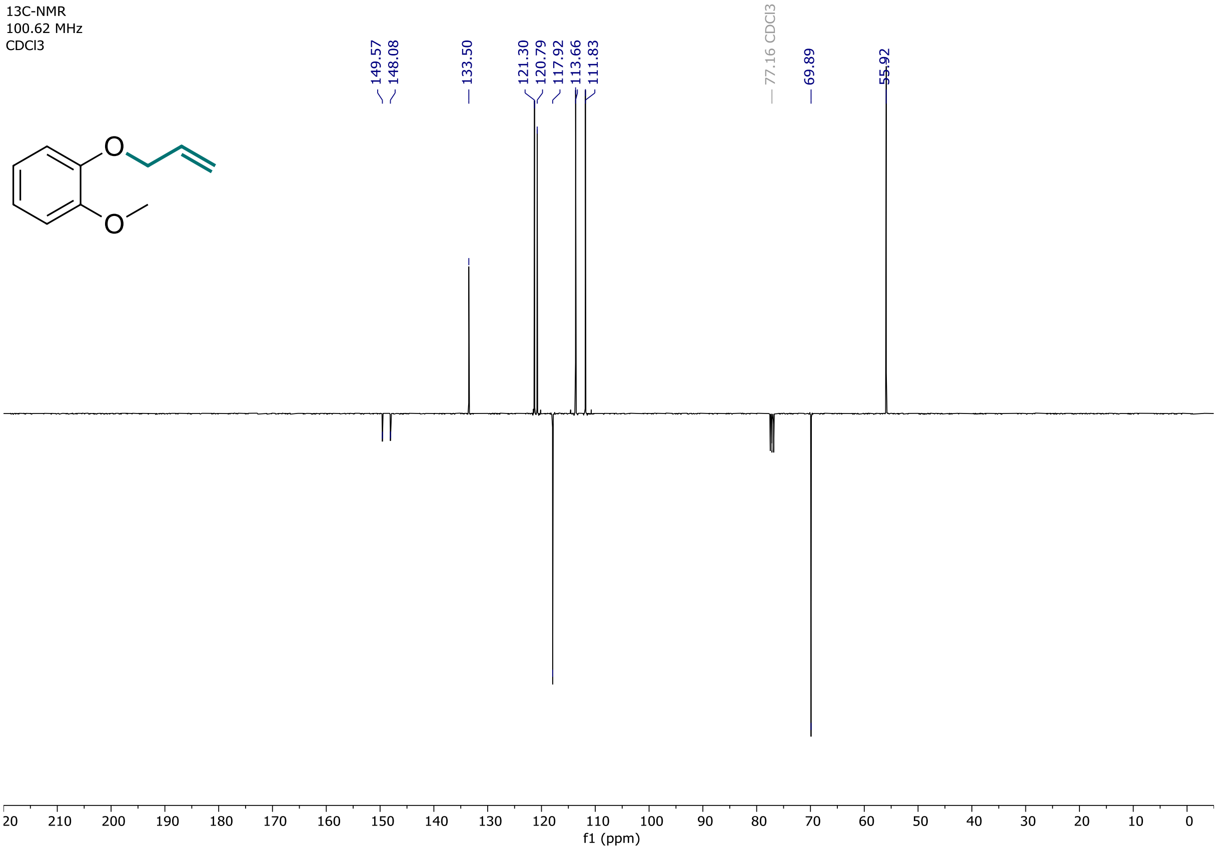

O-Allyl-sesamol **(11)**

1H-NMR
400.13 MHz
CDCl3

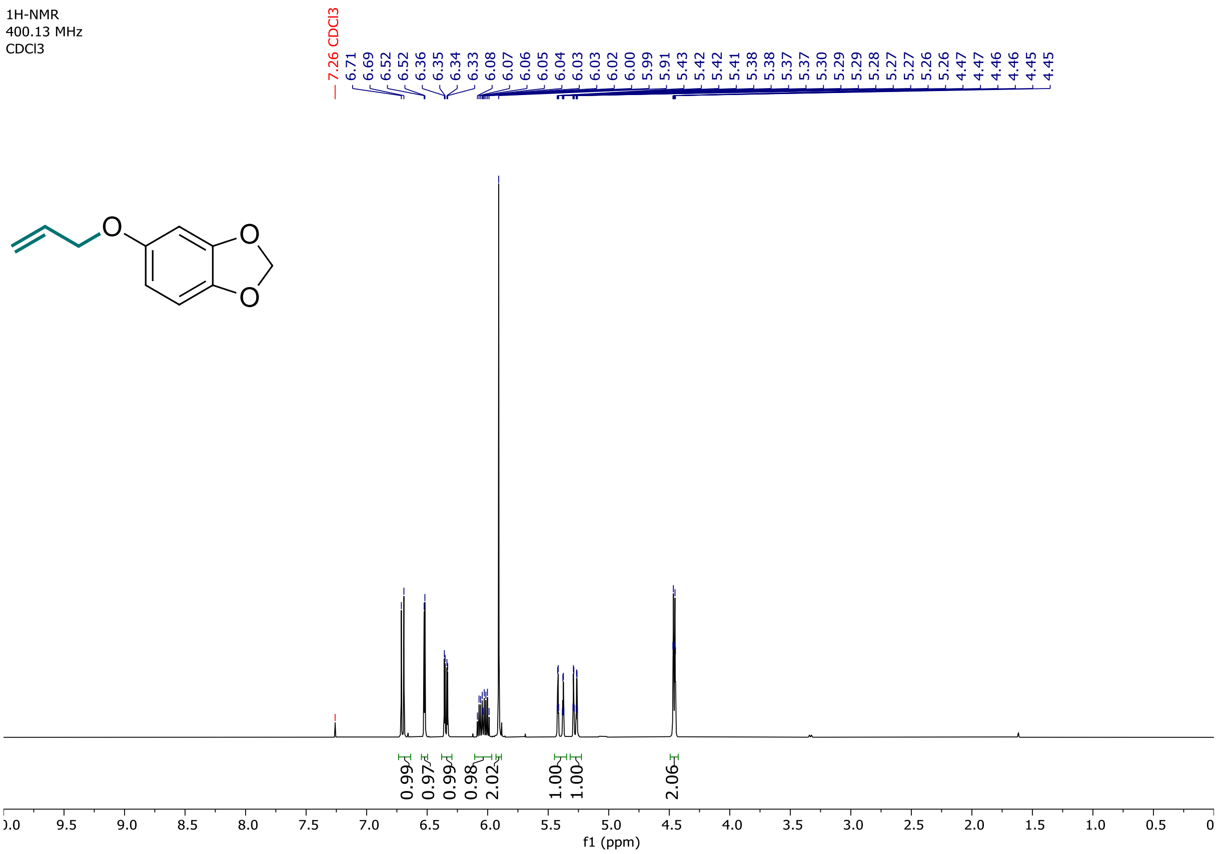

13C-NMR
100.62 MHz
CDCl3

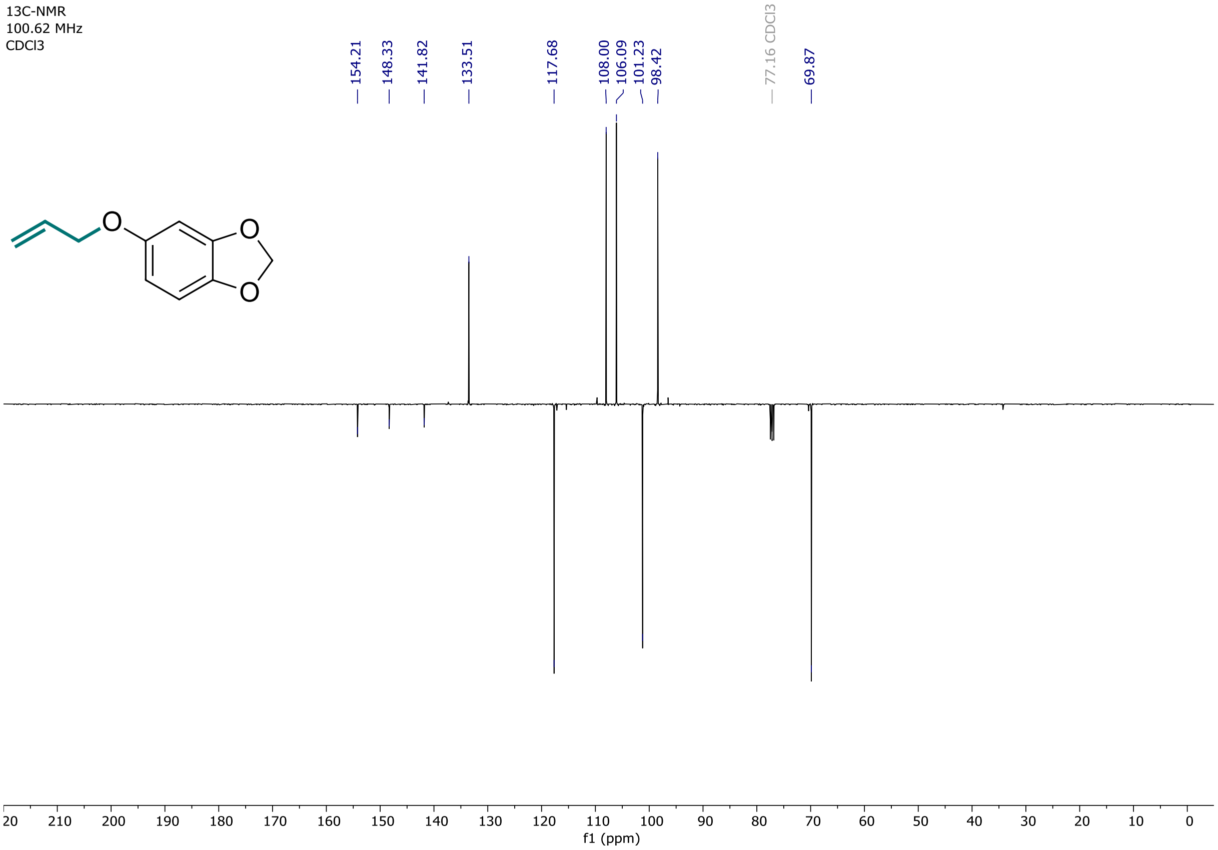

1-Allyloxy-3-nitrobenzene **(12)**

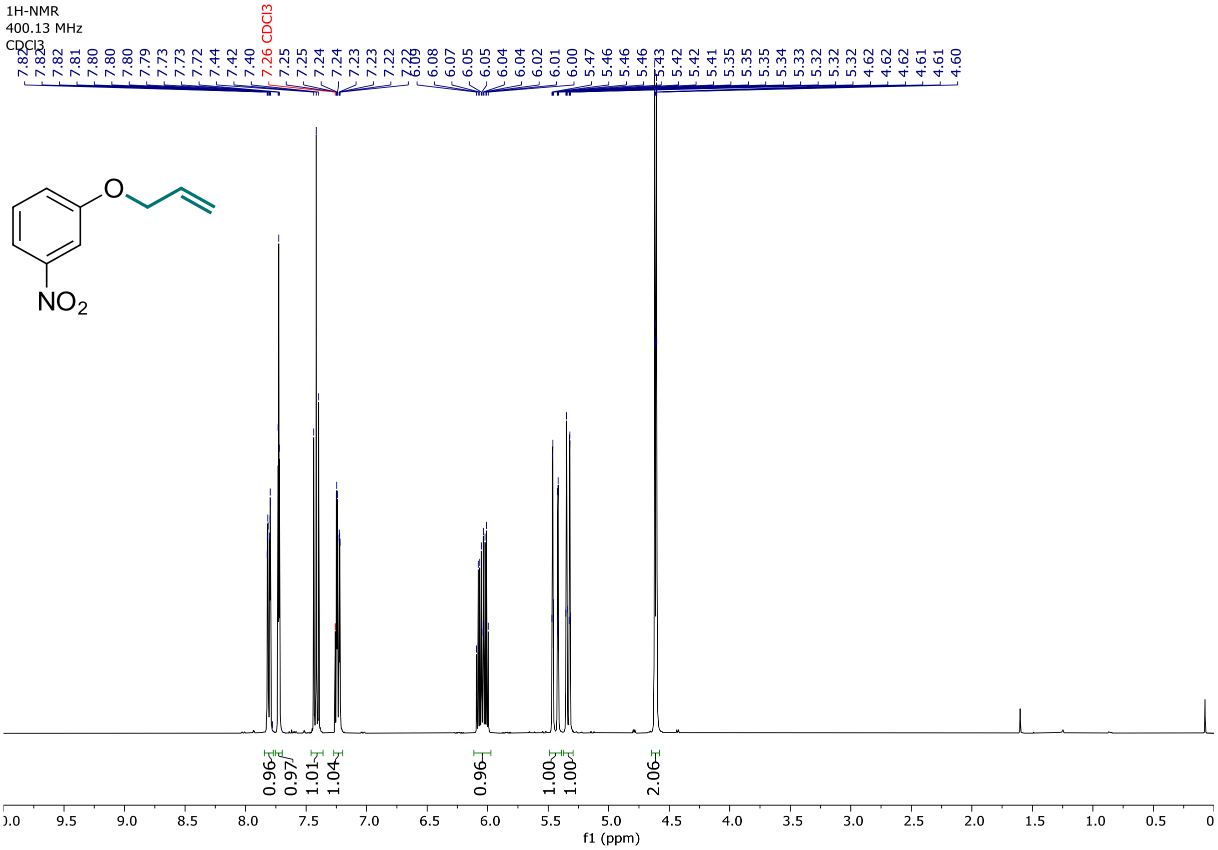

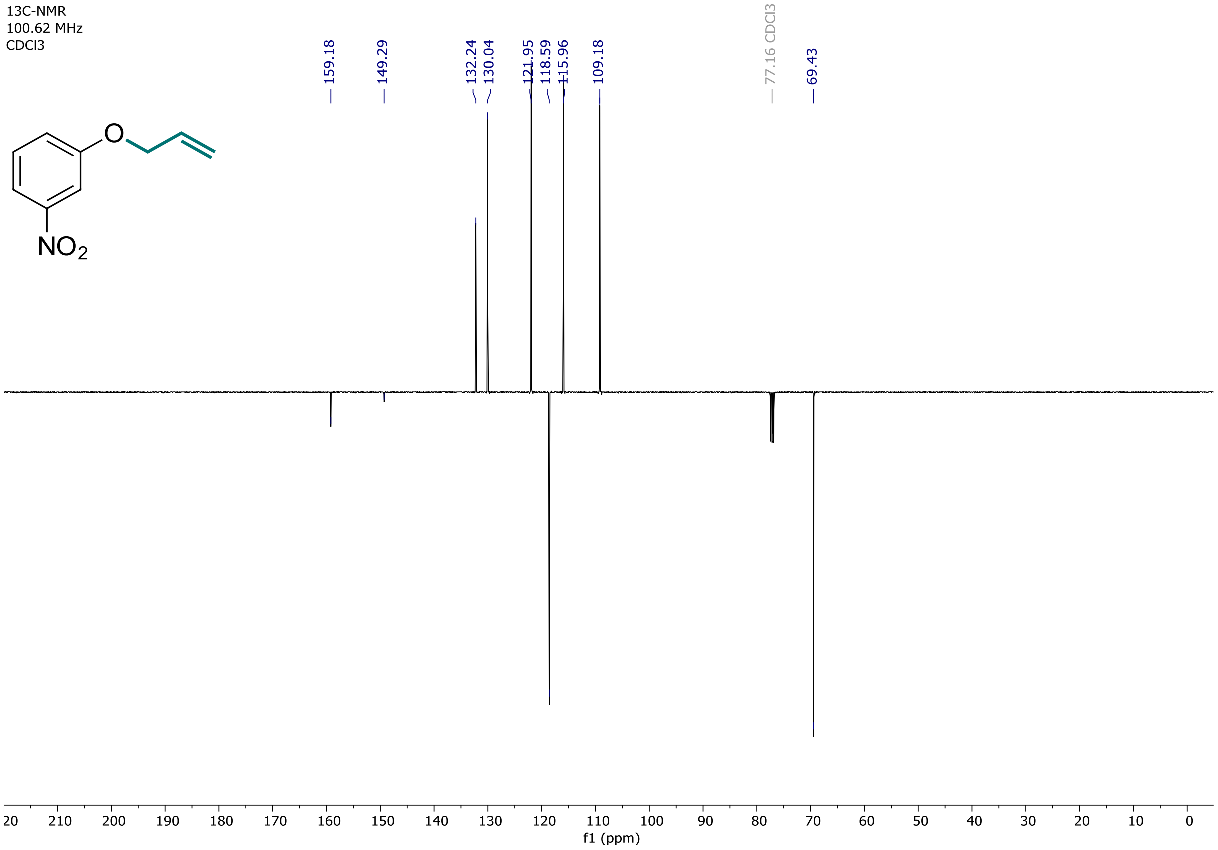

2-(Allyloxy)benzaldehyde **(13)**

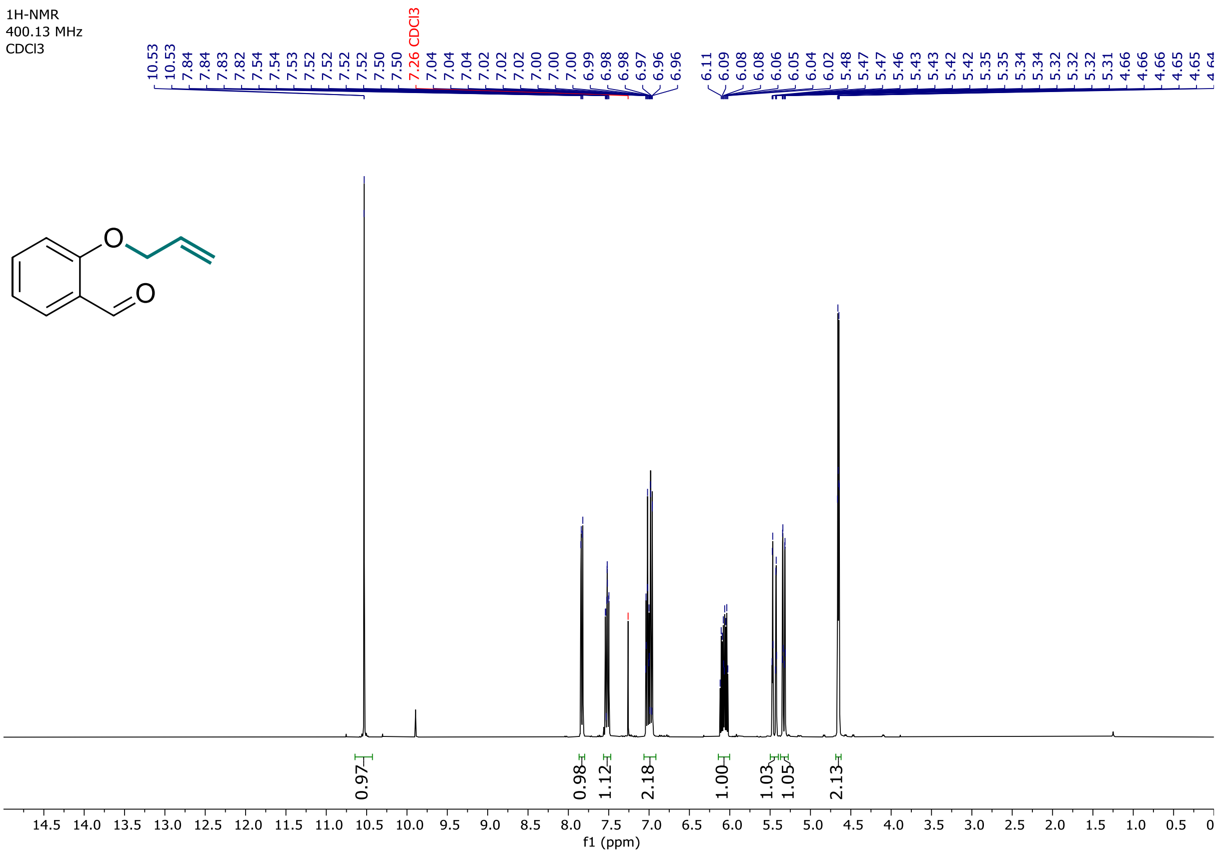

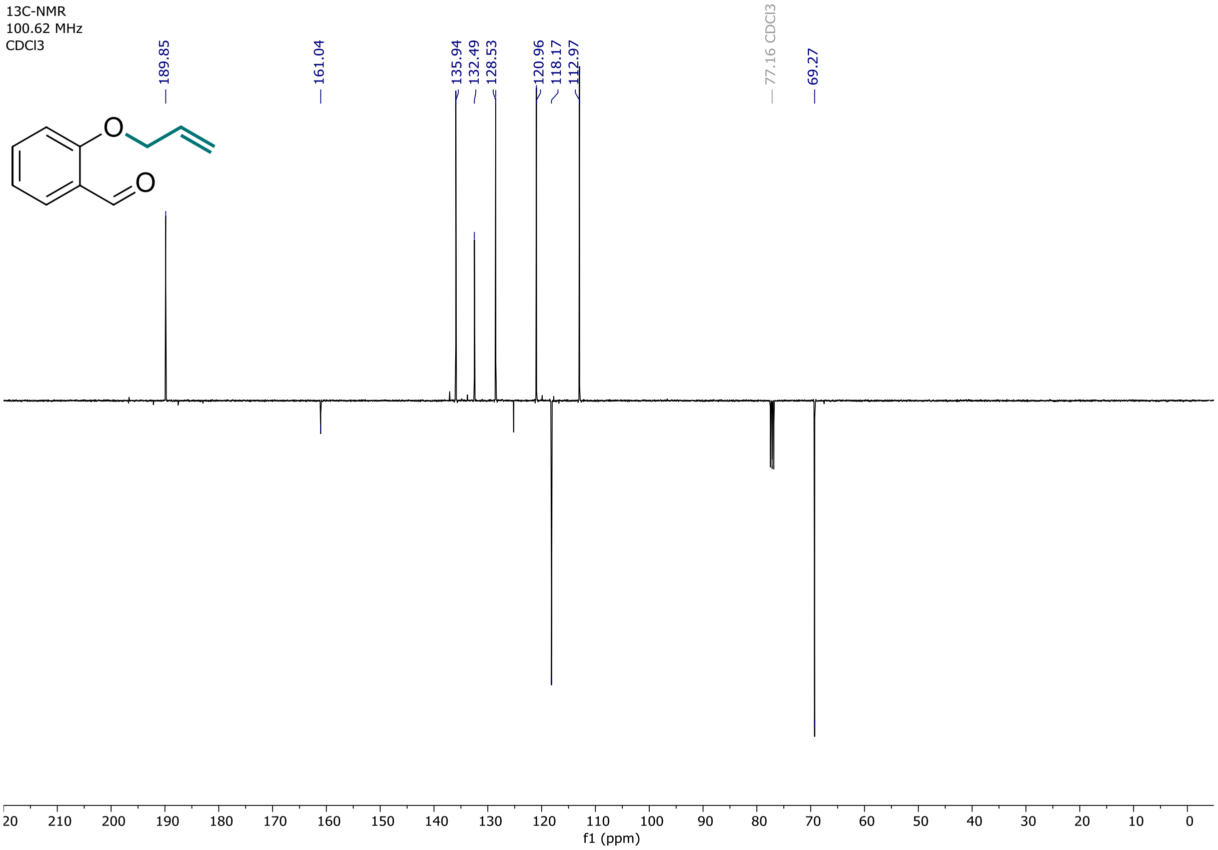

Allyl furfuryl ether **(14)**

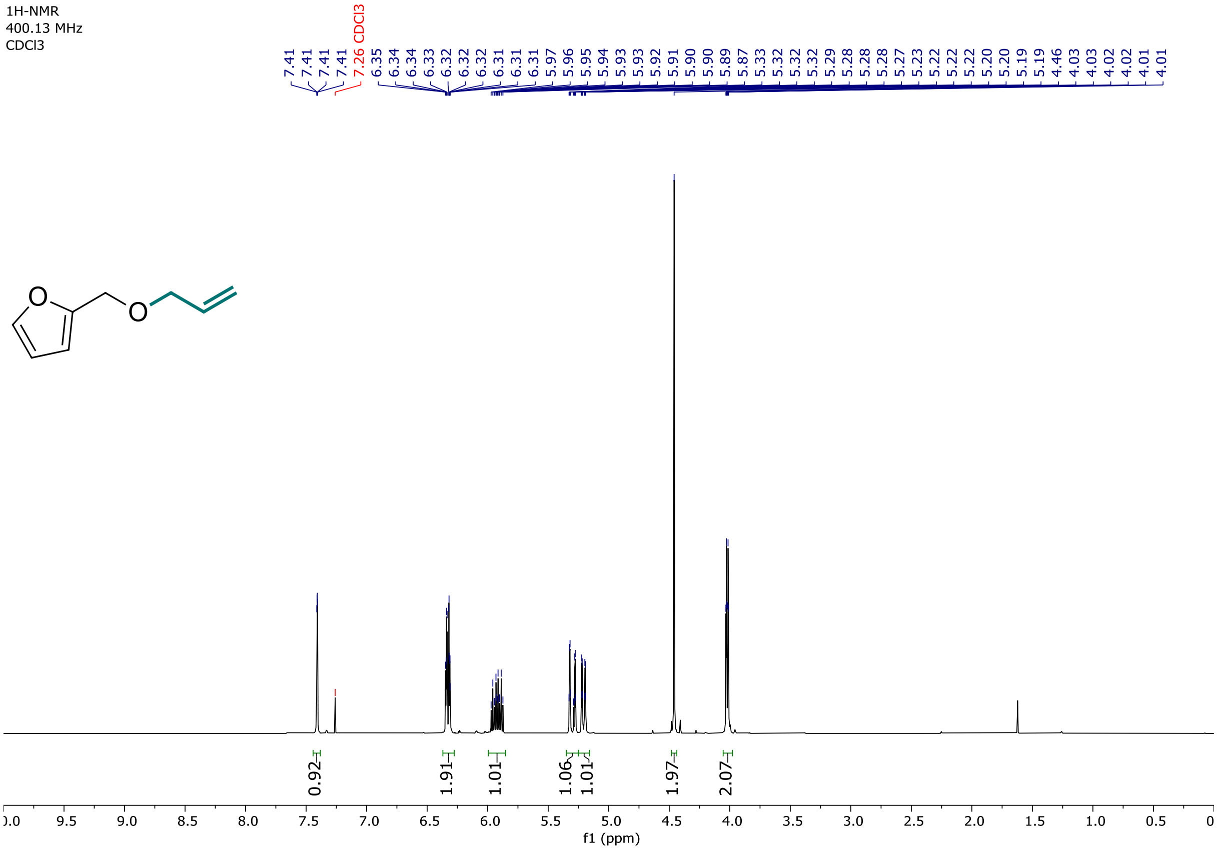

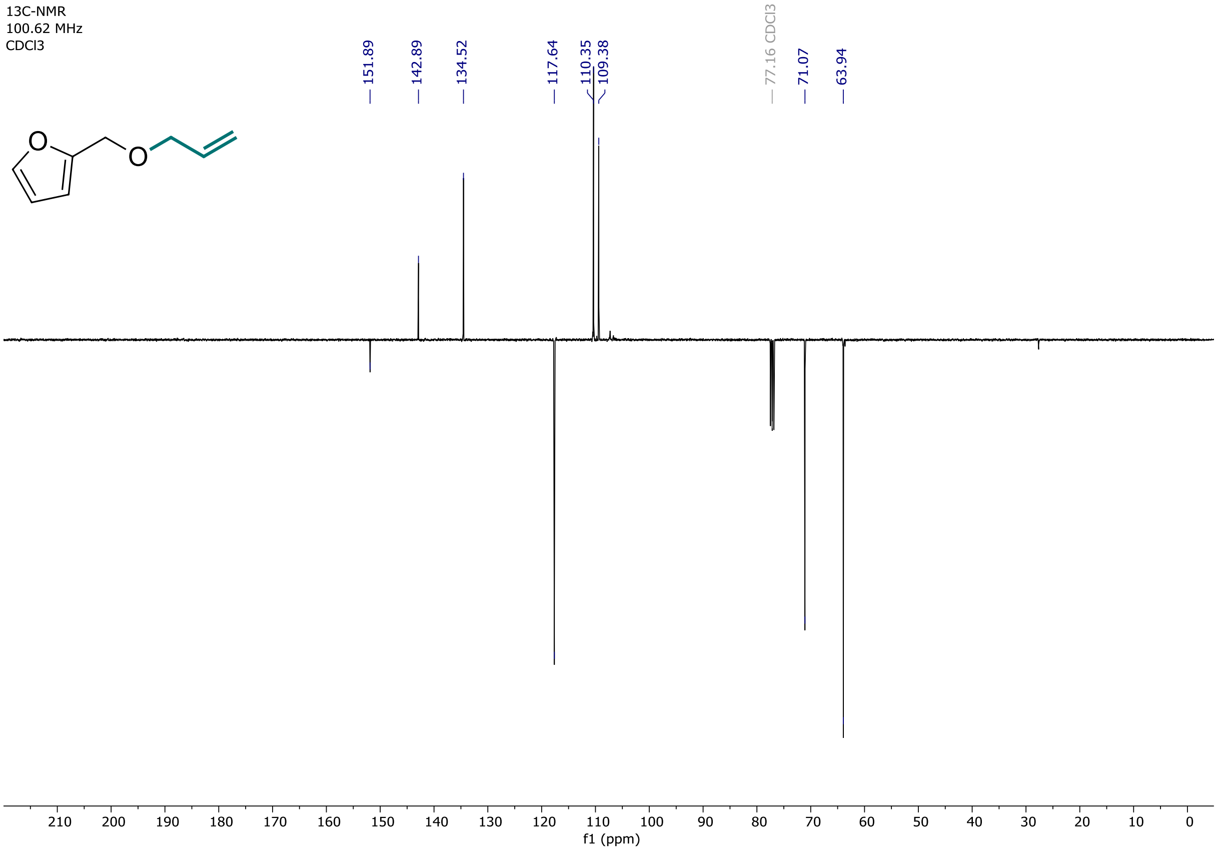

Benzyl allyl ether **(15)**

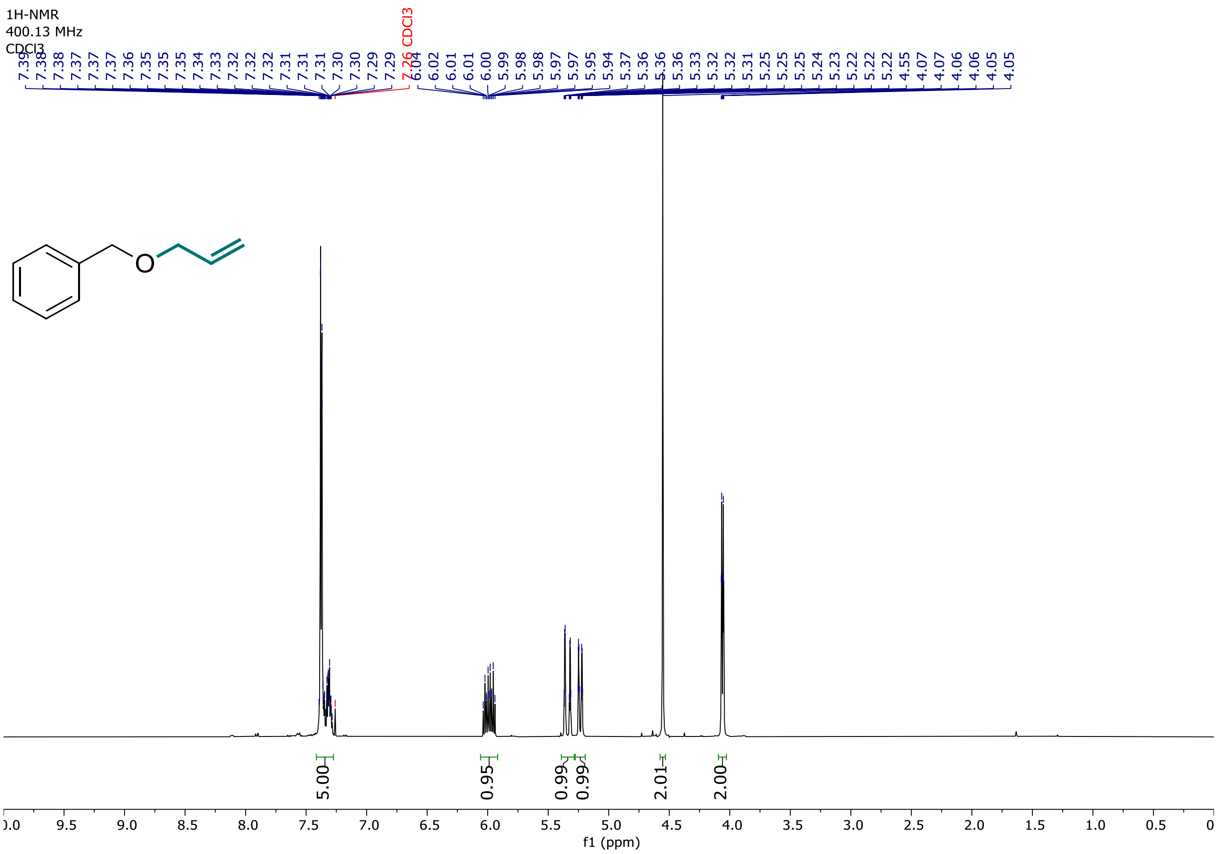

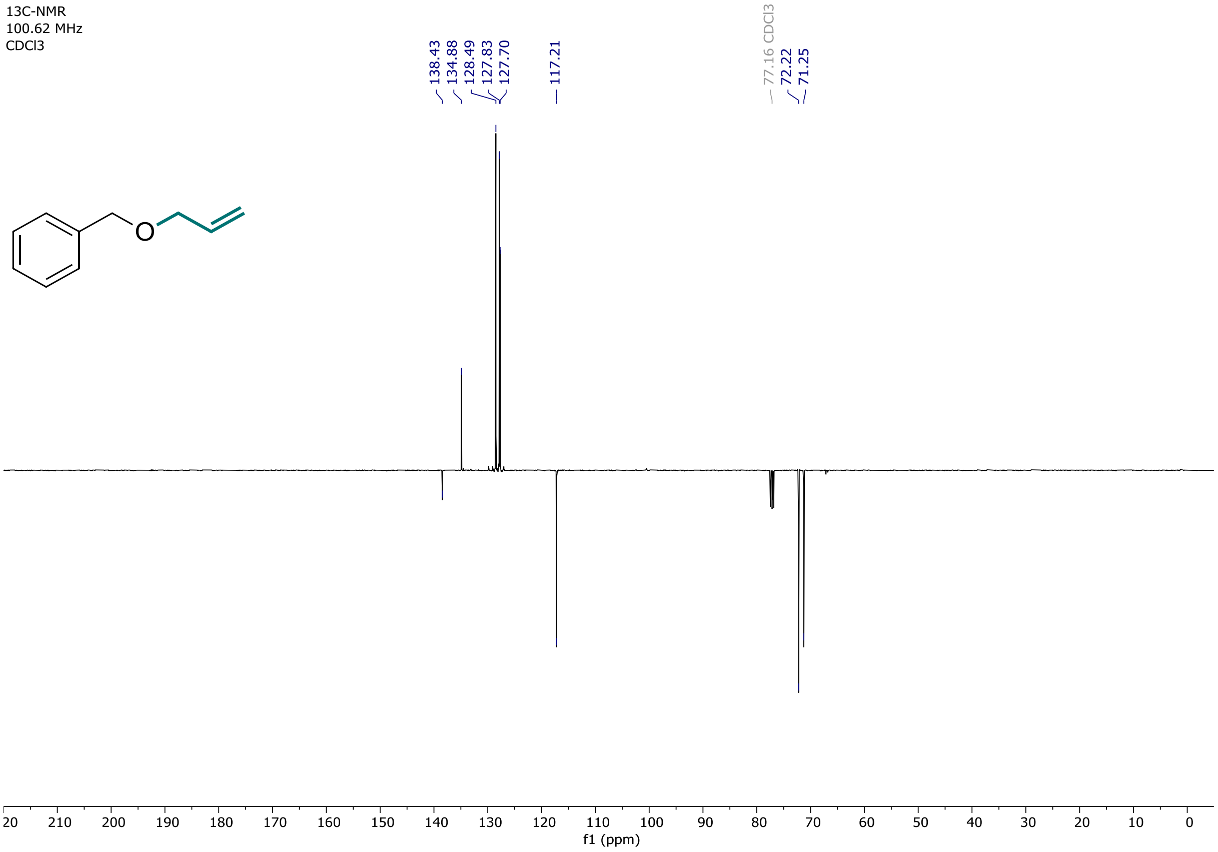

2-(2-propenyloxy)ethylbenzene **(16)**

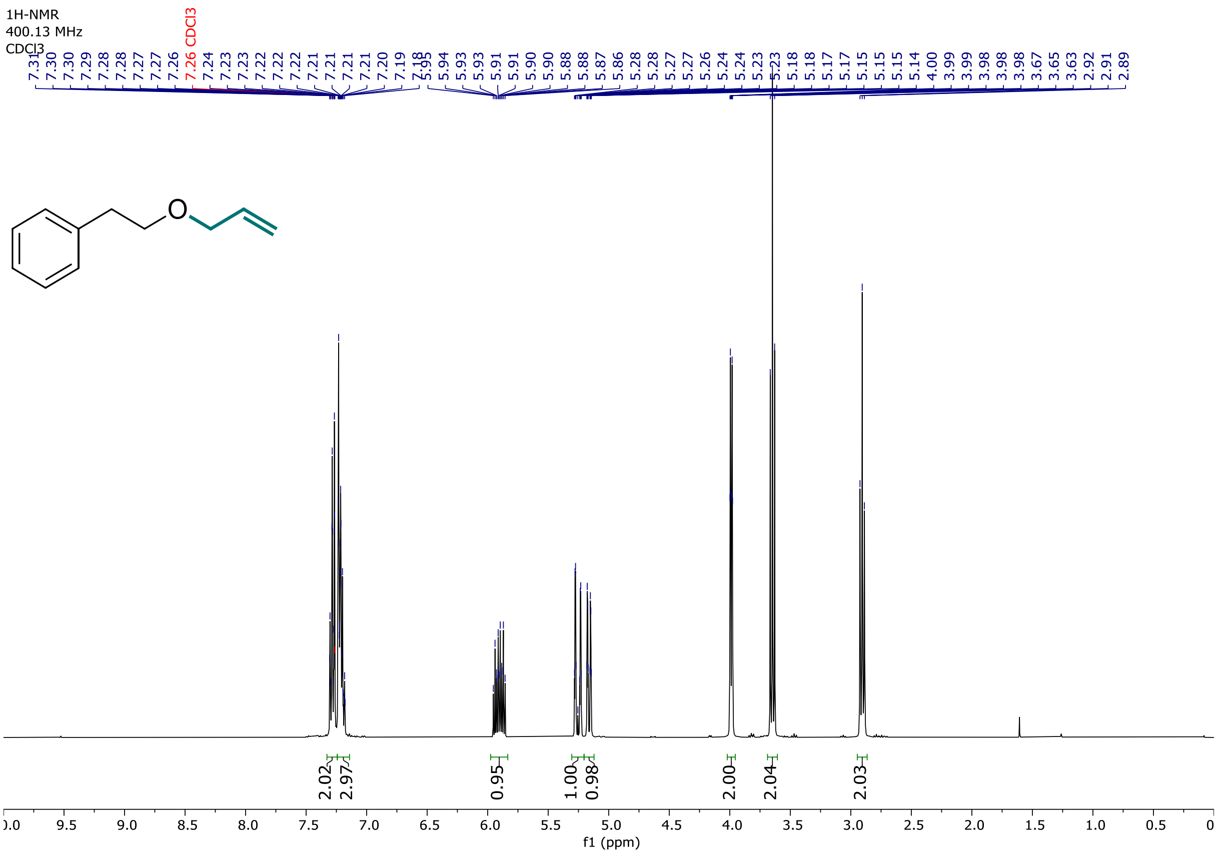

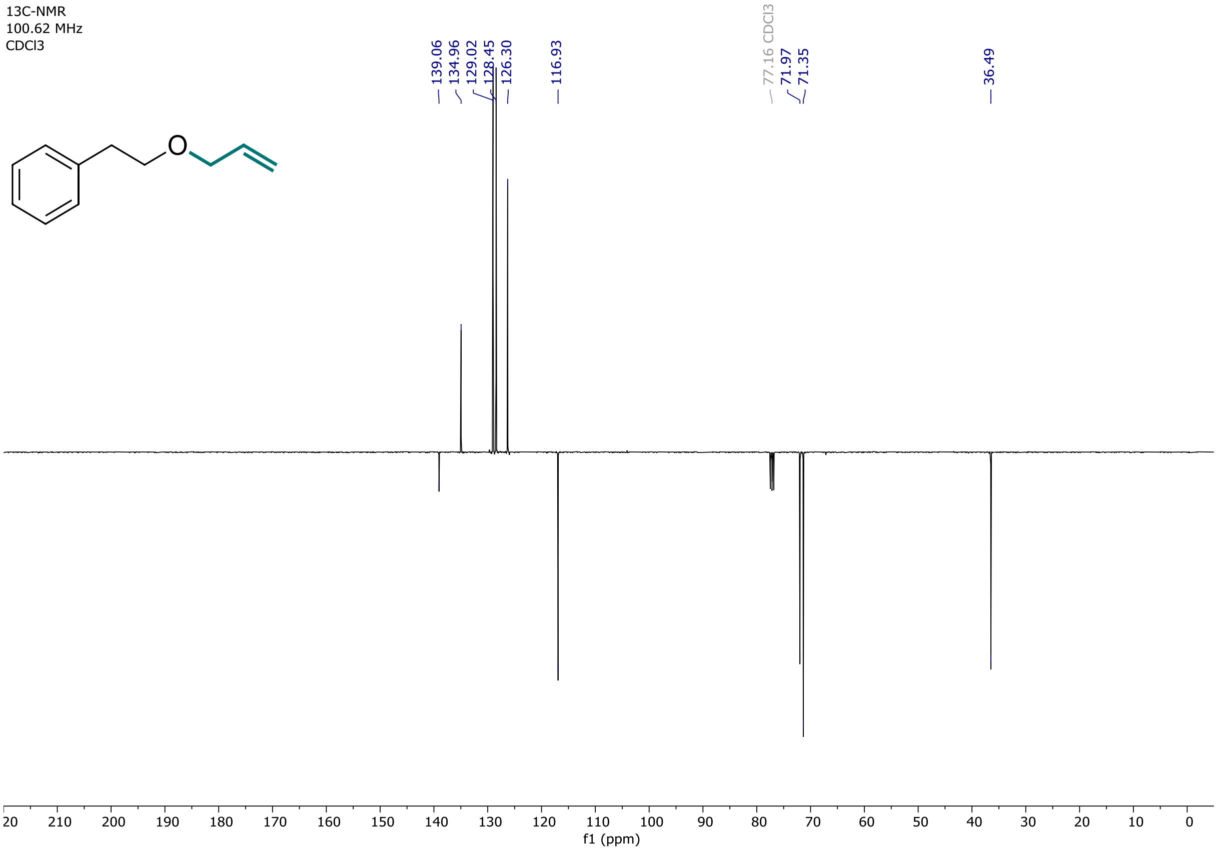

2-[4-(Allyloxy)-3-methoxyphenyl]-1,3-dioxolane (**17**)

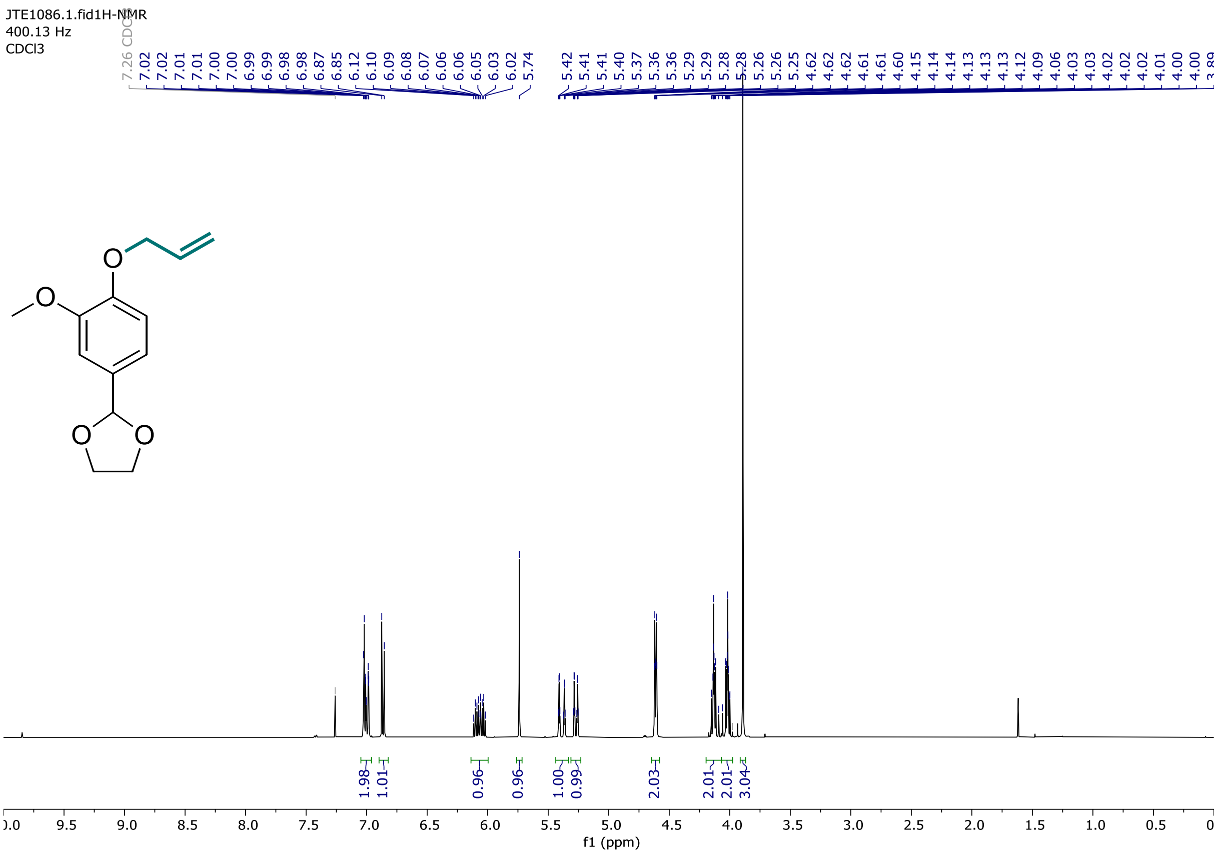

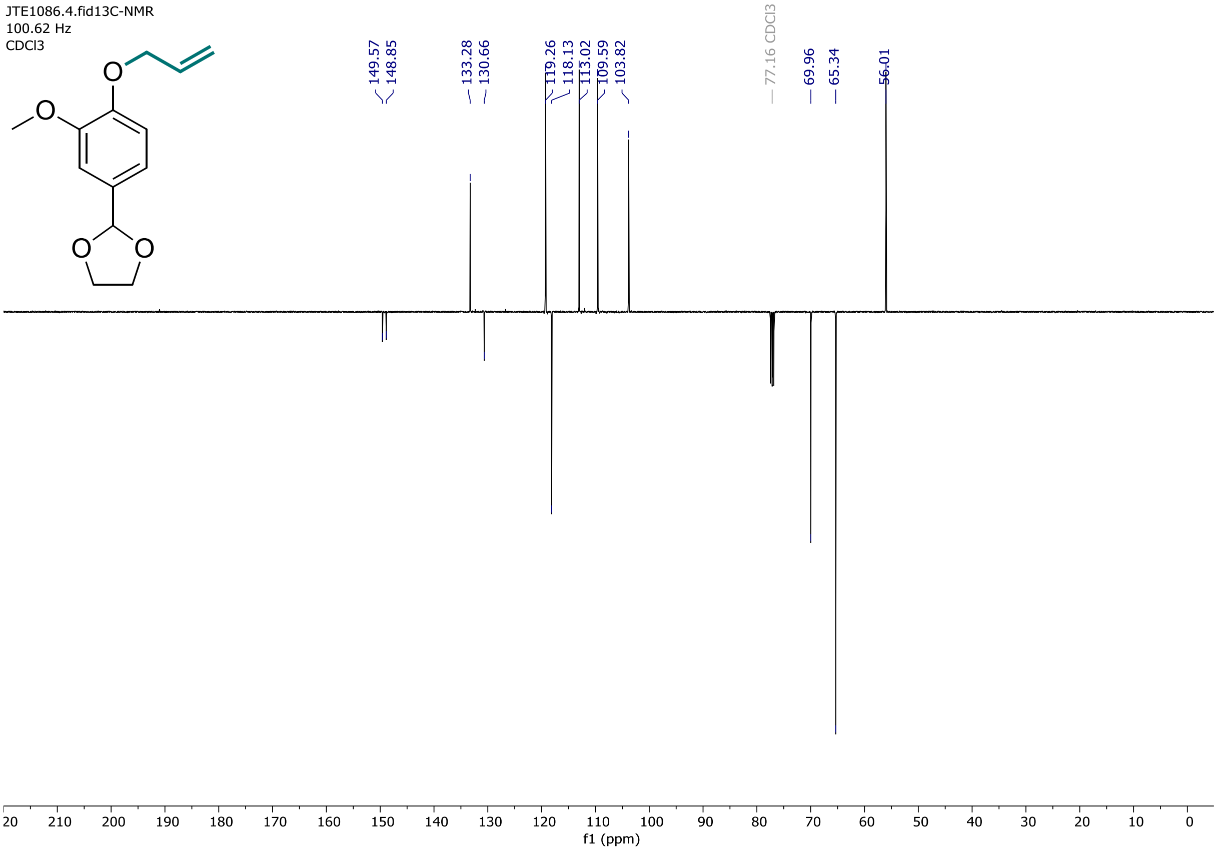

2-(4-(allyloxy)phenyl)pyridine **(18)**

1H-NMR
400.13 MHz
CDCl3

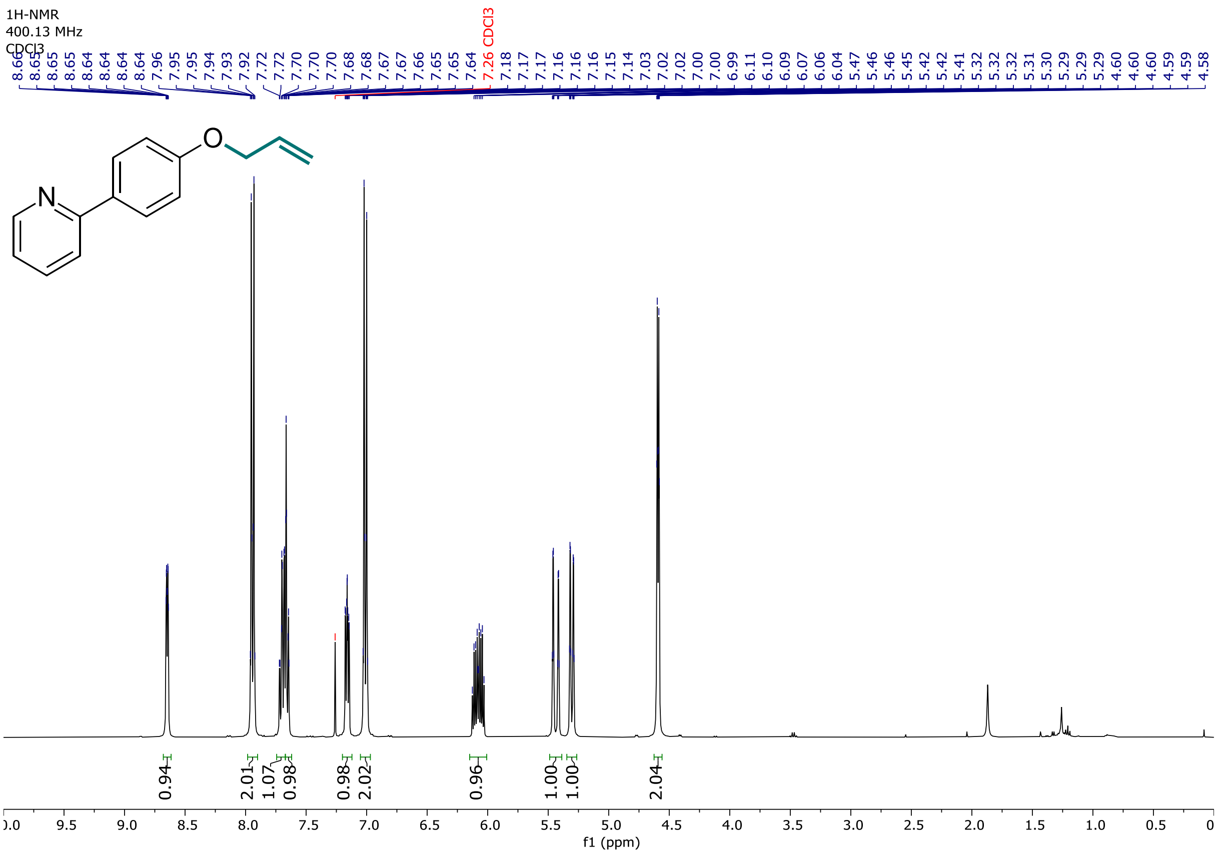

13C-NMR
100.62 MHz
CDCl3

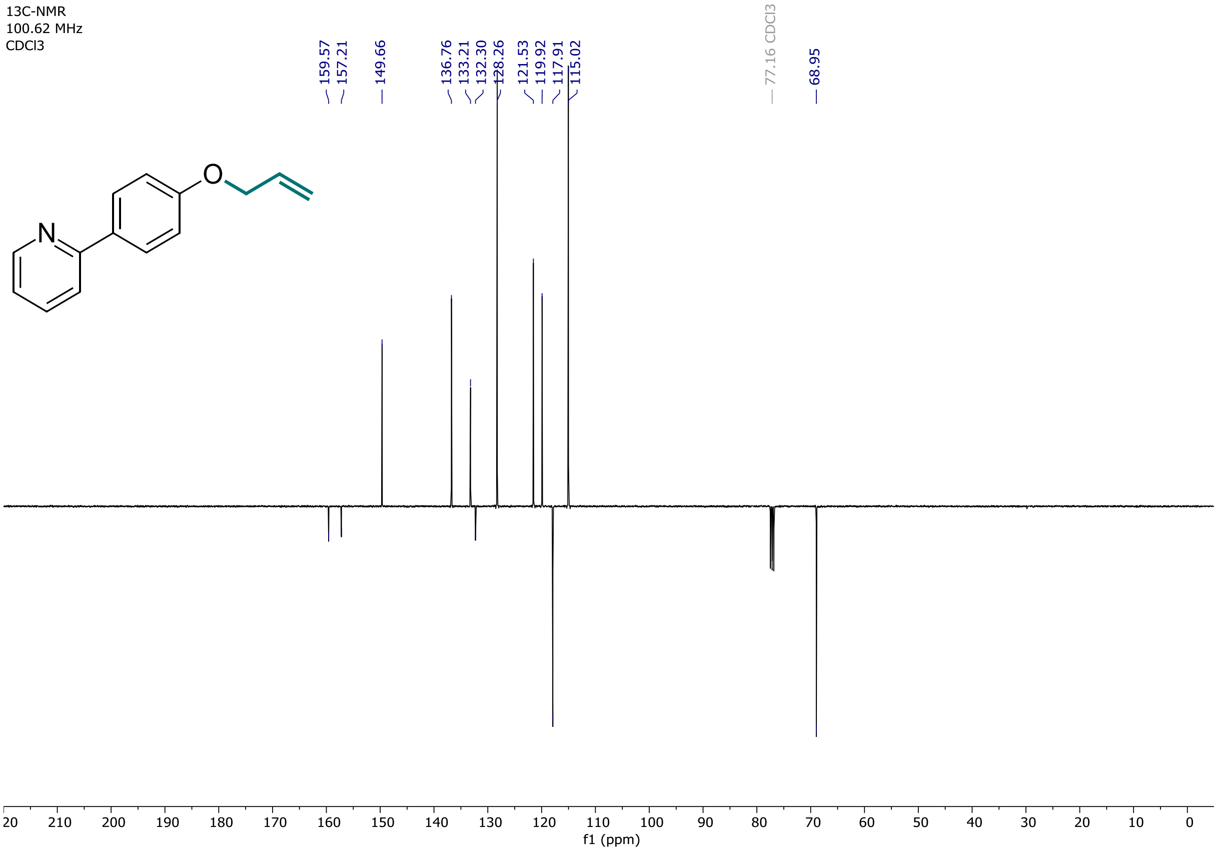

(E)-1-(Allyloxy)-3,7-dimethylocta-2,6-diene **(19)**

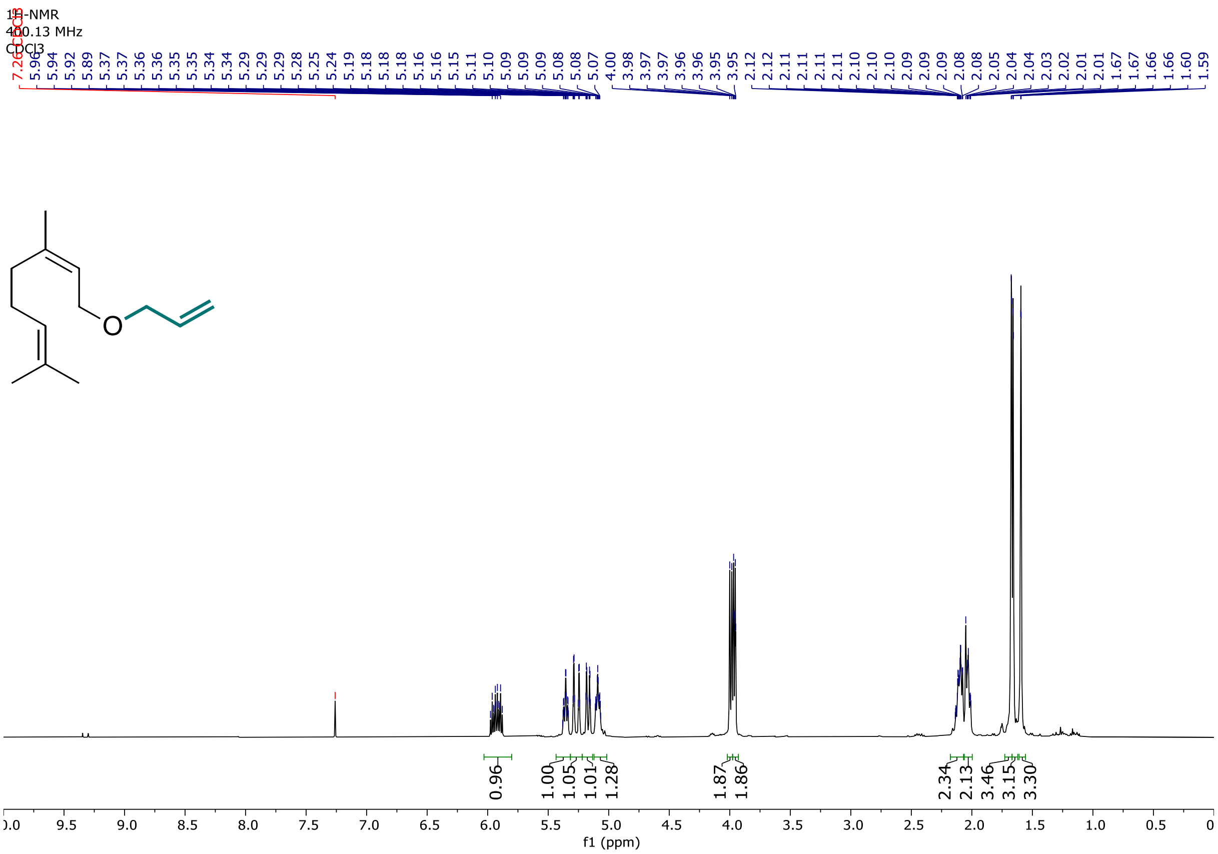

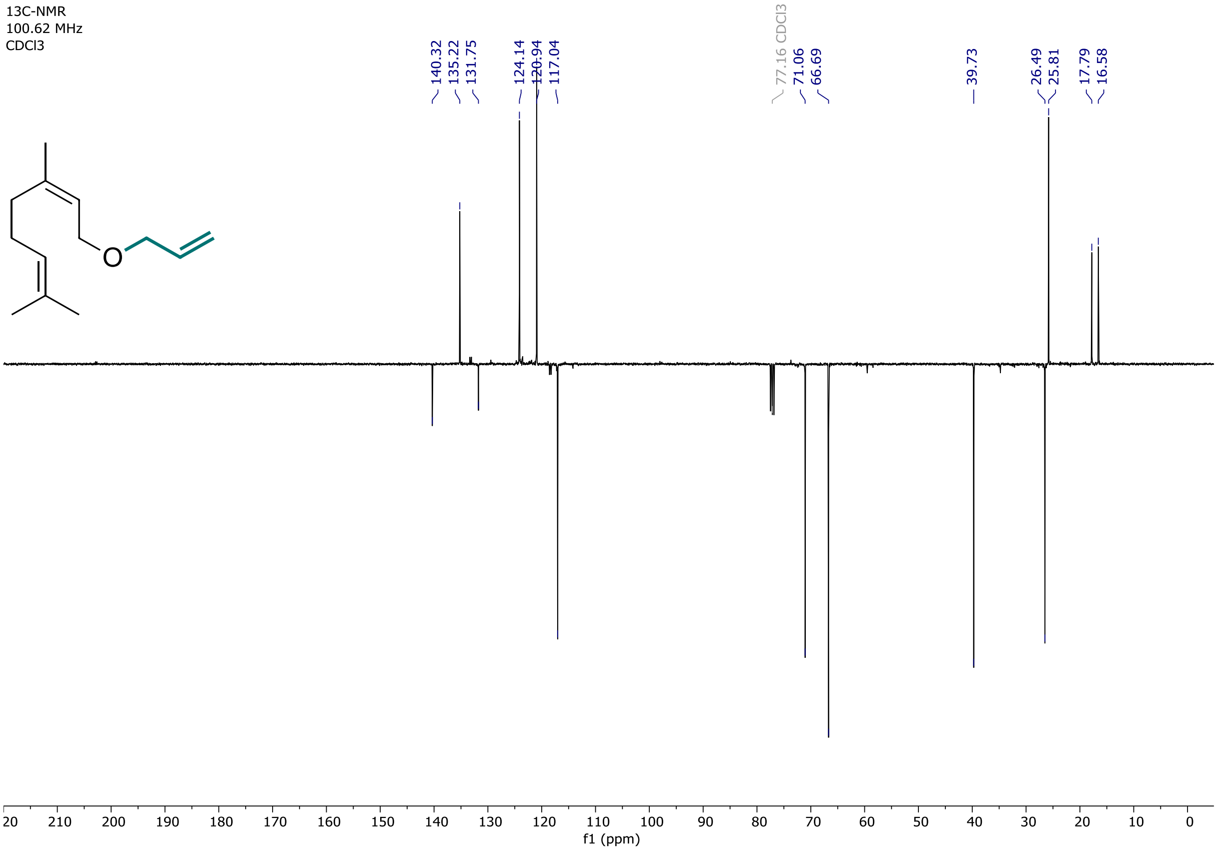

O-Allyl citronellol **(20)**

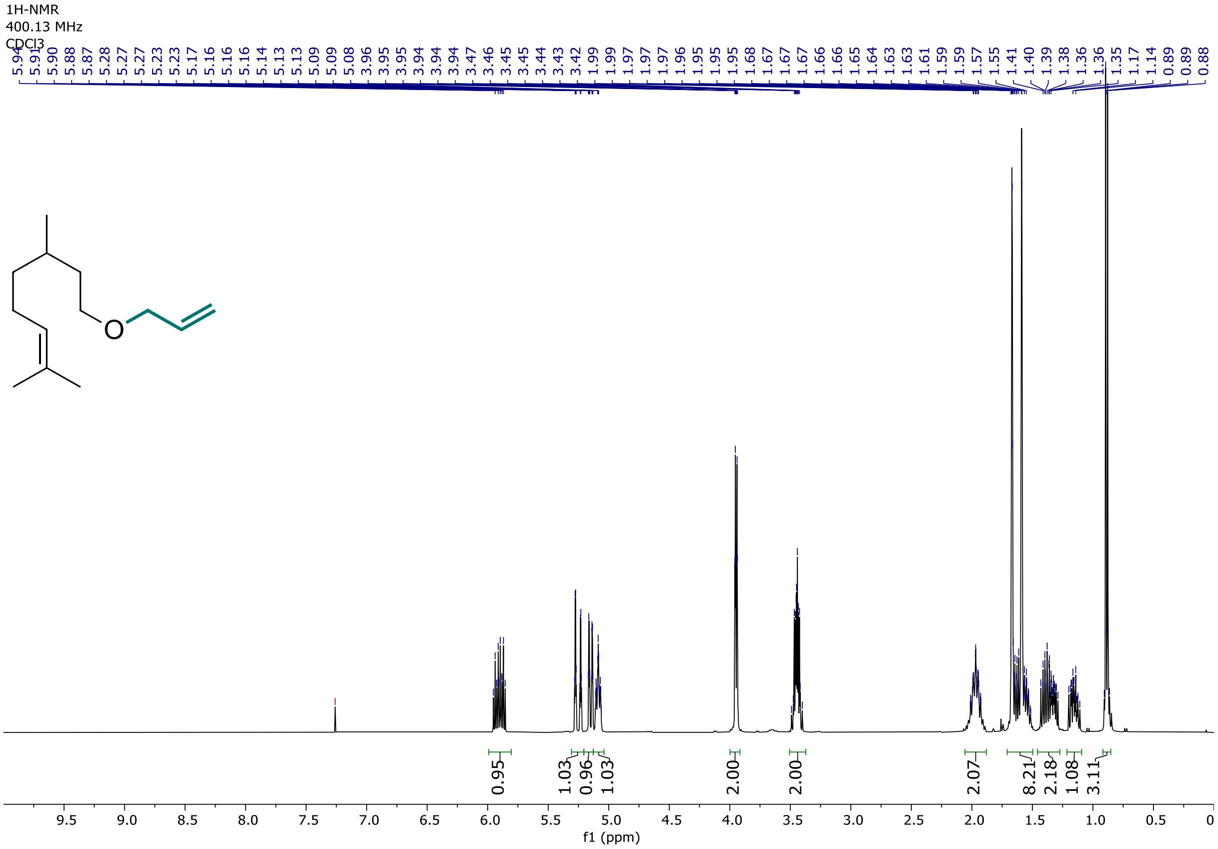

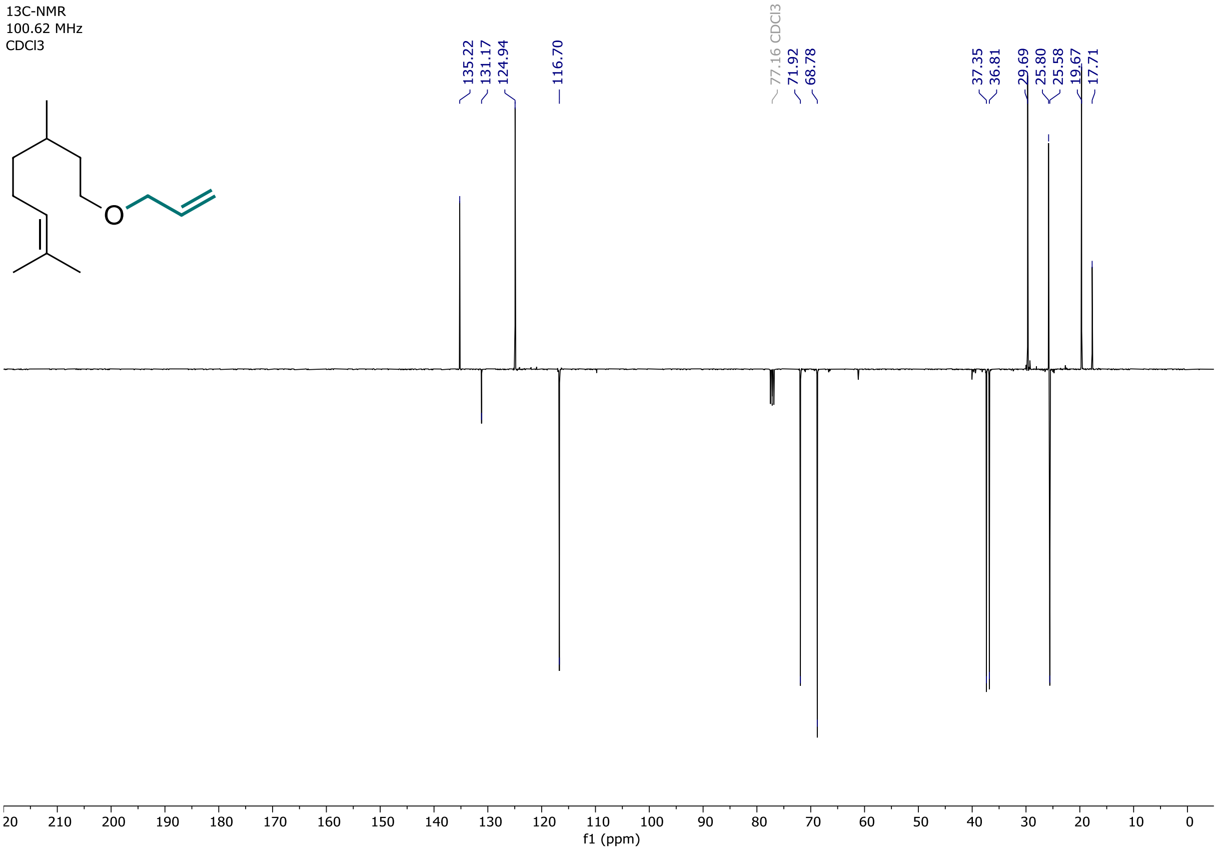

3-allyloxy estrone **(21)**

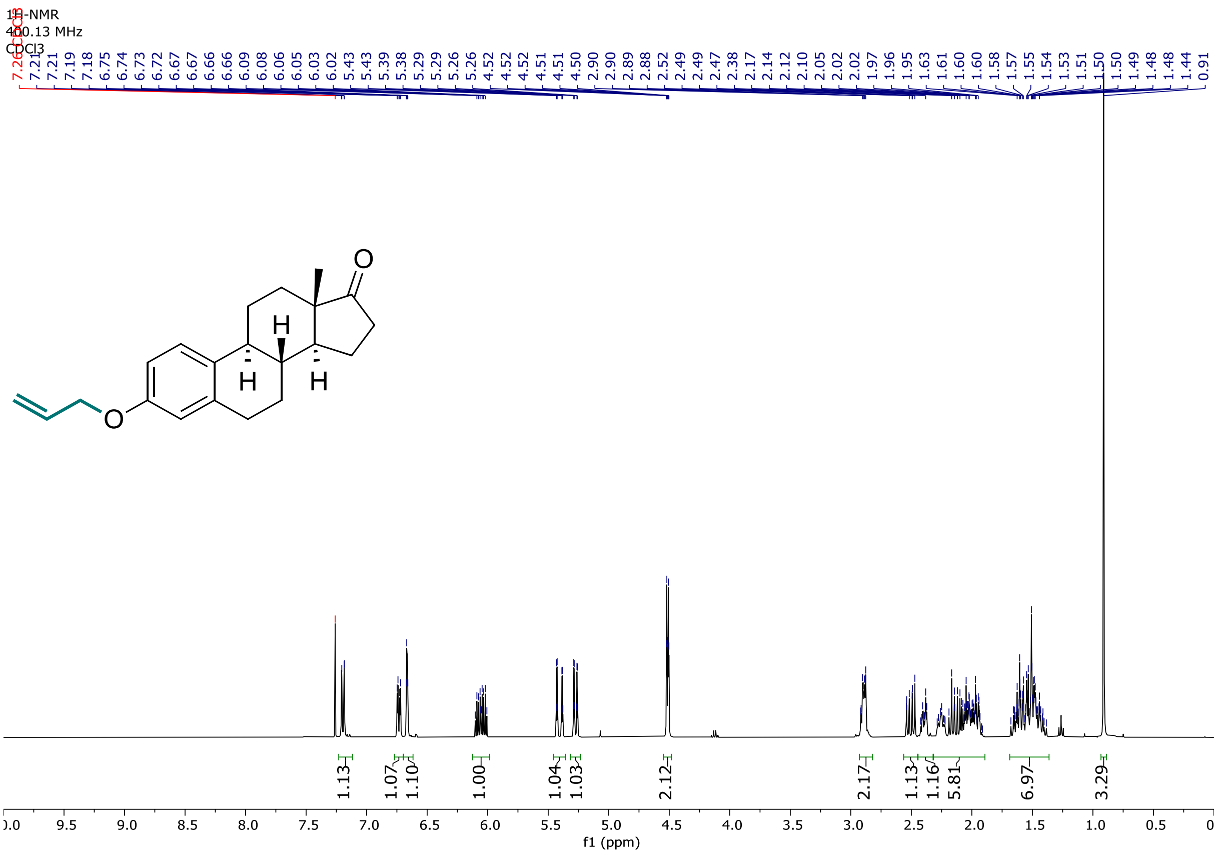

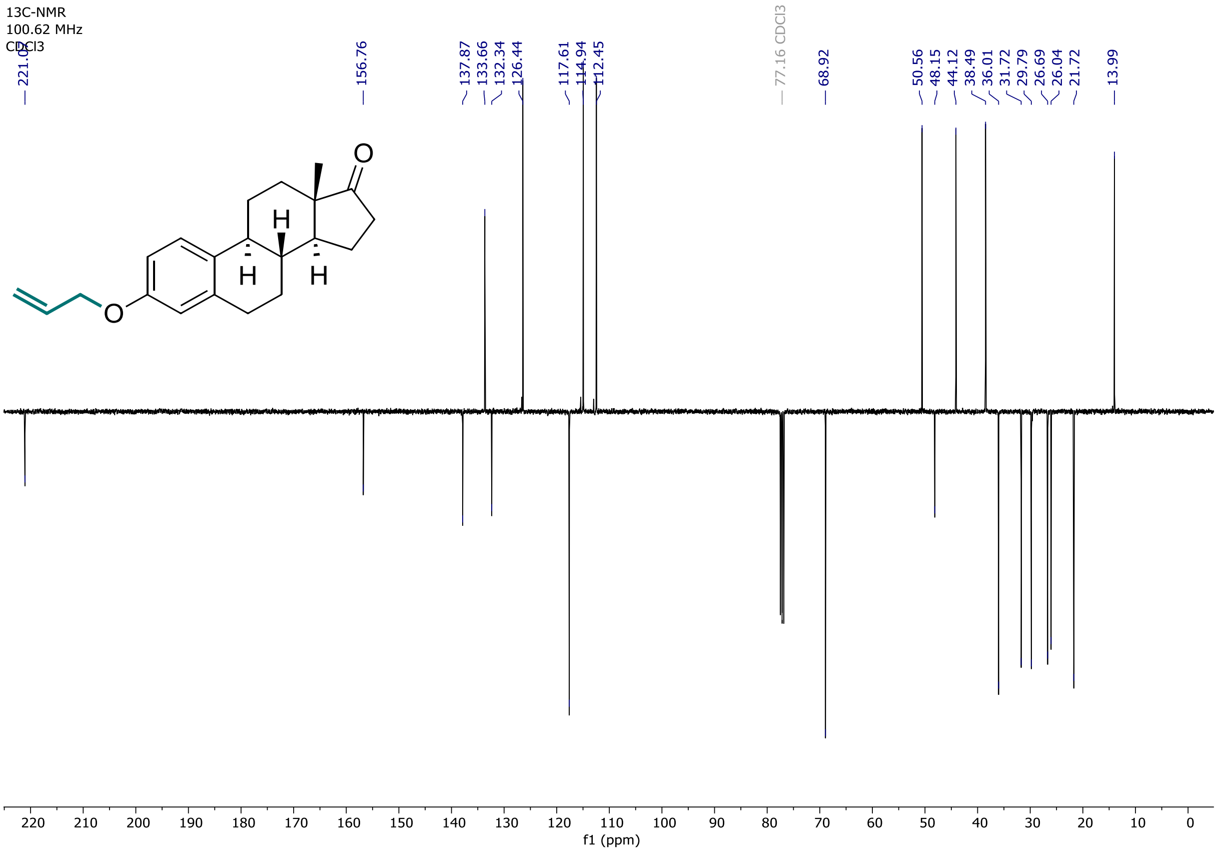

N-Allyl-4-fluoroaniline **(22)**

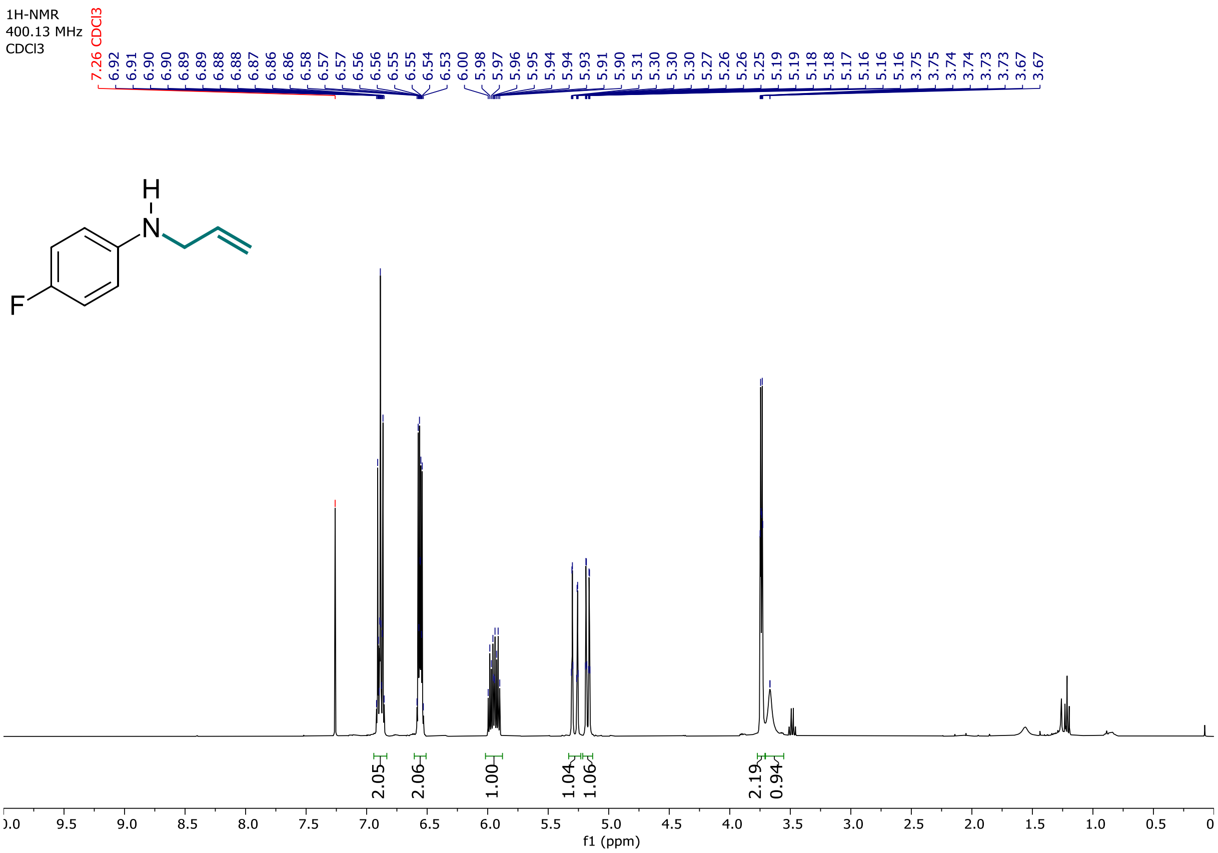

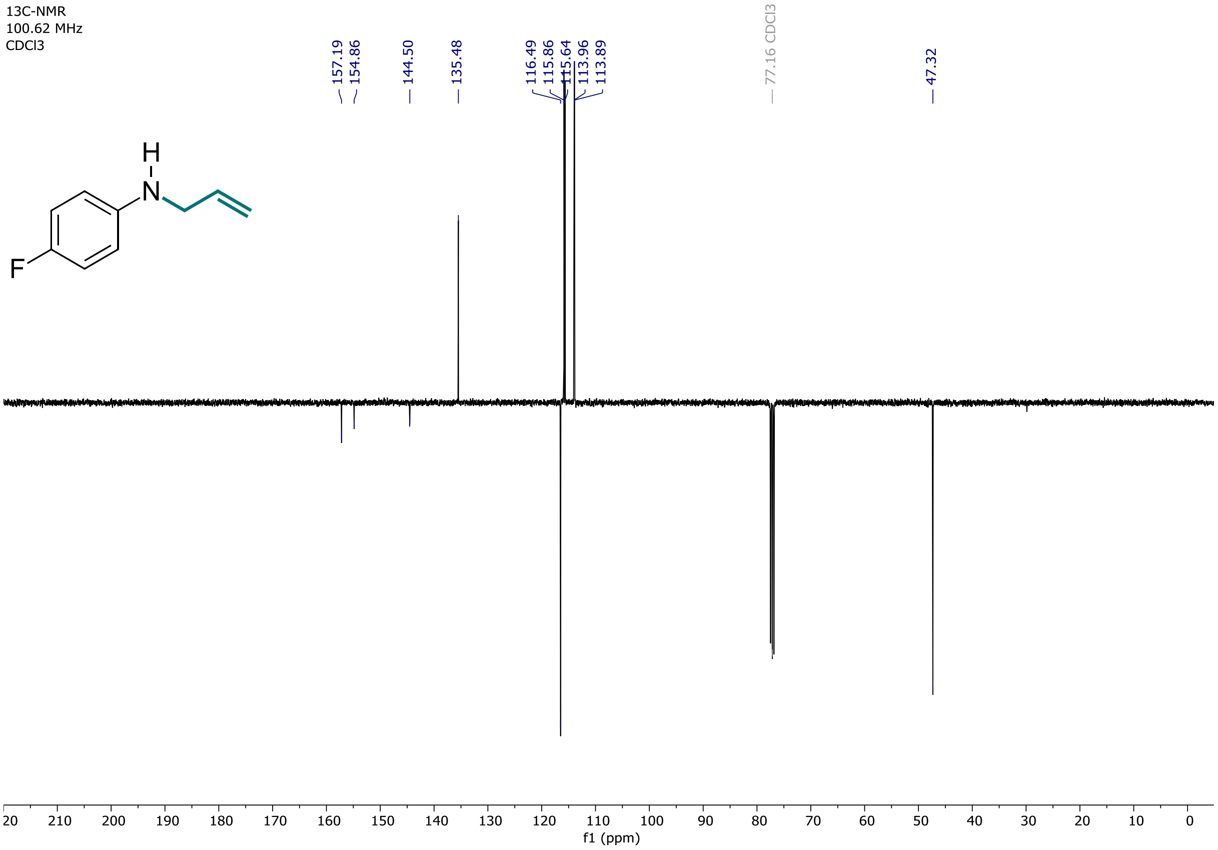

N-Allyl-4-fluoro-*N*-methylaniline **(23)**

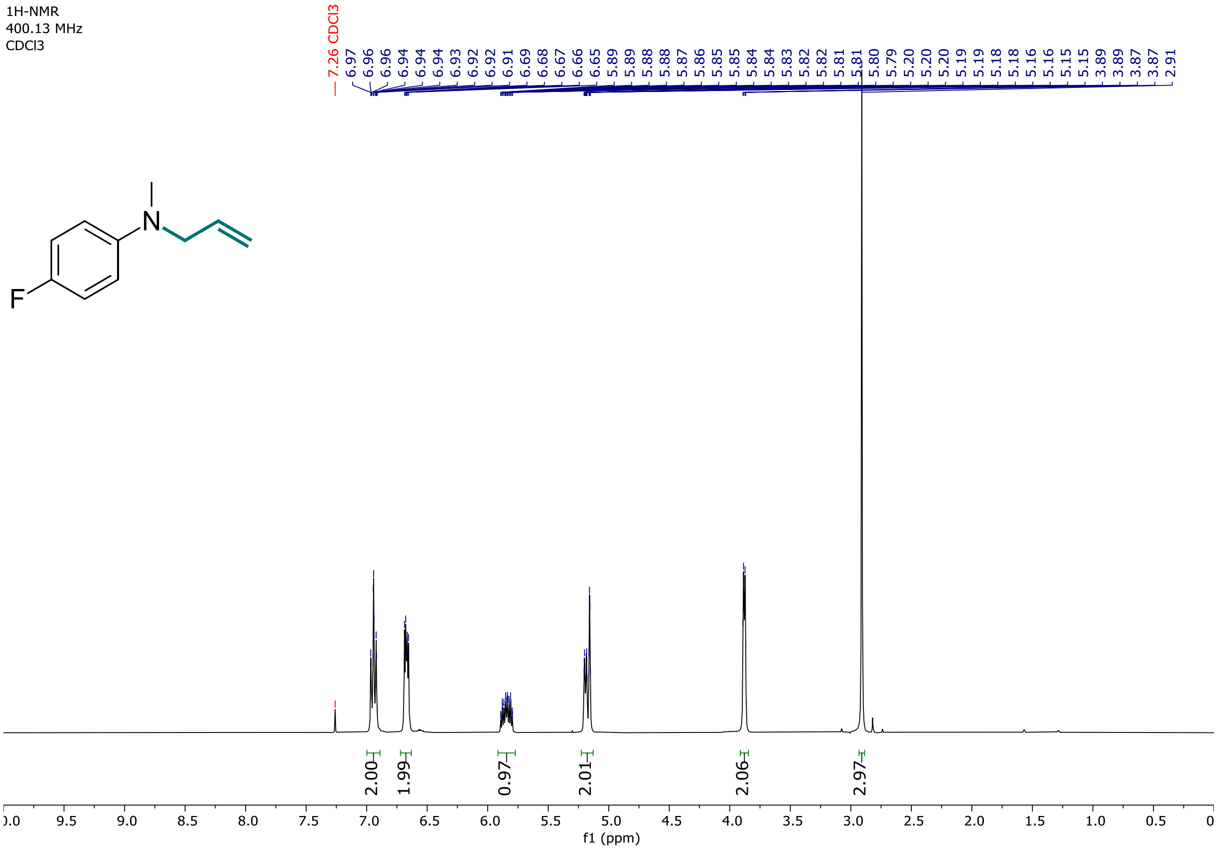

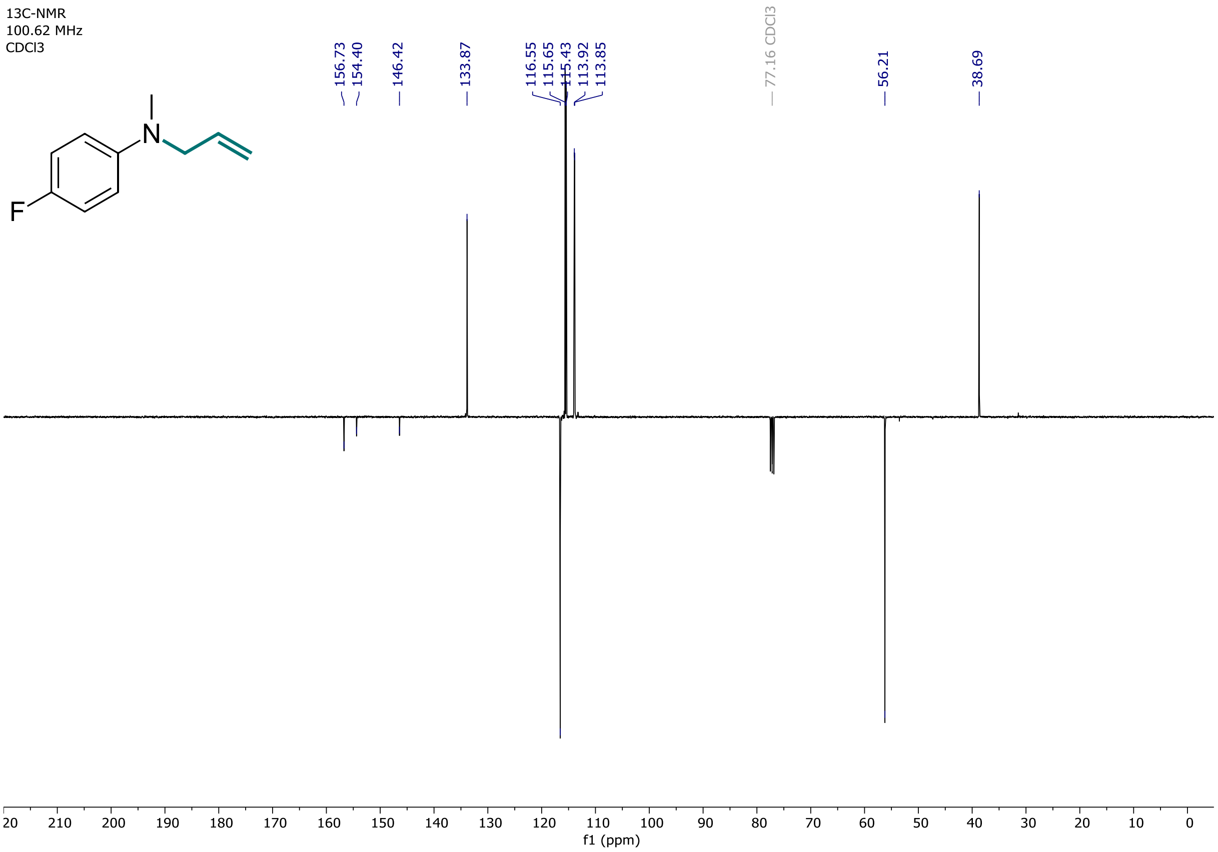

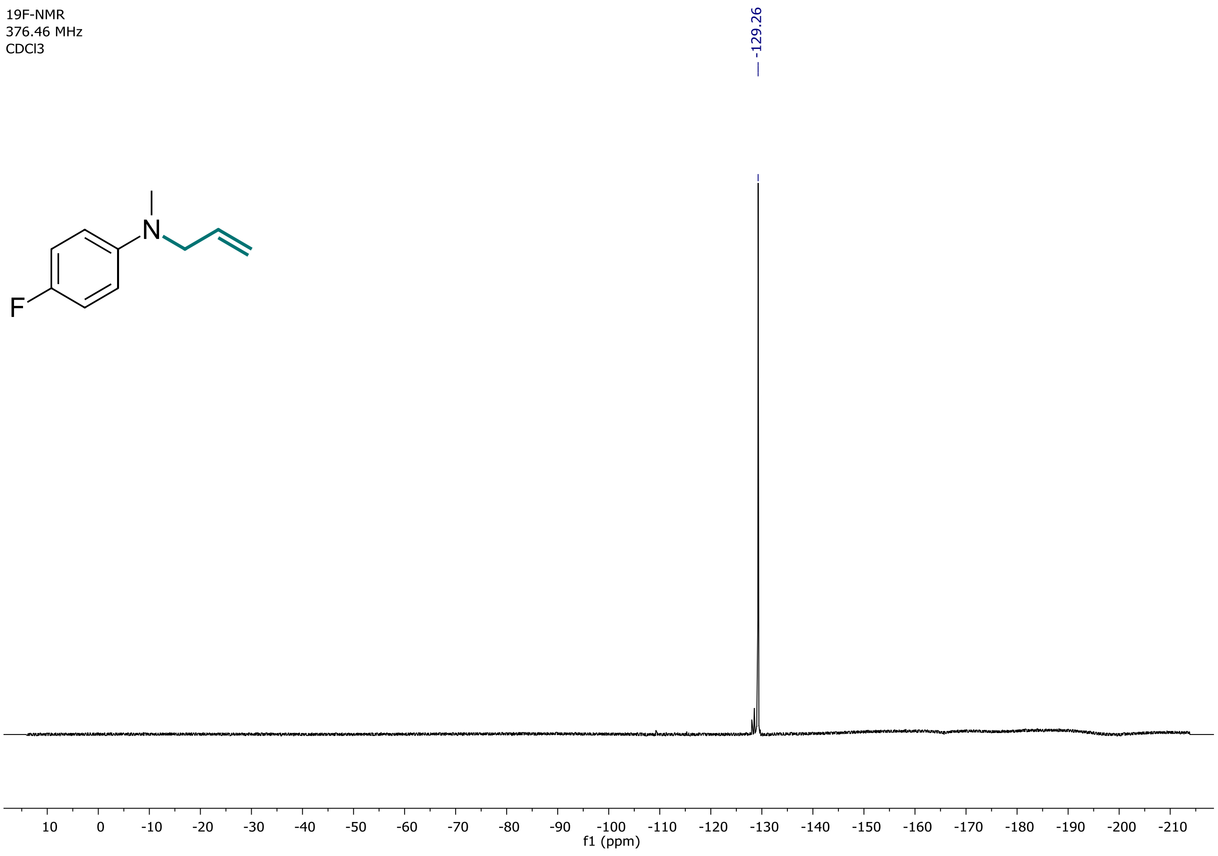

19F-NMR
376.46 MHz
CDCl3
-129.26
F
N
10 0 -10 -20 -30 -40 -50 -60 -70 -80 -90 -100 -110 -120 -130 -140 -150 -160 -170 -180 -190 -200 -210
f1 (ppm)

N,N-diallyl-4-fluoroaniline **(24)**

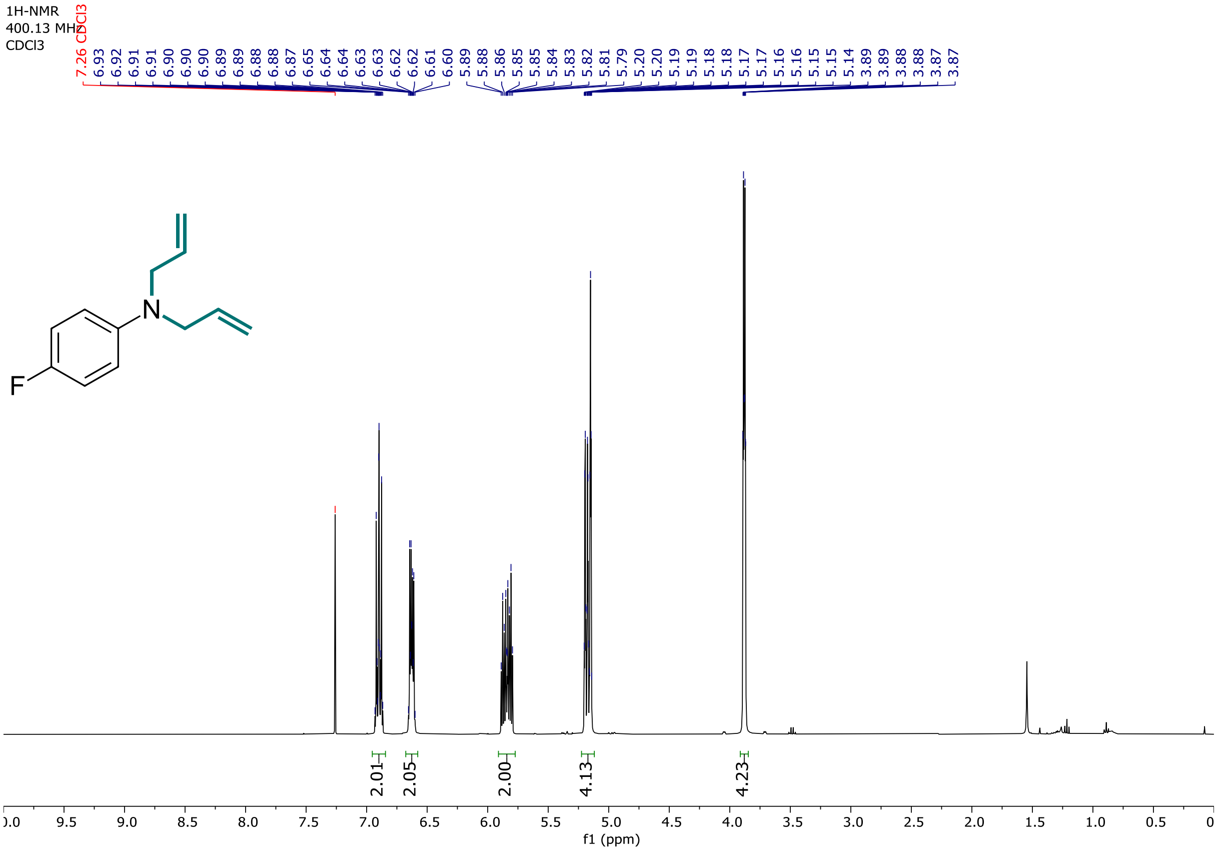

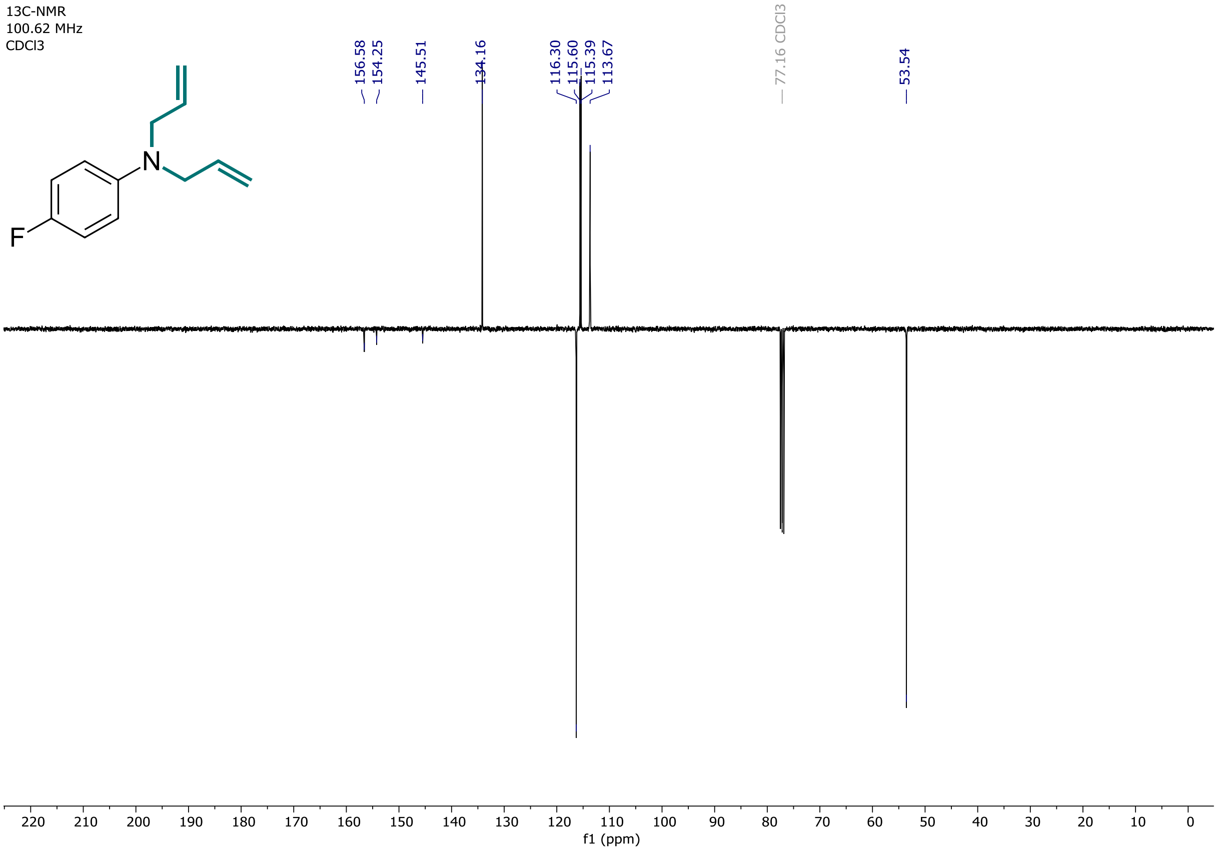

1-Allylbenzimidazole **(25)**

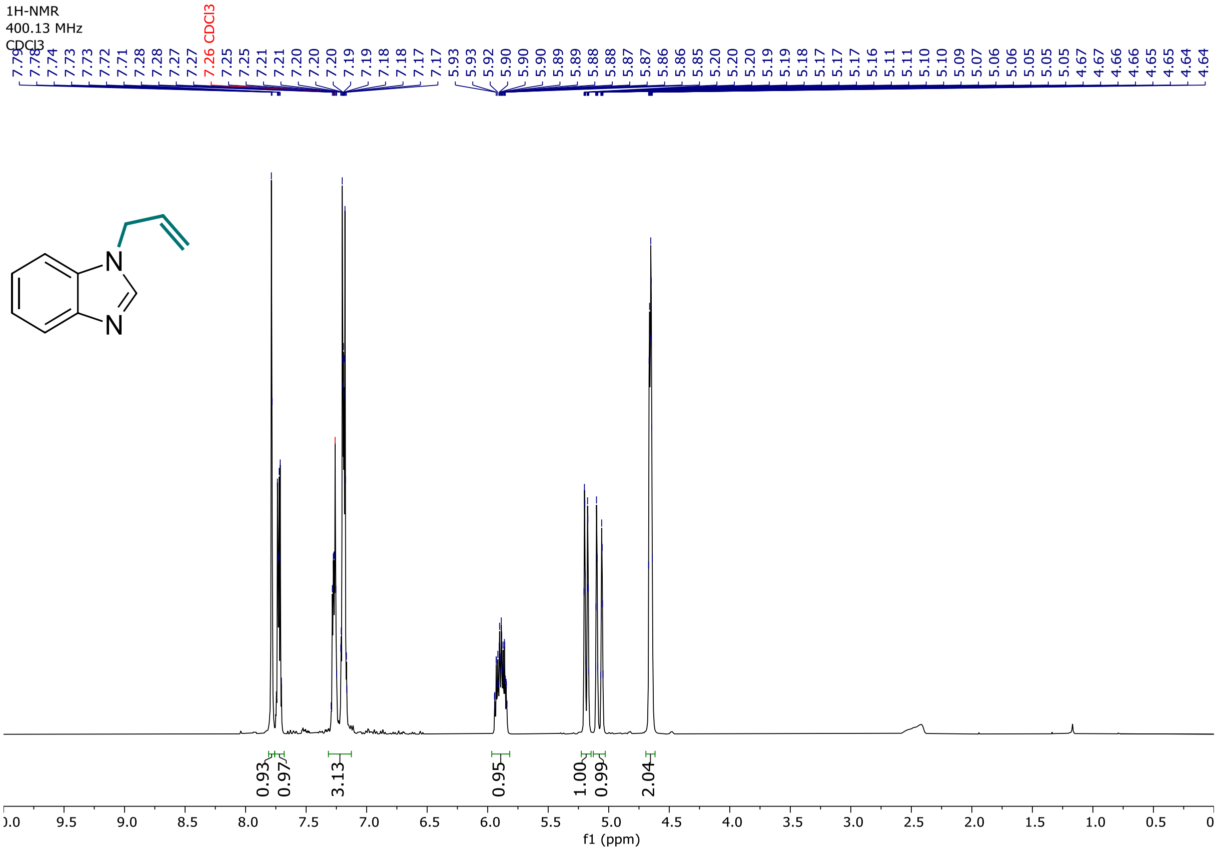

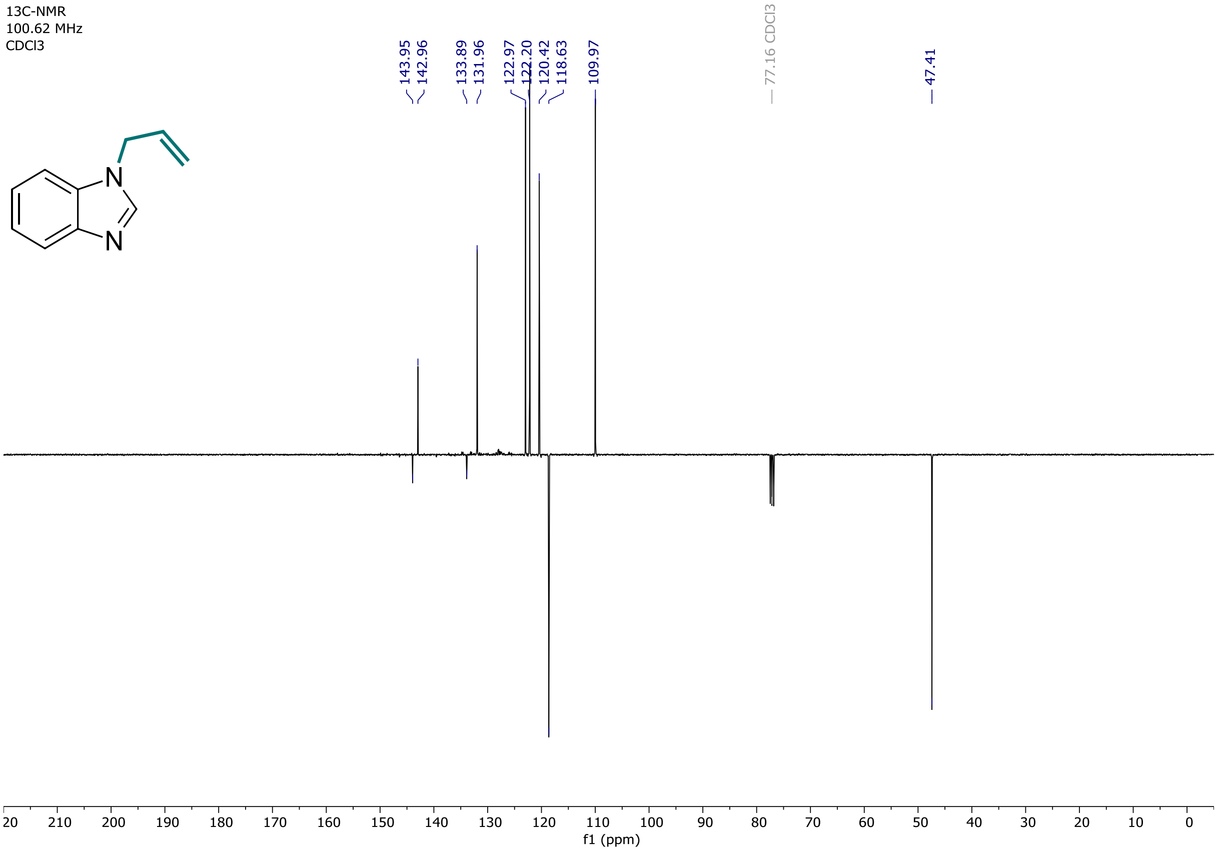

1-Allylbenzotriazole **(26)**

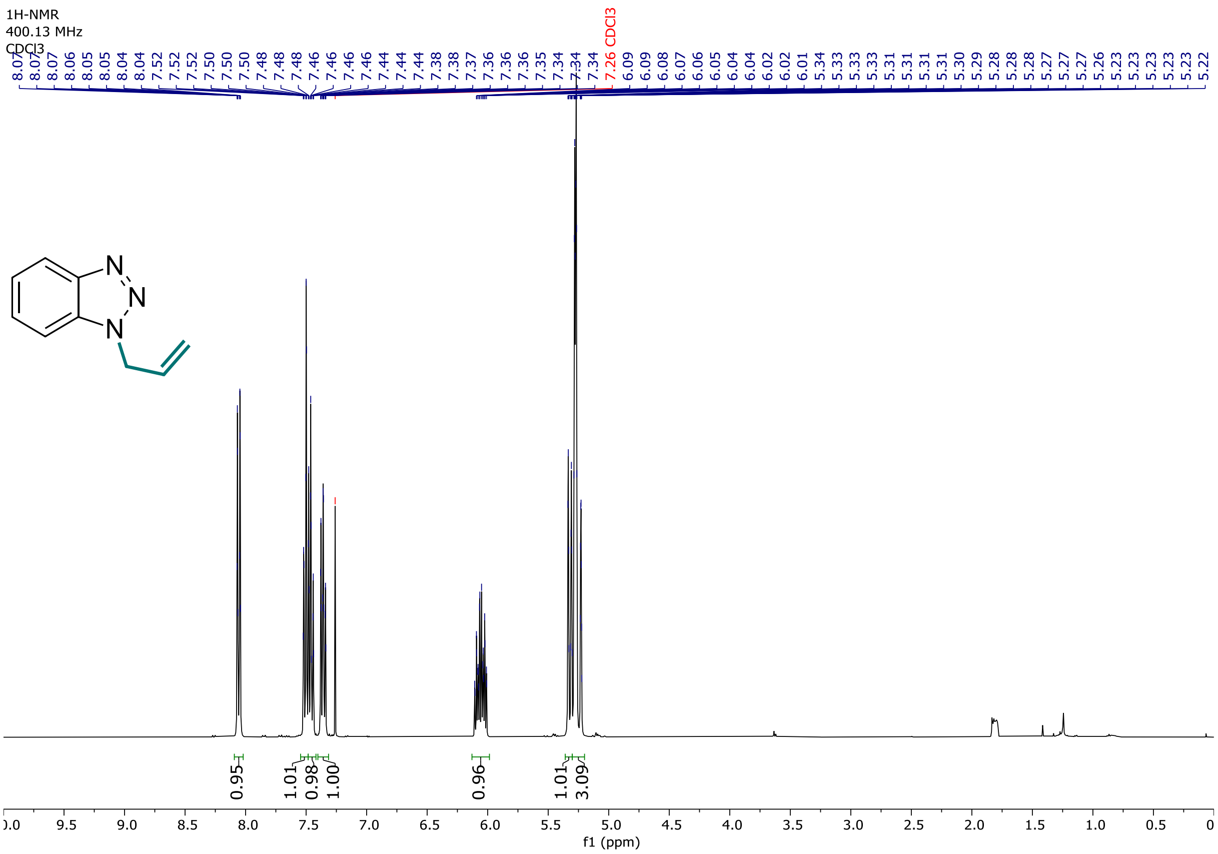

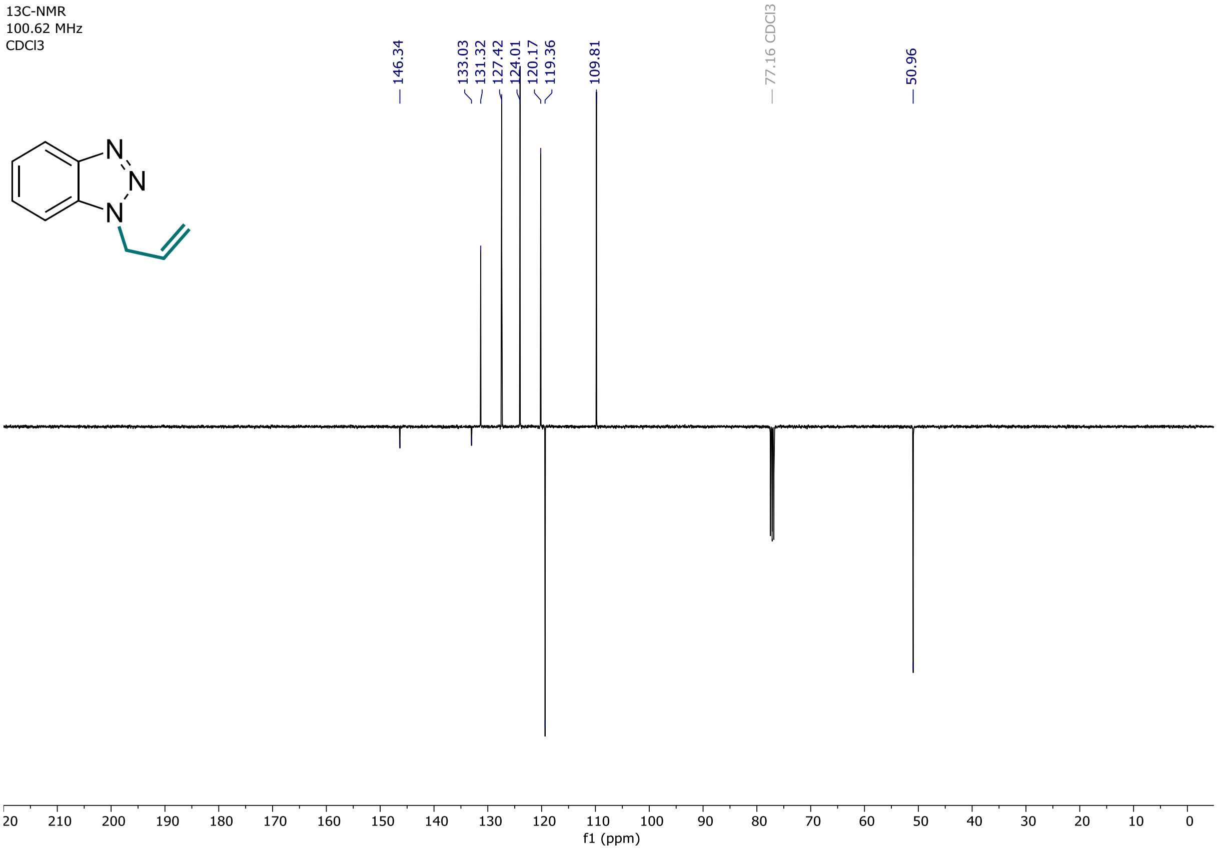

1-Allyl-4-(2-methoxy-phenyl)-piperazine **(27)**

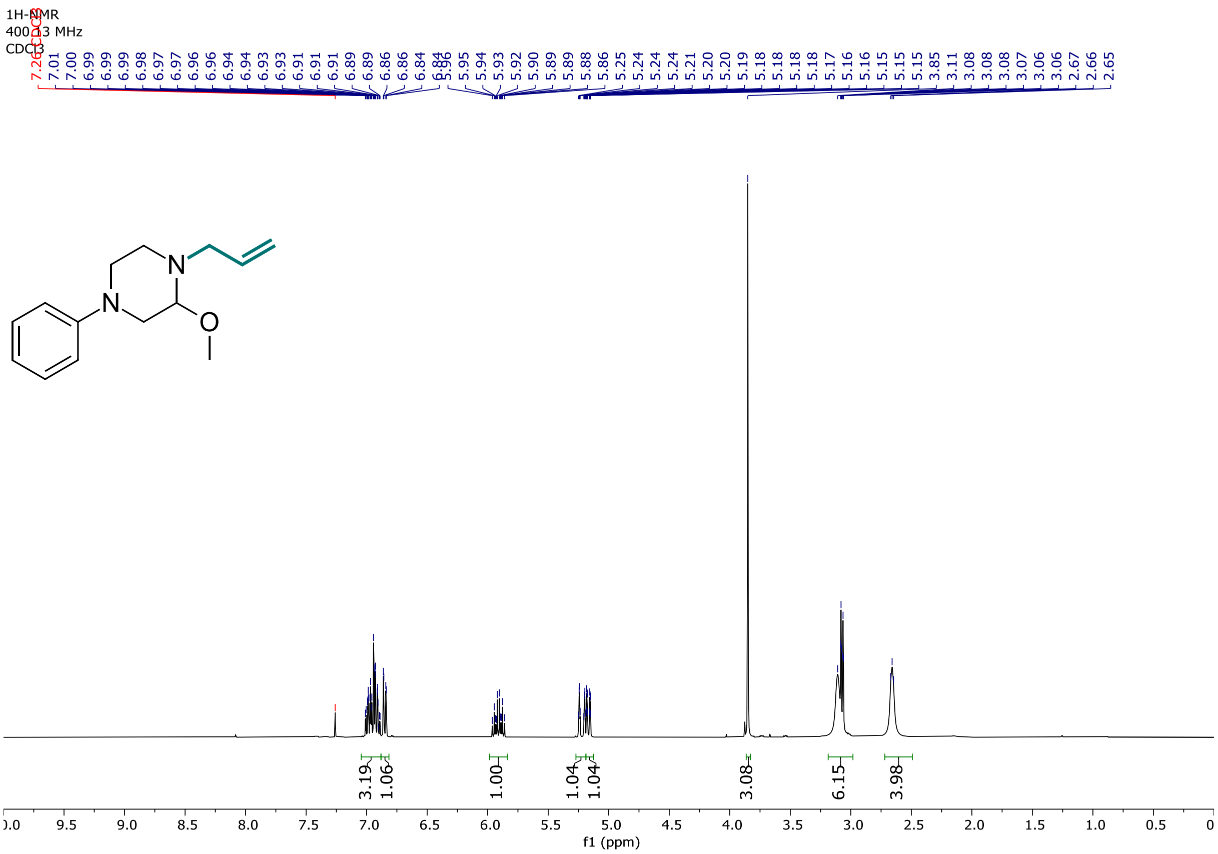

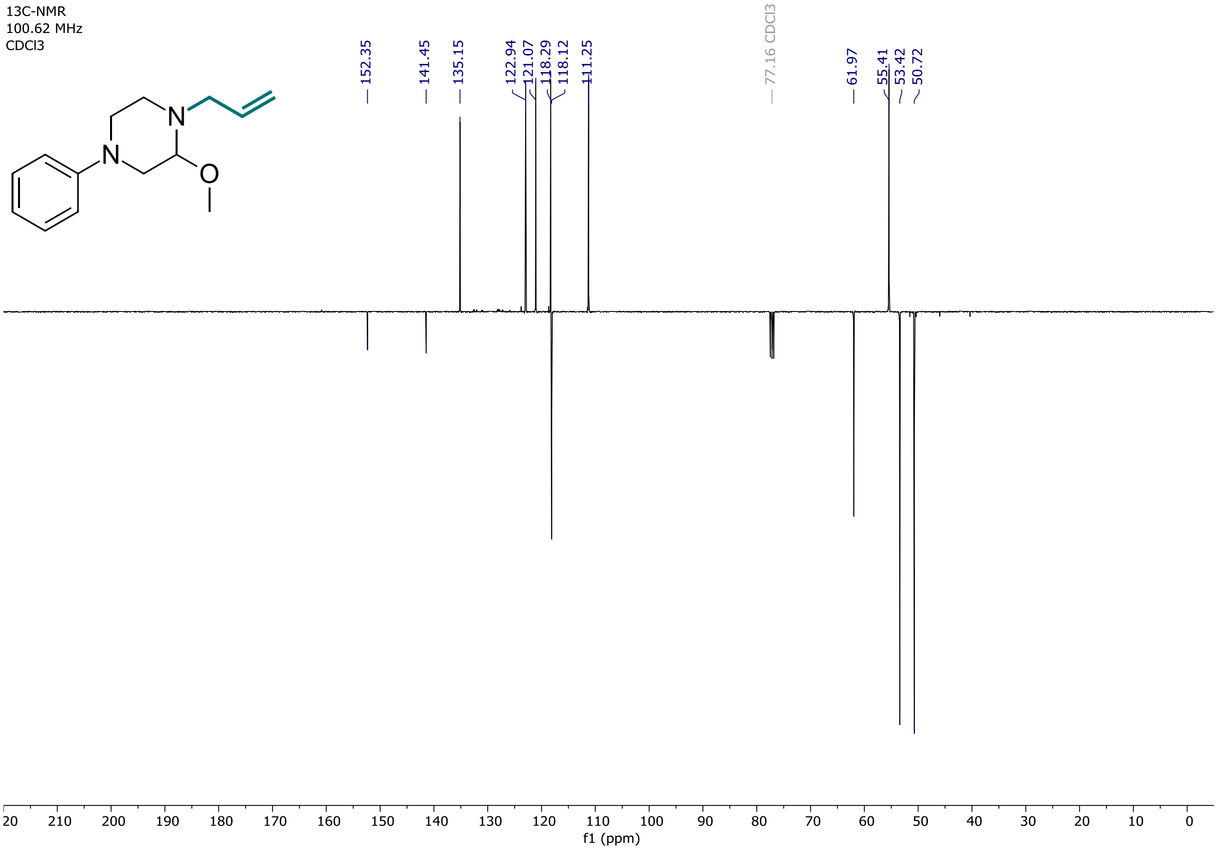

N-Allyl-N-phenylhydrazine **(28)**

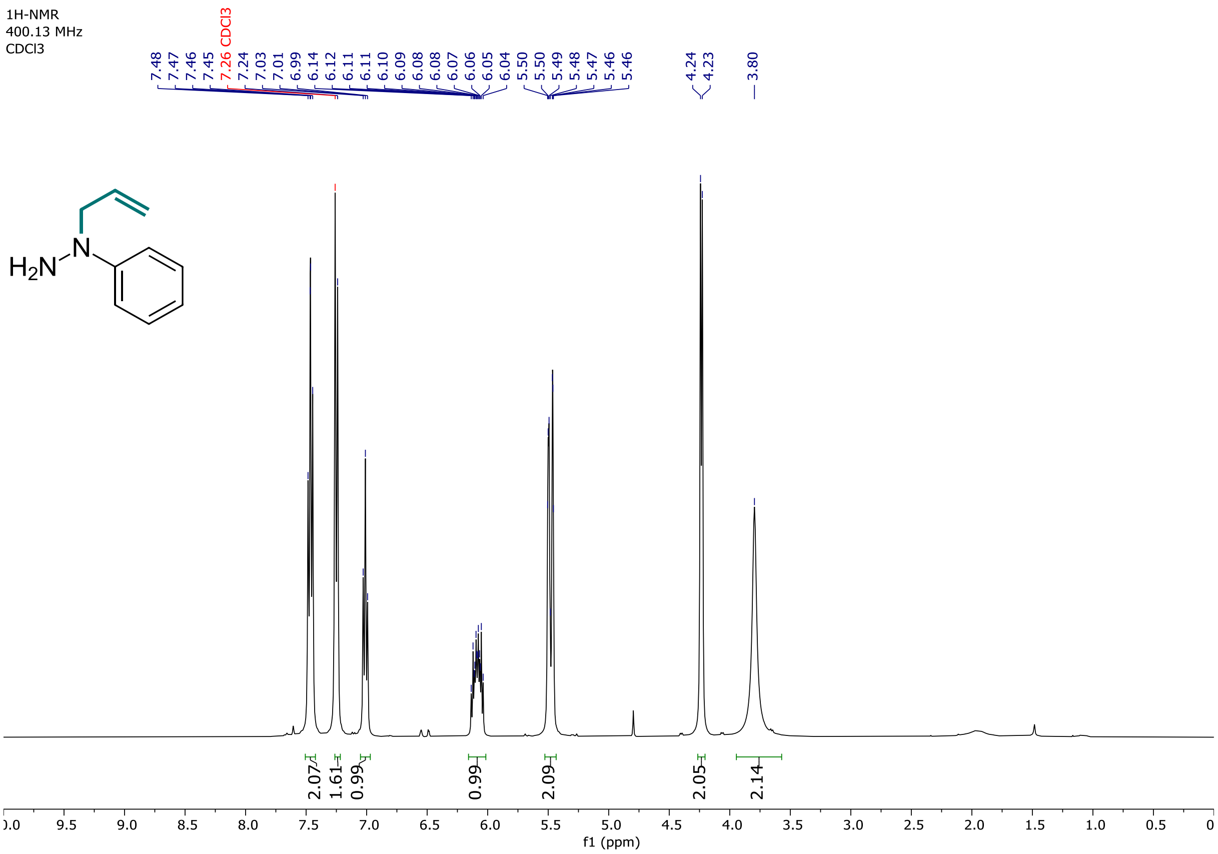

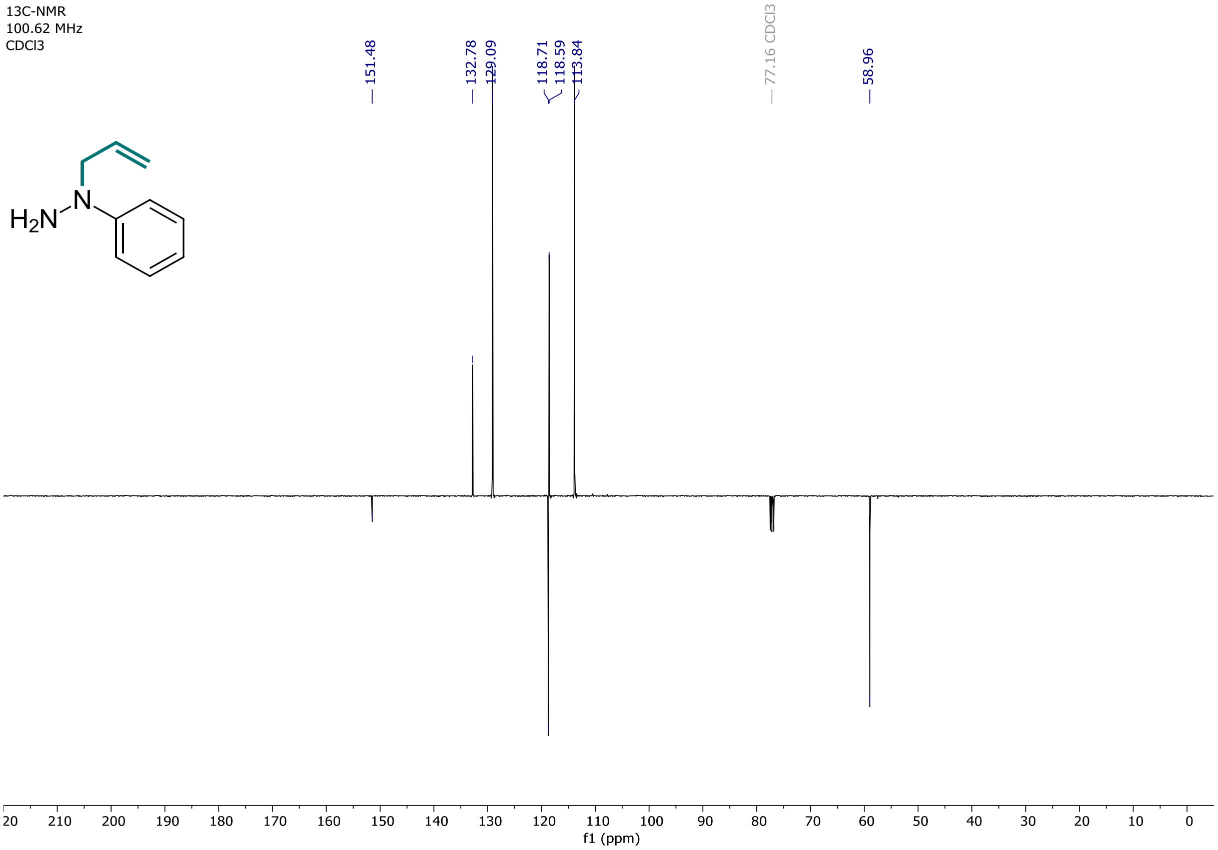

N-Allylphenothiazine **(29)**

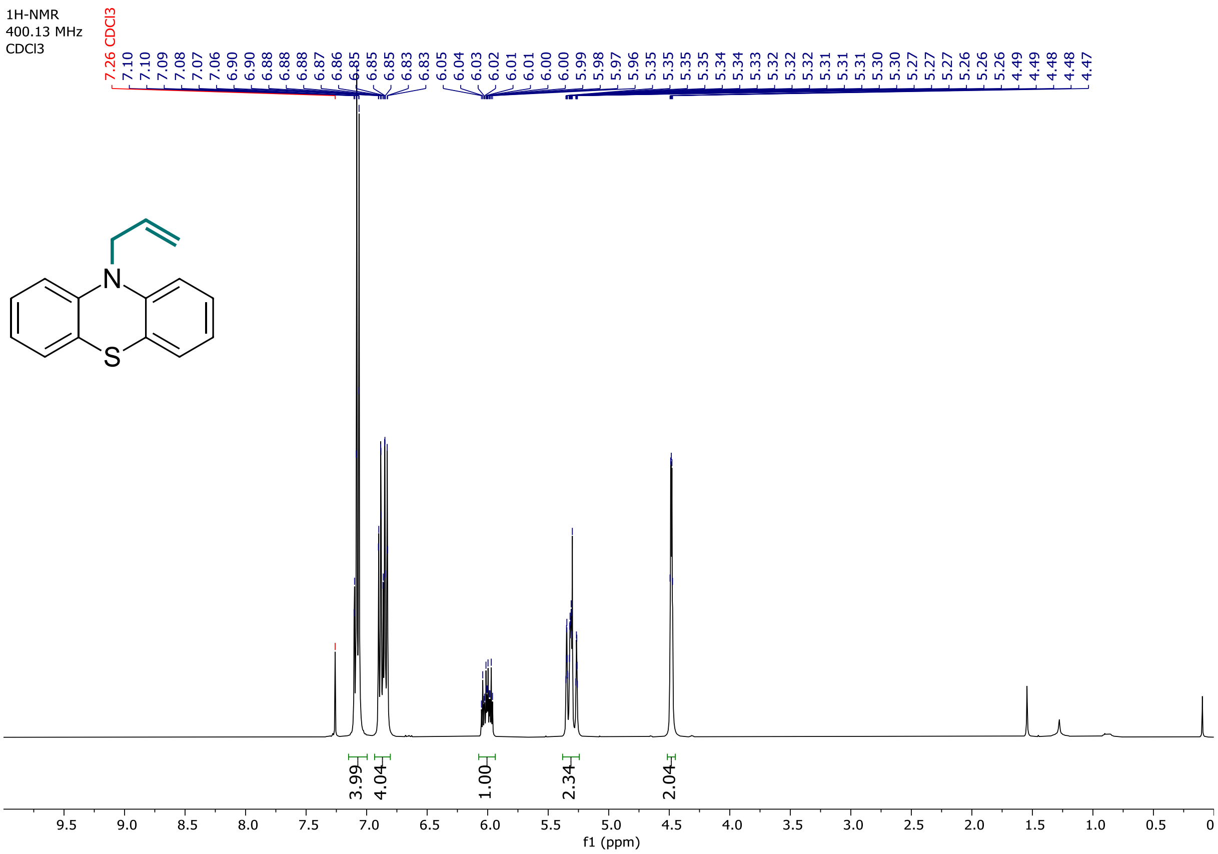

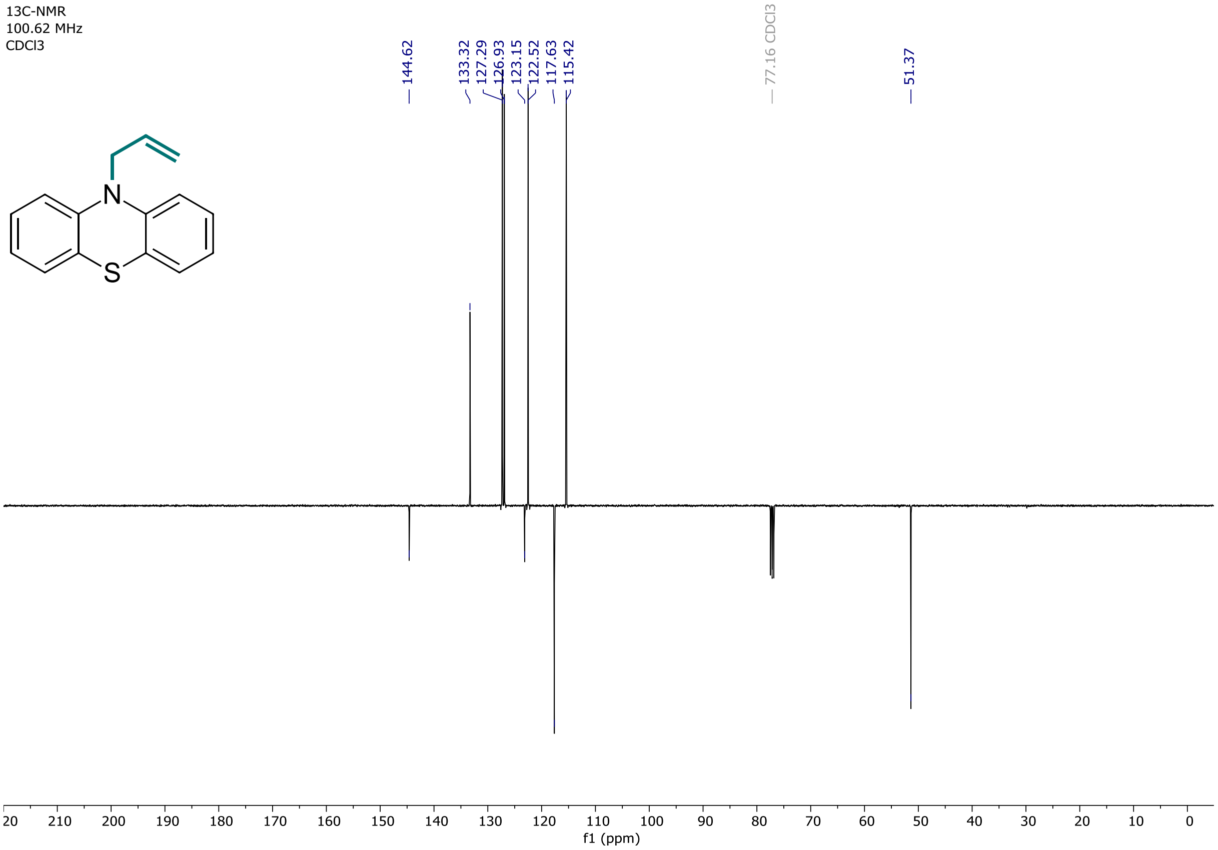

7-Allyl1,3-dimethylxanthine **(30)**

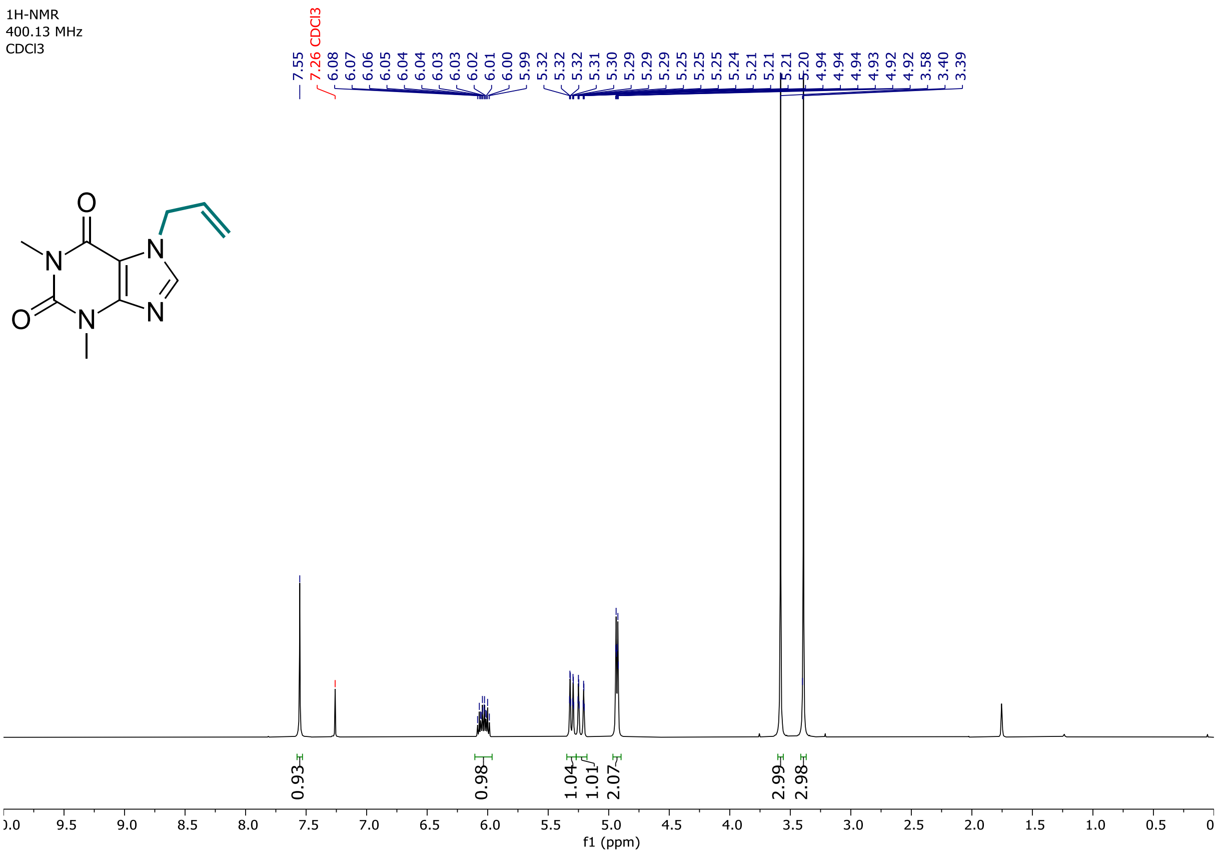

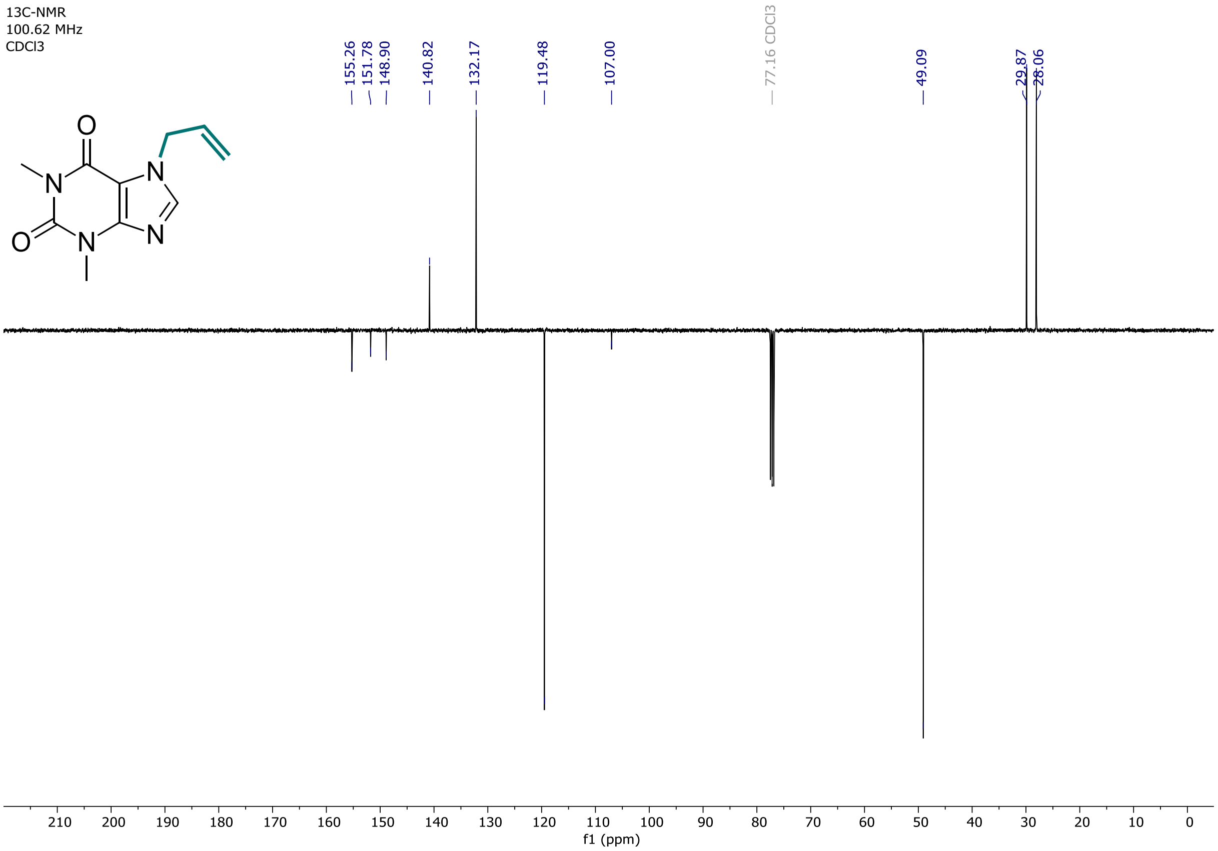

N-allyl azathioprine **(31)**

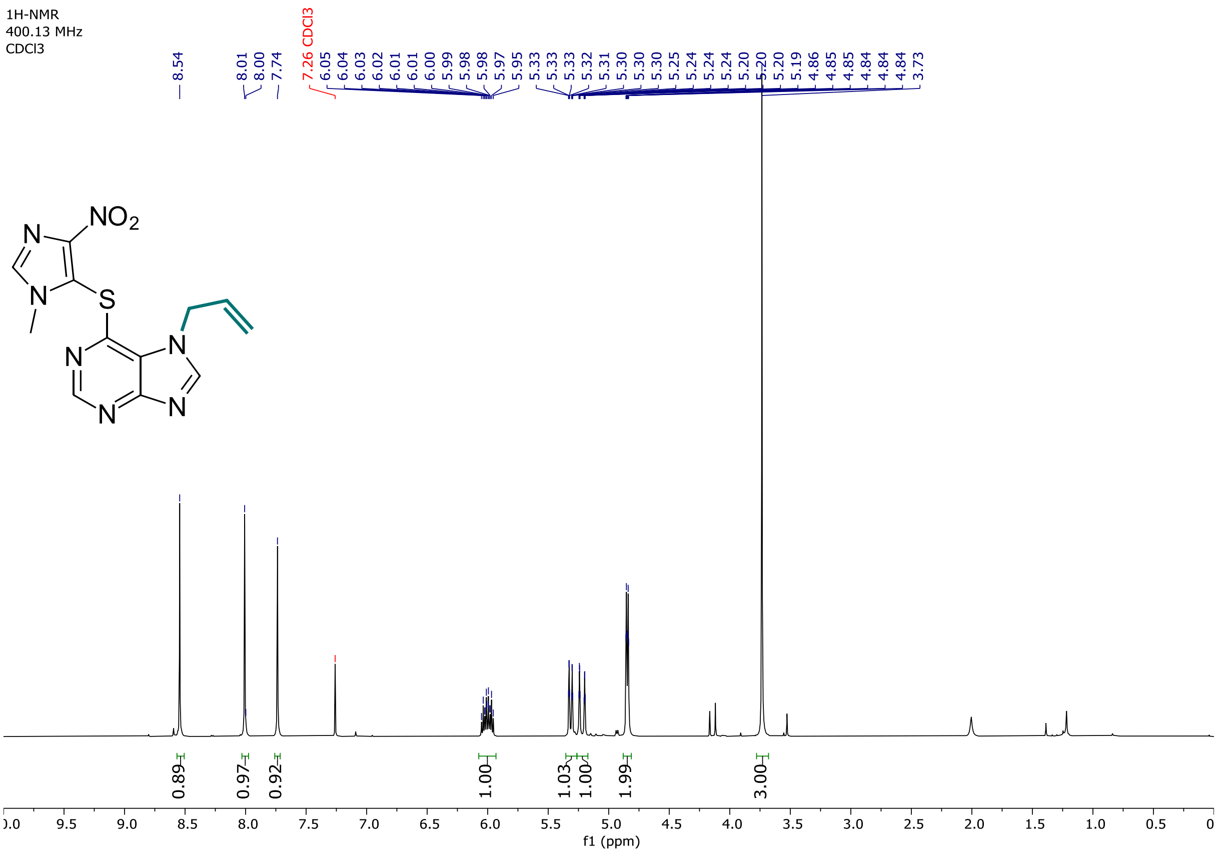

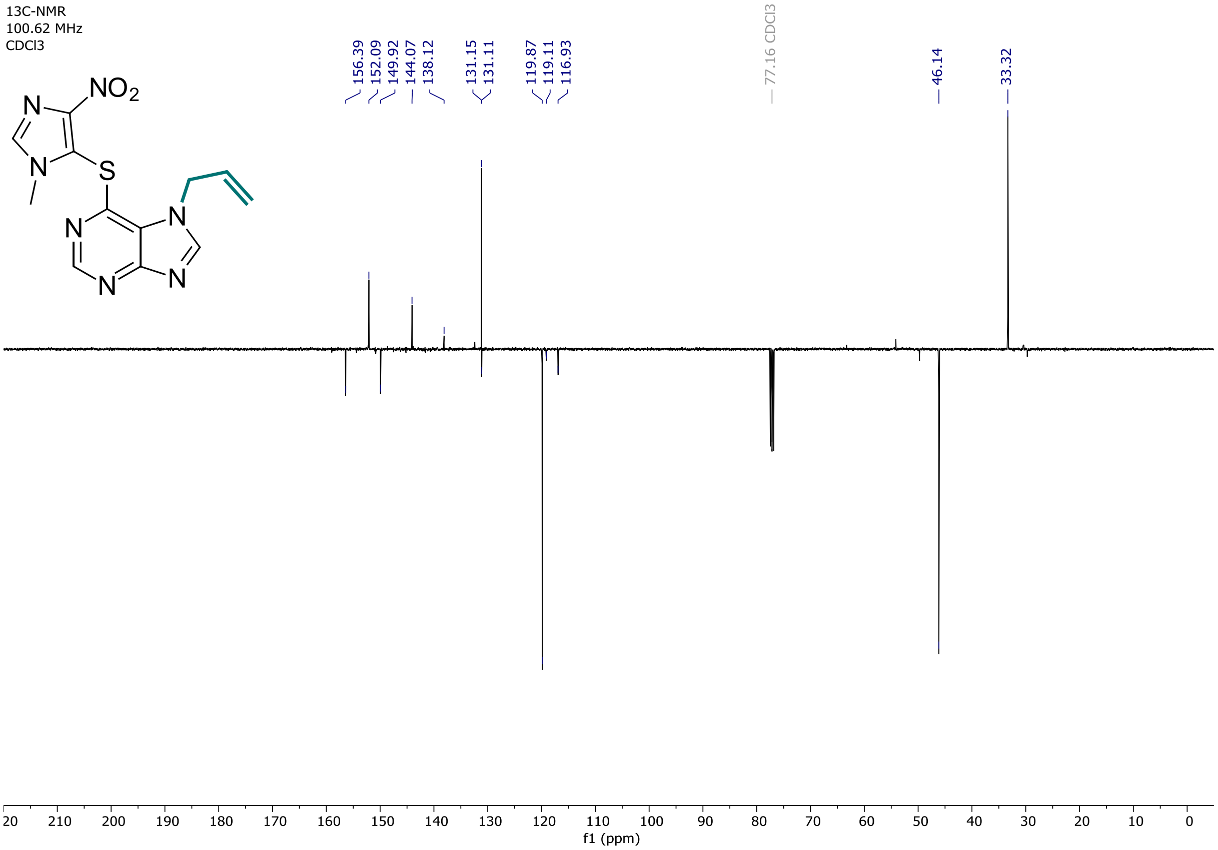

N-Allyl betahistine **(32)**

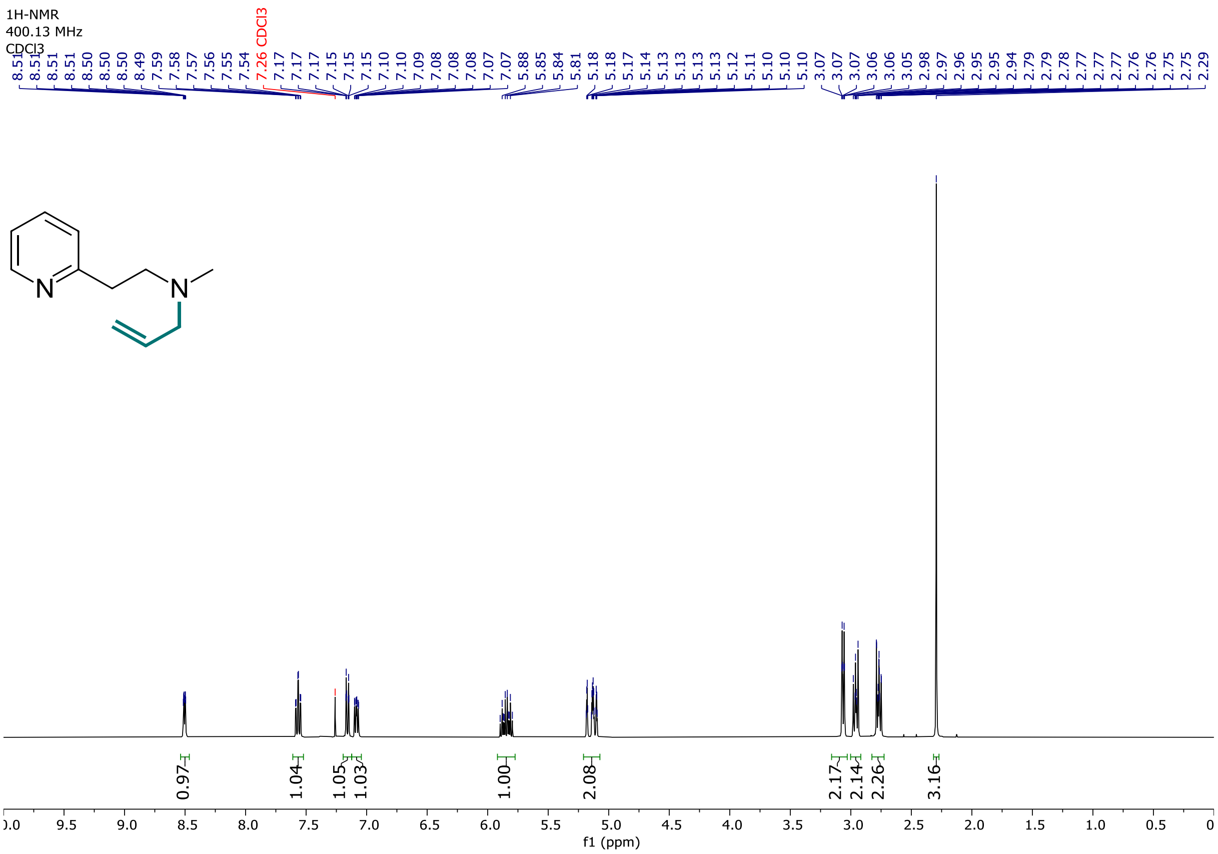

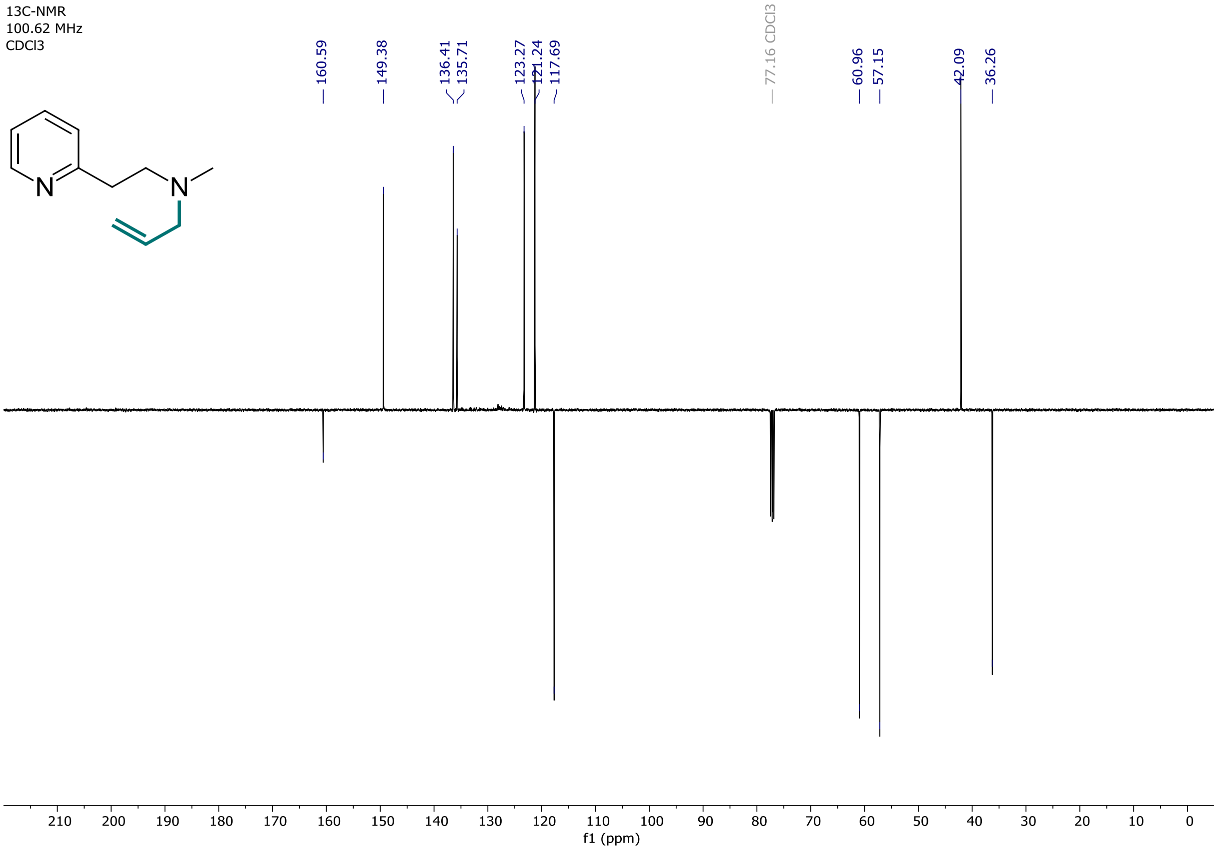

N-Allyl fluoxetine **(33)**

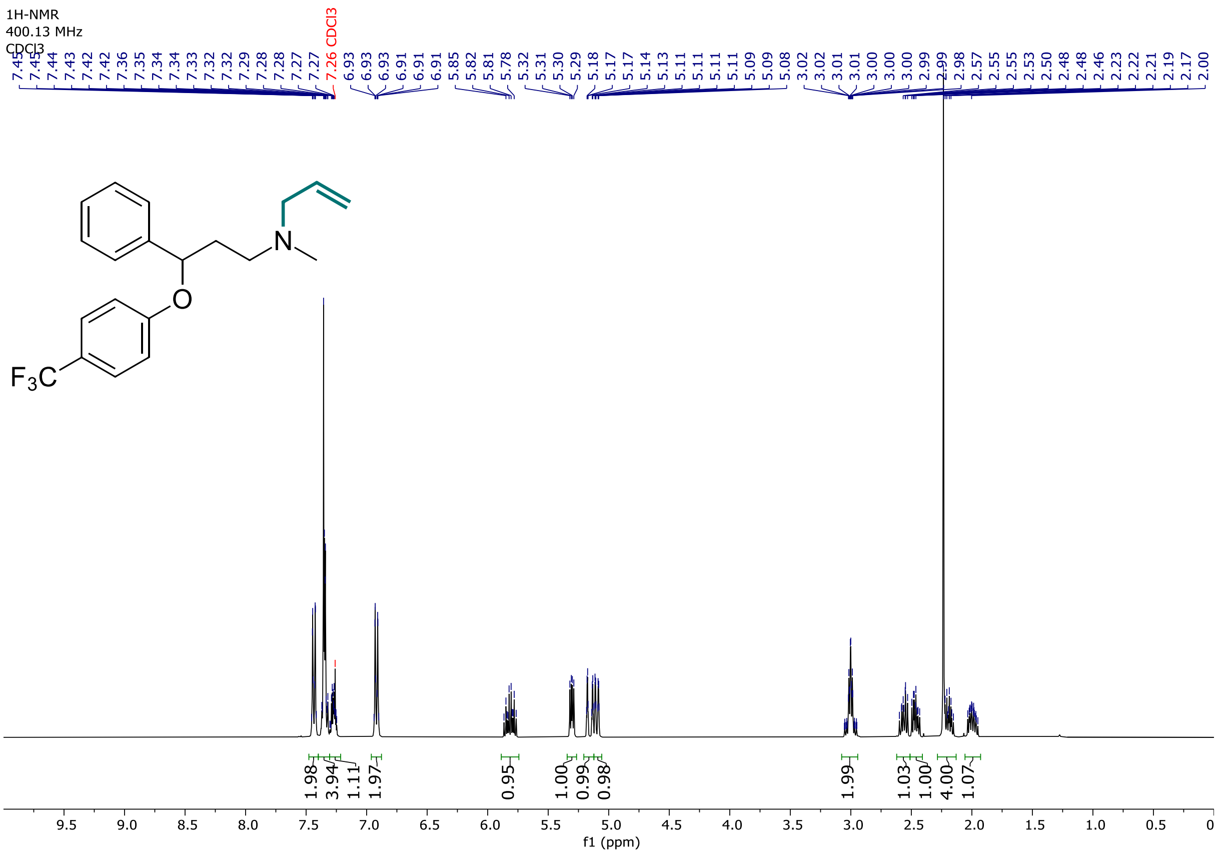

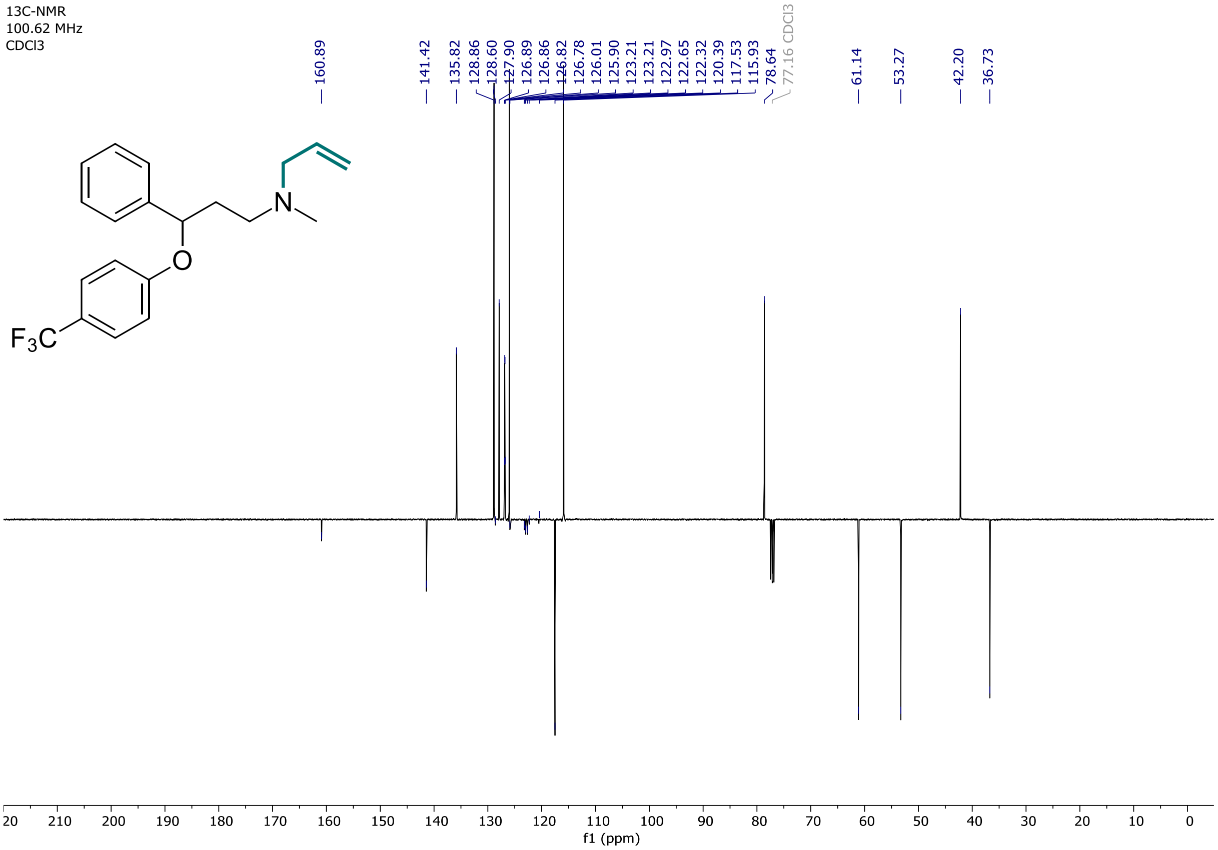

19F-NMR
376.46 MHz
CDCl3

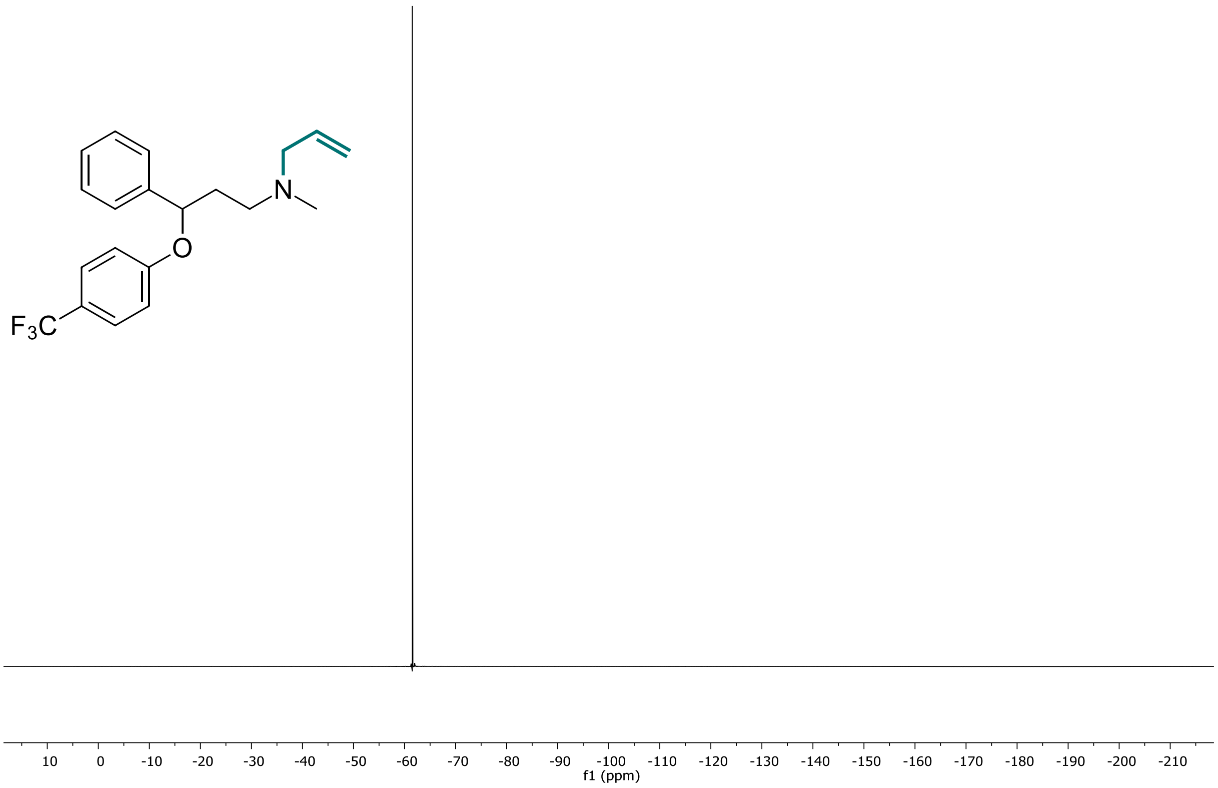

N-Allyl duloxetine **(34)**

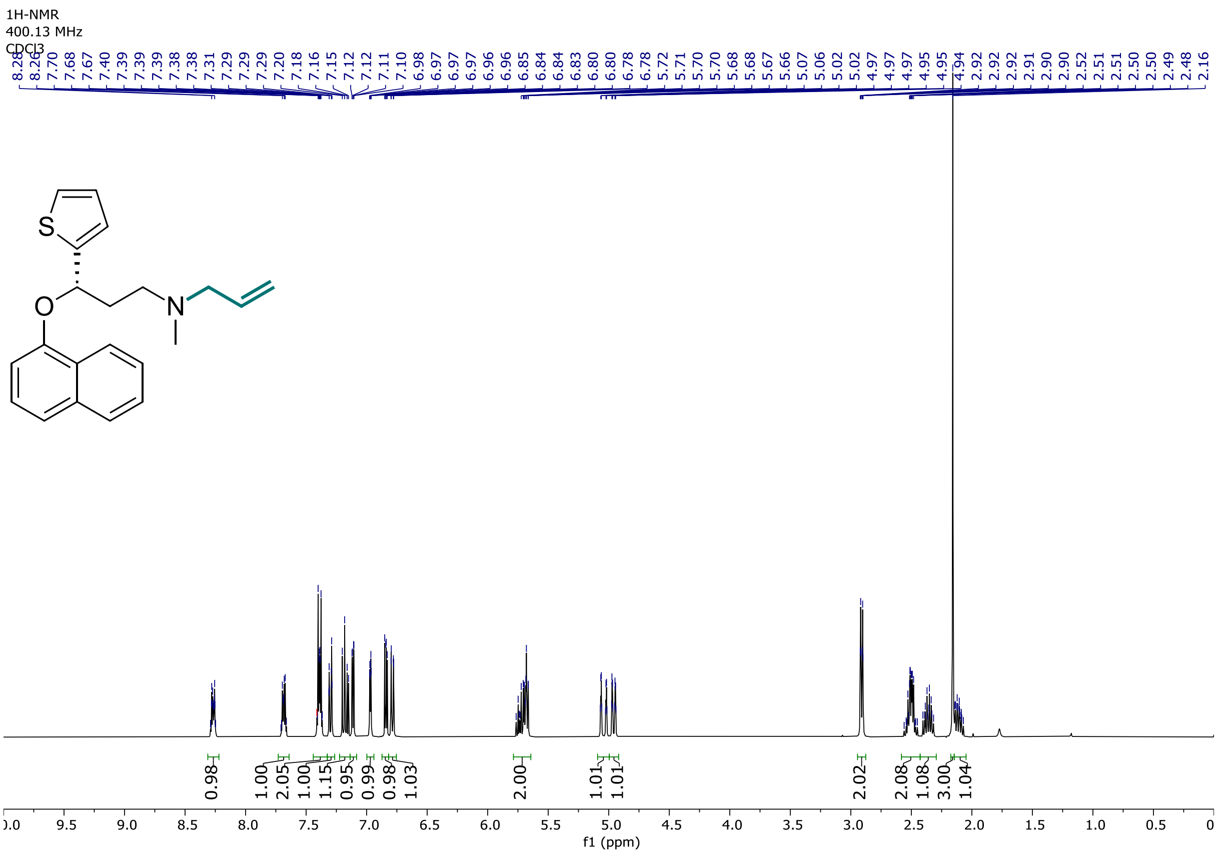

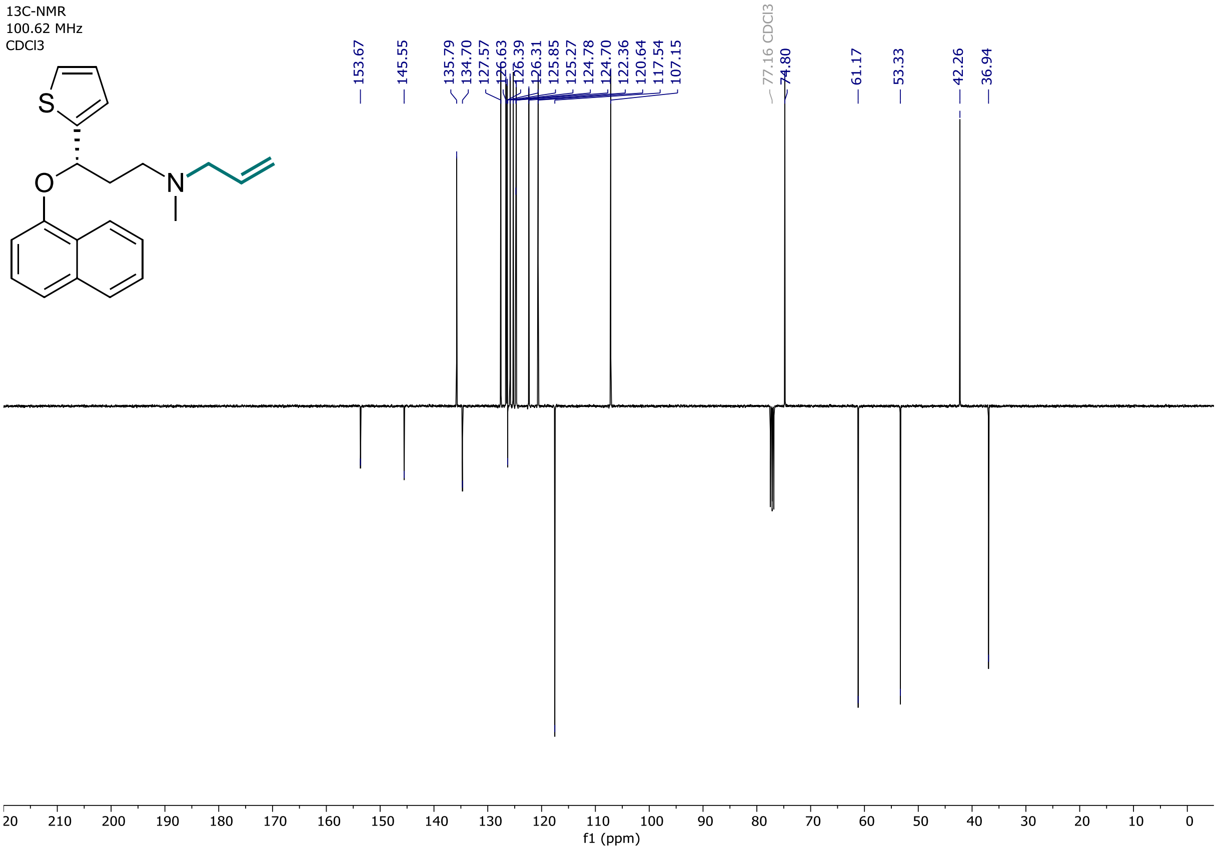

N-Allyl paroxetine **(35)**

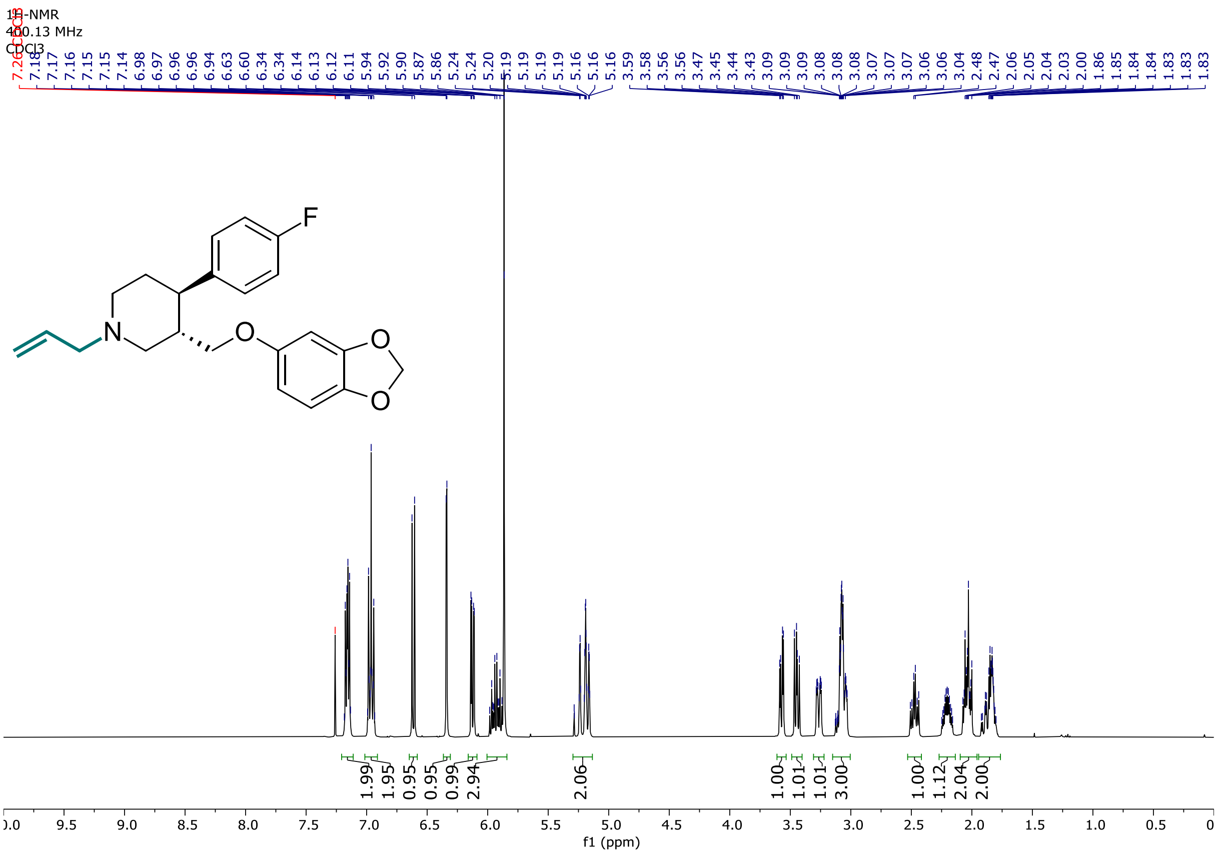

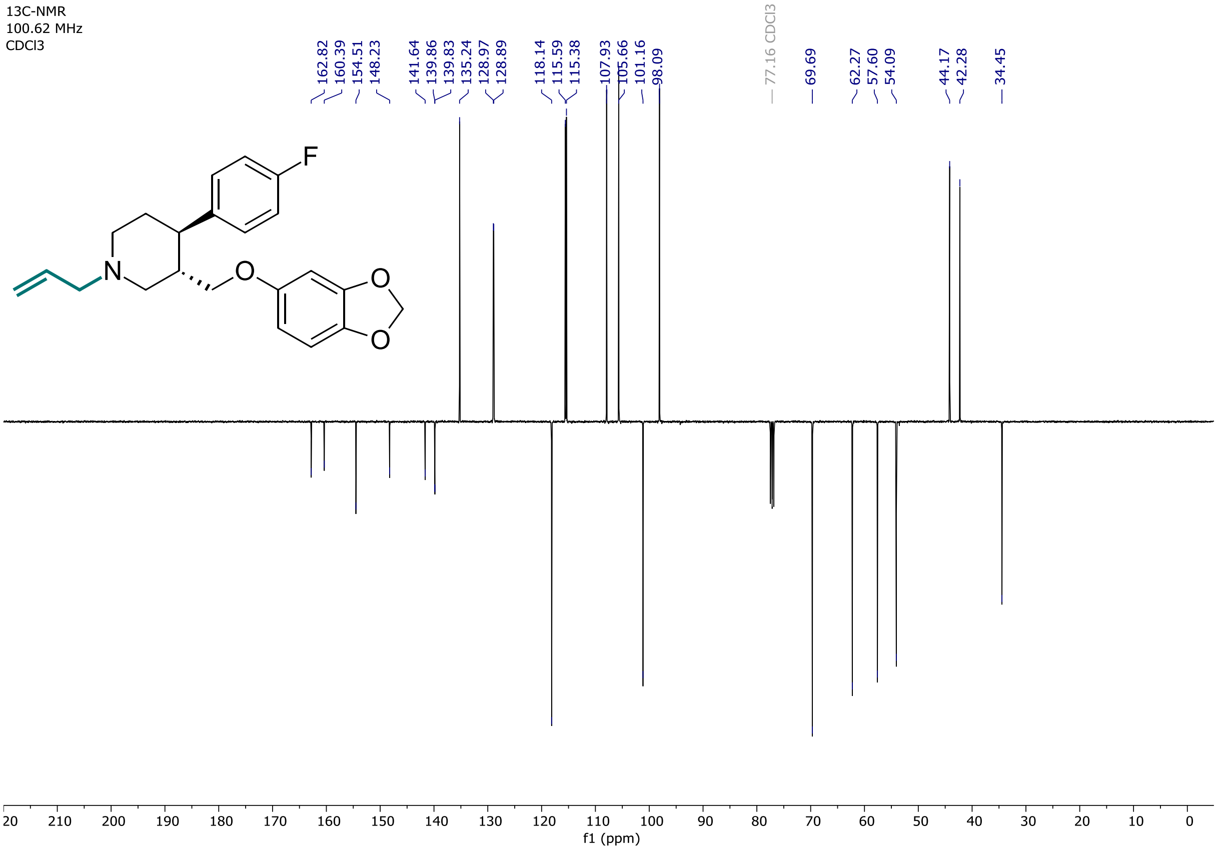

N,N-Diallyl celecoxib **(36)**

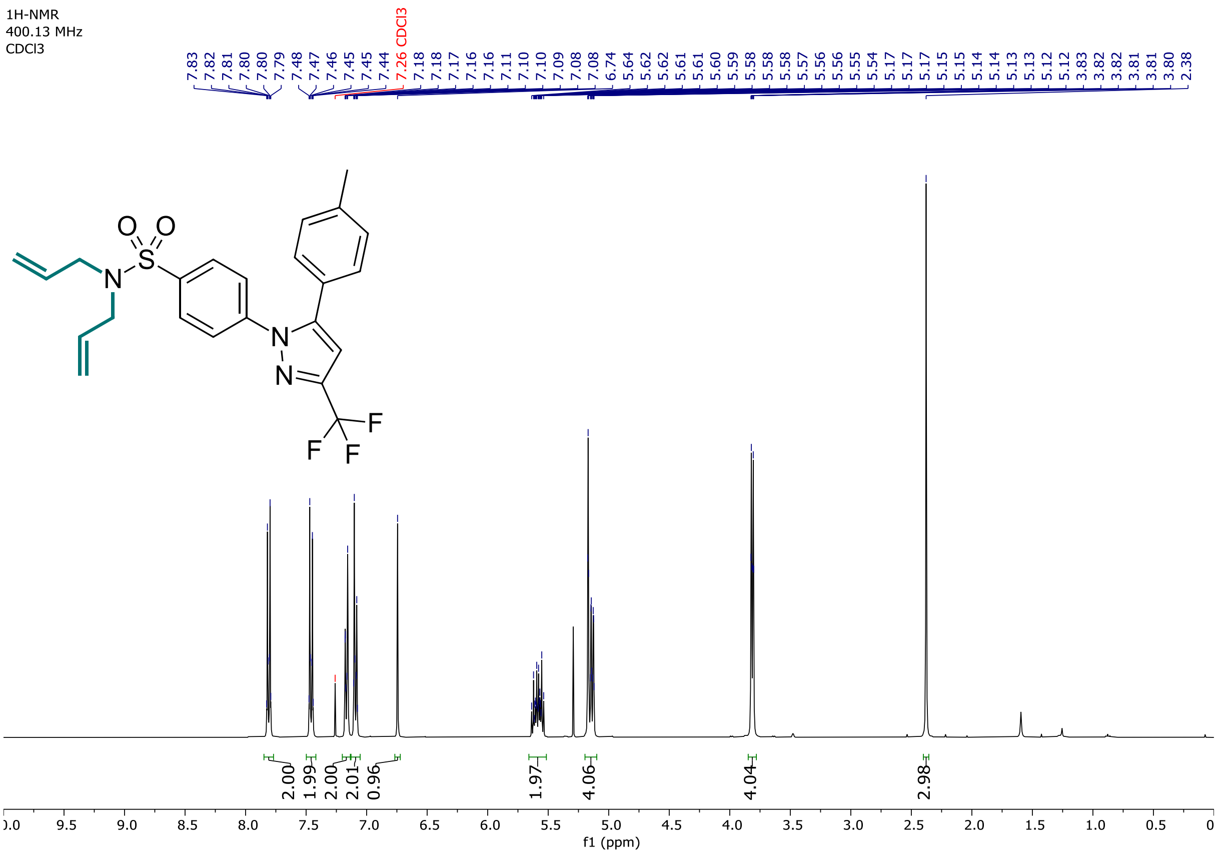

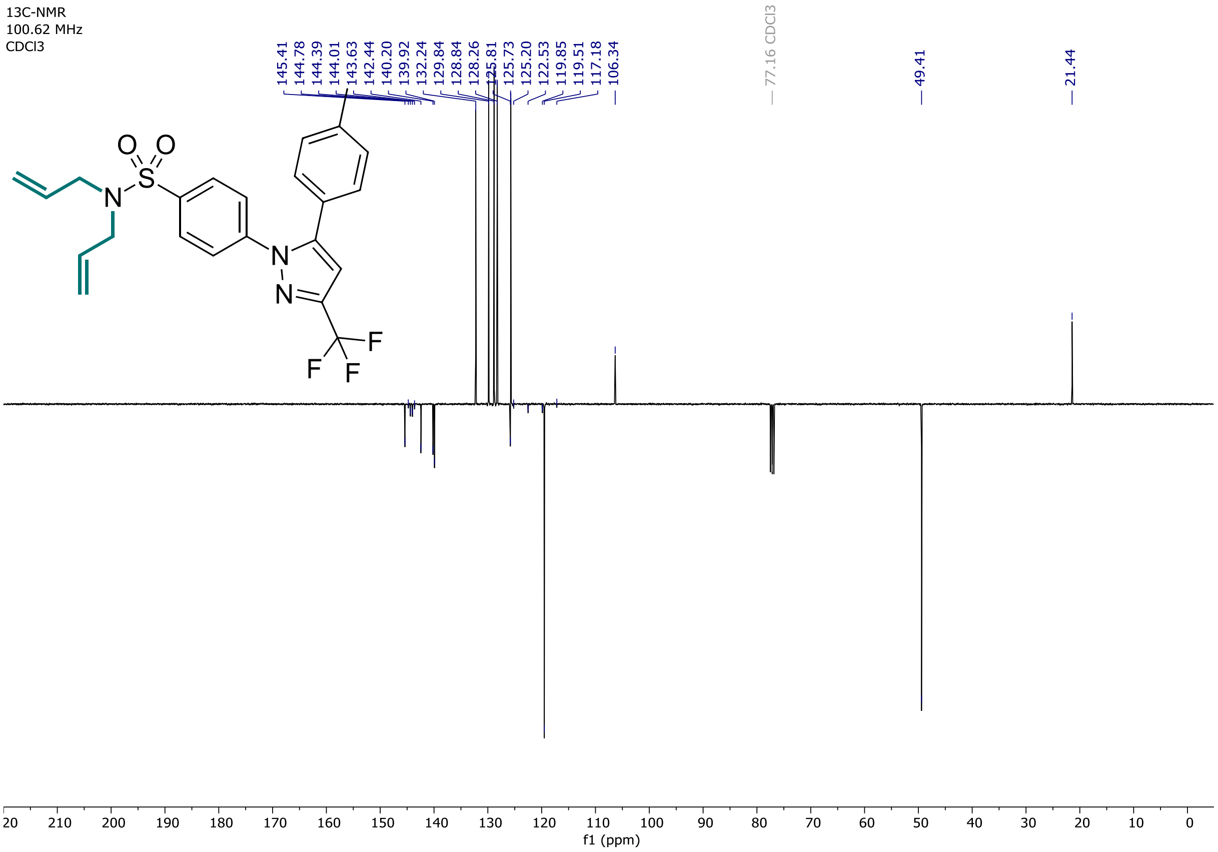

19F-NMR
376.46 MHz
CDCl3

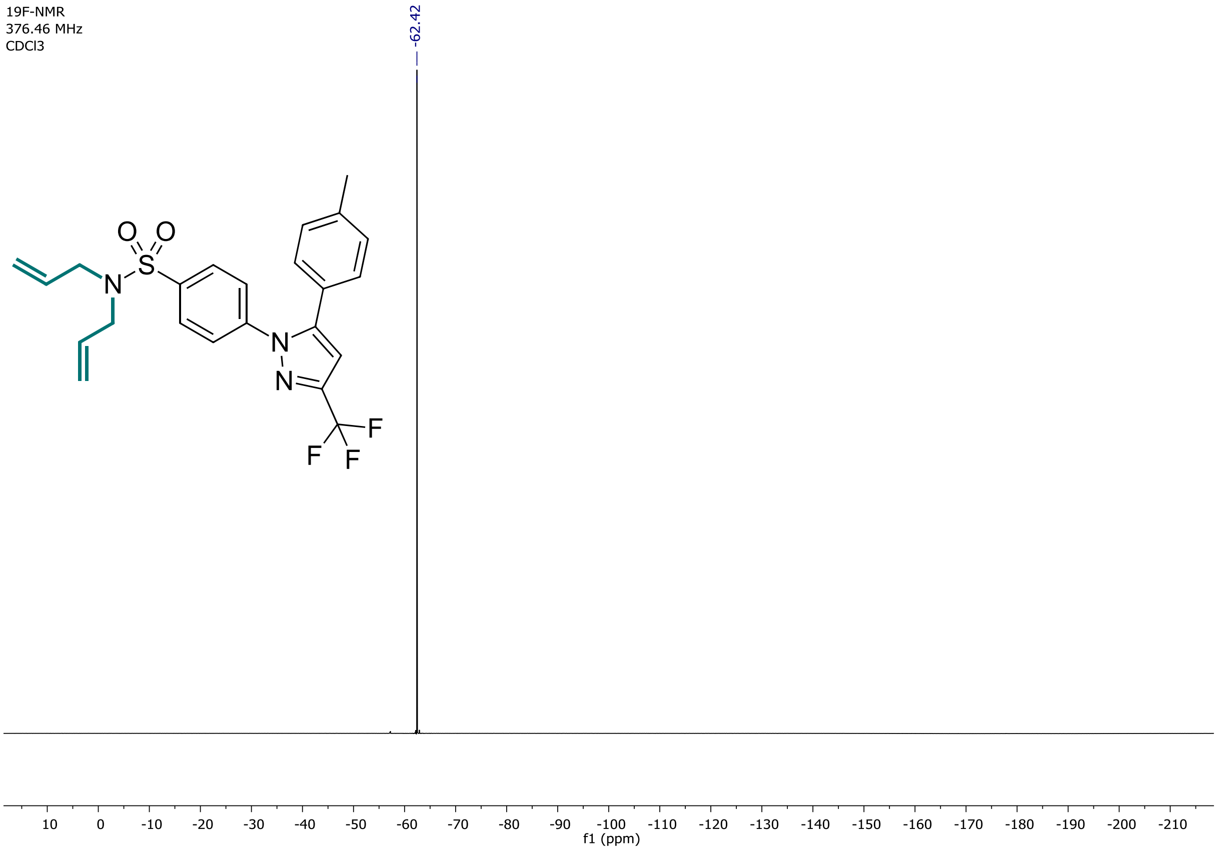

2,2-Diallylmalononitrile **(37)**

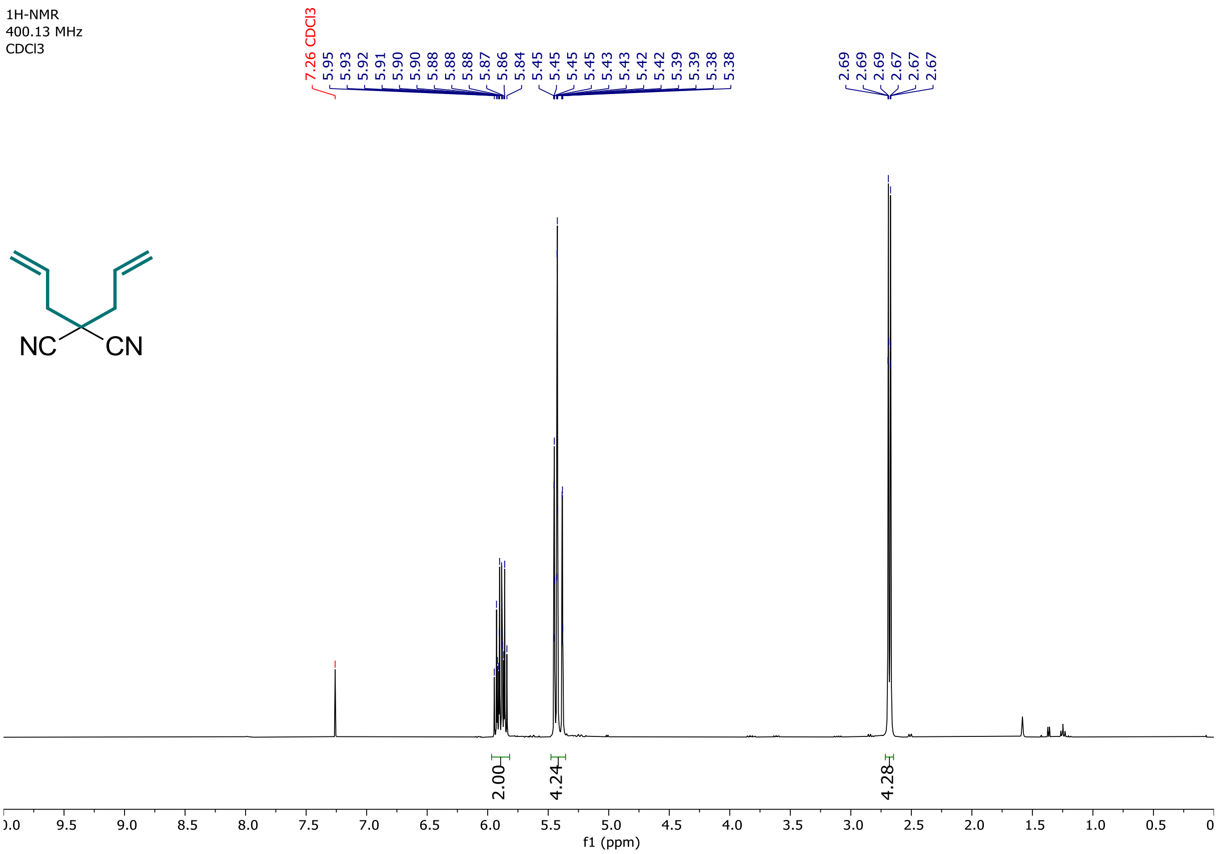

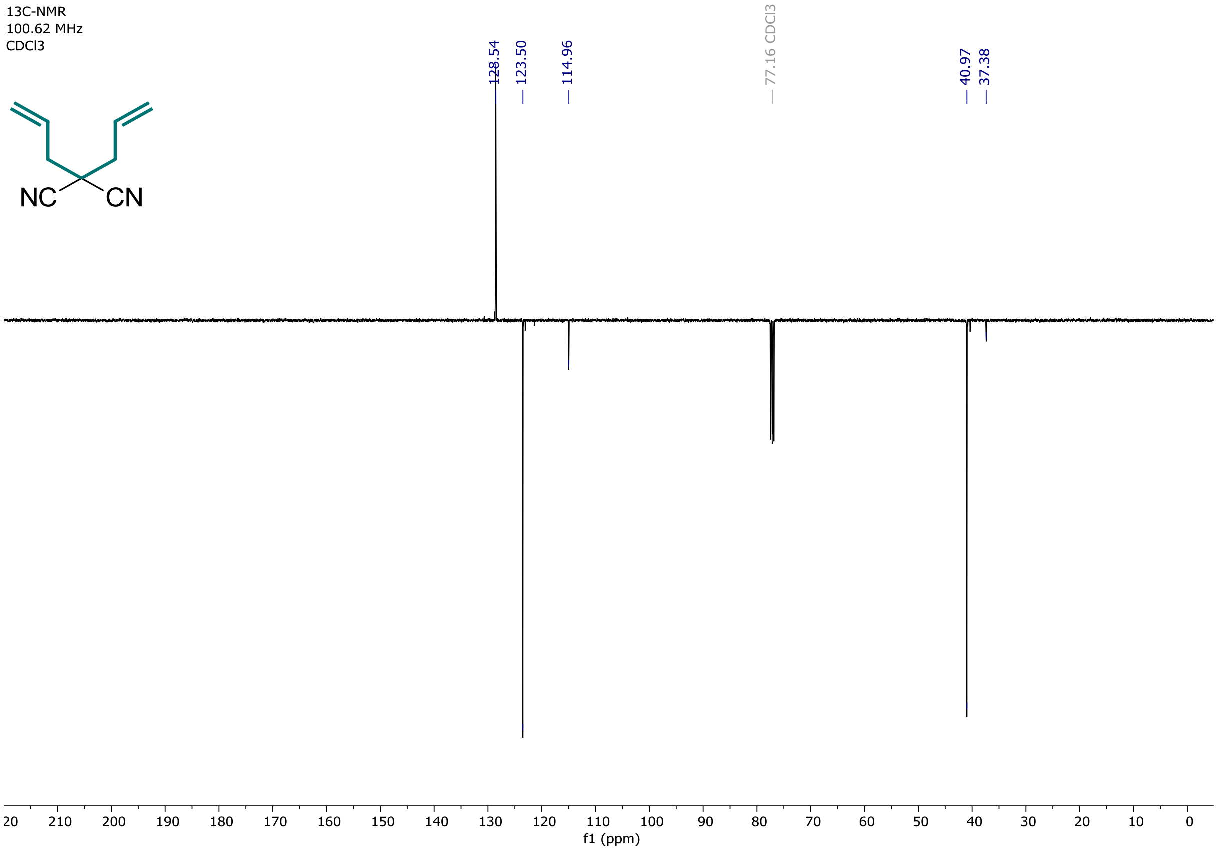

2-(Isopropenylcarbonyloxy)ethyl 2-acetyl-4-pentenoate **(38)**

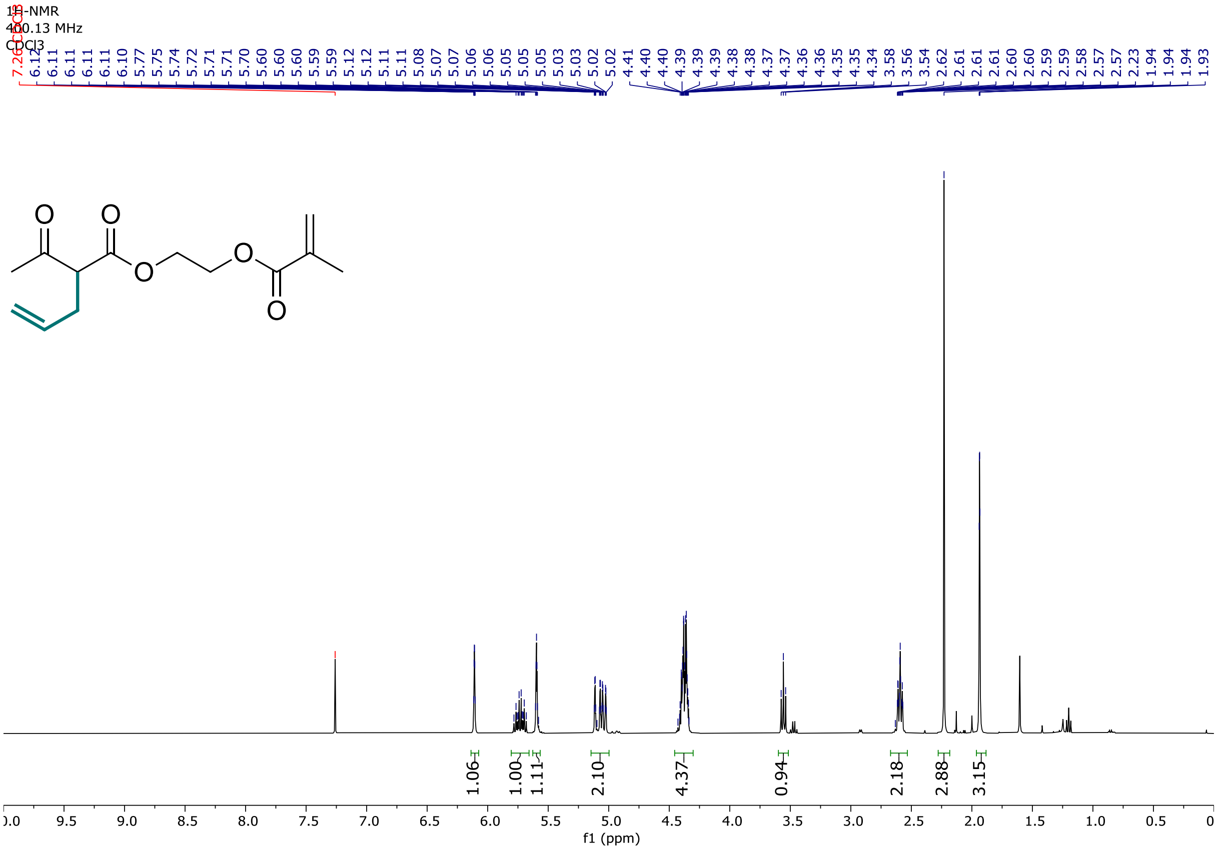

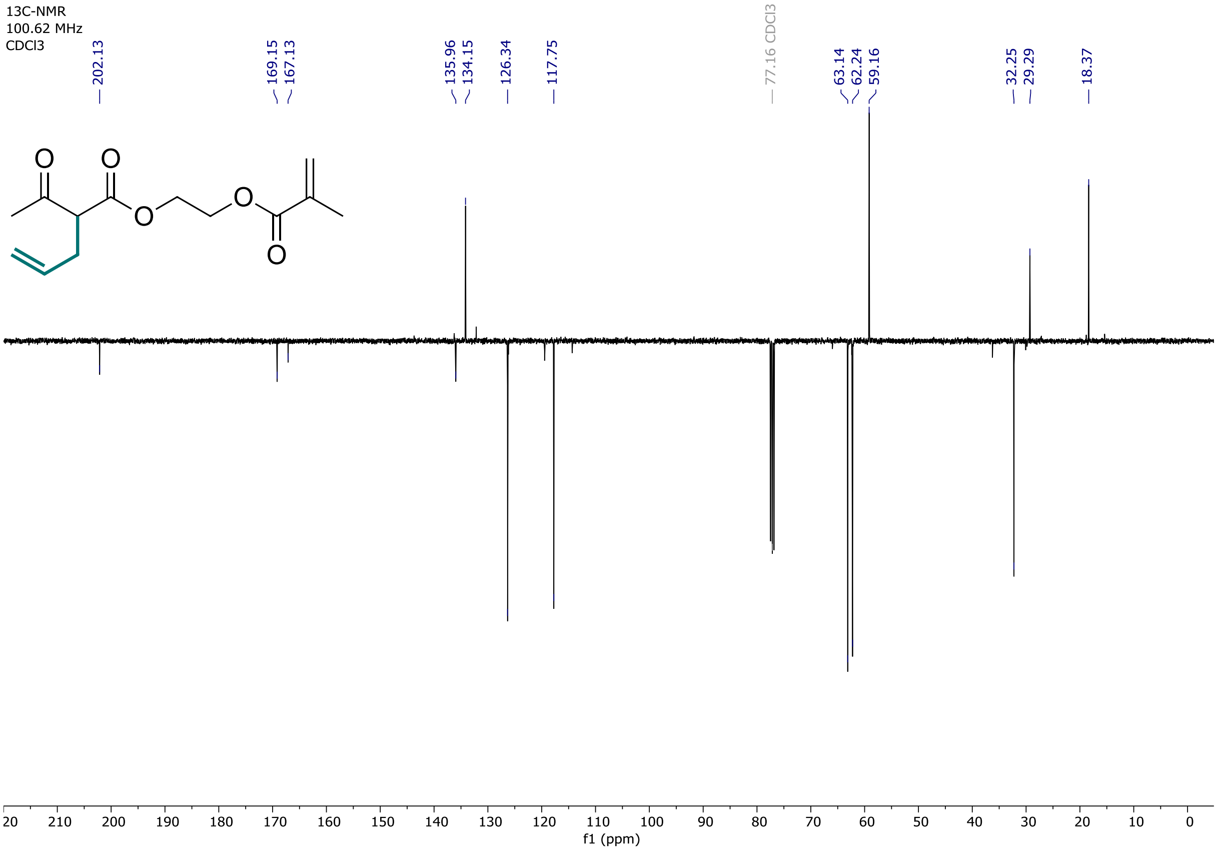

Ethyl 2-benzoylpent-4-enoate **(39)**

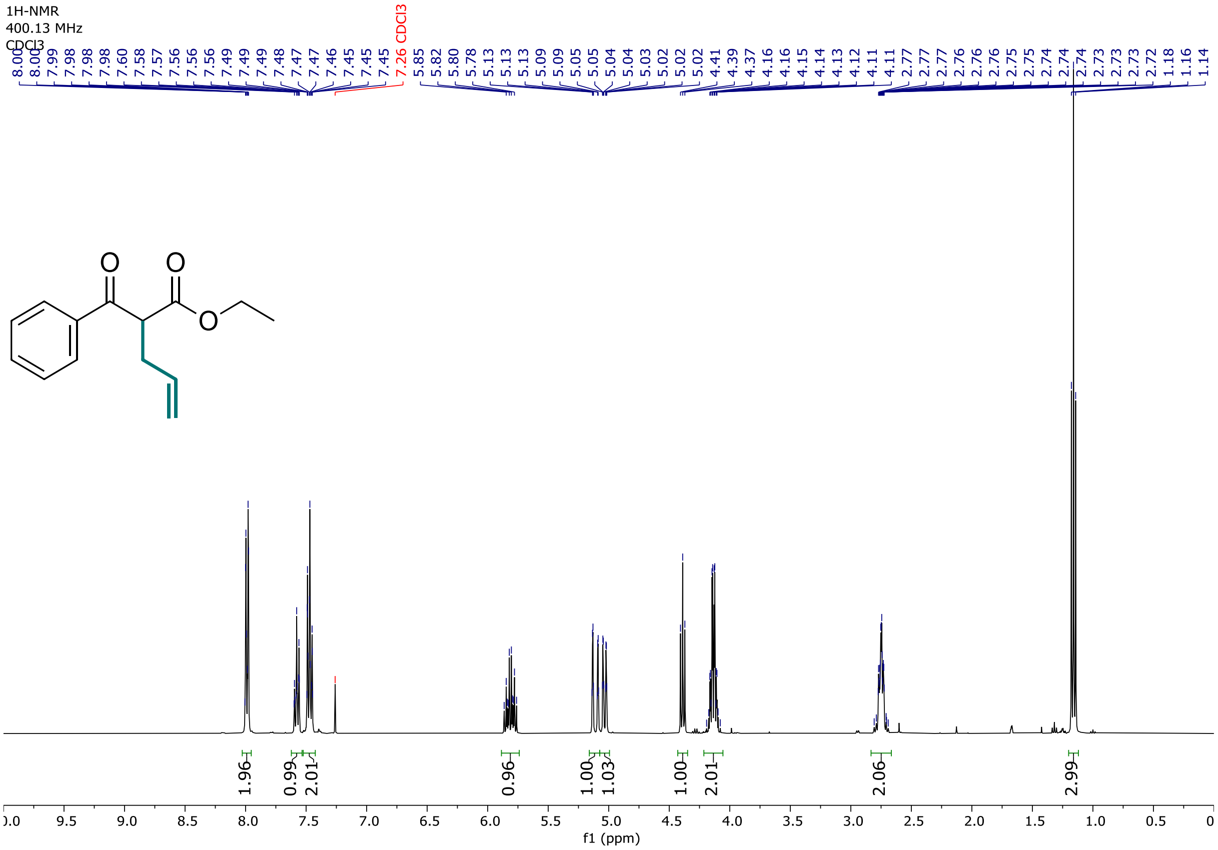

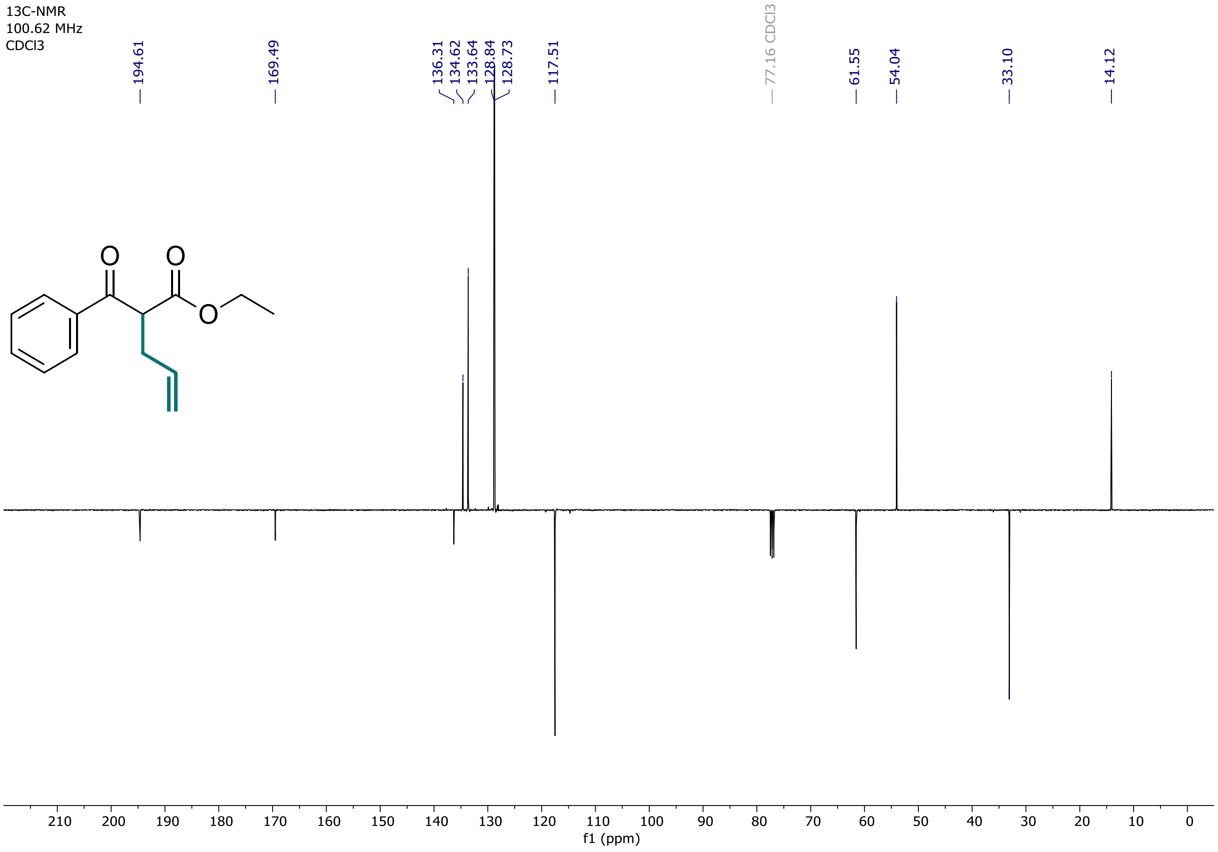

4-Methyl-4-nitro-1-pentene **(40)**

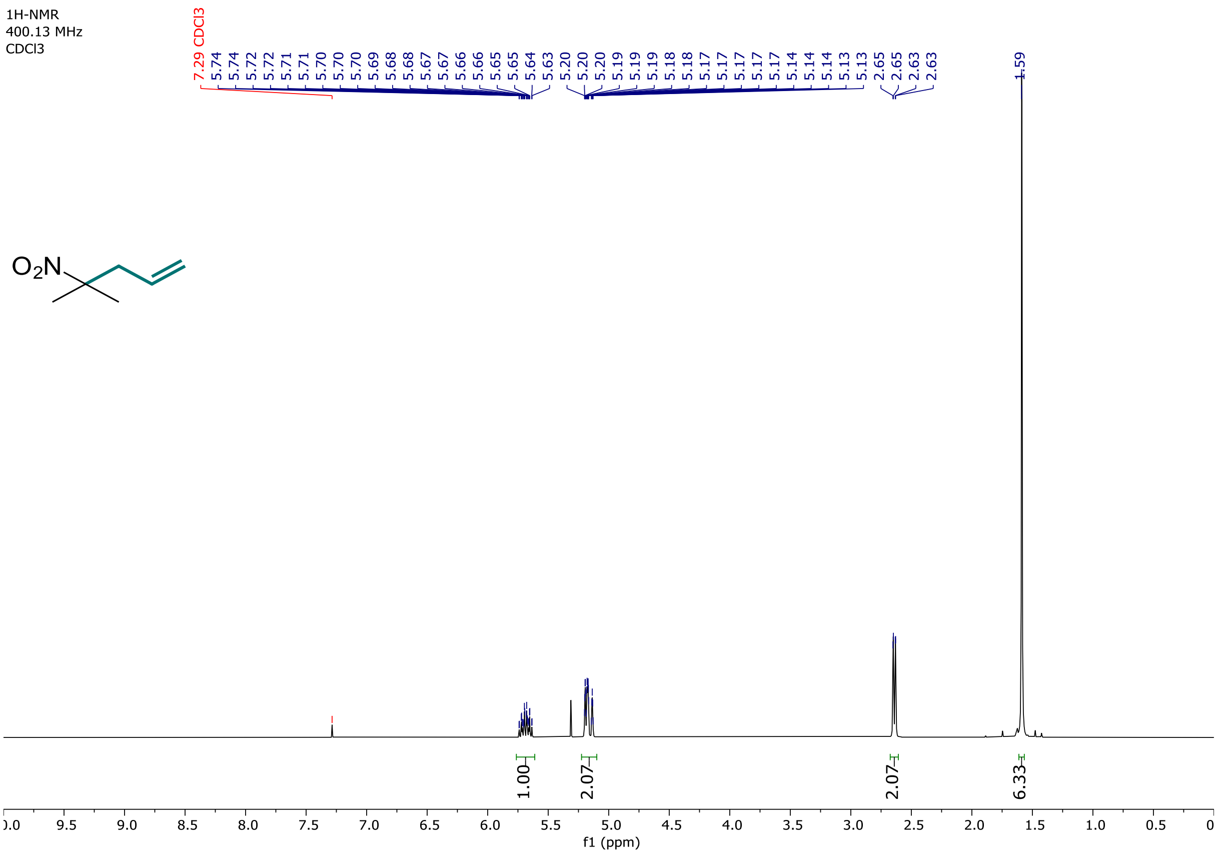

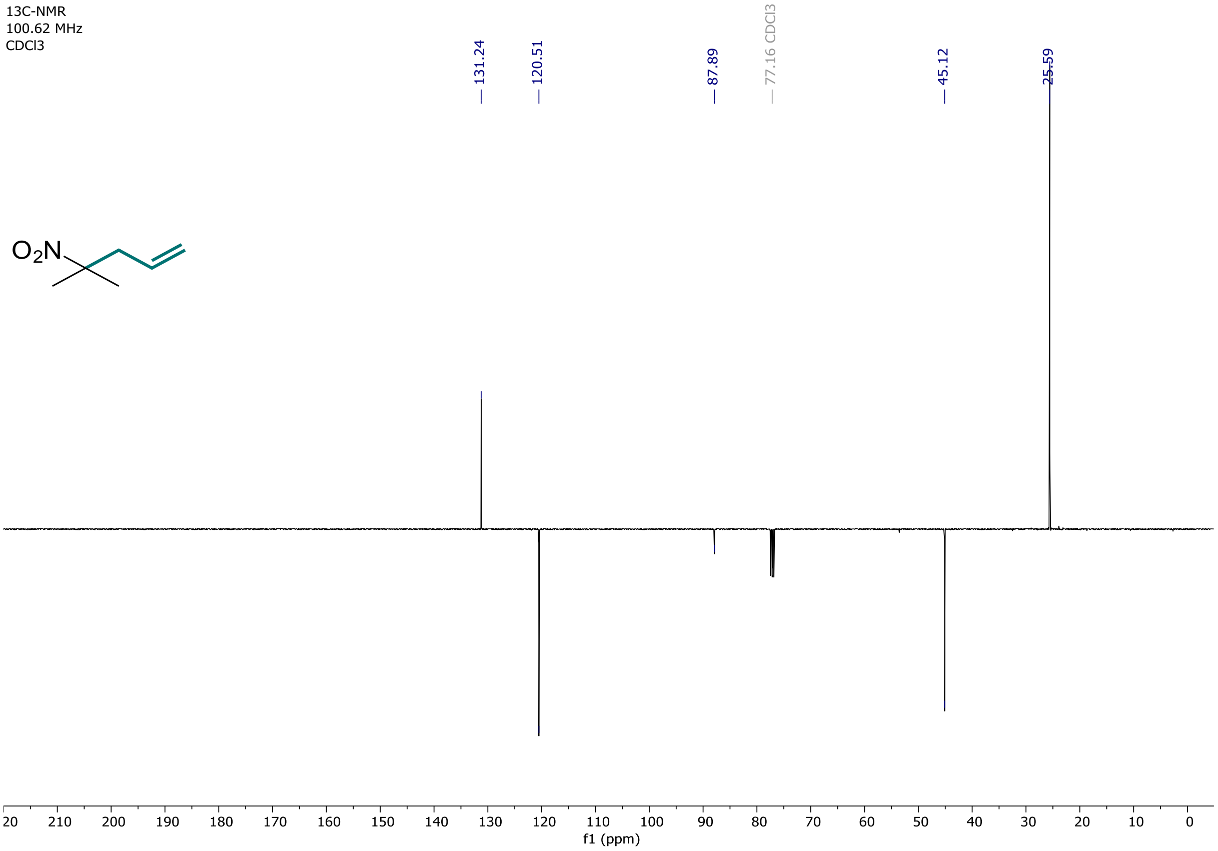

tert-Butyl 2-allyl-2-cyano-4-pentenoate **(41)**

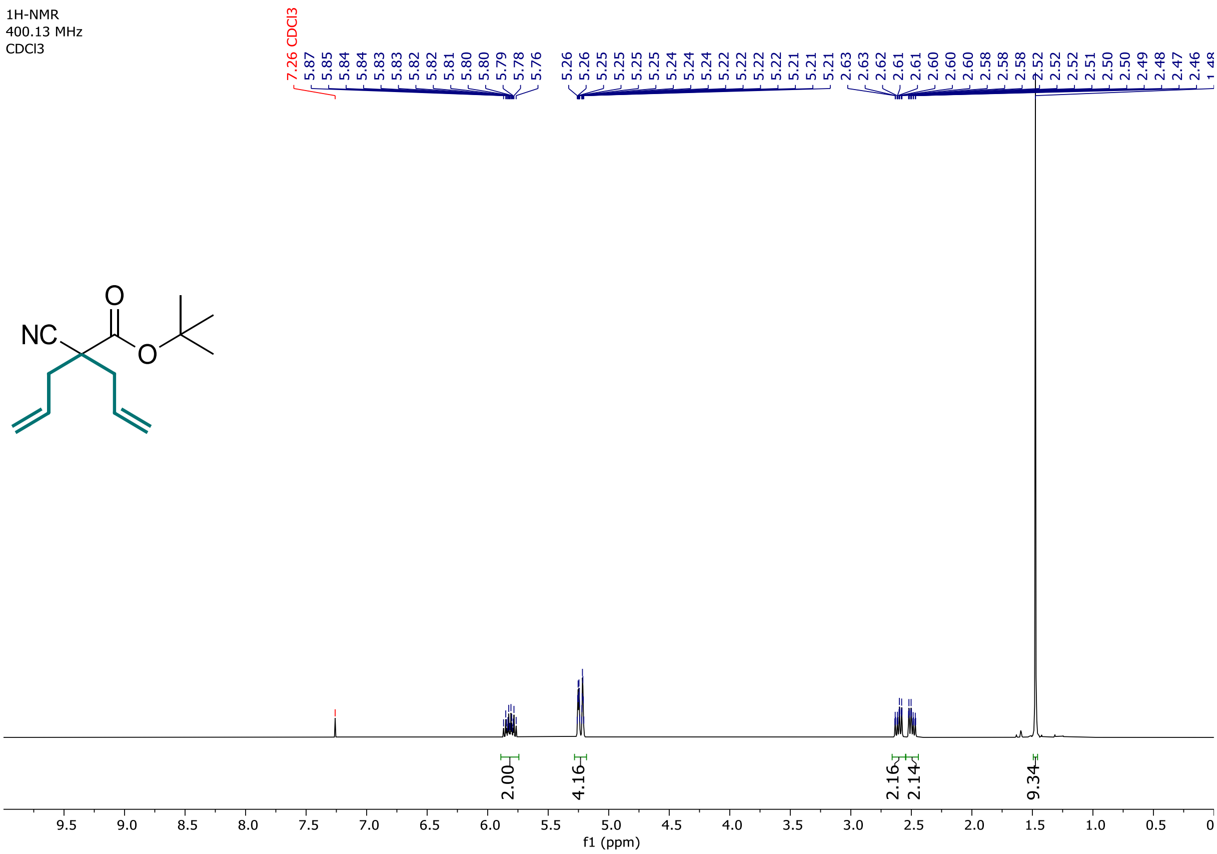

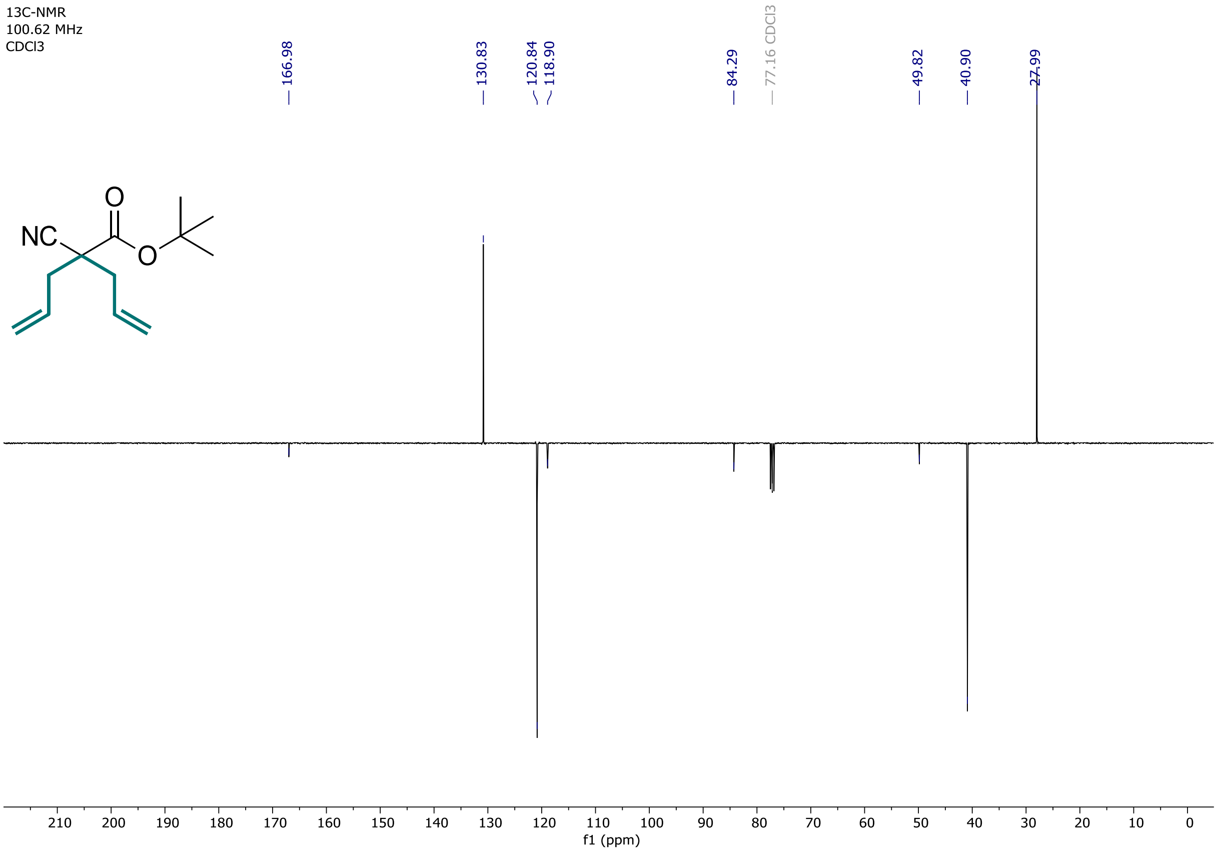

3-(4-Biphenylyloxy)cyclohexene **(42)**

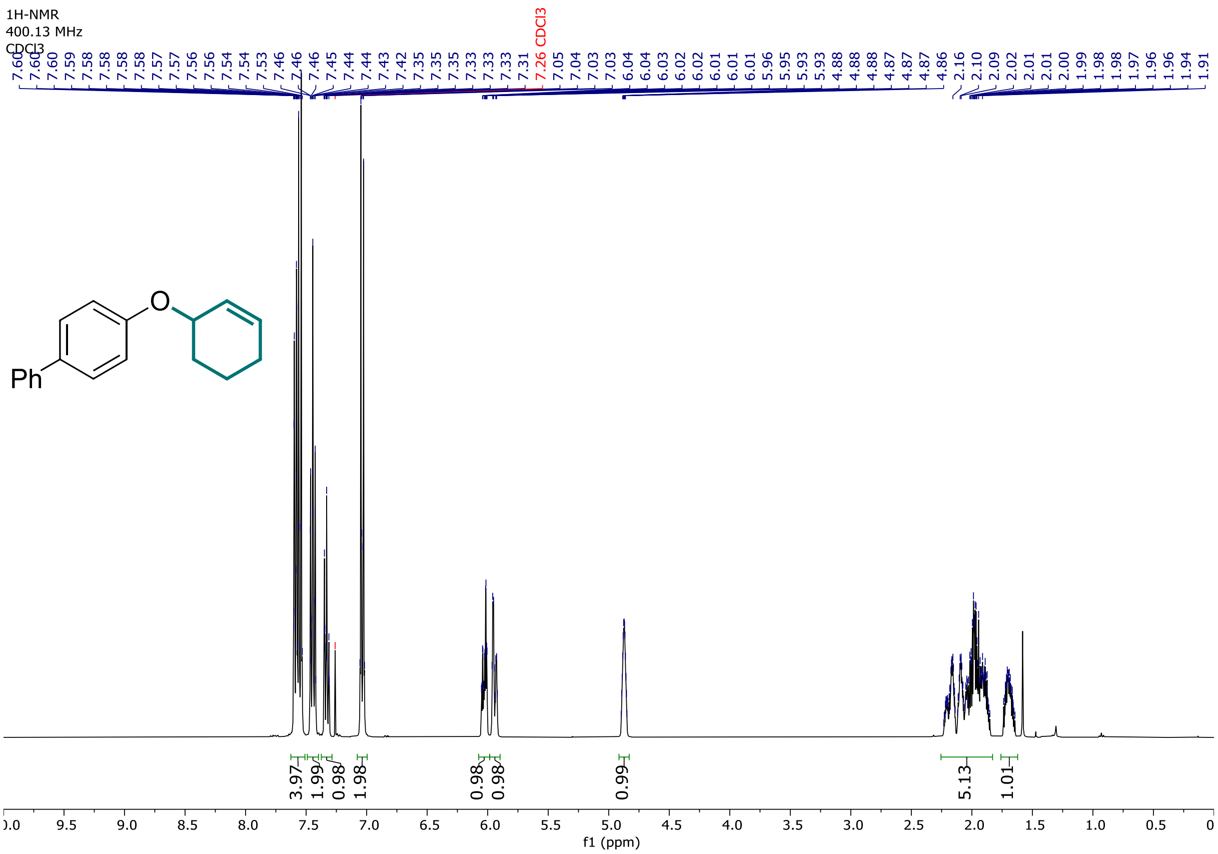

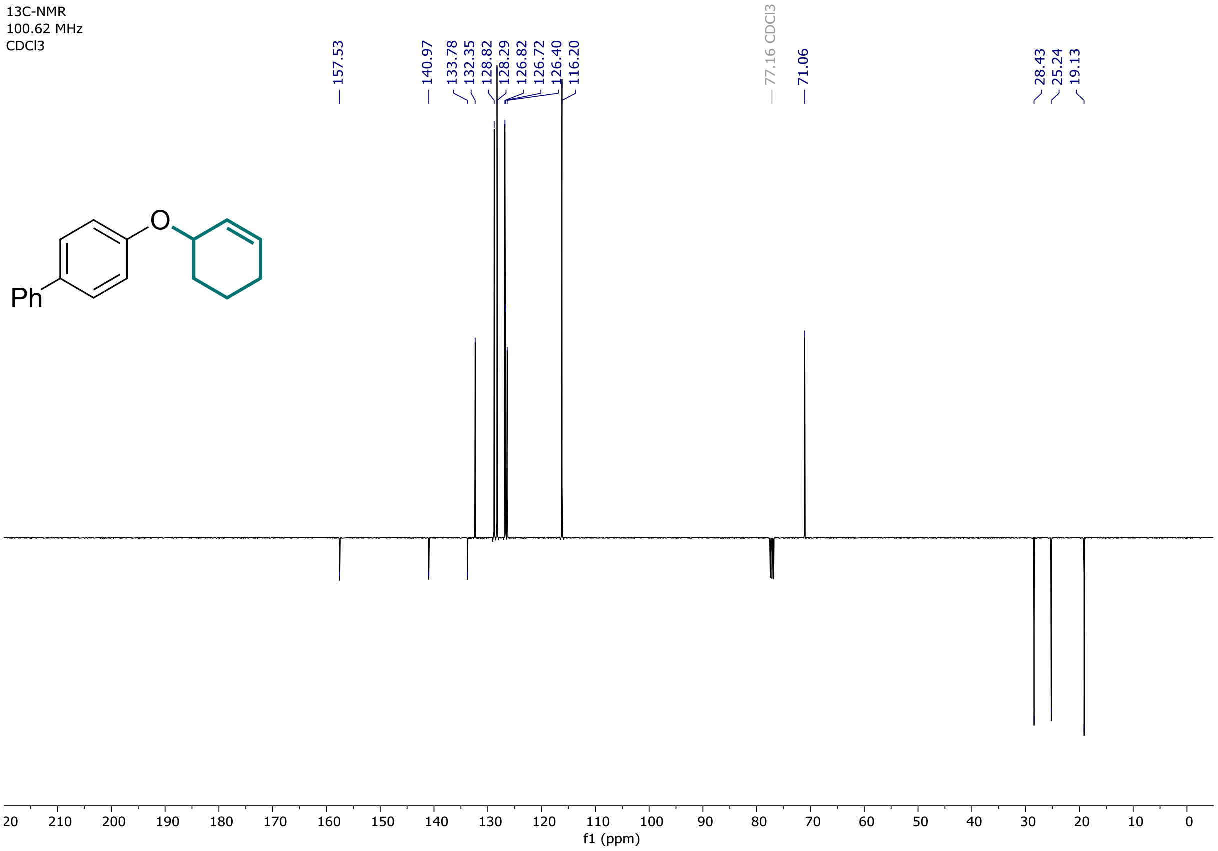

p-(2-Cyclohexen-1-yloxy)methoxybenzene **(43)**

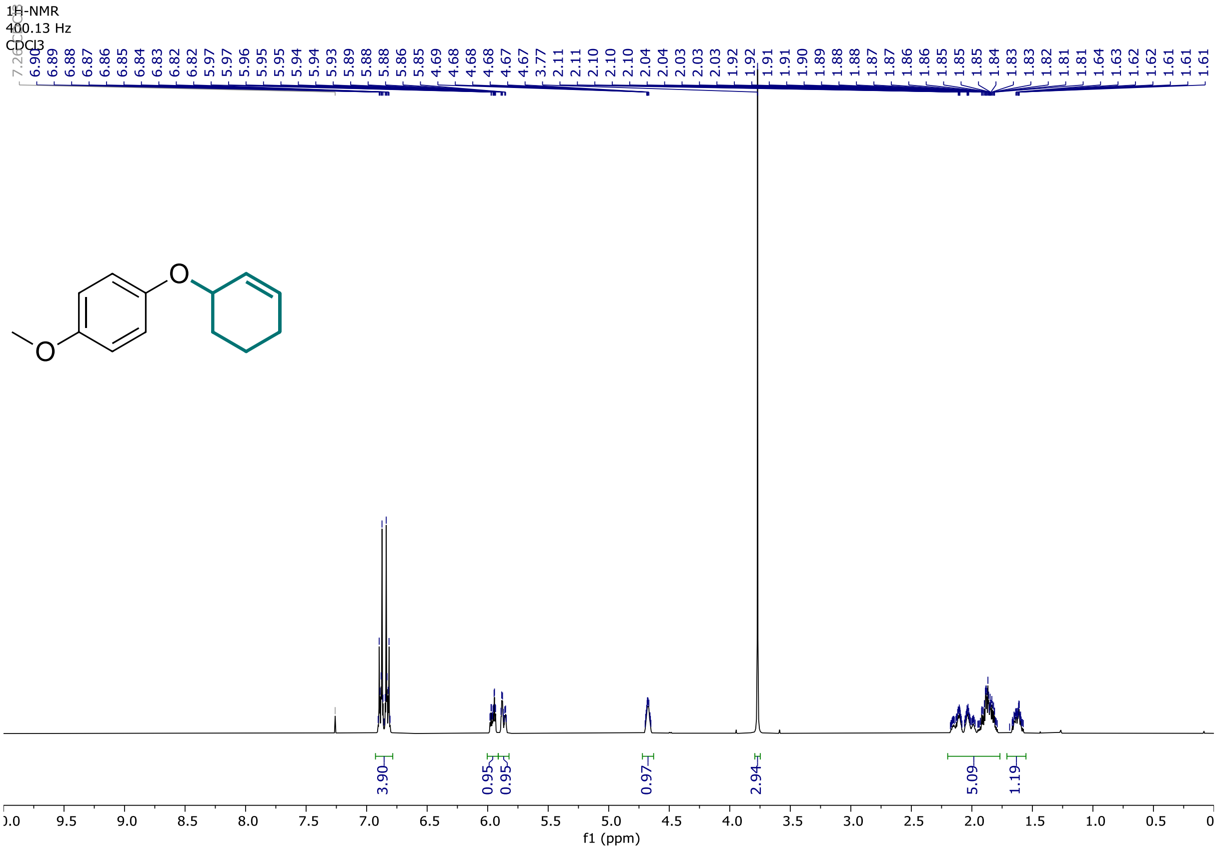

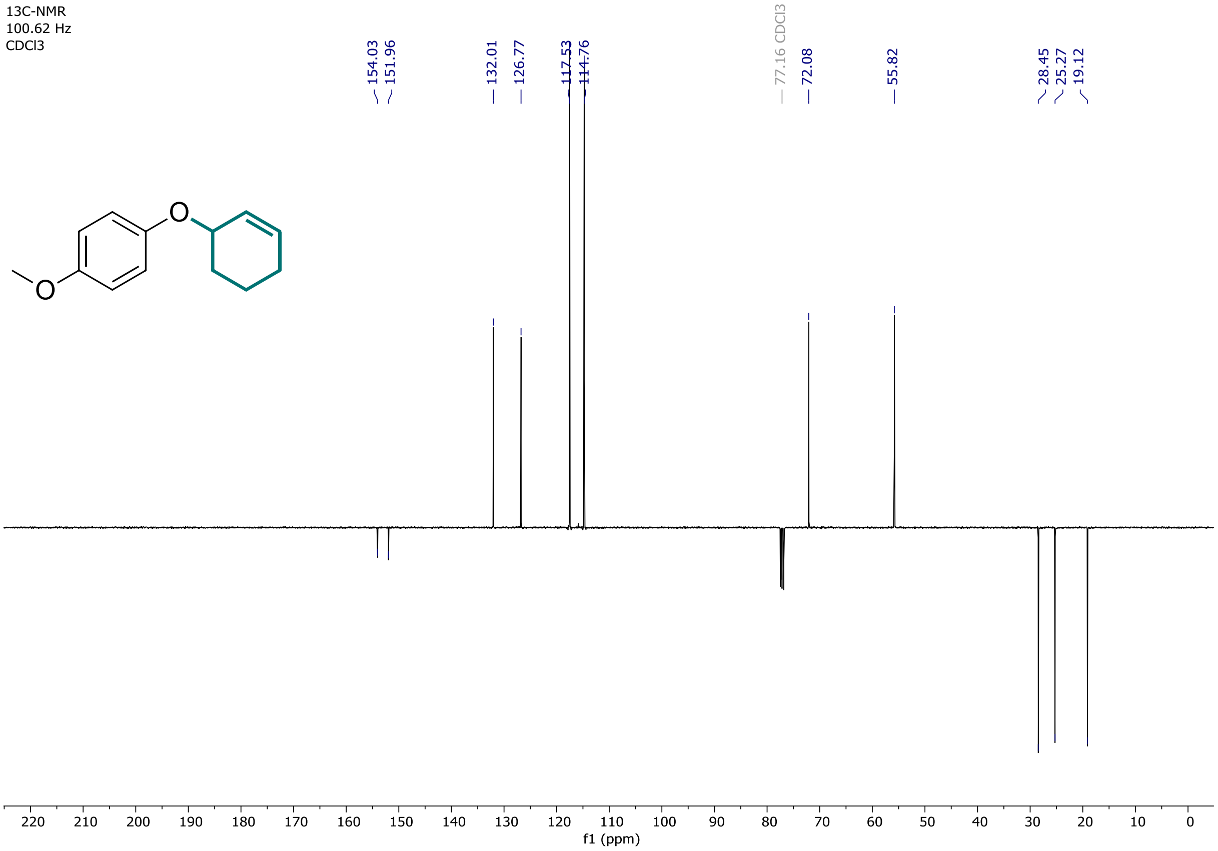

(E)-Cinnamyl 1,1'-biphenyl ether **(44)**

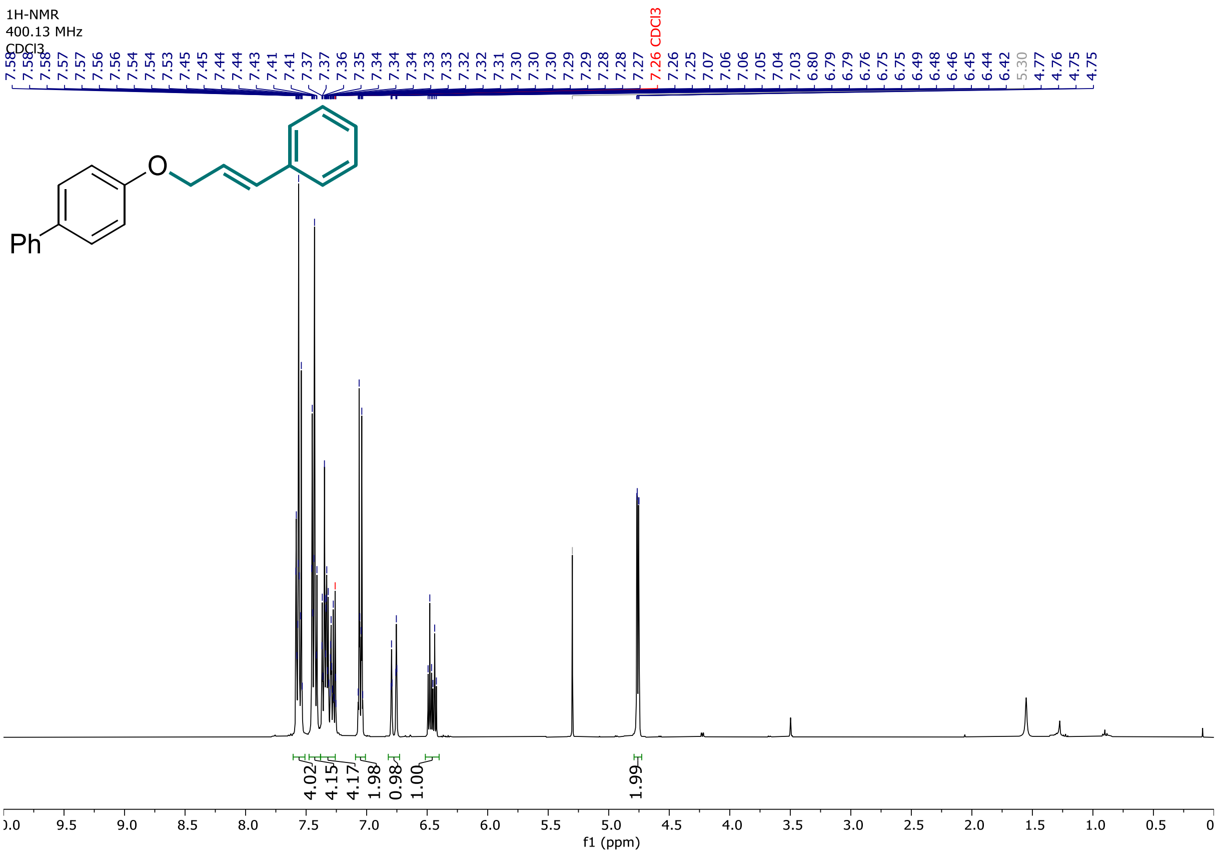

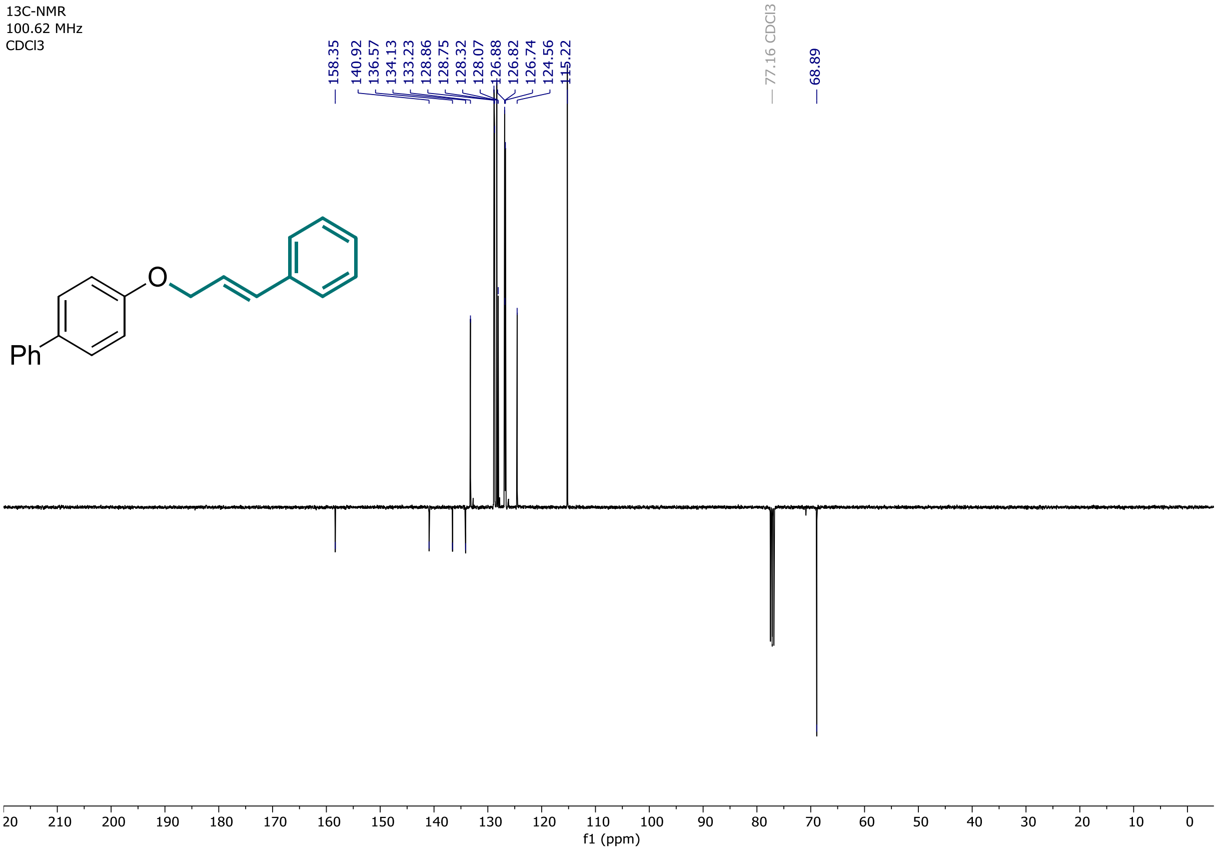

4-[(E)-6,6-Dimethyl-2-hepten-4-ynyloxy]biphenyl **(45)**

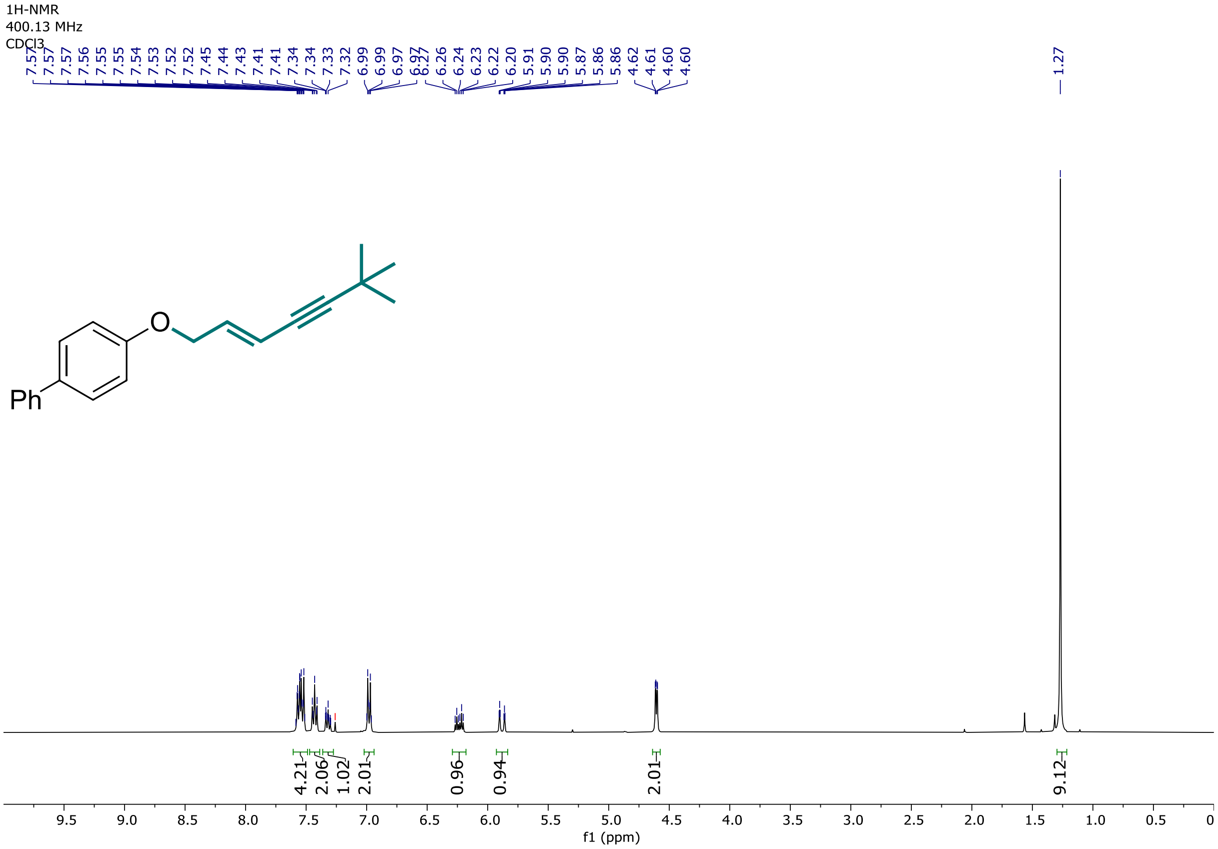

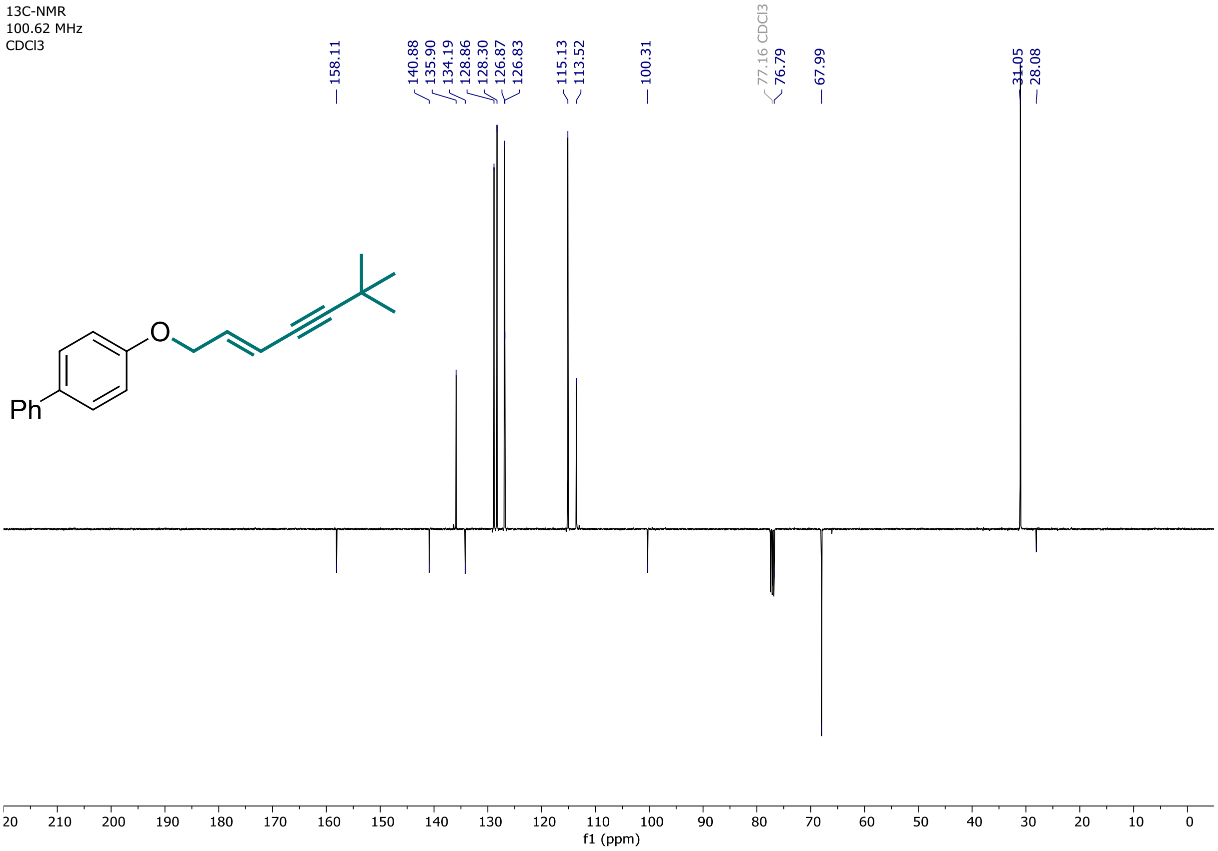

4-biphenylyl 2,4-pentadienyl ether **(46)**

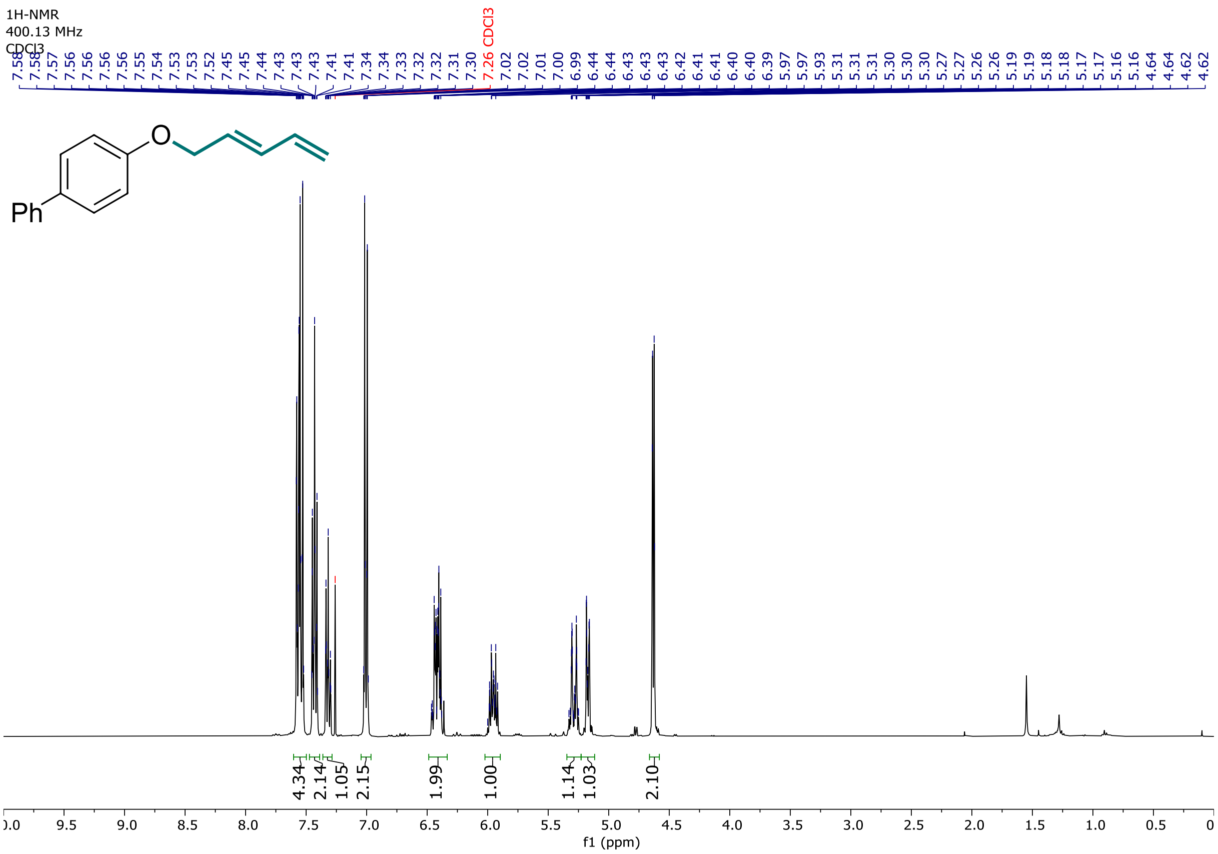

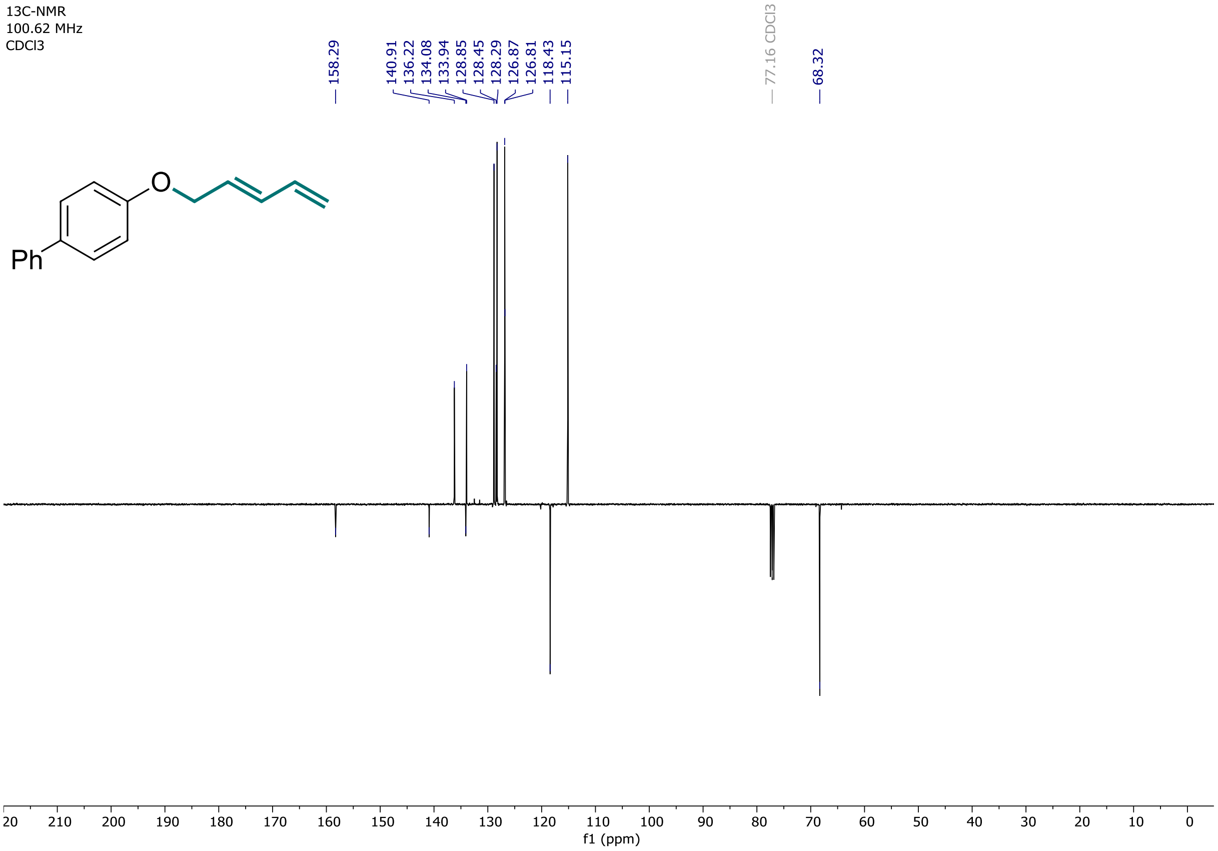

4-[(E)-1-Methyl-2-butenyloxy]biphenyl **(47)**

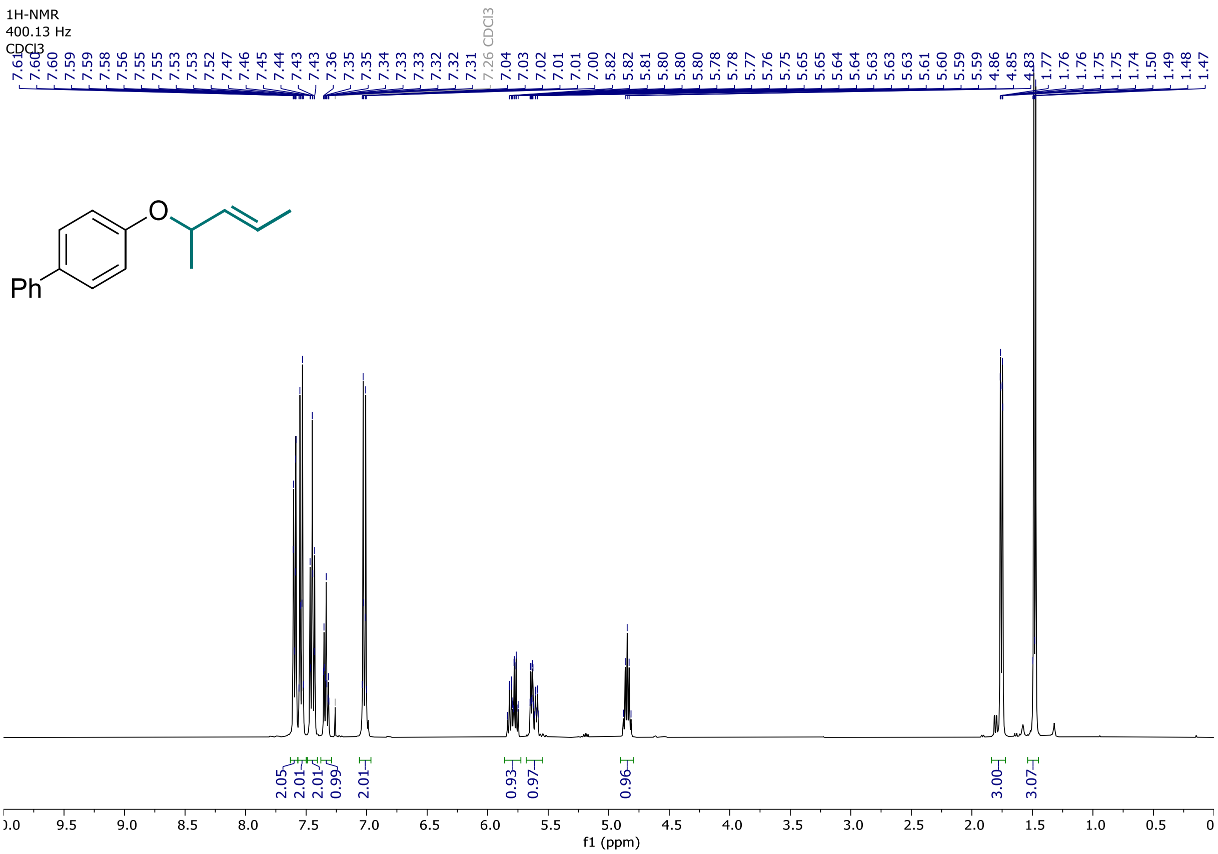

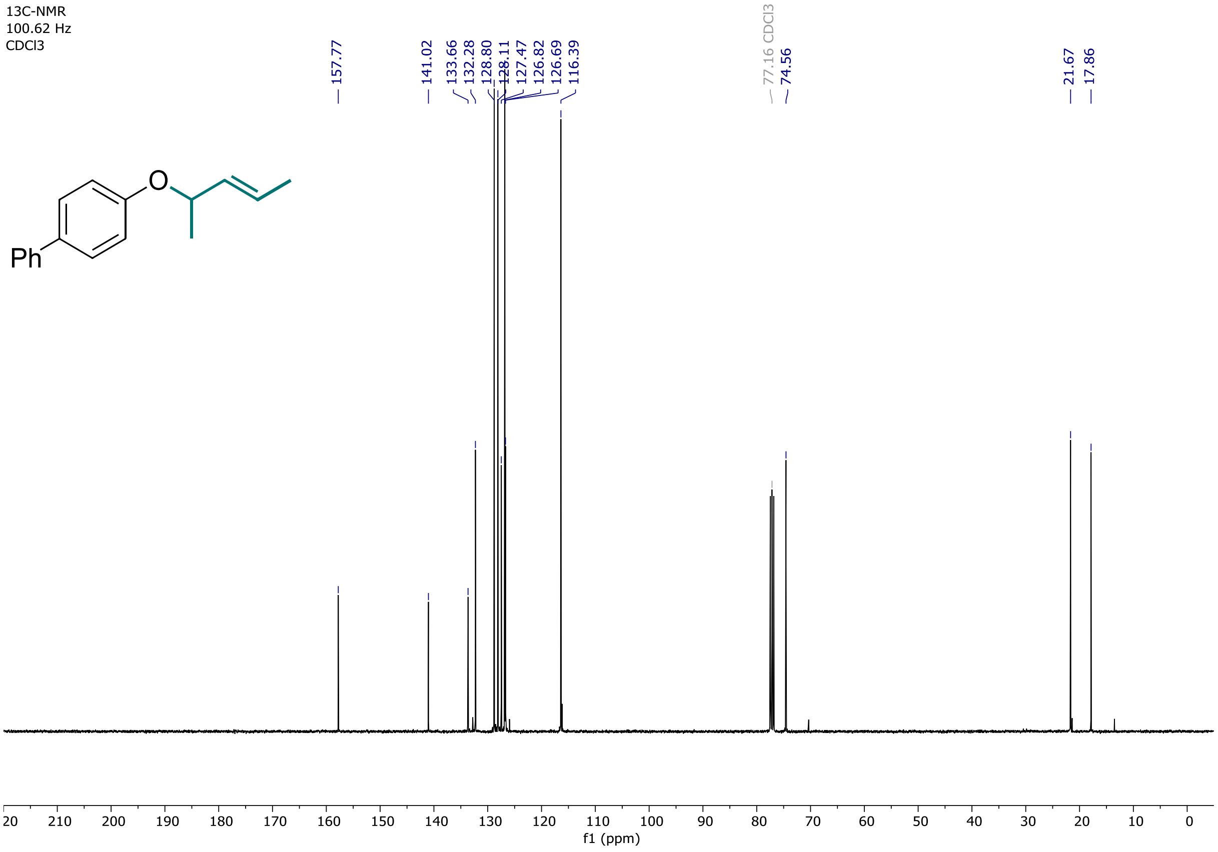

N-2-Cyclohexen-1-yl-N-methyl(p-fluorophenyl)amine **(48)**

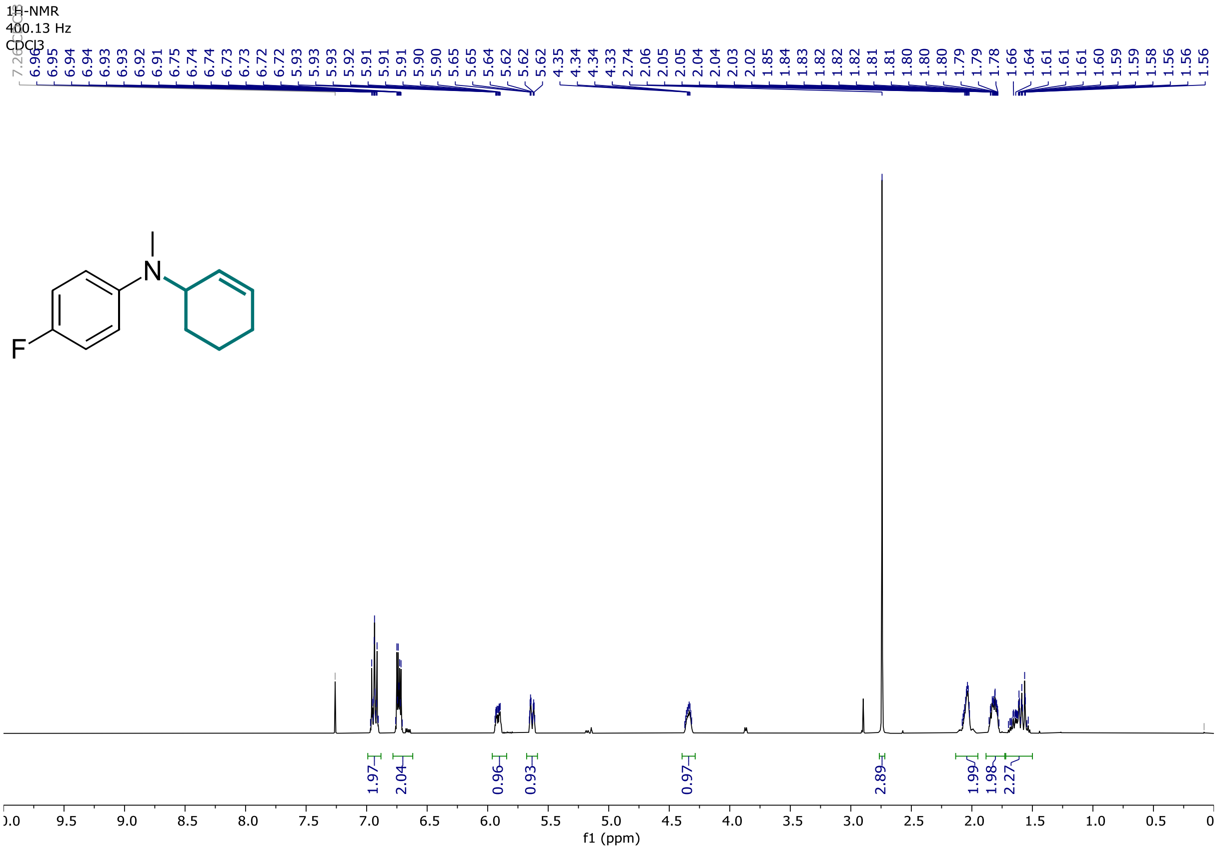

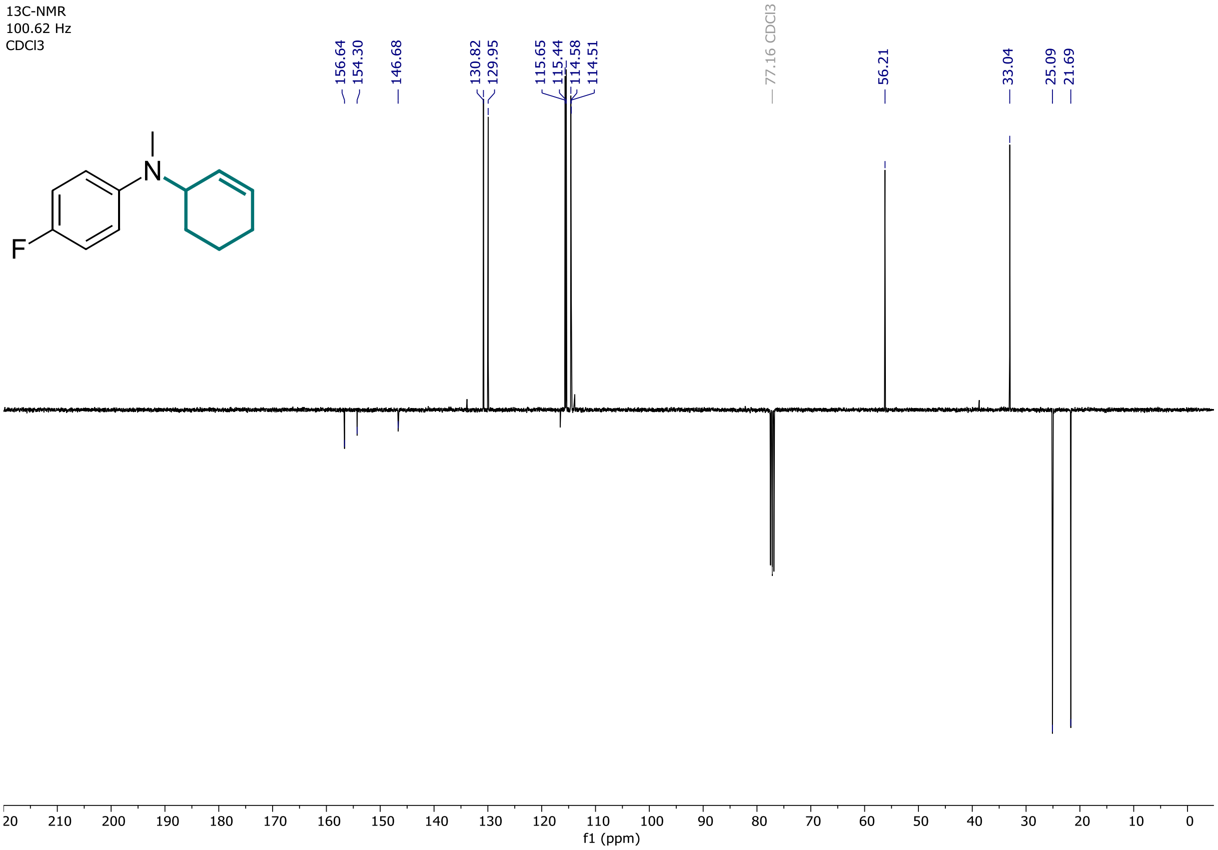

19F-NMR
376.46 Hz
CDCl3

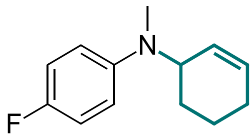

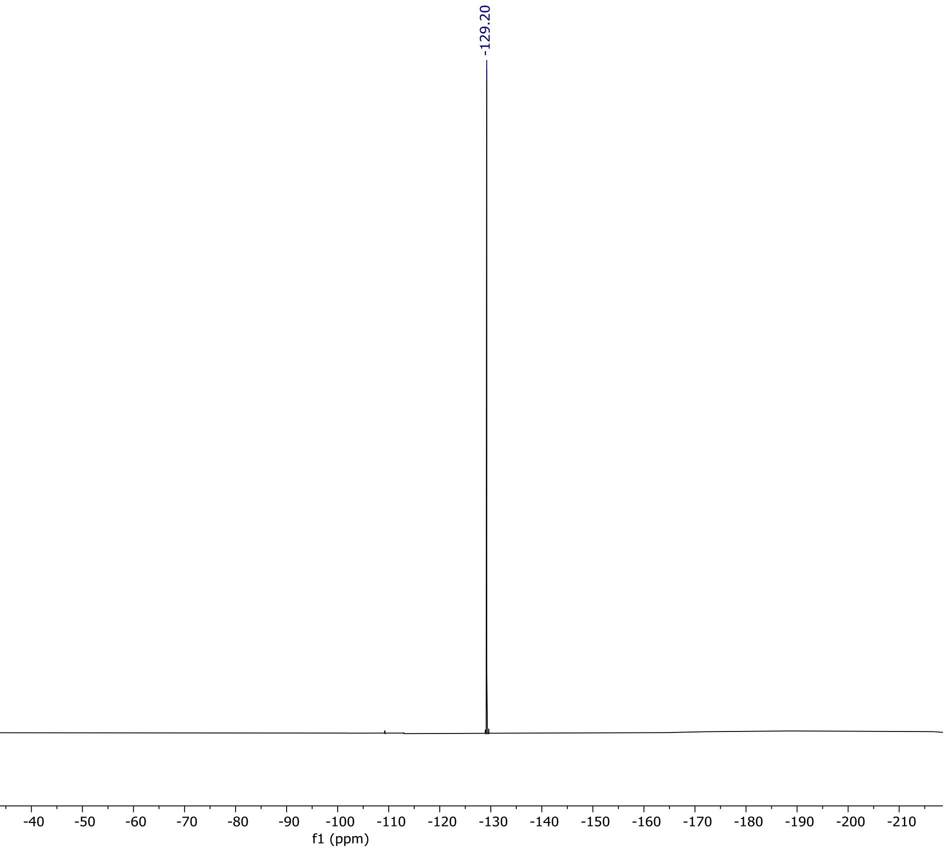

N-Methyl[2-(2-pyridyl)ethyl][(E)-3-phenyl-2-propenyl]amine **(49)**

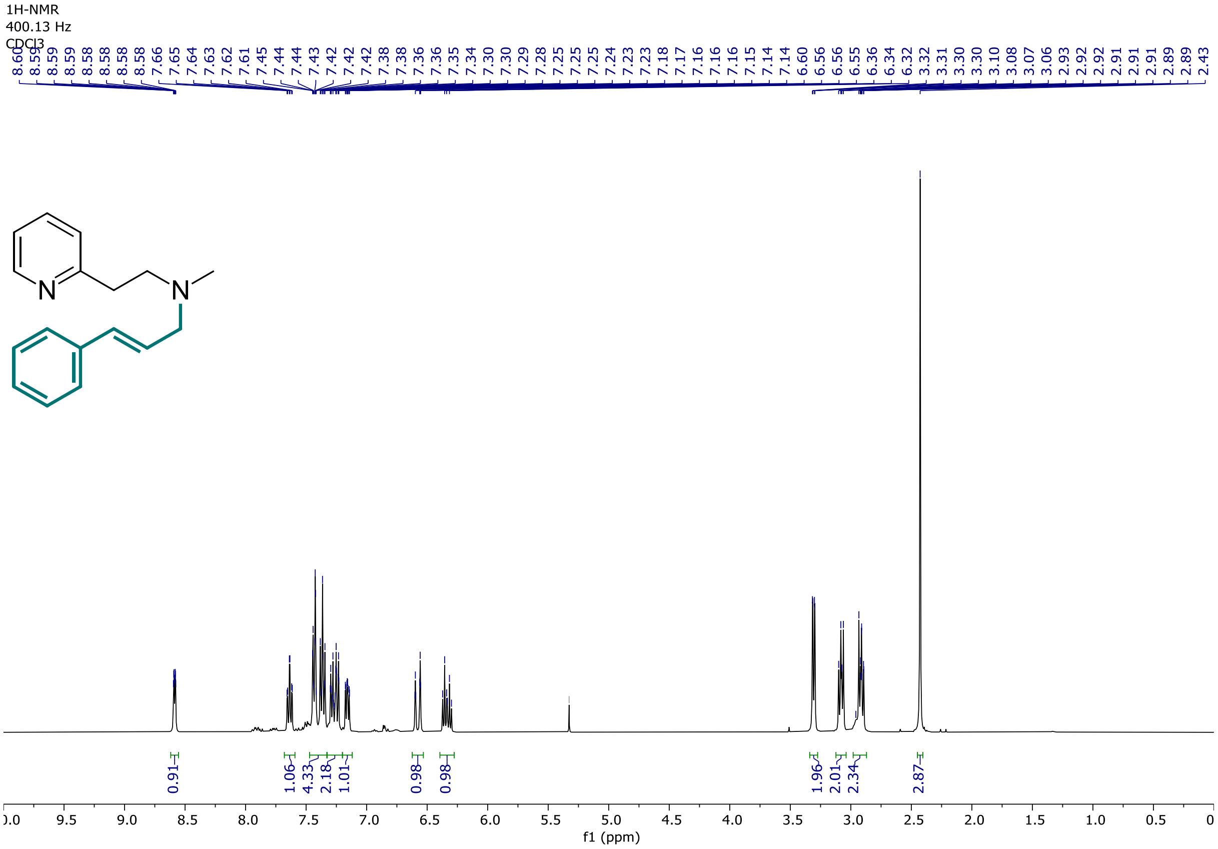

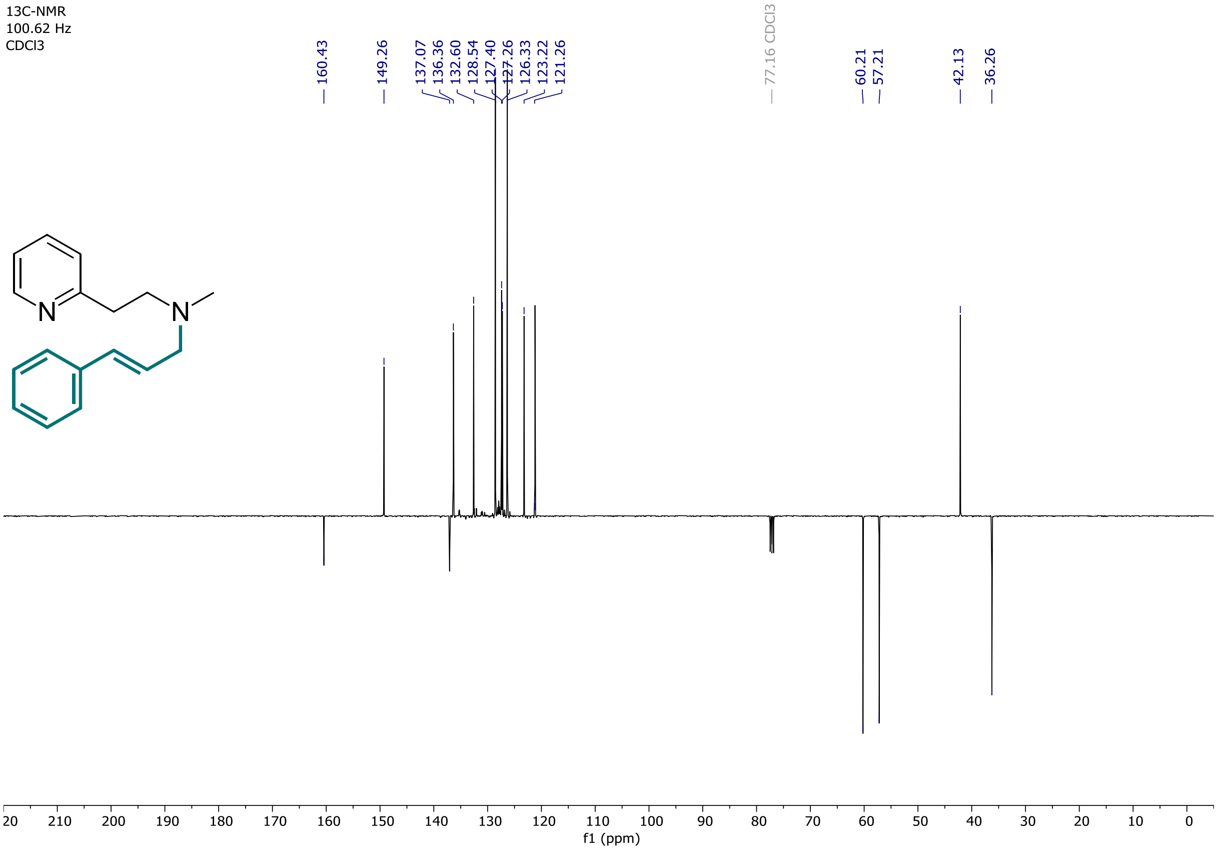

Ethyl [(E)-3-phenyl-2-propenyl]benzoylacetate **(50)**

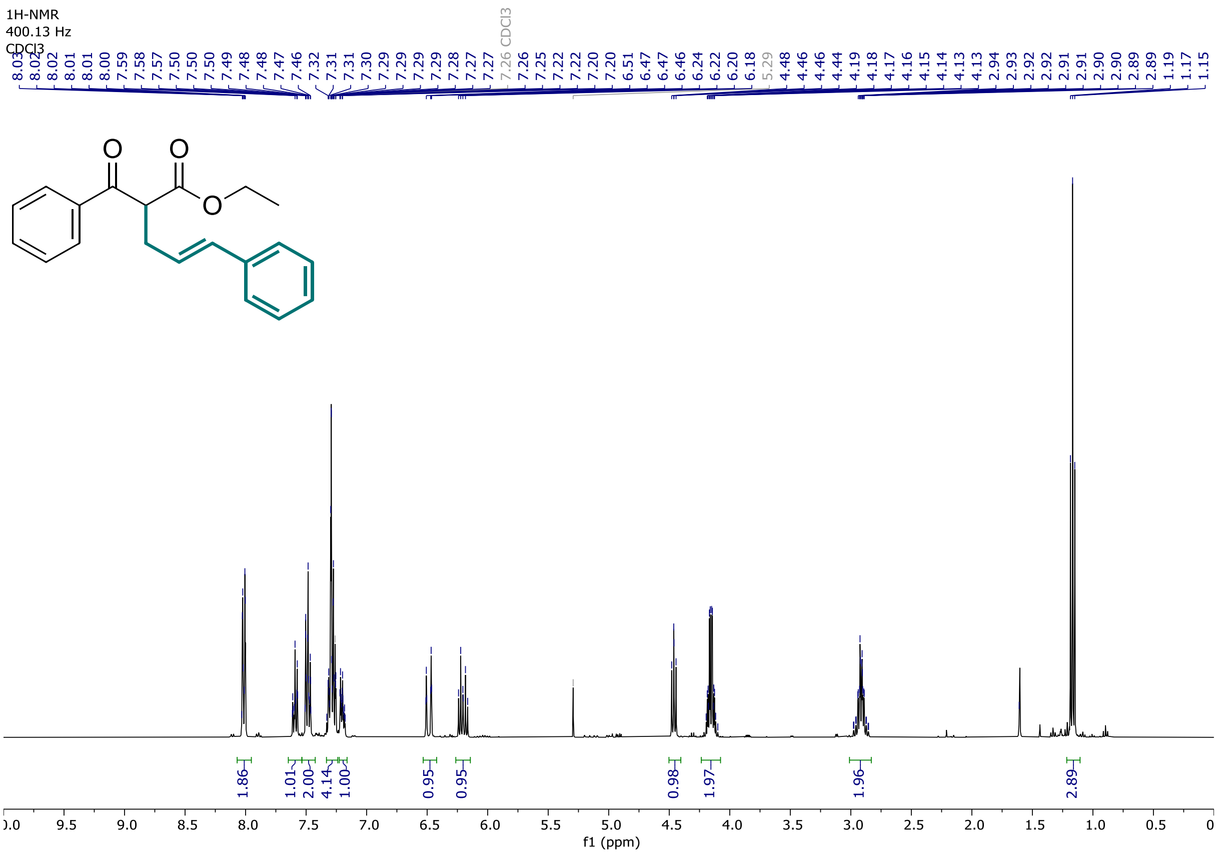

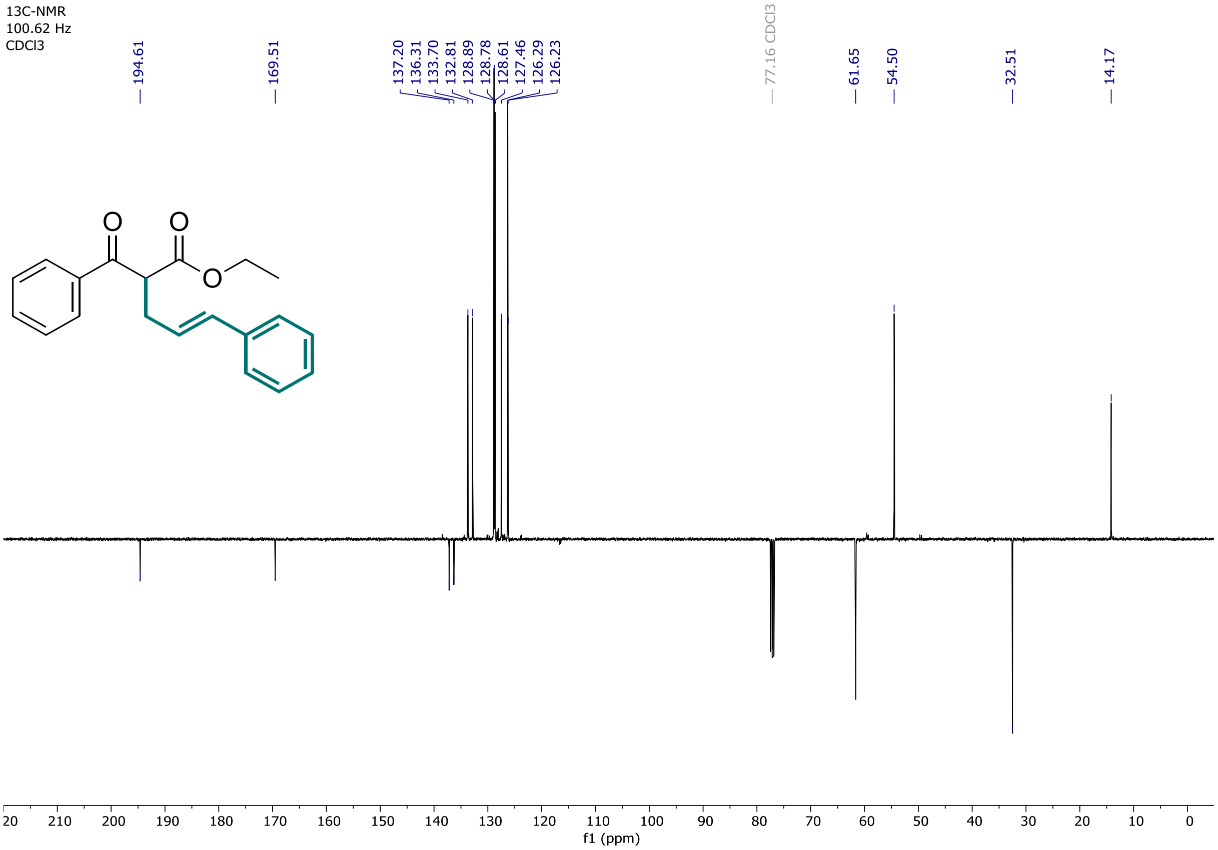

Ethyl *(E)*-2-benzoyl-8,8-dimethyl-4-nonen-6-ynoate **(51)**

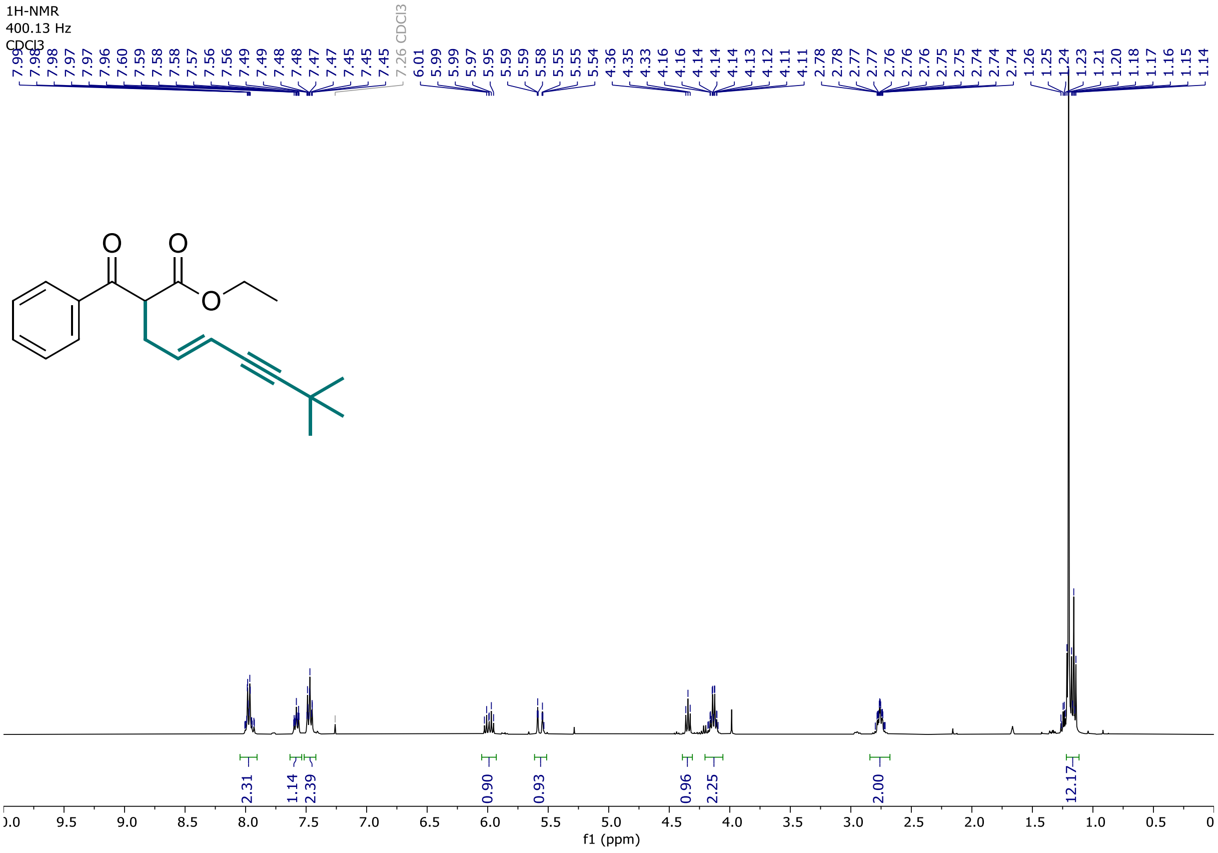

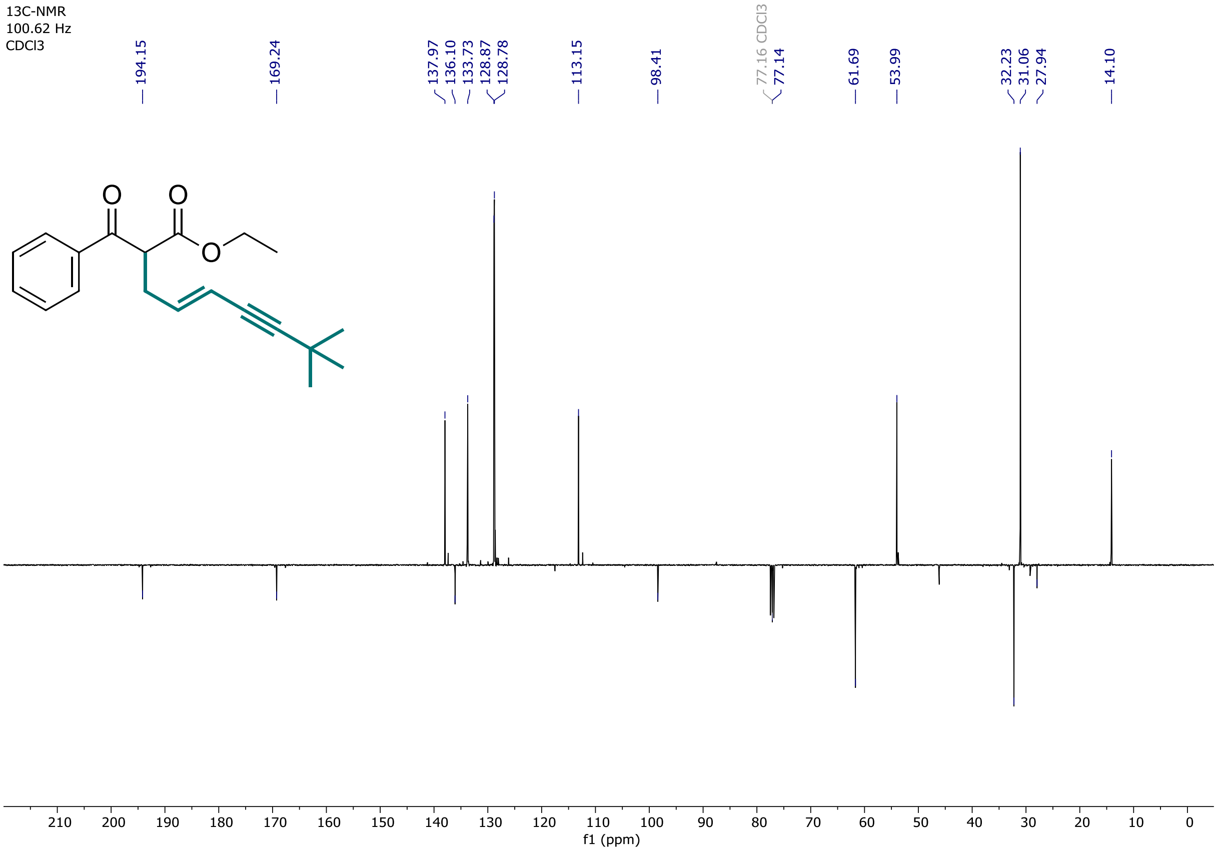

Chromatograms for Chiral HPLC for the synthesis of (R)-/(S)-42 grouped by ligands used.

***rac*-BINAP**

Instrument:U3000   Sequence:Tips lacton   Page 1 of 1

Chromatogram and Results

Injection Name:	**rac-BINAP**	*Run Time (min):*	**30,00**
Injection Volume:	**1,00**	*Channel:*	**UV_VIS_3**
Column:	**OD-2**	*Wavelength:*	**254,0**

Integration Results

No.	Peak Name	Retention Time min	Area mAU*min	Height mAU	Relative Area %	Relative Height %	Amount
1	(R)-43	10,873	27,498	108,504	50,20	55,24	n.a.
2	(S)-43	13,698	27,281	87,920	49,80	44,76	n.a.
**Total:**			**54,779**	**196,424**	**100,00**	**100,00**	

Chromatogram

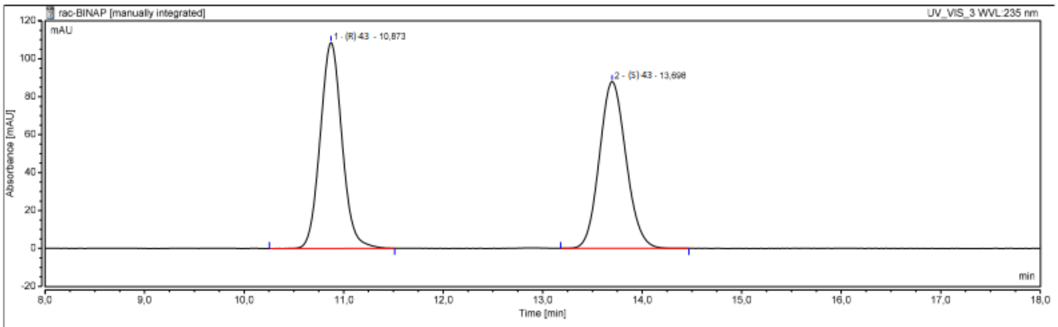

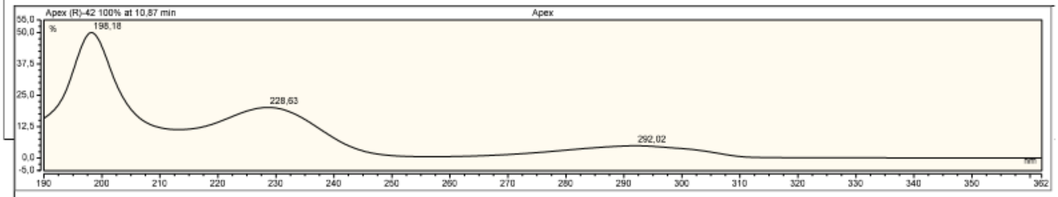

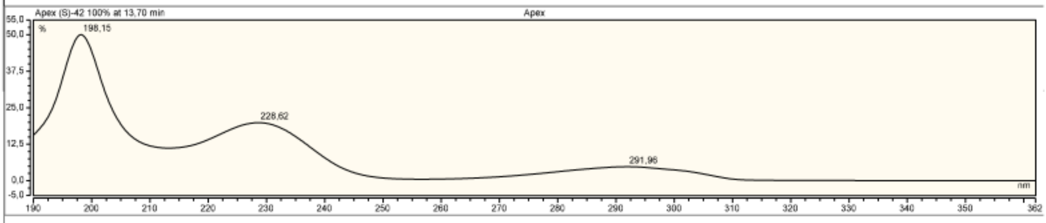

(R)-BINAP

Chromatogram and Results

Injection Name:	**(R)-BINAP**	*Run Time (min):*	**30,00**
Injection Volume:	**1,00**	*Channel:*	**UV_VIS_3**
Column:	**OD-2**	*Wavelength:*	**254,0**

Integration Results

No.	Peak Name	Retention Time min	Area mAU*min	Height mAU	Relative Area %	Relative Height %	Amount
1	(R)-43	10,860	18,764	75,350	35,89	40,83	n.a.
2	(S)-43	13,687	33,512	109,218	64,11	59,17	n.a.
**Total:**			**52,277**	**184,568**	**100,00**	**100,00**	

Chromatogram

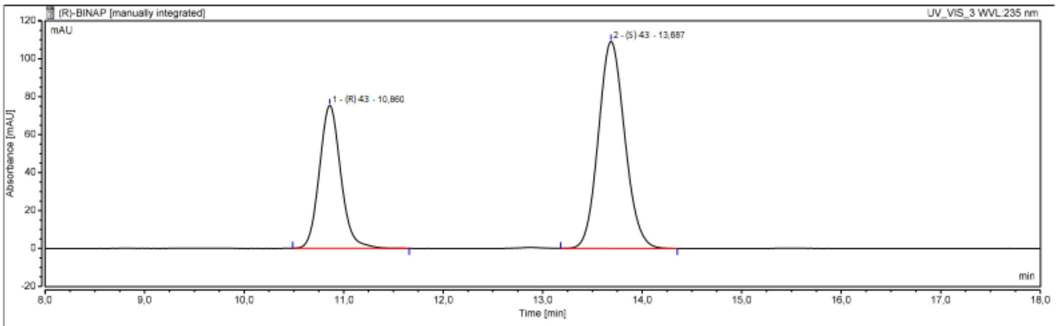

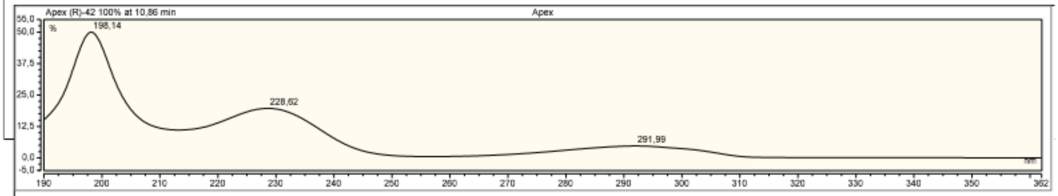

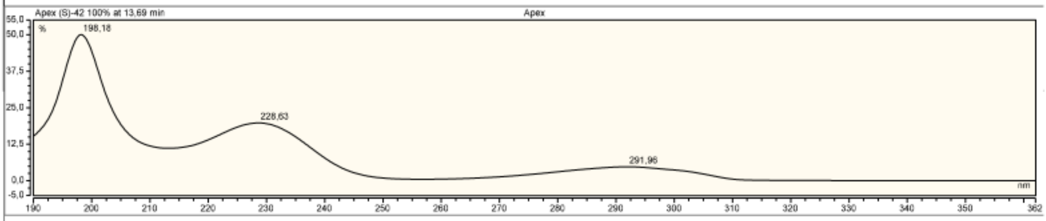

(S)-tol-BINAP

Chromatogram and Results

Injection Name:	**(S)-tol-BINAP**	*Run Time (min):*	**30,00**
Injection Volume:	**1,00**	*Channel:*	**UV_VIS_3**
Column:	**OD-2**	*Wavelength:*	**254,0**

Integration Results

No.	Peak Name	Retention Time min	Area mAU*min	Height mAU	Relative Area %	Relative Height %	Amount
1	(R)-43	10,737	22,360	91,582	61,32	66,00	n.a.
2	(S)-43	13,485	14,106	47,173	38,68	34,00	n.a.
**Total:**			**36,466**	**138,755**	**100,00**	**100,00**	

Chromatogram

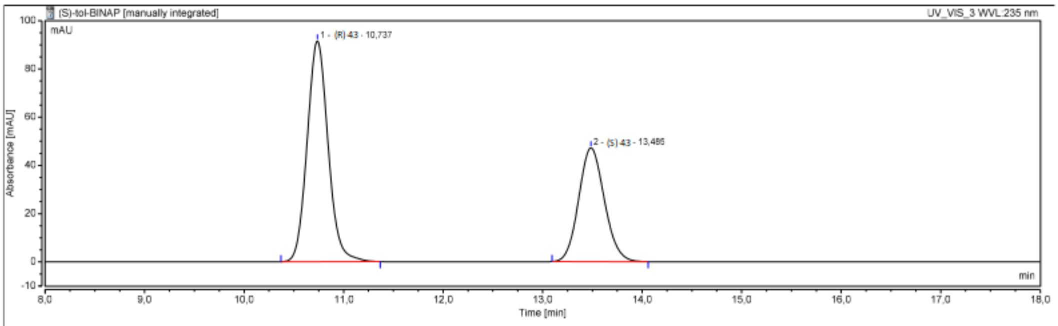

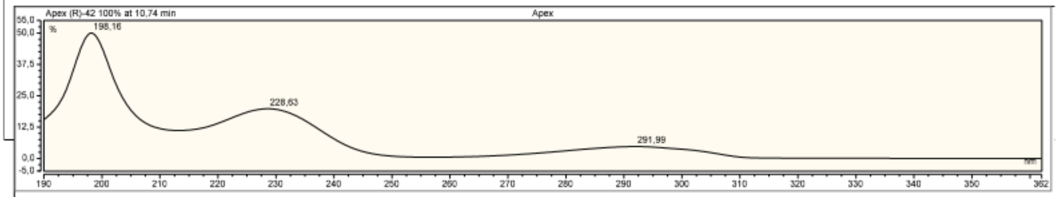

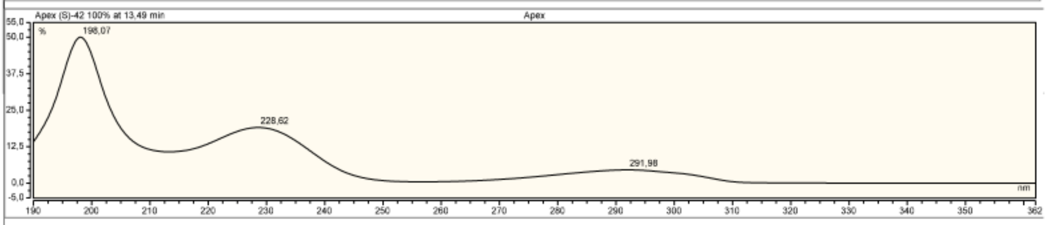

***(1S,2S)*-DACH Trost**

Chromatogram and Results

Injection Name:	**(1S,2S)-DACH-Trost**	*Run Time (min):*	**30,00**
Injection Volume:	**1,00**	*Channel:*	**UV_VIS_3**
Column:	**OD-2**	*Wavelength:*	**254,0**

Integration Results

No.	Peak Name	Retention Time min	Area mAU*min	Height mAU	Relative Area %	Relative Height %	Amount
1	(R)-43	10,862	20,903	83,716	45,93	50,11	n.a.
2	(S)-43	13,610	24,608	83,337	54,07	49,89	n.a.
**Total:**			**45,511**	**167,053**	**100,00**	**100,00**	

Chromatogram

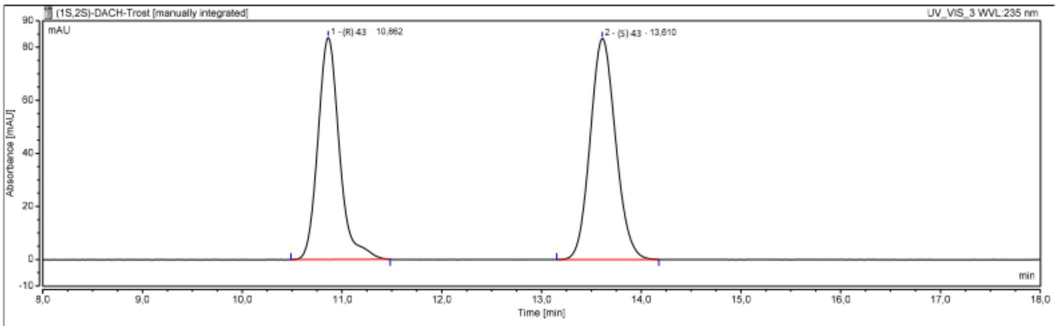

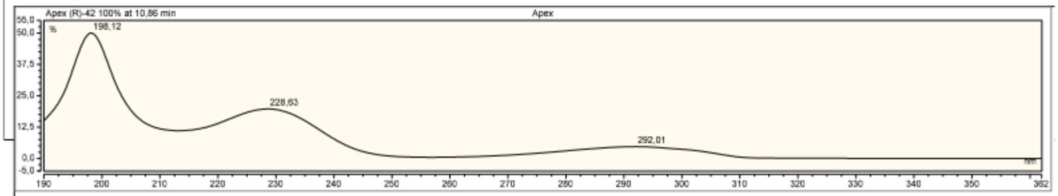

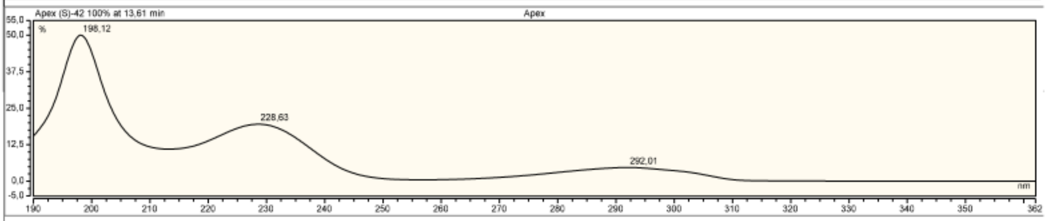

(R)-SEGPHOS

Chromatogram and Results

Injection Name:	**(R)-SEGPHOS ligand**	*Run Time (min):*	**30,00**
Injection Volume:	**1,00**	*Channel:*	**UV_VIS_3**
Column:	**OD-2**	*Wavelength:*	**254,0**

Integration Results

No.	Peak Name	Retention Time min	Area mAU*min	Height mAU	Relative Area %	Relative Height %	Amount
1	(R)-43	10,887	24,543	92,664	75,82	78,01	n.a.
2	(S)-43	13,635	7,826	26,124	24,18	21,99	n.a.
**Total:**			**32,369**	**118,788**	**100,00**	**100,00**	

Chromatogram

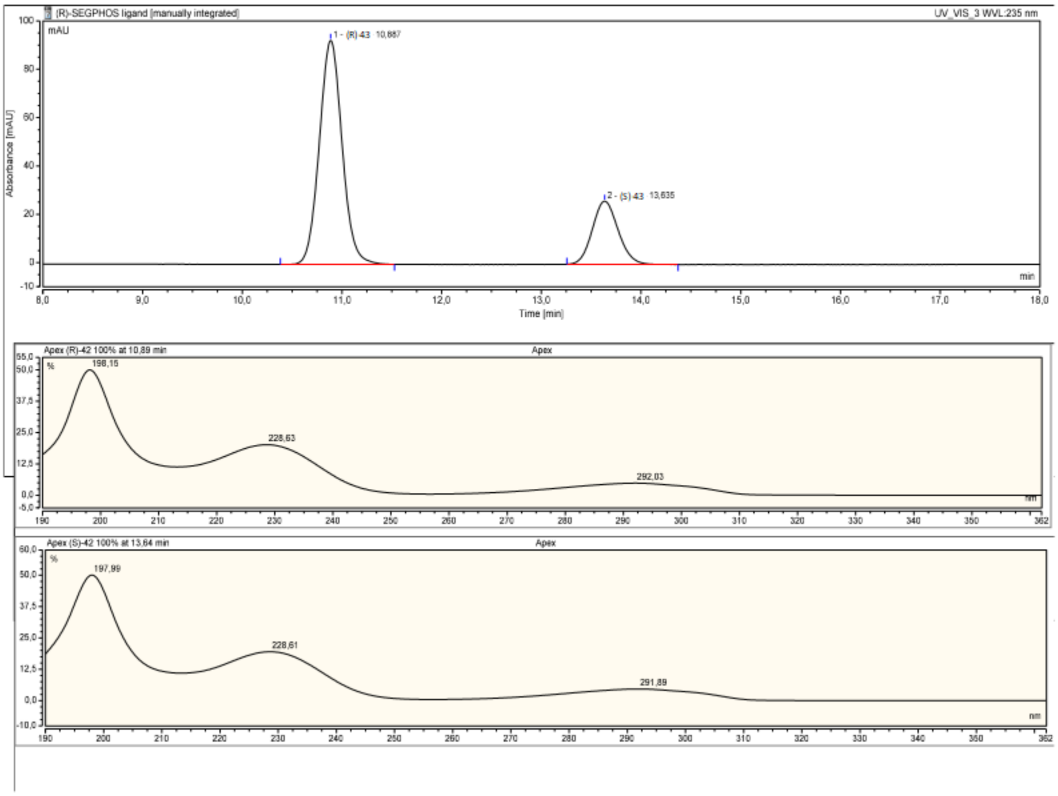

Chromeleon (c) Dionex
Version 7.1.1.1127

List of references

[20] S. V. Sieger, I. Lubins, B. Breit, *ACS Catalysis* **2022**, *12*, 11301-11305.

[27] a) B. M. Trost, M. R. Machacek, H. C. Tsui, *Journal of the American Chemical Society* **2005**, *127*, 7014-7024; b) B. M. Trost, F. D. Toste, *Journal of the American Chemical Society* **1998**, *120*, 815-816.

[28] J. Wang, Y. Wang, G. Ding, X. Wu, L. Yang, S. Fan, Z. Zhang, X. Xie, *Tetrahedron Letters* **2021**, *81*, 153341.

[29] T. Arndt, A. Raina, M. Breugst, *Chemistry – An Asian Journal* **2023**, *18*, e202201279.

[30] S. Bhuyan, A. Gogoi, J. Basumatary, B. Gopal Roy, *European Journal of Organic Chemistry* **2022**, *2022*, e202200148.

[31] I. Triandafillidi, M. G. Kokotou, C. G. Kokotos, *Organic Letters* **2018**, *20*, 36-39.

[32] R. A. Fernandes, S. S. Yadav, P. Kumar, *Organic & Biomolecular Chemistry* **2022**, *20*, 427-443.

[33] A. A. Folgueiras-Amador, A. E. Teuten, M. Salam-Perez, J. E. Pearce, G. Denuault, D. Pletcher, P. J. Parsons, D. C. Harrowven, R. C. D. Brown, *Angewandte Chemie International Edition* **2022**, *61*, e202203694.

[34] N. R. Lee, F. A. Moghadam, F. C. Braga, D. J. Lippincott, B. Zhu, F. Gallou, B. H. Lipshutz, *Organic Letters* **2020**, *22*, 4949-4954.

[35] P. M. Kathe, A. Berkefeld, I. Fleischer, *Synlett* **2021**, *32*, 1629-1632.

[36] J. Wu, M. C. Kozlowski, *Organic Letters* **2023**, *25*, 907-911.

[37] J. Liu, J. Cui, F. Vilela, J. He, M. Zeller, A. D. Hunter, Z. Xu, *Chemical Communications* **2015**, *51*, 12197-12200.

[38] E. D. D. Calder, S. A. I. Sharif, F. I. McGonagle, A. Sutherland, *The Journal of Organic Chemistry* **2015**, *80*, 4683-4696.

[39] Y. Kayaki, T. Koda, T. Ikariya, *The Journal of Organic Chemistry* **2004**, *69*, 2595-2597.

[40] S. Bag, R. Jayarajan, R. Mondal, D. Maiti, *Angewandte Chemie International Edition* **2017**, *56*, 3182-3186.

[41] P. Xu, F. Wang, G. Fan, X. Xu, P. Tang, *Angewandte Chemie International Edition* **2017**, *56*, 1101-1104.

[42] M. L. N. Rao, R. J. Dhanorkar, *European Journal of Organic Chemistry* **2014**, *2014*, 5214-5228.

[43] K. A. Bahou, D. C. Braddock, A. G. Meyer, G. P. Savage, Z. Shi, T. He, *The Journal of Organic Chemistry* **2020**, *85*, 4906-4917.

[44] S. Kyasa, R. N. Meier, R. A. Pardini, T. K. Truttmann, K. T. Kuwata, P. H. Dussault, *The Journal of Organic Chemistry* **2015**, *80*, 12100-12114.

[45] L. W. Lawrence Woo, B. Leblond, A. Purohit, B. V. L. Potter, *Bioorganic & Medicinal Chemistry* **2012**, *20*, 2506-2519.

[46] Z. Chang, T. Ma, Y. Zhang, Z. Dong, H. Zhao, D. Zhao, *Molecules* **2020**, *25*, 1233.

[47] F. Zhao, X.-W. Gu, R. Franke, X.-F. Wu, *Angewandte Chemie International Edition* **2022**, *61*, e202214812.

[48] B. Huang, L. Guo, W. Xia, *Green Chemistry* **2021**, *23*, 2095-2103.

[49] R. Shimazumi, R. Tanimoto, T. Kodama, M. Tobisu, *Journal of the American Chemical Society* **2022**, *144*, 11033-11043.

[50] A. Dierks, L. Fliegel, M. Schmidtmann, J. Christoffers, *European Journal of Organic Chemistry* **2020**, *2020*, 7164-7175.

[51] C. Zhang, R. Su, Y. Guo, D. Li, J. Zhao, Y. Zhao, T. Yu, *Journal of Molecular Structure* **2022**, *1262*, 133062.

[52] S. Govaerts, L. Angelini, C. Hampton, L. Malet-Sanz, A. Ruffoni, D. Leonori, *Angewandte Chemie International Edition* **2020**, *59*, 15021-15028.

[53] H.-J. Zhang, B. Demerseman, Z. Xi, C. Bruneau, *Advanced Synthesis & Catalysis* **2009**, *351*, 2724-2728.

[54] Y. Jiang, C.-M. Park, T.-P. Loh, *Organic Letters* **2014**, *16*, 3432-3435.

[55] A. J. Grenning, J. A. Tunge, *Organic Letters* **2010**, *12*, 740-742.

[56] H. Li, H. Chen, Y. Zhou, J. Huang, J. Yi, H. Zhao, W. Wang, L. Jing, *Chemistry – An Asian Journal* **2020**, *15*, 555-559.
